# Environmental Filters Structure Cushion Bogs’ Floristic Composition along the Southern South American Latitudinal Gradient

**DOI:** 10.3390/plants13162202

**Published:** 2024-08-09

**Authors:** Felipe Figueroa-Ponce, Luis Felipe Hinojosa

**Affiliations:** Laboratory of Paleoecology, Department of Ecological Science, Faculty of Science, University of Chile, Santiago 7800003, Chile

**Keywords:** bogs, environmental filters, Andes, phylogenetic diversity

## Abstract

The environmental filtering hypothesis predicts that abiotic factors restrict communities by selecting species capable of survival and persistence under specific conditions, resulting in variations in beta diversity, phylogenetic clustering, and niche differentiation among communities when studying environmental gradients. Cushion bogs and high-altitude wetlands along the Andes display homogeneous flora contrasting with zonal vegetation. Despite being influenced by microclimatic conditions, these ecosystems are subject to diverse environmental effects. Here, we test the environmental filtering hypothesis on the structure of cushion bog communities along a broad-scale latitudinal gradient from 15° S to 42° S. We analyzed 421 bogs and 293 species across three macroclimatic regions with distinct summer, winter, and transitional arid rainfall regimes. Using variance partitioning and membership-based regionalization models, we examined the impacts of climatic, edaphic, and spatial variables on beta diversity. We also assessed species’ niche overlap and the influence of environmental filters on the communities’ phylogenetic diversity. Results show that species turnover and niche overlap vary with macroclimatic differences, delineating three distinct regions. Notably, phylogenetic clustering in the driest part of the gradient (23° S–24° S) highlights the impact of environmental filtering. Aridity and temperature variations at a broad scale serve as environmental filters shaping the composition of bog communities across southern South America.

## 1. Introduction

Environmental filtering (EF) is a widely used concept in ecology to study the processes underlying community assembly [1,2,3,4,5]. It is generally understood as the abiotic factors that restrict communities by selecting species capable of survival and persistence under specific conditions [1,4]. However, distinguishing the effects of abiotic factors from biotic interactions within a community can be challenging, particularly when dealing with observational data on local scales [4,6]. Environmental filtering should be applied along pronounced macroclimatic gradients on large spatial scales, utilizing multiple lines of independent evidence [4,6].

EF influences alpha diversity by selecting species with similar trait values within communities [2,3,7,8]. Consequently, along a large environmental gradient, EF would impact beta diversity between communities [9,10]. To provide insights into the underlying processes driving differences in species composition, beta diversity can be broken down into two components: (1) turnover, which is the replacement of species between sites, associated with niche diversity along the study gradient, and (2) nestedness, which refers to the loss of species from one site, transforming it into a subset of another, linked to spatial variables and dispersal capacity [11,12,13,14]. Therefore, environmental filtering leads to high beta diversity primarily driven by turnover, where distinct environmental conditions shape distinct communities [15,16,17].

Environmental filters also impact the phylogenetic structure of communities, manifesting in patterns of phylogenetic clustering, where community members are more closely related due, for instance, to shared traits for persistence in a particular environment or niche requirements [18,19]. In contrast, in the absence of environmental filters, a pattern of phylogenetic overdispersion emerges, where individuals are not closely related, and biotic interactions predominate [18,20,21,22].

Accordingly, the environmental filtering hypothesis predicts: (1) An increase in beta diversity along a gradient affected by different or contrasting environmental regimes, primarily due to species turnover; (2) Clustering of communities, based on beta diversity along these gradients, differentiating the gradient macroclimatically; (3) Greater overlap of climatic niches among species within regions than between macroclimatic regions; and (4) Phylogenetic clustering in communities within each macroclimatic region.

**Figure 1:**
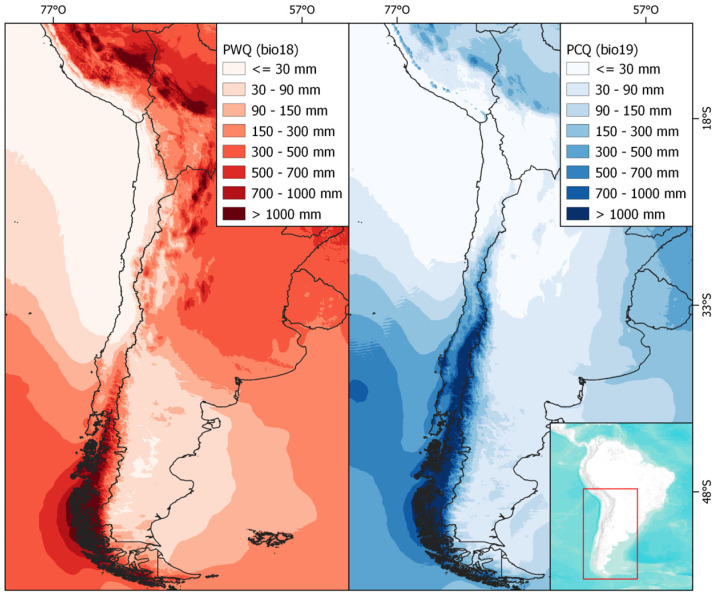
Map of the precipitation regimes in southern South America represented by Precipitation of Warmer Quarter (PWQ) and Precipitation of Coldest Quarter (PCQ). Dotted lines divide the Puna (PU) and Southern Andean steppe (SA) ecosystems.

In southern South America, a biogeographic break is observed near 27° S in the Andes, which divides the ecosystems of the Puna to the north and the Andean steppe to the south [23]. Each is influenced by different precipitation regimes: summer rainfall from easterly winds and winter rainfall from westerly winds, respectively (Figure 1) [23,24]. The aridity resulting from the shift between winter and summer precipitation regimes acts as a barrier that limits north-south dispersal, particularly around 29° S [25]. This phenomenon correlates with a turnover of species, a decrease in richness, and changes in life forms [25,26,27].

Additionally, microclimatic conditions emerge in the context of the interaction between Andean topography and the local environment [28,29], conducive to establishing azonal ecosystems such as cushion bogs [23,25]. These high Andean wetlands, also known as “Bofedales”, “Vegas”, or “Mallines” [30,31], develop near the hydrological and altitudinal limits for plant life in the Andes [30,32] from Colombia/Venezuela to Patagonia [31,33,34]. They are dominated by “bogs-forming” plants, which grow in compact cushions, capable of forming peat, retaining moisture, altering local hydrological conditions, and creating favorable conditions for the colonization of other species [31,35,36]. These plants, mainly Juncaceae like *Oxychloe*, *Distichia*, and *Patosia*, along with Cyperaceae such as *Zameiocirpus* [31], show variations in their distribution. For instance, *Distichia* dominates bogs with a tropical and subtropical distribution from Colombia to northern Chile and Argentina. At the same time, *Oxychloe* is found in subtropical and extratropical distributions from southern Peru to central-southern Chile and Argentina [37,38].

Bogs represent a complex of different species interlocked with each other [39,40,41]. Generally, the flora composing these ecosystems is characterized by rapid vegetative reproduction, high seed production, and both endozoochoric and epizoochoric dispersion [31], as is the case with the dominant Juncaceae, which exhibit clear adaptations for dispersion by birds [25]. Moreover, many of these species demonstrate an anemophilous pollination strategy, and many are considered autogamous [25]. It is proposed that these reproductive characteristics have played a crucial role in the homogeneity of bog flora compared to zonal flora along the latitudinal gradient, regardless of macroclimatic conditions [25,26]. However, bogs are affected by small-scale climatic conditions; for instance, the influence of elevation and temperature on the change in dominance of Juncaceae species has been documented [31,42,43,44,45], as well as the effect of the physicochemical characteristics of associated waters [25,31,33]. Moreover, significant impacts of human activity on floristic composition have been identified [31,39,40], but above all, the aridity-humidity gradients have been proposed as the main factor shaping these communities’ composition [25,26,31,42,43,44,46,47].

Following the environmental filtering hypothesis in addition to the backgrounds on the biogeographic patterns of zonal and azonal vegetation in the Andes, the research question in this work emerges: What environmental filters explain the floristic composition of bogs, azonal vegetation on a large scale along the latitudinal gradient of southern South America? We hypothesize that the variation in precipitation patterns, particularly between the northern macroclimatic areas dominated by summer rains, southern areas dominated by winter rains, and the transition between both precipitation regimes, differentiates the bog communities along the latitudinal gradient of their distribution.

Accordingly, it is predicted: (1) An increase in beta diversity towards the extremes of the latitudinal gradient, primarily due to species turnover; (2) Clustering of communities, based on beta diversity, that differentiates the macroclimatic regions north, transition, and south of the precipitation regimes; (3) Greater overlap of climatic niches among species within regions than between macroclimatic regions; and (4) Phylogenetic clustering in communities within each macroclimatic region. This study aims to determine the influence of environmental filters on the differentiation on a large scale of bog communities along the latitudinal gradient of the Andes.

## 2. Results

### 2.1. Beta Diversity and Environment

#### 2.1.1. Variation Partitioning

**Figure 2:**
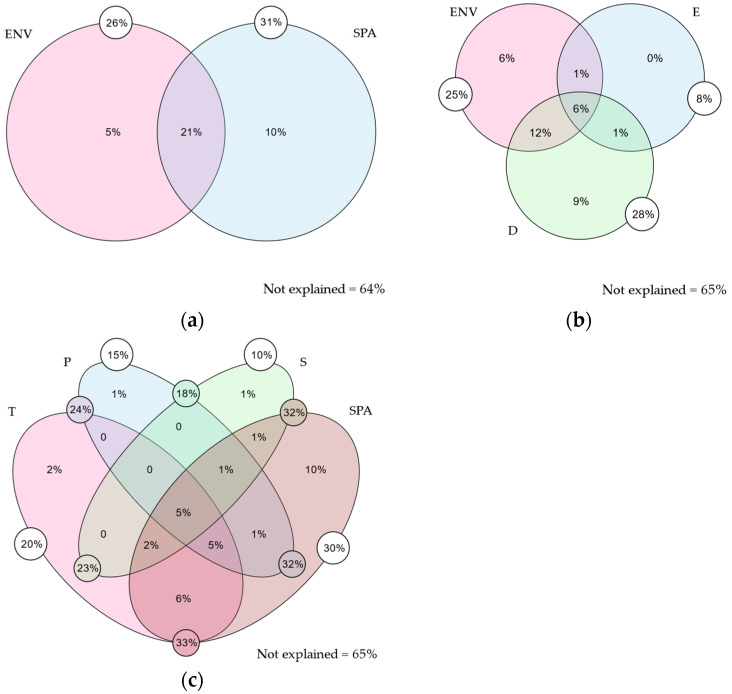
Venn diagrams of the variance partitioning analysis: between environmental and spatial factors (**a**), between separated spatial components and environment (**b**), and between each environmental component and spatial factors (**c**). Percentages inside each circle/oval indicate the pure contributions. The values in the small white circles indicate the total contribution of the variable and percentages in small circles colored according to the intersection indicate the shared contribution. ENV = environment; SPA = spatial; E = elevation; D = geographic distance; T = temperature; P = precipitation; S = soil (see Section 4).

The community composition variation along the gradient is explained by the model by 36% (Figure 2a), attributed to the following factors: (1) pure spatial (10%) and (2) pure environment (5%). Breaking down the spatial components (Figure 2b), 35% of the variation is explained by: (1) pure spatial (9%) and (2) pure environment (6%).

When separating environmental components (Figure 2c), 35% of the variation is explained by (1) pure spatial (10%), (2) pure temperature (2%), (3) pure precipitation (1%), and (4) pure soil (1%).

#### 2.1.2. Beta Diversity and Its Components

**Figure 3:**
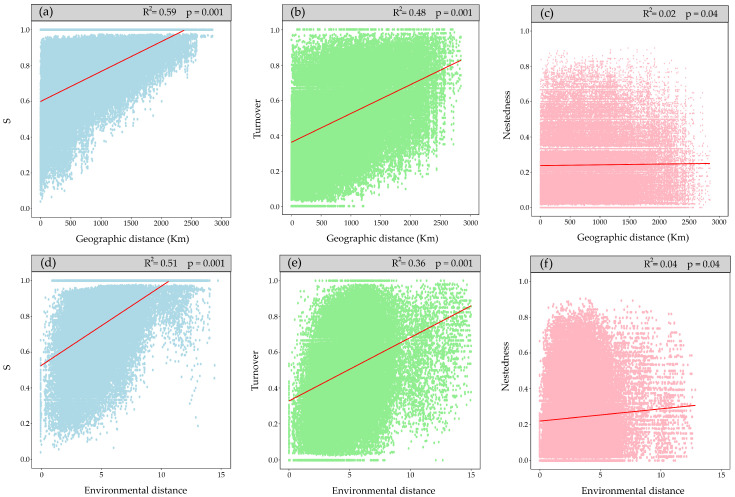
The first row (**a**–**c**) shows the relationships between beta diversity and its components with geographic distance. The second row (**d**–**f**) shows the relationships between beta diversity and its components with environmental distance.

Total beta diversity (BD_total_) indicates a dissimilarity of 0.37, suggesting low species variation along the gradient, with contributions of 68% by the turnover component and 32% by nestedness. Mantel tests (Figure 3) showed that dissimilarity between sites increases with geographic and environmental distance, similarly observed for the turnover component; however, the explanatory power of nestedness was very low (R^2^ of 0.03 and 0.02, respectively; Figure 3c,f).

#### 2.1.3. Clustering and Regionalization

**Figure 4:**
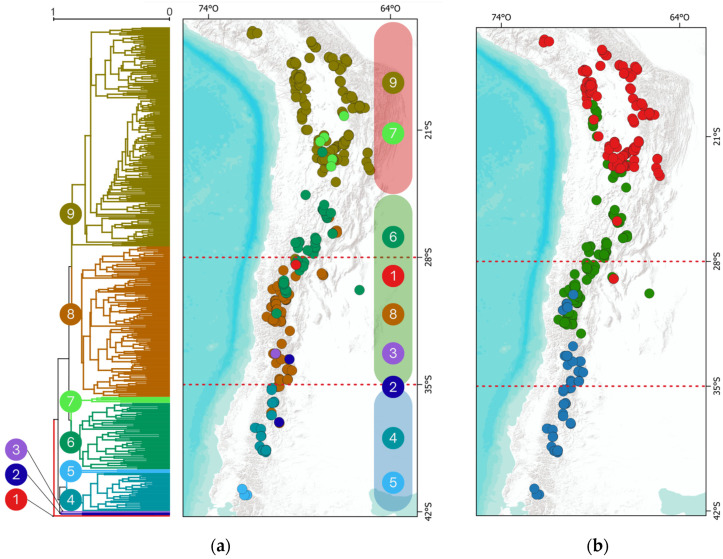
Dendrogram of floristic affinities based on the Sorensen dissimilarity index with its projection onto geographic space in (**a**), where numbers in circles represent the clusters. Regionalization by membership grade model in (**b**), where red circles = North (N); green circles = Transition (T); and blue circles = South (S). The dotted red lines correspond to the operational macro zones defined in this study’s methodology.

The Sorensen index (S) identified nine distinct groups, two consisting of only one site each (Figure 4a). Clusters at the geographical extremes exhibit more significant dissimilarity between them. Certain groups are restricted to specific geographic zones: groups 9 and 7 are exclusively associated with the northern area, while groups 4 and 5 are characteristic of the southern zone. The remaining clusters are heterogeneously grouped in the transition zone.

The regionalization by the Grade of Membership model (Figure 4b) delineates three macro zones, hereafter referred to as bioregions, moving the boundaries of each proposed operational zone northward around 22° S and 32° S.

### 2.2. Niche Overlap

**Figure 5:**
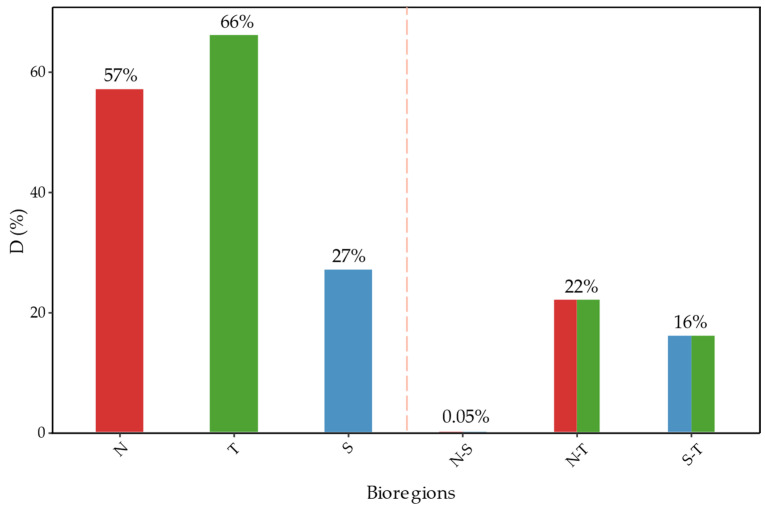
Average “D” Overlap Index as a percentage for species from each bioregion. “N” represents the average overlap among species within the northern bioregion; “T” indicates the average overlap among species within the transition bioregion; “S” refers to the average overlap among species within the southern bioregion. “N-S” denotes the average overlap between species from the north and south bioregions; “N-T” refers to the average overlap between species from the northern and transition bioregions; “S-T” shows the average overlap between species from the southern and transition bioregions.

Greater intra-regional than inter-regional niche overlap was observed, with all comparisons statistically significant (Figure 5). Species in the northern bioregion show an average overlap of 57%, transitional species 66%, while southern species exhibit an average overlap of 27%; however, this low value is due to one species having a limited environmental niche but is almost completely nested within the other two (see “South” in Figure S1).

The lowest average overlap (0.05%) occurs between species from the northern and southern bioregions, differentiated by: (1) Isothermality (Bio3), (2) Annual Temperature Range (Bio7), and (3) Precipitation of the Coldest Quarter (Bio19). The transition zone shows a climatic affinity with the north, with a 22% average overlap, differentiated by (1) Mean Annual Precipitation (Bio12), (2) Isothermality (Bio3), and (3) Annual Temperature Range (Bio7). Between the southern and transitional species, the average overlap is 16%, with main differences in (1) Isothermality (Bio3), (2) Mean Annual Precipitation (Bio12), and (3) Precipitation of the Coldest Quarter (Bio19).

### 2.3. Phylogenetic Diversity

#### 2.3.1. Bioregions

**Figure 6:**
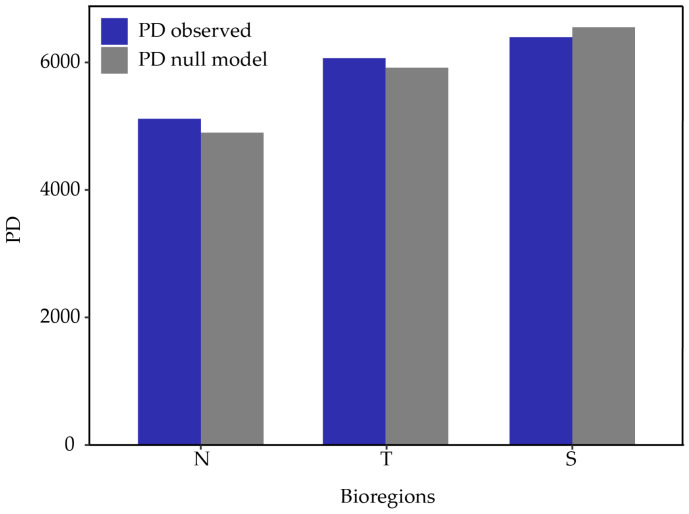
Observed Phylogenetic Diversity (PD) for each bioregion (blue) and expected PD according to the null model (gray).

Observed phylogenetic diversity (PD) shows a north-south increasing pattern (Figure 6), indicating that southern communities are more heterogeneous regarding evolutionary histories from a phylogenetic perspective. Comparing PD metrics, MPD (mean pairwise distance), and MNTD (mean nearest taxon distance) against the null model shows no significant difference indicating clustering or overdispersion at the scale of these bioregions (Table 1).

#### 2.3.2. Latitudinal Bands

**Figure 7:**
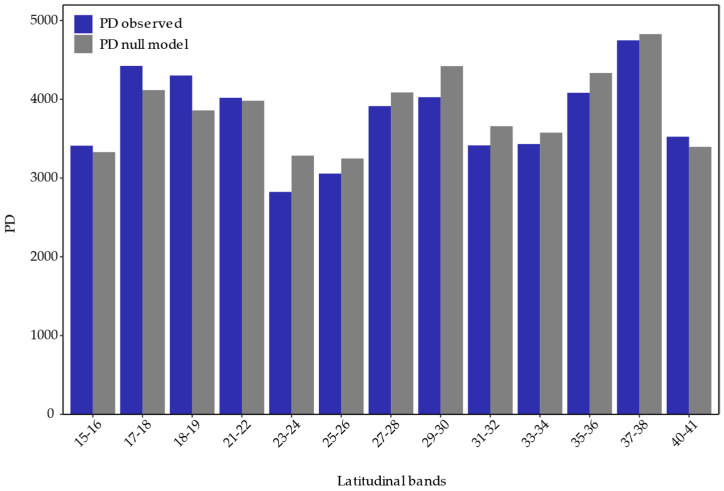
Observed Phylogenetic Diversity (PD) for each 2° latitudinal band (blue) and the expected PD according to the null model (gray).

The increasing observed PD north-south pattern no longer presents; instead, there is a fluctuation along the latitudinal gradient (Figure 7). Comparing PD, MPD, and MNTD against the null model reveals a pattern of overdispersion from 22° S northwards and clustering towards the south, except for the southernmost band. However, it is only significant in the 23° S–24° S band for PD (Table 2), indicating that the species within these communities are more phylogenetically related to each other than by chance, suggesting an environmental filter that restricts them.

## 3. Discussion

### 3.1. Beta Diversity and Environment

#### 3.1.1. Variation Partitioning

The results from the variation partitioning analysis are enlightening in several respects. Firstly, they indicate that spatial factors, understood as limitations to dispersion [48,49], contribute the most on their own (pure spatial), while the isolated contribution from the environment (pure environment) is very low. This suggests that the structuring of the floristic community in bogs is more influenced by landscape configuration and habitat connectivity than by macro-environmental conditions, contrary to studies in other mountainous regions for zonal vegetation [50]. However, their contributions appear more balanced when considering the synergistic effects of environmental and spatial factors (total contributions). This indicates that although spatial factors are crucial, their interaction with the environment cannot be ignored, reflecting that while species may disperse across the landscape, environmental conditions ultimately determine their establishment and persistence.

Secondly, when analyzing the separated spatial components against the environment, geographic distance emerges as the most explanatory factor (pure geographic distance), aligning with the results of the Mantel test (Figure 3a–c). The greater the distance between bogs, the more different the community composition. These results also indicate that the total contribution of elevation is primarily due to its shared effects with other factors, suggesting a correlation between them.

Finally, the detailed analysis of environmental factors revealed that, even though the isolated effects of environmental factors such as pure temperature, pure precipitation, and pure soil variables have a minor or negligible contribution to the structuring of these communities compared to spatial factors, their importance is significantly magnified when considered in interaction with other factors (total contributions). These results suggest that structuring floristic communities in bogs is not governed exclusively by a single environmental or spatial factor but rather by the complex interaction of multiple factors.

Furthermore, 64–65% of the variance remains unexplained in both models, suggesting the influence of unconsidered factors such as biological interactions, the sources and physicochemical characteristics of the waters associated with bogs, topography (slope), and anthropogenic impact variables, all of which have evidenced their impact on floristic composition [30,31,47,51].

#### 3.1.2. Beta Diversity and Its Components

Despite the low BD_total_ value in the results, which could be due to under-sampling, either from original data collection or from species ‘cleaning’ for analysis, it is consistent with previous studies in bogs of South America showing a significant number of species common along the latitudinal gradient [25,26,33,42,46]. This directly impacts the Sorensen dissimilarity index due to the duplication in the importance of shared species, consequently increasing floristic similarity along the gradient. Moreover, the turnover component emerges as the main contributor to beta diversity in the study area, indicating a mechanism of species replacement in response to environmental changes. This aligns with studies by Casagranda and Izquierdo [45], and Méndez [44], which show a turnover in dominant species under colder conditions and at higher altitudes. Arroyo et al. [25] also described species turnover in Andean zonal vegetation, observing that under an aridity gradient, species are replaced by plants more adapted to those conditions. They also noted that bogs, when facing a reduction in area due to aridity, lose fewer species than zonal vegetation, probably due to the constant reintroduction of species through birds and livestock. This explains the low contribution observed by the nestedness component, suggesting that species loss occurs at smaller scales, under gradients of physicochemical water conditions [31,33], or even under human activity, which strongly influences species loss due to the effects of livestock, water extraction, or peat harvesting [42,47].

These explanations also help understand the positive relationships between the dissimilarity of each component (S, turnover, and nestedness) and geographic and environmental distance, a pattern that aligns with other ecosystems, such as tropical forests [52] or relic forests of Chile [53]. The Mantel tests were significant; however, the nestedness (R^2^) explanatory power was very low, revealing no clear pattern. In contrast, the turnover component showed a pattern similar to S, so it was decided to continue working with the latter to avoid overestimating the environmental effect.

#### 3.1.3. Clustering and Regionalization

The hierarchical clustering analysis identified 9 groups based on the Sorensen dissimilarity index, structured similarly to the three proposed operational zones. However, due to the sensitivity of this type of analysis to very homogeneous communities, noisy data, and outliers [54], situations like the one observed occurred, where two groups were formed by a single site each. Additionally, Ruthsatz [46] clarifies that the floristic lists for Chile are incomplete, mainly due to limited time and season, as many plants were observed in the vegetative stage without characteristic organs allowing identification [55,56]. This study used a membership grade model to transform clusters into bioregions. These models offer a more flexible and detailed way to analyze regions and their biotic transitions, particularly useful in cases of gradual transitions and for studies aiming to understand the influence of various factors on regional differences (see [57]). This result, in part, concurs with the hierarchical clustering and with the operational zones defined for this study, with the difference that it pushes the boundaries slightly northward but still coincides with the macroclimatic conditions. It is interesting to observe how the transition bioregion, associated with greater aridity, extends up to northern Chile on the western slope of the Andes, which makes sense since that area corresponds to the “dry Puna,” with a rain shadow effect from the mountain range on the easterly winds [23,24,30].

This grouping into the three bioregions provides evidence of the environmental effect on large-scale community assembly and strongly aligns with the three phytogeographic districts proposed by Biganzoli et al. [58] for the Andean Province of Southern South America, which in turn coincide with the macroclimates present in Chile that extend to Argentina [59].

### 3.2. Niche Overlap

The analysis of niche overlap suggests a significant separation between communities at the extremes of the gradient (North-South). This differentiation is primarily due to factors such as Isothermality, Annual Temperature Range, and Precipitation of the Coldest Quarter. Northern communities are more adapted to stable temperature conditions throughout the year, while southern communities show adaptations to greater temperature variations and more intense precipitation during the coldest month, which are characteristic of the respective macroclimates for the bioregions [23,30]. These findings support the hypothesis that macroclimatic conditions influence these azonal ecosystems, consistent with previous research [31,42,46,47].

The comparison between the transition bioregion and the northern and southern bioregions reveals substantial differences in climatic niches. These differences are mainly attributed to a decrease in annual precipitation in the transition zone. This pattern aligns with multiple studies examining the effects of aridity on the composition of bogs and zonal vegetation [25,26,31,42,46,47,60]. In the transition bioregion, the versatility of species in terms of their climatic niches is notable when compared to those of the north and south. They show a greater affinity for aridity and include temperature variations that separate northern and southern species, likely due to the presence of species from these bioregions in the transition zone, which aligns with observed floristic affinities (Figure 4). This diversity allows them to occupy a broad spectrum within the climatic gradient, consistent with studies that include transition zones in South America [61].

### 3.3. Phylogenetic Diversity

The results from phylogenetic diversity metrics derived from using the three bioregions indicate an observed PD pattern that increases from north to south, consistent with studies on the flora of Chile [62]. However, this pattern is not observed in the analysis conducted by latitudinal bands. This discrepancy is due to high PD between 27° S and 30° S (Figure 7), sites that, according to previous hierarchical clustering and the membership grade model (Figure 4), contain species more related to the communities of the north and south, so greater sampling resolution affects the large-scale PD pattern. The significant standardized effect of PD indicates phylogenetic clustering around 23° S and 24° S, a signal of phylogenetic conservatism in niche preference [62], likely associated with the arid conditions at that latitude, functioning as an environmental filter. Although other results are not significant, both PD, MPD, and MNTD show a clear tendency towards phylogenetic overdispersion north of 23° S and phylogenetic clustering to the south (Table 2), results that contradict observations for the zonal flora of Chile [62] but are consistent with global patterns [63].

These findings should be approached with caution, as many of the species included in the study were not present in the mega-tree used for analysis and were added by inserting the species at the basal node of the genus. If a family or genus has only one representative in the tree, the branch is split to represent taxonomic levels and new species are added at corresponding points of this split (see [64]), reducing the PD. Therefore, it is advisable to work on more detailed phylogenies for high Andean species, such as the phylogeny proposed by Brozova et al. [65] for Juncaceae and Cyperaceae, a phylogeny that has been criticized by some authors (see [66]).

Overall, the results obtained, both in niche overlap and PD analysis, reinforce the predominance of the environmental filter over the role of stochasticity suggested by neutral theory. This theory attributes a fundamental role to ecological drift, random dispersion, and speciation in determining community composition [67]. However, our findings strongly support the niche theory, where environmental factors are key in community assembly [68,69], through the joint effects of the variables studied. Among these variables, dispersal capacity stands out, influenced by spatial factors and determined by mechanisms such as endozoochoric and epizoochoric dispersal, linked to birds and their migratory routes, as well as the movement of livestock and native ungulates like guanacos *(Lama guanicoe)* and vicuñas *(Vicugna vicugna)* [25,31]. Other significant elements are aridity and rainfall regimes, which, while contributing modestly to the variation of taxonomic diversity, are decisive in the climatic characteristics of communities along the latitudinal gradient in southern South America and in the restriction of phylogenetic communities.

In relation to the niche conservatism hypothesis [70], the notable divergence in climatic niches, as well as trends in dispersion and phylogenetic clustering observed in the north and south, respectively, support hypotheses by Cleef [33] and Ruthsatz [42] about different colonization histories for bog plants, with contributions from Antarctic austral origins that advanced as suitable conditions were generated alongside the rise of the Andes, finding taxa in tropical environments with influences from Holarctic species [33,71]. Subsequently, speciation processes with specific adaptations [72] contributed new elements to the mixing zone in the arid conditions of the gradient.

### 3.4. Environmental Filtering

The environmental filtering hypothesis has been widely utilized; however, it is not without controversy [4,6]. A significant point of debate is the challenge of disentangling abiotic effects from biotic interactions within communities, as competition or facilitation can obscure the patterns predicted by environmental filtering, such as phylogenetic clustering [6,73]. Our study addresses this issue using a more inclusive definition of environmental filtering [4,6]. We examined a large-scale study area with pronounced environmental differences along the gradient and utilized independent lines of evidence, such as niche overlap and beta diversity measures [4,6]. On such a scale, abiotic variables are indeed more influential than biotic interactions, even though facilitation, particularly among cushion bog-forming plants, is prevalent in our study [74].

## 4. Materials and Methods

### 4.1. Study Area

**Figure 8:**
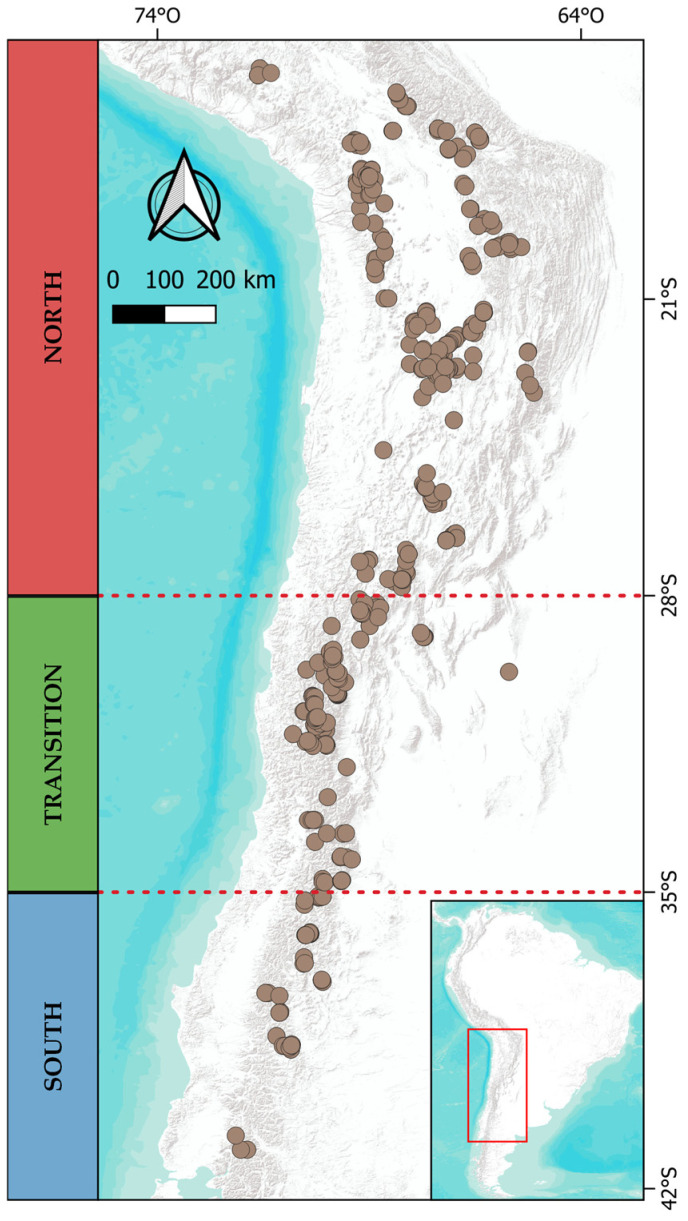
Distribution map of the 421 bogs in southern South America with the macroclimatic regions defined for this study: North (N), Transition (T), and South (S).

The study area includes the eastern and western slopes of the Andes, spanning from 15° S to 42° S across Peru, Bolivia, Argentina, and Chile. The latitudinal gradient was divided into three operational zones (macroclimatic regions) based on rainfall regimes (Figure 8): (1) North, from 15° S to 28° S, influenced by summer rains from the easterly winds; (2) Transition, from 29° S to 35° S, an area between summer and winter rainfall with varying precipitation distribution annually and interannually; and (3) South, from 35° S to 41° S, affected by winter rains from the westerly winds.

### 4.2. Data Preparation

A presence/absence matrix (1 and 0) was constructed using floristic lists from Ruthsatz [31,42,43,46,47] and Monge-Salazar [32], selecting only angiosperms and excluding uncertain identifications marked as “spec”, “cf”, “aff”, and those with two epithets (e.g., *Luzula racemosa/vulcanica*). Varieties and subspecies were recorded under the species of the first epithet, and “sl” (sensu lato) species were retained as such. Nomenclature was updated using the Vascular Plants Catalogue of the Southern Cone [75], the Vascular Plants Catalogue of Chile [37], and the Vascular Plants Catalogue of Bolivia [76], resulting in a total of 421 sites and 293 species (Table S1).

### 4.3. Environmental Variables

Climatic data included the 19 bioclimatic variables from CHELSA 2.1 for the period 1981–2010, proven effective in mountainous regions [77,78,79]. Elevation data was extracted from CHELSA 2.1 [77], and 9 edaphic variables at a depth of 60–100 cm were obtained from ISRIC-World Soil Information [80]. All variables were used at a 30” resolution (Table 3).

### 4.4. Data Analysis

#### 4.4.1. Variation Partitioning

Using the software R 4.3.2 [81], the environmental variables (climatic + edaphic) set was optimized by excluding those with a correlation coefficient > 0.75, preferring those with the highest contribution in a principal components analysis. Geographic distance between sites was calculated using the distm function from the geosphere 1.5–18 library [82], and Moran’s Eigenvalues (MEM) were computed for each site. From the complete set of variables (environmental + MEM + elevation), those with a significant effect were selected using the forward selection method, applied to the raw presence/absence matrix via the forward.sel function of the adespatial library (with 999 permutations and alpha = 0.5) [83].

All variable values were normalized to ensure comparability (Table 4). This allowed for a variance partitioning analysis, decomposing the contribution of environmental (temperature + precipitation + soil) and spatial components (geographic distance through Moran’s Eigenvector Maps + elevation) to the original presence/absence matrix see [84,85,86,87]. The contribution of each environmental and spatial variable was evaluated using the varpart function of the vegan library [88], with significance tested through ANOVA.

#### 4.4.2. Beta Diversity and Its Components

Beta diversity among sites was assessed using the Sorensen dissimilarity index from the presence/absence matrix. Total beta diversity (BDtotal), corresponding to the total variance of the community matrix (see [89]), was calculated and partitioned into its components of turnover (ReplS) and nestedness (RichDiffS), as shown in Table 5, using Podani family indices [12] with the beta.div.com function of the adespatial 0.3–23 library [83].

The relationship between beta diversity and the environment was analyzed through a permutation-based forward selection analysis using non-correlated environmental variables (climatic and edaphic), as shown in Table 6, transforming them into Euclidean distance matrices via the vegan 2.6–4 library [88]. A Mantel test was then used to analyze the correlation between environmental and geographic distance with beta diversity and its components, using the mantel function of the vegan library [88].

#### 4.4.3. Clustering and Regionalization

Based on beta diversity, a hierarchical clustering analysis was conducted on sites to identify clusters along the latitudinal gradient. The dendrogram was constructed using the UPGMA clustering algorithm, with an optimal number of clusters set to nine. The best clustering algorithm was selected using the select_linkage function based on the cophenetic correlation coefficient of Sokal and Rohlf [91], and the optimal number of clusters was determined using the “elbow” method (see [92,93]) from the function optimal_phyloregion, both from the phyloregion 1.0.8 library [94]. However, the hierarchical clustering resulted in numerous clusters, some consisting of only a single community, indicating potential over-segmentation.

To address these issues and validate the clustering results, a membership grade model was then developed to regionalize the sites into three areas (based on the proposed macroclimatic regions) using the fitgom function of the phyloregion library [94]. This model is advantageous over hierarchical methods as it allows for partial memberships, reflecting the gradual transitions often observed in ecological data, and does not assume hierarchical nestedness of biotic structure [95] (White et al., 2019). This process involved fitting the model to the original presence/absence matrix and assigning bioregions based on the probability of each site belonging to each bioregion, considering taxonomic diversity (see [57]).

#### 4.4.4. Niche Overlap

To measure niche overlap, three representative species from each bioregion were selected using the “Kullback-Leibler divergence” method (Table 7) with the indicators function from the phyloregion library [94]. Occurrences for each species were obtained from the Global Biodiversity Information Facility (GBIF) [96], with anomalous data, country centroids, and museum or research center records filtered using the CoordinateCleaner 3.0.1 library [97].

In the analysis, one occurrence per climatic grid (~1 km^2^) was filtered. The 19 bioclimatic variables and 9 edaphic variables (Table 1) were used, removing those with a correlation coefficient > 0.75, prioritizing the most influential according to a principal component analysis (Table 8 and Table S2). Niche overlap was calculated using Schoener’s D index [98] with the ecospat 4.0.0 library [99]. The significance of the results was assessed through a niche equivalence test, based on the D index, comparing the observed value with a distribution of values obtained from random samples. Comparisons were made between the three species from each bioregion and with those from other bioregions, generating three D values for each zone (north-north, transition-transition, and south-south) and nine between each comparison (north-transition, north-south, and south-transition).

#### 4.4.5. Phylogenetic Diversity

To assess phylogenetic diversity metrics [100], the mega-tree included in the V.PhyloMaker2 library [101] was pruned. Two scales were established for analysis: the first corresponds to the three bioregions resulting from the membership grade model, and the second involves latitudinal bands of 2°. The standardized effect of phylogenetic diversity (PD), mean pairwise distance (MPD), and mean nearest taxon distance (MNTD) was analyzed in both contexts. The observed metrics were compared with a null model that randomizes species composition in the community using the Picante 1.8.2 library [102]. Positive and significant values indicate phylogenetic overdispersion, while negative and significant values indicate phylogenetic clustering. The main distinction between MPD and MNTD lies in the analysis encompassing deeper levels of the tree (orders and families) and towards the branch tips (genera and species), respectively [18].

## 5. Conclusions

This study provides a comprehensive assessment of how macro-environmental variables function as filters in the differentiation of communities in bogs along the Andes (15° S–41° S). The findings indicate low total beta diversity across this gradient, primarily influenced by dispersal limitations and macro-environmental conditions. Three distinct bioregions were identified based on taxonomic diversity, corresponding to the macroclimates of Chile and the phytogeographic districts of the high Andean province of southern South America.

Notably, species at the extremes of the north-south gradient exhibited significant differences in their climatic niches, with a broader niche width in the transition zone. Phylogenetic metric analyses indicate clustering between rainfall regimes in the arid transition zone, reflecting phylogenetic conservatism in niche preference.

In conclusion, this study validates the proposed hypothesis by identifying a clear separation of communities in the transition of rainfall regimes. However, it adds temperature variation as an influential factor in community formation. Significantly, macro-environmental conditions exert a considerable effect on the biodiversity of azonal flora in the Southern Andes of South America, playing a critical role in shaping these unique communities.

## Figures and Tables

**Table 1:** Standardized effects of PD, MPD, and MNTD compared to the null model (z-score) for each bioregion with statistical significance (α).

Bioregion	PD z-Score	αPD	MPD z-Score	αMPD	MNTD z-Score	αMNTD
N	0.801	0.783	1.865	0.987	0.481	0.687
T	0.612	0.703	1.504	0.961	0.239	0.593
S	−0.521	0.312	−1.250	0.148	−0.564	0.282

**Table 2:** Standardized PD, MPD, and MNTD effects relative to the null model (z-score) for each 2° latitudinal band with statistical significance (α) indicated in bold.

Band	PD z-Score	αPD	MPD z-Score	αMPD	MNTD z-Score	αMNTD
15–16	0.318	0.636	1.437	0.843	0.368	0.647
17–18	1.129	0.860	1.791	0.940	1.192	0.872
18–19	1.728	0.951	2.125	0.974	1.742	0.950
21–22	0.042	0.542	1.838	0.949	0.183	0.602
23–24	**−1.825**	**0.015**	−0.890	0.134	−1.327	0.091
25–26	−0.717	0.251	−0.480	0.340	−0.489	0.325
27–28	−0.565	0.296	−0.635	0.300	−0.746	0.244
29–30	−1.408	0.082	−0.294	0.502	−1.448	0.065
31–32	−0.793	0.226	−0.852	0.173	−0.292	0.411
33–34	−0.514	0.306	−0.109	0.609	−0.789	0.222
35–36	−0.885	0.193	−0.575	0.319	−1.293	0.089
37–38	−0.309	0.404	−0.745	0.258	0.196	0.581
40–41	0.485	0.693	−0.335	0.436	0.610	0.730

**Table 3:** Environmental variables and their abbreviations used in this study.

Type	Variable (Abbreviation)	
Climate	Annual Mean Temperature (Bio1)	Mean Temperature of Coldest Quarter (Bio11)
	Mean Diurnal Range (Bio2)	Annual Precipitation (Bio12)
	Isothermality (Bio3)	Precipitation of Wettest Month (Bio13)
	Temperature Seasonality (Bio4)	Precipitation of Driest Month (Bio14)
	Max Temperature of Warmest Month (Bio5)	Precipitation Seasonality (Bio15)
	Min Temperature of Coldest Month (Bio6)	Precipitation of Wettest Quarter (Bio16)
	Temperature Annual Range (Bio7)	Precipitation of Driest Quarter (Bio17)
	Mean Temperature of Wettest Quarter (Bio8)	Precipitation of Warmest Quarter (Bio18)
	Mean Temperature of Driest Quarter (Bio9)	Precipitation of Coldest Quarter (Bio19)
	Mean Temperature of Warmest Quarter (Bio10)	
Edaphic	Soil organic carbon in fine earth (Soc)	Total nitrogen (Nitrogen)
	Bulk density of the fine earth fraction (Bdod)	Vol. fraction of coarse fragments (>2 mm) (Cfvo)
	pH H_2_O (Phh2o)	Organic Carbon density (Ocd)
	Silt (Silt)	Sand (Sand)
	Clay (Clay)	
Elevación	Digital elevation model (Elev)	

**Table 4:** Environmental variables after correlation analysis and forward selection used for variation partitioning.

	Type	Variables		
Environment	Temperature	Bio2		
		Bio7		
		Bio9		
		Bio10		
		Bio11		
	Precipitation	Bio15		
		Bio18		
	Edaphic	Bdod		
		Phh2o		
		Cfvo		
Spatial	Elevation	Elev		
	MEM	4	1	3
		5	9	8
		2	12	51
		19	17	10
		32	59	28
		11	37	6
		16	40	24
		23	20	33
		22	52	42
		31	36	

**Table 5:** Equations used for calculating beta diversity. In (1), (3), and (4), a = species shared by the compared sites; b = species present exclusively in one site; and c = species present exclusively in the other site. In (2), n = sites in the dissimilarity matrix, created from Equation (1); D^2^_hi_ = √S between each site.

Index	Equation	Source	
S (Sorensen)	$\frac{b+c}{2a+b+c}$	(Chao et al., 2006) [90]	(1)
BDtotal	$\frac{1}{n}\overset{n-1}{\underset{h=1}{\sum }}\overset{n}{\underset{i=h+1}{\sum }}D_{hi}^{2}$	(Legendre, 2013) [89]	(2)
Turnover (ReplS)	$\frac{2\times \min\left(b,c\right)}{2a+b+c}$	(Legendre, 2014) [12]	(3)
Nestedness (RichDiffS)	$\frac{\left|b,c\right|}{2a+b+c}$	(Legendre, 2014) [12]	(4)

**Table 6:** Environmental variables after correlation analysis and forward selection used for Mantel tests.

Type	S	Turnover	Nestedness
Temperature	Bio2	Bio2	Bio11
	Bio7	Bio7	
	Bio9	Bio9	
	Bio10	Bio10	
	Bio11	Bio11	
Precipitation	Bio15	Bio14	Bio16
	Bio16	Bio15	
	Bio18	Bio16	
Edaphic	Bdod	Bio18	Silt
	Phh2o	Bdod	Bdod
	Silt	Phh2o	Soc
	Sand	Silt	
	Cfvo	Cfvo	
Elevation	Elev	Elev	

**Table 7:** Selected species for niche overlap analysis.

	North (N)	Transition (T)	South (S)
1	*Plantago tubulosa*	*Deschampsia eminens*	*Ochetophila nana*
2	*Distichia muscoides*	*Cinnagrostis velutina*	*Ranunculus peduncularis*
3	*Hypochaeris taraxacoides*	*Eleocharis pseudoalbibracteata*	*Hordeum comosum*

**Table 8:** Environmental variables used in niche overlap analysis after correlation and forward selection analysis.

Climate		Edaphic
Bio2	Bio11	Bdod
Bio3	Bio12	Cfvo
Bio5	Bio15	Clay
Bio7	Bio19	Nitrogen
Bio9		Silt

## Data Availability

The data presented in this study are available on GBIF (https://www.gbif.org/, accessed on 1 November 2023), and CHELSA (https://chelsa-climate.org/downloads/, accessed on 1 June 2024).

## Supplementary Materials

The following supporting information can be downloaded at: https://www.mdpi.com/article/10.3390/plants13162202/s1, Figure S1: Principal Components Analysis. In green, the environmental niche of the first species mentioned in the title of each box; in red, the environmental niche of the second species mentioned in the title of each box; and in blue, the environmental niche shared by both species. The red arrow indicates the movement in the environmental space of the niche centroids for each species. The shaded areas correspond to the highest concentration of species occurrences, and the solid and dashed lines represent 100% and 75% of the total climatic envelope of the studied gradient, respectively. The species codes (e.g., N1) are according to Table 7. Figure S2: Biplot of environmental variables in Principal Components Analysis on which niche overlap was performed; Table S1: Complete list of high Andean bogs flora of southern South America used in this study.
