# Supplementary material for: Environmental Filters Structure Cushion Bogs’ Floristic Composition along the Southern South American Latitudinal Gradient
Source: Plants (Basel). 2024 Aug 9;13(16):2202. doi: 10.3390/plants13162202 (PMC11359879; doi:10.3390/plants13162202)
Supplement: Supplementary file 1 [file plants-13-02202-s001.zip › Table S1.pdf]

Table S1. *Complete list of high Andean bogs flora of southern South America used in this study for all sites. "Operational Zone" corresponds to those established in the methodology of this work. "Cluster" indicates the group to which each bog belongs according to hierarchical clustering analysis (Figure 3a). "Bioregion" indicates the corresponding group for each bog according to the membership grade model (Figure 3b). N = North; T = Transition; and S = South.*

**Bog**	**1**	**2**	**3**	**4**	**5**	**6**	**7**	**8**	**9**	**10**	**11**	**12**	**13**	**14**	**15**	**16**	**17**	**18**	**19**	**20**	**21**	**22**	**23**	**24**	**25**	**26**	**27**	**28**	**29**
**Operational zone**	**N**	**N**	**N**	**N**	**N**	**N**	**N**	**N**	**N**	**N**	**N**	**N**	**N**	**N**	**N**	**N**	**N**	**N**	**N**	**N**	**N**	**N**	**N**	**N**	**S**	**S**	**N**	**N**	**N**
**Cluster**	**4**	**1**	**1**	**1**	**1**	**1**	**1**	**1**	**1**	**1**	**1**	**1**	**1**	**1**	**1**	**1**	**1**	**1**	**1**	**1**	**1**	**1**	**1**	**1**	**7**	**7**	**2**	**2**	**1**
**Bioregion**	**T**	**T**	**T**	**T**	**T**	**T**	**T**	**N**	**N**	**N**	**N**	**N**	**N**	**N**	**T**	**N**	**T**	**N**	**N**	**N**	**N**	**T**	**N**	**N**	**S**	**S**	**T**	**T**	**T**
**Longitude**	**-68.66**	**-67.75**	**-67.60**	**-68.04**	**-68.86**	**-68.86**	**-68.85**	**-65.32**	**-65.25**	**-65.11**	**-66.54**	**-65.20**	**-66.95**	**-66.55**	**-66.93**	**-65.26**	**-67.00**	**-69.22**	**-69.22**	**-69.28**	**-69.29**	**-69.29**	**-69.19**	**-69.19**	**-71.39**	**-71.45**	**-68.22**	**-68.25**	**-68.26**
**Latitude**	**-24.57**	**-23.32**	**-23.07**	**-22.53**	**-20.22**	**-20.05**	**-20.42**	**-22.74**	**-22.24**	**-23.21**	**-22.33**	**-23.03**	**-22.67**	**-22.71**	**-22.59**	**-22.26**	**-23.86**	**-18.85**	**-18.53**	**-18.27**	**-18.22**	**-18.30**	**-18.12**	**-18.15**	**-37.38**	**-37.38**	**-27.62**	**-27.63**	**-27.64**
Acaena antarctica	.	.	.	.	.	.	.	.	.	.	.	.	.	.	.	.	.	.	.	.	.	.	.	.	.	.	.	.	.
Acaena macrocephala	.	.	.	.	.	.	.	.	.	.	.	.	.	.	.	.	.	.	.	.	.	.	.	.	.	1	.	.	.
Acaena magellanica	.	.	.	.	.	.	.	.	.	.	.	.	.	.	.	.	.	.	.	.	.	.	.	.	.	.	.	.	.
Acaena ovalifolia	.	.	.	.	.	.	.	.	.	.	.	.	.	.	.	.	.	.	.	.	.	.	.	.	.	1	.	.	.
Acaena pinnatifida	.	.	.	.	.	.	.	.	.	.	.	.	.	.	.	.	.	.	.	.	.	.	.	.	.	.	.	.	.
Adesmia retusa	.	.	.	.	.	.	.	.	.	.	.	.	.	.	.	.	.	.	.	.	.	.	.	.	.	.	.	.	.
Agrostis breviculmis	.	.	.	.	.	.	.	.	.	.	.	.	.	.	.	.	.	.	.	.	.	.	.	.	.	.	.	.	.
Agrostis imberbis	.	.	.	.	.	.	.	.	.	.	.	.	.	.	.	.	.	.	.	.	.	.	.	.	.	.	.	.	.
Agrostis meyenii	.	.	.	.	.	.	.	.	.	.	.	.	.	.	.	.	.	.	.	.	.	.	.	.	.	1	.	.	.
Agrostis perennans	.	.	.	.	.	.	.	.	.	.	.	.	.	.	.	.	.	.	.	.	.	.	.	.	.	.	.	.	.
Alchemilla pinnata	.	.	.	.	.	.	.	.	.	.	.	.	.	.	.	.	.	.	.	.	.	.	.	.	.	.	.	.	.
Alopecurus magellanicus	.	.	.	.	.	.	.	.	.	.	.	.	.	.	.	.	.	.	.	.	.	.	.	.	1	.	.	.	.
Amphiscirpus nevadensis	.	.	.	.	.	.	.	.	.	.	.	.	.	.	.	.	.	.	.	.	.	.	.	.	.	.	.	.	.
Anagallis alternifolia	.	.	.	.	.	.	.	.	.	.	.	.	.	.	.	.	.	.	.	.	.	.	.	.	1	1	.	.	.
Antennaria chilensis	.	.	.	.	.	.	.	.	.	.	.	.	.	.	.	.	.	.	.	.	.	.	.	.	.	.	.	.	.
Anthoxanthum redolens	.	.	.	.	.	.	.	.	.	.	.	.	.	.	.	.	.	.	.	.	.	.	.	.	.	.	.	.	.

**Bog**	**1**	**2**	**3**	**4**	**5**	**6**	**7**	**8**	**9**	**10**	**11**	**12**	**13**	**14**	**15**	**16**	**17**	**18**	**19**	**20**	**21**	**22**	**23**	**24**	**25**	**26**	**27**	**28**	**29**
Apium panul	.	.	.	.	.	.	.	.	.	.	.	.	.	.	.	.	.	.	.	.	.	.	.	.	1	.	.	.	.
Arenaria rivularis	.	.	.	.	.	.	.	.	.	.	.	.	.	.	.	.	.	.	.	.	.	.	.	.	.	.	.	.	.
Arenaria serpens	1	1	.	1	1	.	1	1	1	1	1	1	1	1	1	1	1	1	1	1	1	1	1	1	.	1	.	.	1
Arjona pusilla	.	.	.	.	.	.	.	.	.	.	.	.	.	.	.	.	.	.	.	.	.	.	.	.	.	.	.	.	.
Astragalus bustillosii	.	.	.	.	1	.	.	.	.	.	.	.	.	.	.	.	.	.	.	1	1	1	1	.	.	.	.	.	.
Astragalus micranthellus	.	.	.	1	.	.	.	.	.	.	.	.	.	.	.	.	.	.	.	.	.	.	.	.	.	.	.	.	.
Azolla filiculoides	.	.	.	.	.	.	.	.	.	.	.	.	.	.	.	.	.	.	.	.	.	1	1	.	.	.	.	.	.
Azorella boelckei	.	.	.	.	.	.	.	.	.	.	.	.	.	.	.	.	.	.	.	.	.	.	.	.	.	.	.	.	.
Azorella burkartii	.	.	.	.	.	.	.	.	.	.	.	.	.	.	.	.	.	.	.	.	.	.	.	.	.	.	.	.	.
Azorella cryptantha	.	.	.	.	.	.	.	.	.	.	.	.	.	.	.	.	.	.	.	.	.	.	.	.	.	.	.	.	.
Azorella lycopodioides	.	.	.	.	.	.	.	.	.	.	.	.	.	.	.	.	.	.	.	.	.	.	.	.	.	1	.	.	.
Azorella trifoliolata	.	.	.	.	.	.	.	.	.	.	.	.	.	.	.	.	.	.	.	.	.	.	.	.	.	.	.	.	.
Baccharis acaulis	.	.	.	.	.	.	1	.	.	.	.	.	.	.	.	.	1	.	.	.	.	.	.	.	.	.	.	.	.
Baccharis caespitosa	.	.	.	.	.	.	.	.	.	.	.	.	.	.	.	.	.	.	.	.	.	.	.	.	.	.	.	.	.
Baccharis magellanica	.	.	.	.	.	.	.	.	.	.	.	.	.	.	.	.	.	.	.	.	.	.	.	.	.	.	.	.	.
Belloa chilensis	.	.	.	.	.	.	.	.	.	.	.	.	.	.	.	.	.	.	.	.	.	.	.	.	.	.	.	.	.
Bromus catharticus	.	.	.	.	.	.	.	.	.	.	.	.	.	.	.	.	.	.	.	.	.	.	.	.	.	.	.	.	.
Calandrinia acaulis	.	.	.	.	.	.	.	1	.	.	1	.	.	.	.	.	.	.	.	.	.	.	.	.	.	.	.	.	.
Calandrinia compacta	.	.	.	.	.	.	.	1	.	1	.	1	1	1	1	1	1	.	.	.	.	.	.	.	.	.	.	.	.
Calceolaria biflora	.	.	.	.	.	.	.	.	.	.	.	.	.	.	.	.	.	.	.	.	.	.	.	.	.	.	.	.	.
Calceolaria cana	.	.	.	.	.	.	.	.	.	.	.	.	.	.	.	.	.	.	.	.	.	.	.	.	.	.	.	.	.
Calceolaria corymbosa	.	.	.	.	.	.	.	.	.	.	.	.	.	.	.	.	.	.	.	.	.	.	.	.	.	.	.	.	.
Calceolaria filicaulis	.	.	.	.	.	.	.	.	.	.	.	.	.	.	.	.	.	.	.	.	.	.	.	.	.	.	.	.	.
Callitriche lechleri	.	.	.	.	.	.	.	.	.	.	.	.	.	.	.	.	.	.	.	.	.	.	.	.	.	.	.	.	.
Caltha appendiculata	.	.	.	.	.	.	.	.	.	.	.	.	.	.	.	.	.	.	.	.	.	.	.	.	.	.	.	.	.
Caltha sagittata	.	.	.	.	.	.	.	.	.	.	.	.	.	.	.	.	.	.	.	.	.	.	.	.	1	1	.	.	.
Cardamine cordata	.	.	.	.	.	.	.	.	.	.	.	.	.	.	.	.	.	.	.	.	.	.	.	.	.	.	.	.	.
Cardamine glacialis	.	.	.	.	.	.	.	.	.	.	.	.	.	.	.	.	.	.	.	.	.	.	.	.	.	.	.	.	.
Cardamine tenuirostris	.	.	.	.	.	.	.	.	.	.	.	.	.	.	.	.	.	.	.	.	.	.	.	.	.	.	.	.	.
Cardamine volckmannii	.	.	.	.	.	.	.	1	1	.	.	.	.	.	.	1	.	.	.	.	.	.	.	.	.	.	.	.	.

**Bog**	**1**	**2**	**3**	**4**	**5**	**6**	**7**	**8**	**9**	**10**	**11**	**12**	**13**	**14**	**15**	**16**	**17**	**18**	**19**	**20**	**21**	**22**	**23**	**24**	**25**	**26**	**27**	**28**	**29**
Carex acaulis	.	.	.	.	.	.	.	.	.	.	.	.	.	.	.	.	.	.	.	.	.	.	.	.	.	.	.	.	.
Carex atropicta	.	.	.	.	.	.	.	.	.	.	.	.	.	.	.	.	.	.	.	.	.	.	.	.	.	1	.	.	.
Carex banksii	.	.	.	.	.	.	.	.	.	.	.	.	.	.	.	.	.	.	.	.	.	.	.	.	1	.	.	.	.
Carex caduca	.	.	.	.	.	.	.	.	.	.	.	.	.	.	.	.	.	.	.	.	.	.	.	.	.	.	.	.	.
Carex decidua	.	.	.	.	.	.	.	.	.	.	.	.	.	.	.	.	.	.	.	.	.	.	.	.	1	.	.	.	.
Carex fuscula	.	.	.	.	.	.	.	.	.	.	.	.	.	.	.	.	.	.	.	.	.	.	.	.	1	1	.	.	.
Carex gayana	.	.	.	.	.	.	.	.	1	.	.	1	1	1	.	1	1	.	.	.	.	.	.	.	1	1	1	.	.
Carex hypoleucos	.	.	.	.	.	.	.	.	.	.	.	.	.	.	.	.	.	.	.	.	.	.	.	.	1	.	.	.	.
Carex macloviana	.	.	.	.	.	.	.	.	.	.	.	.	.	.	.	.	.	.	.	.	.	.	.	.	1	1	.	.	.
Carex magellanica	.	.	.	.	.	.	.	.	.	.	.	.	.	.	.	.	.	.	.	.	.	.	.	.	.	1	.	.	.
Carex malmei	.	.	.	.	.	.	.	.	.	.	.	.	.	.	.	.	.	.	.	.	.	.	.	.	.	.	.	.	.
Carex maritima	1	1	1	1	1	.	.	1	1	1	1	1	1	1	1	.	1	.	.	.	.	.	.	.	.	.	.	.	.
Carex microglochin	.	.	.	.	.	.	.	1	.	.	.	.	.	.	.	1	.	.	.	.	.	.	.	.	.	.	.	.	1
Carex pleioneura	.	.	.	.	.	.	.	.	.	.	.	.	.	.	.	.	.	.	.	.	.	.	.	.	.	.	.	.	.
Carex ruthsatzae	.	.	.	.	.	.	.	.	.	.	.	.	.	.	.	.	.	.	.	.	.	.	.	.	.	.	.	.	.
Carex vallis-pulchrae	.	.	.	.	.	.	.	.	.	.	.	.	.	.	.	.	.	.	.	.	.	.	.	.	.	.	.	1	1
Carpha schoenoides	.	.	.	.	.	.	.	.	.	.	.	.	.	.	.	.	.	.	.	.	.	.	.	.	.	.	.	.	.
Castilleja pumila	.	.	.	.	.	.	.	1	1	.	.	.	.	.	.	1	.	.	.	.	.	1	.	.	.	.	.	.	.
Catabrosa werdermannii	.	.	1	.	.	.	.	.	1	.	1	1	1	1	.	1	1	.	.	.	.	.	.	.	.	.	.	.	.
Cerastium humifusum	.	.	.	.	.	.	.	.	.	.	.	.	.	.	.	.	.	.	.	.	.	.	.	.	.	.	.	.	.
Cerastium montioides	.	.	.	.	.	.	.	.	.	.	.	.	.	.	.	.	.	.	.	.	.	.	.	.	.	.	.	.	.
Chiliotrichum diffusum	.	.	.	.	.	.	.	.	.	.	.	.	.	.	.	.	.	.	.	.	.	.	.	.	.	1	.	.	.
Chusquea culeou	.	.	.	.	.	.	.	.	.	.	.	.	.	.	.	.	.	.	.	.	.	.	.	.	.	.	.	.	.
Colobanthus quitensis	.	1	.	1	.	.	.	1	1	1	1	1	1	1	1	1	1	1	1	.	1	.	.	.	.	1	1	1	.
Cortaderia egmontiana	.	.	.	.	.	.	.	.	.	.	.	.	.	.	.	.	.	.	.	.	.	.	.	.	.	1	.	.	.
Cotula mexicana	.	.	.	.	.	.	.	1	1	1	1	.	.	1	.	1	.	.	.	.	.	.	.	.	.	.	.	.	.
Crassula peduncularis	.	.	.	.	.	.	.	.	.	.	.	.	.	.	.	.	.	.	.	.	.	.	.	.	.	.	.	.	.
Cuatrecasasiella argentina	.	.	.	.	.	.	.	1	1	.	1	.	.	.	.	1	.	.	1	.	.	1	.	.	.	.	.	.	.
Deschampsia antárctica	.	.	.	.	.	.	.	.	.	.	.	.	.	.	.	.	.	.	.	.	.	.	.	.	.	.	.	.	.

**Bog**	**1**	**2**	**3**	**4**	**5**	**6**	**7**	**8**	**9**	**10**	**11**	**12**	**13**	**14**	**15**	**16**	**17**	**18**	**19**	**20**	**21**	**22**	**23**	**24**	**25**	**26**	**27**	**28**	**29**
Deschampsia caespitosa	.	.	.	.	.	.	.	.	.	.	.	.	.	.	.	.	.	.	.	.	.	.	.	.	.	.	.	.	.
Deschampsia patula	.	.	.	.	.	.	.	.	.	.	.	.	.	.	.	.	.	.	.	.	.	.	.	.	.	.	.	.	.
Cinnagrostis brevifolia	.	.	.	.	.	.	.	.	.	.	.	.	.	.	.	.	.	.	.	.	.	.	.	.	.	.	.	.	.
Deschampsia chrysantha	.	.	1	1	.	.	.	1	.	.	1	1	1	.	1	.	.	.	1	1	1	1	1	1	.	.	.	.	.
Cinnagrostis chrysophylla	.	.	.	.	.	.	.	.	.	.	.	.	.	.	.	.	.	.	.	.	.	.	.	.	.	.	.	.	.
Deschampsia chrysostachya	.	.	.	.	.	.	.	.	.	.	.	1	.	1	1	.	1	.	.	.	.	.	.	.	.	.	.	.	.
Deschampsia eminens	.	.	.	.	.	.	.	1	1	1	1	1	1	1	1	1	1	.	.	.	.	.	.	.	.	.	1	1	1
Deschampsia hackelii	.	.	.	.	.	.	.	.	.	.	.	.	.	.	.	.	.	.	.	.	.	.	.	.	.	.	.	.	.
Cinnagrostis minima	.	.	.	.	.	.	.	1	1	1	1	1	1	1	1	1	1	.	.	.	.	.	.	.	.	.	.	.	.
Deschampsia ovata	.	.	.	.	.	.	.	.	.	.	.	.	.	.	.	.	.	.	.	.	.	.	.	.	.	.	.	.	.
Cinnagrostis rigescens	.	.	.	.	.	.	.	1	1	1	1	.	.	.	.	1	.	.	.	.	.	.	.	.	.	.	.	.	.
Cinnagrostis spicigera	.	.	.	.	.	.	.	.	.	.	.	.	.	.	.	.	.	.	.	.	.	.	.	.	.	.	.	.	.
Cinnagrostis velutina	1	1	1	1	1	1	.	1	1	1	1	1	1	1	1	1	1	1	1	1	1	1	1	1	.	.	.	.	.
Cinnagrostis vicunarum	.	.	.	.	.	.	.	.	.	.	.	.	.	.	.	.	.	.	.	.	.	.	.	.	.	.	.	.	.
Distichia filamentosa	.	.	.	.	.	.	.	.	.	.	.	.	.	.	.	.	.	.	.	.	.	.	.	1	.	.	.	.	.
Distichia muscoides	.	.	.	.	.	.	.	1	1	1	1	1	1	1	.	.	.	1	1	1	1	1	1	1	.	.	.	.	.
Distichlis humilis	.	.	.	.	1	.	1	.	.	.	.	.	.	.	1	.	1	.	.	.	.	.	.	.	.	.	1	.	.
Distichlis scoparia	.	.	.	.	.	.	.	.	.	.	.	.	.	.	.	.	.	.	.	.	.	.	.	.	.	.	.	.	.
Distichlis spicata	.	.	.	.	.	.	.	.	.	.	.	.	.	.	.	.	.	.	.	.	.	.	.	.	.	.	.	.	.
Draba pusilla	.	.	.	.	.	.	.	.	.	.	.	.	.	.	.	.	.	.	.	.	.	.	.	.	.	.	.	.	.
Eleocharis melanomphala	.	.	.	.	.	.	.	.	.	.	.	.	.	.	.	.	.	.	.	.	.	.	.	.	.	.	1	.	1
Eleocharis pseudoalbibracteata	.	.	.	.	.	.	.	.	.	.	.	.	.	.	.	.	.	.	.	.	.	.	.	.	.	.	.	1	.
Elodea potamogeton	.	.	.	.	.	.	.	.	.	.	.	.	.	.	.	.	.	.	.	.	.	.	.	.	.	.	.	.	.
Empetrum rubrum	.	.	.	.	.	.	.	.	.	.	.	.	.	.	.	.	.	.	.	.	.	.	.	.	.	1	.	.	.
Epilobium australe	.	.	.	.	.	.	.	.	.	.	.	.	.	.	.	.	.	.	.	.	.	.	.	.	.	1	.	.	.
Epilobium barbeyanum	.	.	.	.	.	.	.	.	.	.	.	.	.	.	.	.	.	.	.	.	.	.	.	.	1	.	.	.	.
Epilobium ciliatum	.	.	.	.	.	.	.	.	.	.	.	.	.	.	.	.	.	.	.	.	.	.	.	.	1	1	.	.	.

**Bog**	**1**	**2**	**3**	**4**	**5**	**6**	**7**	**8**	**9**	**10**	**11**	**12**	**13**	**14**	**15**	**16**	**17**	**18**	**19**	**20**	**21**	**22**	**23**	**24**	**25**	**26**	**27**	**28**	**29**
Epilobium denticulatum																													
Epilobium fragile	.	.	.	.	.	.	.	.	.	.	.	.	.	.	.	.	.	.	.	.	.	.	.	.	.	.	.	.	.
Epilobium glaucum	.	.	.	.	.	.	.	.	.	.	.	.	.	.	.	.	.	.	.	.	.	.	.	.	.	.	.	.	.
Epilobium nivale	.	.	.	.	.	.	.	.	.	.	.	.	.	.	.	.	.	.	.	.	.	.	.	.	1	.	.	.	.
Erigeron andicola	.	.	.	.	.	.	.	.	.	.	.	.	.	.	.	.	.	.	.	.	.	.	.	.	.	.	.	.	.
Erigeron leptopetalus	.	.	.	.	.	.	.	.	.	.	.	.	.	.	.	.	.	.	.	.	.	.	.	.	.	.	.	.	.
Erigeron myosotis	.	.	.	.	.	.	.	.	.	.	.	.	.	.	.	.	.	.	.	.	.	.	.	.	.	.	.	.	.
Erigeron patagonicus	.	.	.	.	.	.	.	.	.	.	.	.	.	.	.	.	.	.	.	.	.	.	.	.	.	1	.	.	.
Erythranthe cuprea	.	.	.	.	.	.	.	.	.	.	.	.	.	.	.	.	.	.	.	.	.	.	.	.	.	1	.	.	.
Erythranthe depressa	.	.	.	.	.	.	.	.	.	.	.	.	.	.	.	.	.	.	.	.	.	.	.	.	.	.	.	.	.
Erythranthe glabrata	.	1	.	.	1	.	.	1	1	1	1	1	1	1	1	1	1	.	.	.	.	1	.	.	.	.	1	.	.
Erythranthe lutea	.	.	.	.	.	.	.	.	.	.	.	.	.	.	.	.	.	.	.	.	.	.	.	.	.	.	.	.	.
Escallonia virgata	.	.	.	.	.	.	.	.	.	.	.	.	.	.	.	.	.	.	.	.	.	.	.	.	1	1	.	.	.
Euphrasia antarctica	.	.	.	.	.	.	.	.	.	.	.	.	.	.	.	.	.	.	.	.	.	.	.	.	1	1	.	.	.
Euphrasia chrysantha	.	.	.	.	.	.	.	1	1	1	.	.	.	.	.	1	.	.	.	.	.	.	.	.	.	.	.	.	.
Euphrasia subexserta	.	.	.	.	.	.	.	.	.	.	.	.	.	.	.	.	.	.	.	.	.	.	.	.	.	1	.	.	.
Festuca hypsophila	.	.	.	.	.	.	.	.	.	.	.	.	.	.	.	.	.	.	.	.	.	.	.	.	1	1	.	.	.
Festuca kurtziana	.	.	.	.	.	.	.	.	.	.	.	.	.	.	.	.	.	.	.	.	.	.	.	.	.	.	.	.	.
Festuca lilloi	.	.	.	.	.	.	.	.	.	.	.	.	.	.	.	.	.	.	.	.	.	.	.	.	.	.	.	.	.
Festuca magellanica	.	.	.	.	.	.	.	1	.	.	1	1	.	1	1	1	.	.	.	.	.	.	.	.	.	.	.	.	.
Festuca nardifolia	.	.	.	.	.	.	.	.	.	.	.	.	.	.	.	.	.	.	.	.	.	.	.	.	.	1	.	.	.
Festuca rigescens	1	.	.	.	.	.	.	1	1	1	1	1	1	1	1	1	1	.	.	.	.	.	.	.	.	.	.	.	.
Festuca werdermannii	1	1	1	1	1	1	1	.	.	.	.	.	.	.	.	.	.	1	1	1	1	1	1	1	.	.	.	.	.
Frankenia triandra	.	.	.	.	.	.	.	.	.	.	.	.	.	.	.	.	.	.	.	.	.	.	.	.	.	.	.	.	.
Gamocarpha graminea	.	.	.	.	.	.	1	.	.	.	.	.	.	.	.	.	.	1	.	.	.	.	.	.	.	.	.	.	.
Gamocarpha ventosa	.	.	.	.	.	.	.	.	.	.	.	.	.	.	.	.	.	.	.	.	.	.	.	.	.	1	.	.	.
Gamochaeta chamissonis	.	.	.	.	.	.	.	.	.	.	.	.	.	.	.	.	.	.	.	.	.	.	.	.	.	.	.	.	.
Gamochaeta longipedicellata	.	.	.	.	.	.	.	.	.	.	.	.	.	.	.	.	.	.	.	.	.	.	.	.	.	.	.	.	.
	.	.	.	.	.	.	.	1	1	.	1	.	.	.	.	1	.	.	.	.	.	.	.	.	.	.	.	.	.
Gamochaeta neuquensis	.	.	.	.	.	.	.	.	.	.	.	.	.	.	.	.	.	.	.	.	.	.	.	.	.	.	.	.	.

**Bog**	**1**	**2**	**3**	**4**	**5**	**6**	**7**	**8**	**9**	**10**	**11**	**12**	**13**	**14**	**15**	**16**	**17**	**18**	**19**	**20**	**21**	**22**	**23**	**24**	**25**	**26**	**27**	**28**	**29**
Gaultheria antarctica	.	.	.	.	.	.	.	.	.	.	.	.	.	.	.	.	.	.	.	.	.	.	.	.	.	.	.	.	.
Gaultheria caespitosa	.	.	.	.	.	.	.	.	.	.	.	.	.	.	.	.	.	.	.	.	.	.	.	.	.	1	.	.	.
Gaultheria pumila	.	.	.	.	.	.	.	.	.	.	.	.	.	.	.	.	.	.	.	.	.	.	.	.	.	1	.	.	.
Gavilea chica	.	.	.	.	.	.	.	.	.	.	.	.	.	.	.	.	.	.	.	.	.	.	.	.	.	.	.	.	.
Gentiana prostrata	.	1	1	1	1	.	.	1	1	1	1	1	1	1	1	1	1	1	1	1	1	1	1	1	.	1	1	1	1
Gentianella fiebrigii	.	.	.	.	.	.	.	.	.	.	.	.	.	.	.	.	.	.	.	.	.	.	.	.	.	.	.	.	.
Gentianella magellanica	.	.	.	.	.	.	.	.	.	.	.	.	.	.	.	.	.	.	.	.	.	.	.	.	.	.	.	.	.
Gentianella multicaulis	.	.	.	.	.	.	.	.	.	.	.	.	.	.	.	.	.	.	.	.	.	.	.	.	.	.	.	.	.
Gentianella ottonis	.	.	.	.	.	.	.	.	.	.	.	.	.	.	.	.	.	.	.	.	.	.	.	.	.	.	.	.	.
Gentianella primuloides	.	.	.	.	.	.	.	.	.	.	.	.	.	.	.	.	.	.	.	.	.	.	.	.	.	.	.	.	.
Gentianella pseudocrassula	.	.	.	.	.	.	.	.	.	.	.	.	.	.	.	.	.	.	.	.	.	.	.	.	.	.	.	.	.
Geranium sessiliflorum	.	.	.	.	.	.	.	.	.	.	.	.	.	.	.	.	.	.	.	.	.	.	.	.	.	1	.	.	.
Gunnera magellanica	.	.	.	.	.	.	.	.	.	.	.	.	.	.	.	.	.	.	.	.	.	.	.	.	1	1	.	.	.
Halenia caespitosa	.	.	.	.	.	.	.	.	.	.	.	.	.	.	.	.	.	.	.	.	.	.	.	.	.	.	.	.	.
Halerpestes cymbalaria	1	.	1	1	1	1	.	.	1	.	1	.	.	1	1	1	1	.	.	.	.	.	.	.	.	.	1	.	.
Halerpestes exilis	.	.	.	.	.	.	.	1	.	1	1	1	1	1	1	.	1	.	.	.	.	.	.	.	.	.	.	.	.
Hieracium antarcticum	.	.	.	.	.	.	.	.	.	.	.	.	.	.	.	.	.	.	.	.	.	.	.	.	.	.	.	.	.
Hordeum comosum	.	.	.	.	.	.	.	.	.	.	.	.	.	.	.	.	.	.	.	.	.	.	.	.	.	.	.	.	.
Hordeum muticum	.	.	.	.	.	.	.	.	.	.	.	.	.	.	.	.	.	.	.	.	.	.	.	.	.	.	1	.	.
Hypochaeris acaulis	.	.	.	.	.	.	.	.	.	.	.	.	.	.	.	.	.	.	.	.	.	.	.	.	.	1	.	.	.
Hypochaeris chondrilloides	.	.	.	.	.	.	.	.	.	.	.	.	.	.	.	.	.	.	.	.	.	.	.	.	.	.	.	.	.
Hypochaeris meyeniana	.	.	.	.	.	.	.	.	.	.	.	1	.	1	1	.	1	.	.	.	.	.	.	.	.	.	.	.	.
Hypochaeris palustris	.	.	.	.	.	.	.	.	.	.	.	.	.	.	.	.	.	.	.	.	.	.	.	.	.	.	.	.	.
Hypochaeris taraxacoides	.	.	.	.	.	.	.	1	1	1	1	1	1	1	1	1	.	1	1	1	1	1	1	1	.	.	.	.	.
Hypochaeris tenerifolia	.	.	.	.	.	.	.	.	.	.	.	.	.	.	.	.	.	.	.	.	.	.	.	.	.	1	.	.	.
Isolepis nigricans	.	.	.	.	.	.	.	.	.	.	.	.	.	.	.	.	.	.	.	.	.	.	.	.	.	.	.	.	.
Isolepsis inundata	.	.	.	.	.	.	.	.	.	.	.	.	.	.	.	.	.	.	.	.	.	.	.	.	.	1	.	.	.
Juncus balticus	.	1	.	.	.	.	.	.	1	1	.	.	.	1	1	1	1	.	.	.	.	.	.	.	1	1	1	1	.
Juncus stipulatus	1	1	.	1	1	.	1	1	1	1	1	1	1	1	1	1	1	1	1	1	1	1	1	1	1	1	1	.	.

**Bog**	**1**	**2**	**3**	**4**	**5**	**6**	**7**	**8**	**9**	**10**	**11**	**12**	**13**	**14**	**15**	**16**	**17**	**18**	**19**	**20**	**21**	**22**	**23**	**24**	**25**	**26**	**27**	**28**	**29**
Koeleria kurtzii	.	.	.	.	.	.	.	.	.	.	.	.	.	.	.	.	.	.	.	.	.	.	.	.	.	.	.	.	.
Lachemilla diplophylla	.	.	.	.	.	.	.	1	1	.	.	.	.	.	.	1	.	1	1	1	.	1	1	.	.	.	.	.	.
Lachemilla pinnata	.	.	.	.	.	.	.	1	1	1	1	1	1	1	.	1	.	.	.	.	.	.	.	.	.	.	.	.	.
Lagenophora nudicaulis	.	.	.	.	.	.	.	.	.	.	.	.	.	.	.	.	.	.	.	.	.	.	.	.	.	.	.	.	.
Lemna minuta	.	.	.	.	.	.	.	1	.	.	1	.	1	.	.	1	.	.	.	.	.	.	.	.	.	.	.	.	.
Leptinella scariosa	.	.	.	.	.	.	.	.	.	.	.	.	.	.	.	.	.	.	.	.	.	.	.	.	.	.	.	.	.
Leucheria candidissima	.	.	.	.	.	.	.	.	.	.	.	.	.	.	.	.	.	.	.	.	.	.	.	.	.	.	.	.	.
Leucheria nutans	.	.	.	.	.	.	.	.	.	.	.	.	.	.	.	.	.	.	.	.	.	.	.	.	.	.	.	.	.
Lilaea scilloides	.	.	.	.	.	.	.	1	.	.	1	.	.	.	.	1	1	.	.	.	.	.	.	.	.	.	.	.	.
Lilaeopsis macloviana	.	1	1	1	1	.	.	1	1	1	1	1	1	1	1	1	1	1	1	1	1	1	1	.	1	.	1	.	.
Limosella australis	.	.	.	.	.	.	.	1	1	.	.	.	.	.	.	.	.	.	.	.	.	.	.	.	.	.	.	.	.
Lobelia oligophylla	1	1	.	1	1	.	1	1	1	1	1	1	1	1	1	1	1	1	1	1	1	1	1	1	1	1	1	1	.
Luzula brachyphylla	.	.	.	.	.	.	.	.	.	.	.	.	.	.	.	.	.	.	.	.	.	.	.	.	.	.	.	.	.
Luzula chilensis	.	.	.	.	.	.	.	.	.	.	.	.	.	.	.	.	.	.	.	.	.	.	.	.	.	1	.	.	.
Luzula racemosa	.	.	.	.	.	.	.	1	1	.	1	.	.	.	.	1	.	.	.	.	.	.	.	.	.	.	.	.	.
Luzula vulcanica	.	.	.	.	.	.	.	.	.	.	.	.	.	.	.	.	.	.	.	.	1	.	.	1	.	.	.	.	.
Lysipomia pumila	.	.	.	.	.	.	.	.	.	.	.	.	.	.	.	.	.	.	.	.	.	.	.	.	.	.	.	.	.
Marsippospermum philippii	.	.	.	.	.	.	.	.	.	.	.	.	.	.	.	.	.	.	.	.	.	.	.	.	.	.	.	.	.
Marsippospermum reichei	.	.	.	.	.	.	.	.	.	.	.	.	.	.	.	.	.	.	.	.	.	.	.	.	.	.	.	.	.
Montia fontana	.	.	.	.	.	.	.	1	.	.	1	.	.	1	.	1	.	.	.	.	.	.	.	.	.	.	.	.	.
Muhlenbergia asperifolia	.	.	.	.	.	.	.	.	.	.	.	.	.	.	.	.	.	.	.	.	.	.	.	.	.	.	.	.	.
Myriophyllum quitense	.	.	1	.	1	.	.	1	.	.	1	1	1	1	1	.	1	.	1	.	1	.	1	.	1	.	.	.	.
Myrosmodes nervosa	.	.	.	.	.	.	.	.	.	.	.	.	.	.	.	.	.	.	1	.	.	1	1	.	.	.	.	.	.
Myrosmodes paludosa	.	.	.	.	.	.	.	1	1	.	.	.	.	.	.	1	.	.	.	.	.	.	.	.	.	.	.	.	.
Myrteola nummularia	.	.	.	.	.	.	.	.	.	.	.	.	.	.	.	.	.	.	.	.	.	.	.	.	.	.	.	.	.
Nanodea muscosa	.	.	.	.	.	.	.	.	.	.	.	.	.	.	.	.	.	.	.	.	.	.	.	.	.	.	.	.	.
Neobartsia crenoloba	.	.	.	.	.	.	.	1	1	.	.	.	.	.	.	1	.	.	.	.	.	.	.	.	.	.	.	.	.
Neobartsia pedicularoides	.	.	.	.	.	.	.	.	.	.	.	.	.	.	.	.	.	.	.	.	.	.	.	.	.	.	.	.	.

Bog	1	2	3	4	5	6	7	8	9	10	11	12	13	14	15	16	17	18	19	20	21	22	23	24	25	26	27	28	29
Neobartsia peruviana	.	.	.	.	.	.	.	.	.	.	.	.	.	.	.	.	.	.	.	.	.	.	.	.	.	.	.	.	.
Nertera granadensis	.	.	.	.	.	.	.	.	.	.	.	.	.	.	.	.	.	.	.	.	.	.	.	.	.	.	.	.	.
Nicoraepoa andina	.	.	.	.	.	.	.	.	.	.	.	.	.	.	.	.	.	.	.	.	.	.	.	.	.	1	.	.	.
Nicoraepoa pugionifolia	.	.	.	.	.	.	.	.	.	.	.	.	.	.	.	.	.	.	.	.	.	.	.	.	.	.	.	.	.
Nicoraepoa subenervis	.	.	.	.	.	.	.	.	.	.	.	.	.	.	.	.	.	.	.	.	.	.	.	.	.	.	.	.	.
Nitrophila australis	.	.	.	.	.	.	.	.	.	.	.	.	.	.	.	.	.	.	.	.	.	.	.	.	.	.	.	.	.
Nothofagus antarctica	.	.	.	.	.	.	.	.	.	.	.	.	.	.	.	.	.	.	.	.	.	.	.	.	1	1	.	.	.
Nototriche rugosa	.	.	.	1	.	.	.	.	.	.	.	.	.	.	.	.	.	.	.	.	.	.	.	.	.	.	.	.	.
Ochetophila nana	.	.	.	.	.	.	.	.	.	.	.	.	.	.	.	.	.	.	.	.	.	.	.	.	.	1	.	.	.
Olsynium junceum	.	.	.	.	.	.	.	.	.	.	.	.	.	.	.	.	.	.	.	.	.	.	.	.	.	.	.	.	.
Oreobolus obtusangulus	.	.	.	.	.	.	.	.	.	.	.	.	.	.	.	.	.	.	.	.	.	.	.	.	.	.	.	.	.
Oritrophium limnophilum	.	.	.	.	.	.	.	.	.	.	.	.	.	.	.	.	.	.	.	.	.	.	.	.	.	.	.	.	.
Osmorhiza glabrata	.	.	.	.	.	.	.	.	.	.	.	.	.	.	.	.	.	.	.	.	.	.	.	.	.	.	.	.	.
Ourisia alpina	.	.	.	.	.	.	.	.	.	.	.	.	.	.	.	.	.	.	.	.	.	.	.	.	.	.	.	.	.
Ourisia muscosa	.	.	.	.	.	.	.	1	.	.	1	.	.	.	.	.	.	.	.	.	.	.	.	.	.	.	.	.	.
Ourisia ruelloides	.	.	.	.	.	.	.	.	.	.	.	.	.	.	.	.	.	.	.	.	.	.	.	.	.	.	.	.	.
Oxychloe andina	1	.	1	1	1	1	.	1	.	1	1	1	1	1	1	.	1	1	1	1	1	1	1	1	.	.	1	1	1
Oxychloe bisexualis	.	.	.	.	.	.	.	.	.	.	.	.	.	.	.	.	.	.	.	.	.	.	.	.	.	.	.	.	.
Oxychloe castellanosii	.	.	.	.	.	.	.	.	.	.	.	.	.	.	.	.	.	.	.	.	.	.	.	.	.	.	.	.	.
Oxychloe haumaniana	.	.	.	.	.	.	.	.	.	.	.	.	.	.	.	.	.	.	.	.	.	.	.	.	.	.	.	.	.
Oxychloe mendocina	.	.	.	.	.	.	.	.	.	.	.	.	.	.	.	.	.	.	.	.	.	.	.	.	.	.	.	.	.
Patosia clandestina	.	.	.	.	.	.	.	.	.	.	.	.	.	.	.	.	.	.	.	.	.	.	.	.	.	1	.	.	.
Perezia capito	.	.	.	.	.	.	.	.	.	.	.	.	.	.	.	.	.	.	.	.	.	.	.	.	.	.	.	.	.
Perezia delicata	.	.	.	.	.	.	.	.	.	.	.	.	.	.	.	.	.	.	.	.	.	.	.	.	.	.	.	.	.
Perezia fonkii	.	.	.	.	.	.	.	.	.	.	.	.	.	.	.	.	.	.	.	.	.	.	.	.	.	.	.	.	.
Perezia pedicularidifolia	.	.	.	.	.	.	.	.	.	.	.	.	.	.	.	.	.	.	.	.	.	.	.	.	.	.	.	.	.
Perezia pinnatifida	.	.	.	.	.	.	.	1	1	1	1	1	1	1	1	1	.	.	.	.	.	.	.	.	.	.	.	.	.
Petroravenia friesii	.	.	.	.	.	.	.	1	1	1	1	1	1	1	1	.	.	.	.	.	.	.	.	.	.	.	.	.	.

Bog	1	2	3	4	5	6	7	8	9	10	11	12	13	14	15	16	17	18	19	20	21	22	23	24	25	26	27	28	29
Petroravenia werdermannii	.	.	.	.	.	.	.	.	.	.	.	.	.	.	.	.	.	.	.	.	.	.	.	.	.	.	.	.	.
Phleum alpinum	.	.	.	.	.	.	.	.	.	.	.	.	.	.	.	.	.	.	.	.	.	.	.	.	1	1	.	.	.
Phylloscirpus acaulis	.	1	.	.	.	.	.	1	1	.	.	1	.	.	1	1	.	.	.	.	.	.	.	.	1	.	1	1	.
Phylloscirpus boliviensis	.	.	.	.	.	.	.	1	.	.	.	.	.	.	.	.	.	.	.	.	.	.	.	.	.	.	.	.	.
Phylloscirpus deserticola	.	1	.	1	1	1	1	1	1	.	1	1	1	1	1	1	1	1	1	1	1	.	1	1	.	.	.	.	.
Pinguicula antarctica	.	.	.	.	.	.	.	.	.	.	.	.	.	.	.	.	.	.	.	.	.	.	.	.	.	.	.	.	.
Plantago barbata	.	.	.	.	.	.	.	.	.	.	.	.	.	.	.	.	.	.	.	.	.	.	.	.	.	1	.	.	.
Plantago rigida	.	.	.	.	.	.	.	.	.	.	.	.	.	.	.	.	.	.	.	.	.	.	.	.	.	.	.	.	.
Plantago tubulosa	.	.	.	.	.	.	.	1	1	1	1	1	1	1	1	1	1	.	.	.	.	.	.	.	.	.	.	.	.
Plantago uniglumis	.	.	.	.	.	.	.	.	.	.	.	.	.	.	.	.	.	.	.	.	.	.	.	.	.	.	.	.	.
Poa alopecurus	.	.	.	.	.	.	.	.	.	.	.	.	.	.	.	.	.	.	.	.	.	.	.	.	.	.	.	.	.
Poa hachadoensis	.	.	.	.	.	.	.	.	.	.	.	.	.	.	.	.	.	.	.	.	.	.	.	.	1	.	.	.	.
Poa perligulata	.	.	.	.	.	.	.	1	1	1	1	1	1	1	.	1	.	.	.	1	1	1	1	1	.	.	.	.	.
Polypogon interruptus	.	.	.	.	1	.	.	.	.	.	.	.	.	.	.	.	.	.	.	.	.	.	.	.	1	.	.	.	.
Primula magellanica	.	.	.	.	.	.	.	.	.	.	.	.	.	.	.	.	.	.	.	.	.	.	.	.	.	.	.	.	.
Puccinellia frigida	1	1	1	1	1	.	1	.	.	.	.	.	.	.	.	.	.	1	1	.	.	.	.	.	.	.	.	.	.
Quinchamalium chilense	.	.	.	.	.	.	.	.	.	.	.	.	.	.	.	.	.	.	.	.	.	.	.	.	.	.	.	.	.
Ranunculus breviscapus	.	.	.	.	.	.	.	.	.	.	.	.	.	.	.	.	.	.	.	.	.	.	.	.	.	.	.	.	.
Ranunculus fuegianus	.	.	.	.	.	.	.	.	.	.	.	.	.	.	.	.	.	.	.	.	.	.	.	.	.	.	.	.	.
Ranunculus mandonianus	.	.	.	.	.	.	.	.	.	.	.	.	.	.	.	.	.	.	.	.	.	.	.	.	.	.	.	.	.
Ranunculus peduncularis	.	.	.	.	.	.	.	.	.	.	.	.	.	.	.	.	.	.	.	.	.	.	.	.	1	1	.	.	.
Ranunculus trichophyllus	.	.	.	.	.	.	.	.	.	.	.	.	.	.	.	.	.	.	.	1	1	.	.	.	.	.	.	.	.
Halerpestes uniflora	.	.	.	.	.	.	.	.	.	.	.	.	.	.	.	.	.	.	.	.	.	.	.	.	.	.	.	.	.
Rubus geoides	.	.	.	.	.	.	.	.	.	.	.	.	.	.	.	.	.	.	.	.	.	.	.	.	1	.	.	.	.
Rumex magellanicus	.	.	.	.	.	.	.	.	.	.	.	.	.	.	.	.	.	.	.	.	.	.	.	.	1	.	.	.	.
Rytidosperma lechleri	.	.	.	.	.	.	.	.	.	.	.	.	.	.	.	.	.	.	.	.	.	.	.	.	.	1	.	.	.
Sarcocornia pulvinata	.	.	.	.	.	.	1	.	.	.	.	.	.	.	.	.	.	.	.	.	.	.	.	.	.	.	.	.	.

Bog	1	2	3	4	5	6	7	8	9	10	11	12	13	14	15	16	17	18	19	20	21	22	23	24	25	26	27	28	29
Schoenoplectus pungens	.	.	.	.	.	.	1	.	.	.	.	.	.	.	.	.	.	.	.	.	.	.	.	.	.	.	.	.	.
Schoenus andinus	.	.	.	.	.	.	.	.	.	.	.	.	.	.	.	.	.	.	.	.	.	.	.	.	.	1	.	.	.
Senecio breviscapus	.	.	.	.	.	.	.	.	1	.	1	1	1	.	.	1	.	.	.	.	.	.	.	.	.	.	.	.	.
Senecio diemii	.	.	.	.	.	.	.	.	.	.	.	.	.	.	.	.	.	.	.	.	.	.	.	.	.	.	.	.	.
Senecio fistulosus	.	.	.	.	.	.	.	.	.	.	.	.	.	.	.	.	.	.	.	.	.	.	.	.	1	1	.	.	.
Senecio parodii	.	.	.	.	.	.	.	.	.	.	.	.	.	.	.	.	.	.	.	.	.	.	.	.	.	1	.	.	.
Senecio peteroanus	.	.	.	.	.	.	.	.	.	.	.	.	.	.	.	.	.	.	.	.	.	.	.	.	.	.	.	.	.
Senecio serratifolius	.	.	.	.	.	.	.	.	.	.	1	1	1	.	.	.	.	1	.	.	.	.	.	.	.	.	.	.	.
Senecio trifurcatus	.	.	.	.	.	.	.	.	.	.	.	.	.	.	.	.	.	.	.	.	.	.	.	.	.	.	.	.	.
Sisyrinchium chilense	.	.	.	.	.	.	.	.	.	.	.	.	.	.	.	.	.	.	.	.	.	.	.	.	.	.	.	.	.
Sisyrinchium patagonicum	.	.	.	.	.	.	.	.	.	.	.	.	.	.	.	.	.	.	.	.	.	.	.	.	.	1	.	.	.
Sisyrinchium pearcei	.	.	.	.	.	.	.	.	.	.	.	.	.	.	.	.	.	.	.	.	.	.	.	.	.	.	.	.	.
Stellaria debilis	.	.	.	.	.	.	.	.	.	.	.	.	.	.	.	.	.	.	.	.	.	.	.	.	1	.	.	.	.
Stuckenia filiformis	1	.	1	.	1	.	1	.	.	.	.	.	.	.	.	.	.	.	1	.	.	.	1	.	.	.	.	.	.
Stuckenia striata	.	.	.	.	.	.	.	.	.	1	.	.	.	1	1	.	1	.	.	.	.	.	.	.	.	.	.	.	.
Symphyotrichum peteroanum	.	.	.	.	.	.	.	.	.	.	.	.	.	.	.	.	.	.	.	.	.	.	.	.	1	.	.	.	.
Symphyotrichum vahlii	.	.	.	.	.	.	.	.	.	.	.	.	.	.	.	.	.	.	.	.	.	.	.	.	1	1	.	.	.
Tetroncium magellanicum	.	.	.	.	.	.	.	.	.	.	.	.	.	.	.	.	.	.	.	.	.	.	.	.	.	.	.	.	.
Tribeles australis	.	.	.	.	.	.	.	.	.	.	.	.	.	.	.	.	.	.	.	.	.	.	.	.	.	.	.	.	.
Trifolium amabile	.	.	.	.	.	.	.	.	.	.	.	.	.	.	.	.	.	.	.	.	.	.	.	.	.	.	.	.	.
Trifolium polymorphum	.	.	.	.	.	.	.	.	.	.	.	.	.	.	.	.	.	.	.	.	.	.	.	.	.	.	.	.	.
Triglochin concinna	.	.	.	.	.	.	.	.	.	.	.	.	.	.	.	.	.	.	.	.	.	.	.	.	.	.	.	.	.
Triglochin palustris	.	.	.	.	.	.	.	.	1	.	.	.	.	.	.	1	1	.	.	.	.	.	.	.	.	.	1	.	.
Triglochin striata	.	1	1	.	1	1	1	.	.	.	.	1	1	.	1	.	1	.	.	.	.	.	.	.	.	.	.	.	.
Trisetum caudulatum	.	.	.	.	.	.	.	.	.	.	.	.	.	.	.	.	.	.	.	.	.	.	.	.	.	.	.	.	.
Trisetum preslei	.	.	.	.	.	.	.	.	.	.	.	.	.	.	.	.	.	.	.	.	.	.	.	.	.	.	.	.	.
Koeleria spicata	.	.	.	.	.	.	.	.	.	.	.	.	.	.	.	.	.	.	.	.	.	.	.	.	.	.	.	.	.
Utricularia gibba	.	.	.	.	.	.	.	.	.	.	.	.	.	.	.	.	.	.	.	.	.	.	.	.	.	.	.	.	.
Vahlodea atropurpurea	.	.	.	.	.	.	.	.	.	.	.	.	.	.	.	.	.	.	.	.	.	.	.	.	.	.	.	.	.

Bog	1	2	3	4	5	6	7	8	9	10	11	12	13	14	15	16	17	18	19	20	21	22	23	24	25	26	27	28	29
Valeriana fonckii	.	.	.	.	.	.	.	.	.	.	.	.	.	.	.	.	.	.	.	.	.	.	.	.	.	.	.	.	.
Valeriana macrorhiza	.	.	.	.	.	.	.	.	.	.	.	.	.	.	.	.	.	.	.	.	.	.	.	.	.	1	.	.	.
Viola pygmaea	.	.	.	.	.	.	.	.	.	.	.	.	.	.	.	.	.	.	.	.	.	.	.	.	.	.	.	.	.
Werneria apiculata	.	.	.	.	.	.	.	.	.	.	.	.	.	.	.	.	.	1	1	.	.	.	1	.	.	.	.	.	.
Werneria pinnatifida	1	.	1	1	.	.	.	1	1	1	1	1	1	1	1	1	.	.	.	.	1	.	1	1	.	.	.	.	.
Werneria pygmaea	.	.	.	1	1	.	.	1	1	1	1	1	1	1	1	1	1	1	1	1	1	1	1	1	.	.	1	1	1
Werneria solivifolia	.	.	.	.	.	.	.	1	1	1	1	1	1	1	1	1	.	1	1	1	1	1	1	1	.	.	.	.	.
Werneria spathulata	.	.	.	1	1	.	.	.	.	.	1	1	1	1	.	.	.	.	1	1	1	1	1	1	.	.	.	.	.
Xenophyllum incisum	.	.	.	.	.	.	.	.	.	.	.	.	.	1	1	.	1	.	.	.	.	.	.	.	.	.	.	.	.
Zameioscirpus atacamensis	.	.	.	.	.	.	.	.	.	.	.	1	1	.	1	.	1	.	.	.	.	.	.	.	.	.	.	.	.
Zameioscirpus gaimardiodes	.	.	.	.	.	.	.	.	.	.	.	.	.	.	.	.	.	.	.	.	.	.	.	.	.	.	.	.	.
Zameioscirpus muticus	.	.	.	.	.	.	.	1	1	1	1	1	1	1	1	1	1	.	.	.	.	.	.	.	.	.	1	1	1

**Bog**	**30**	**31**	**32**	**33**	**34**	**35**	**36**	**37**	**38**	**39**	**40**	**41**	**42**	**43**	**44**	**45**	**46**	**47**	**48**	**49**	**50**	**51**	**52**	**53**	**54**	**55**	**56**	**57**	**58**
**Operational zone**	**N**	**N**	**N**	**N**	**N**	**N**	**N**	**N**	**N**	**N**	**N**	**N**	**N**	**N**	**N**	**N**	**N**	**N**	**N**	**N**	**N**	**N**	**N**	**N**	**N**	**N**	**N**	**N**	**N**
**Cluster**	**1**	**2**	**4**	**4**	**4**	**4**	**4**	**4**	**2**	**4**	**4**	**4**	**4**	**2**	**2**	**2**	**2**	**2**	**2**	**2**	**2**	**4**	**2**	**4**	**2**	**2**	**1**	**4**	**4**
**Bioregion**	**T**	**T**	**T**	**T**	**T**	**T**	**T**	**T**	**T**	**T**	**T**	**T**	**T**	**T**	**T**	**T**	**T**	**T**	**T**	**T**	**T**	**T**	**T**	**T**	**T**	**T**	**N**	**T**	**T**
**Longitude**	**-68.27**	**-68.31**	**-68.41**	**-68.23**	**-68.11**	**-68.14**	**-68.13**	**-68.07**	**-68.22**	**-68.13**	**-68.07**	**-68.07**	**-68.55**	**-67.00**	**-66.97**	**-67.03**	**-66.98**	**-66.95**	**-66.94**	**-68.23**	**-67.18**	**-67.18**	**-67.18**	**-67.18**	**-67.37**	**-67.18**	**-67.51**	**-67.51**	**-67.52**
**Latitude**	**-27.64**	**-27.62**	**-27.67**	**-27.80**	**-27.47**	**-27.42**	**-27.18**	**-27.02**	**-27.62**	**-26.92**	**-27.02**	**-27.02**	**-27.61**	**-26.56**	**-26.58**	**-26.58**	**-26.54**	**-26.53**	**-26.64**	**-27.62**	**-26.69**	**-26.69**	**-26.69**	**-26.70**	**-25.81**	**-26.70**	**-25.74**	**-25.74**	**-25.72**
Acaena antarctica	.	.	.	.	.	.	.	.	.	.	.	.	.	.	.	.	.	.	.	.	.	.	.	.	.	.	.	.	.
Acaena macrocephala	.	.	.	.	.	.	.	.	.	.	.	.	.	.	.	.	.	.	.	.	.	.	.	.	.	.	.	.	.
Acaena magellanica	.	.	.	.	.	.	.	.	.	.	.	.	.	.	1	.	.	.	1	.	.	1	1	.	.	.	.	.	.
Acaena ovalifolia	.	.	.	.	.	.	.	.	.	.	.	.	.	.	.	.	.	.	.	.	.	.	.	.	.	.	.	.	.
Acaena pinnatifida	.	.	.	.	.	.	.	.	.	.	.	.	.	.	.	.	.	.	.	.	.	.	.	.	.	.	.	.	.
Adesmia retusa	.	.	.	.	.	.	.	.	.	.	.	.	.	.	.	.	.	.	.	.	.	.	.	.	.	.	.	.	.

Bog	30	31	32	33	34	35	36	37	38	39	40	41	42	43	44	45	46	47	48	49	50	51	52	53	54	55	56	57	58
Agrostis breviculmis	.	.	.	.	.	.	.	.	.	.	.	.	.	.	.	.	.	.	.	.	.	.	.	.	.	.	.	.	.
Agrostis imberbis	.	.	.	.	.	.	.	.	.	.	.	.	.	.	.	.	.	.	.	.	.	.	.	.	.	.	.	.	.
Agrostis meyenii	.	.	.	.	.	.	.	.	.	.	.	.	.	.	.	.	.	.	.	.	.	.	.	.	.	.	.	.	.
Agrostis perennans	.	.	.	.	.	.	.	.	.	.	.	.	.	.	.	.	.	.	.	.	.	.	.	.	.	.	.	.	.
Alchemilla pinnata	.	.	.	.	.	.	.	.	.	.	.	.	.	.	.	.	.	.	.	.	.	.	.	.	.	.	.	.	.
Alopecurus magellanicus	.	.	.	.	.	.	.	.	.	.	.	.	.	.	.	.	.	.	.	.	.	.	.	.	.	.	.	.	.
Amphiscirpus nevadensis	.	.	1	1	1	1	1	1	.	.	.	.	.	.	.	.	.	.	1	.	.	.	.	.	.	.	.	.	.
Anagallis alternifolia	.	.	.	.	.	.	.	.	.	.	.	.	.	.	.	.	1	1	.	.	.	.	.	.	.	.	.	.	.
Antennaria chilensis	.	.	.	.	.	.	.	.	.	.	.	.	.	.	.	.	.	.	.	.	.	.	.	.	.	.	.	.	.
Anthoxanthum redolens	.	.	.	.	.	.	.	.	.	.	.	.	.	.	.	.	.	.	.	.	.	.	.	.	.	.	.	.	.
Apium panul	.	.	.	.	.	.	.	.	.	.	.	.	.	.	.	.	.	.	.	.	.	.	.	.	.	.	.	.	.
Arenaria rivularis	.	.	.	.	.	.	.	.	.	.	.	.	.	.	.	.	.	.	.	.	.	.	.	.	.	.	.	.	.
Arenaria serpens	.	.	.	.	.	1	.	.	.	.	.	.	.	.	.	.	.	.	.	.	.	.	.	.	1	.	.	1	.
Arjona pusilla	.	.	.	.	.	.	.	.	.	.	.	.	.	.	.	.	.	.	.	.	.	.	.	.	.	.	.	.	.
Astragalus bustillosii	.	.	.	.	.	.	.	.	.	.	.	.	.	.	.	.	.	.	.	.	.	.	.	.	.	.	.	.	.
Astragalus micranthellus	.	.	.	.	.	.	.	.	.	.	.	.	.	.	.	.	.	.	.	.	.	.	.	.	.	.	.	.	.
Azolla filiculoides	.	.	.	.	.	.	.	.	.	.	.	.	.	.	.	.	.	.	.	.	.	.	.	.	.	.	.	.	.
Azorella boelckei	.	.	.	.	.	.	.	.	.	.	.	.	.	.	.	.	.	.	.	.	.	.	.	.	.	.	.	.	.
Azorella burkartii	.	.	.	.	.	.	.	.	.	.	.	.	.	.	.	.	.	.	.	.	.	.	.	.	.	.	.	.	.
Azorella cryptantha	.	.	.	.	.	.	.	.	.	.	.	.	.	.	.	.	.	.	.	.	.	.	.	.	.	.	.	.	.
Azorella lycopodioides	.	.	.	.	.	.	.	.	.	.	.	.	.	.	.	.	.	.	.	.	.	.	.	.	.	.	.	.	.
Azorella trifoliolata	.	.	.	.	.	.	.	.	.	.	.	.	.	.	.	.	.	.	.	.	.	.	.	.	.	.	.	.	.
Baccharis acaulis	.	.	.	.	.	.	.	.	.	.	.	.	.	.	.	.	.	.	1	.	.	.	.	.	.	.	.	.	.
Baccharis caespitosa	.	.	.	.	.	.	.	.	.	.	.	.	.	.	.	.	.	.	.	.	.	.	.	.	.	.	.	.	.
Baccharis magellanica	.	.	.	.	.	.	.	.	.	.	.	.	.	.	.	.	.	.	.	.	.	.	.	.	.	.	.	.	.
Belloa chilensis	.	.	.	.	.	.	.	.	.	.	.	.	.	.	.	.	.	.	.	.	.	.	.	.	.	.	.	.	.
Bromus catharticus	.	.	.	.	.	.	.	.	.	.	.	.	.	.	.	.	.	.	.	.	.	.	.	.	.	.	.	.	.
Calandrinia acaulis	.	.	.	.	.	.	.	.	.	.	.	.	.	.	.	.	.	.	.	.	.	.	.	.	.	.	.	.	.

Bog	30	31	32	33	34	35	36	37	38	39	40	41	42	43	44	45	46	47	48	49	50	51	52	53	54	55	56	57	58
Calandrinia compacta	.	.	.	.	.	.	.	.	.	.	.	.	.	.	.	.	.	.	.	.	.	.	.	.	.	.	.	.	.
Calceolaria biflora	.	.	.	.	.	.	.	.	.	.	.	.	.	.	.	.	.	.	.	.	.	.	.	.	.	.	.	.	.
Calceolaria cana	.	.	.	.	.	.	.	.	.	.	.	.	.	.	.	.	.	.	.	.	.	.	.	.	.	.	.	.	.
Calceolaria corymbosa	.	.	.	.	.	.	.	.	.	.	.	.	.	.	.	.	.	.	.	.	.	.	.	.	.	.	.	.	.
Calceolaria filicaulis	.	.	.	.	.	.	.	.	.	.	.	.	.	.	.	.	.	.	.	.	.	.	.	.	.	.	.	.	.
Callitriche lechleri	.	.	.	.	.	.	.	.	.	.	.	.	.	.	.	.	.	.	.	.	.	.	.	.	.	.	.	.	.
Caltha appendiculata	.	.	.	.	.	.	.	.	.	.	.	.	.	.	.	.	.	.	.	.	.	.	.	.	.	.	.	.	.
Caltha sagittata	.	.	.	.	.	.	.	.	.	.	.	.	.	.	.	.	.	.	.	.	.	.	.	.	.	.	.	.	.
Cardamine cordata	.	.	.	.	.	.	.	.	.	.	.	.	.	.	.	.	.	.	.	.	.	.	.	.	.	.	.	.	.
Cardamine glacialis	.	.	.	.	.	.	.	.	.	.	.	.	.	.	.	.	.	.	.	.	.	.	.	.	.	.	.	.	.
Cardamine tenuirostris	.	.	.	.	.	.	.	.	.	.	.	.	.	.	.	.	.	.	.	.	.	.	.	.	.	.	.	.	.
Cardamine volckmannii	.	.	.	.	.	.	.	.	.	.	.	.	.	.	.	.	.	.	.	.	.	.	.	.	.	.	.	.	.
Carex acaulis	.	.	.	.	.	.	.	.	.	.	.	.	.	.	.	.	.	.	.	.	.	.	.	.	.	.	.	.	.
Carex atropicta	.	.	.	.	.	.	.	.	.	.	.	.	.	.	.	.	.	.	.	.	.	.	.	.	.	.	.	.	.
Carex banksii	.	.	.	.	.	.	.	.	.	.	.	.	.	.	.	.	.	.	.	.	.	.	.	.	.	.	.	.	.
Carex caduca	.	.	.	.	.	.	.	.	.	.	.	.	.	.	.	.	.	.	.	.	.	.	.	.	.	.	.	.	.
Carex decidua	.	.	.	.	.	.	.	.	.	.	.	.	.	.	.	.	.	.	.	.	.	.	.	.	.	.	.	.	.
Carex fuscula	.	.	.	.	.	.	.	.	.	.	.	.	.	.	.	.	.	.	.	.	.	.	.	.	.	.	.	.	.
Carex gayana	.	1	.	1	.	.	.	1	1	1	.	.	.	1	1	1	1	1	1	.	.	1	1	1	1	1	.	.	.
Carex hypoleucos	.	.	.	.	.	.	.	.	.	.	.	.	.	.	.	.	.	.	.	.	.	.	.	.	.	.	.	.	.
Carex macloviana	.	.	.	.	.	.	.	.	.	.	.	.	.	.	.	.	.	.	.	.	.	.	.	.	.	.	.	.	.
Carex magellanica	.	.	.	.	.	.	.	.	.	.	.	.	.	.	.	.	.	.	.	.	.	.	.	.	.	.	.	.	.
Carex malmei	.	.	.	.	.	.	.	.	.	.	.	.	.	.	.	.	.	.	.	.	.	.	.	.	.	.	.	.	.
Carex maritima	.	1	1	.	.	.	1	1	1	1	1	1	.	.	1	.	.	1	1	.	1	1	1	.	.	1	.	1	1
Carex microglochin	1	1	.	.	.	.	.	.	1	.	.	.	.	.	.	.	.	.	.	.	.	.	.	.	.	.	.	.	.
Carex pleioneura	.	.	.	.	.	.	.	.	.	.	.	.	.	.	.	.	.	.	.	.	.	.	.	.	.	.	.	.	.
Carex ruthsatzae	.	.	.	.	.	.	.	.	.	.	.	.	.	.	.	.	.	.	.	.	.	.	.	.	.	.	.	.	.
Carex vallis-pulchrae	.	1	.	.	.	.	.	.	.	.	.	.	.	1	1	.	1	.	.	.	.	.	.	.	.	.	.	1	.
Carpha schoenoides	.	.	.	.	.	.	.	.	.	.	.	.	.	.	.	.	.	.	.	.	.	.	.	.	.	.	.	.	.
Castilleja pumila	.	.	.	.	.	.	.	.	.	.	.	.	.	1	.	1	.	.	.	.	.	.	.	.	.	.	.	.	.

Bog	30	31	32	33	34	35	36	37	38	39	40	41	42	43	44	45	46	47	48	49	50	51	52	53	54	55	56	57	58
Catabrosa werdermannii	.	.	.	1	.	1	.	1	.	1	1	1	1	.	.	.	.	.	.	.	.	.	.	.	.	.	.	.	.
Cerastium humifusum	.	.	.	.	.	.	.	.	.	.	.	.	.	.	.	.	.	.	.	.	.	.	.	.	.	.	.	.	.
Cerastium montioides	.	.	.	.	.	.	.	.	.	.	.	.	.	.	.	.	.	.	.	.	.	.	.	.	.	.	.	.	.
Chiliotrichum diffusum	.	.	.	.	.	.	.	.	.	.	.	.	.	.	.	.	.	.	.	.	.	.	.	.	.	.	.	.	.
Chusquea culeou	.	.	.	.	.	.	.	.	.	.	.	.	.	.	.	.	.	.	.	.	.	.	.	.	.	.	.	.	.
Colobanthus quitensis	.	1	.	.	.	.	.	.	1	.	.	.	.	.	1	1	1	.	1	.	1	.	.	.	.	.	.	1	.
Cortaderia egmontiana	.	.	.	.	.	.	.	.	.	.	.	.	.	.	.	.	.	.	.	.	.	.	.	.	.	.	.	.	.
Cotula mexicana	.	.	.	.	.	.	.	.	.	.	.	.	.	.	1	1	1	1	1	.	.	.	1	.	1	.	.	.	.
Crassula peduncularis	.	.	.	.	.	.	.	.	.	.	.	.	.	.	.	.	.	.	.	.	.	.	.	.	.	.	.	.	.
Cuatrecasasiella argentina	.	.	.	.	.	.	.	.	.	.	.	.	.	.	.	.	.	.	.	.	.	.	.	.	.	.	.	.	.
Deschampsia antarctica	.	.	.	.	.	.	.	.	.	.	.	.	.	.	.	.	.	.	.	.	.	.	.	.	.	.	.	.	.
Deschampsia caespitosa	.	.	.	.	.	.	.	.	.	.	.	.	.	.	.	.	.	.	.	.	.	.	.	.	.	.	.	.	.
Deschampsia patula	.	.	.	.	.	.	.	.	.	.	.	.	.	.	.	.	.	.	.	.	.	.	.	.	.	.	.	.	.
Cinnagrostis brevifolia	.	.	.	.	.	.	.	.	.	.	.	.	.	.	.	.	.	.	.	.	.	.	.	.	.	.	.	.	.
Deschampsia chrysantha	.	.	.	.	.	.	.	.	.	.	.	.	.	.	.	.	.	.	.	.	.	.	.	.	.	.	.	.	.
Cinnagrostis chrysophylla	.	.	.	.	.	.	.	.	.	.	.	.	.	.	.	.	.	.	.	.	.	.	.	.	.	.	.	.	.
Deschampsia chrysostachya	.	.	.	.	.	.	.	.	.	.	.	.	.	.	.	.	.	.	.	.	.	.	.	.	.	.	.	.	.
Deschampsia eminens	1	1	.	1	.	.	1	1	.	.	1	1	.	1	.	1	1	.	.	1	.	.	.	.	.	.	.	.	.
Deschampsia hackelii	.	.	.	.	.	.	.	.	.	.	.	.	.	.	.	.	.	.	.	.	.	.	.	.	.	.	.	.	1
Cinnagrostis minima	.	.	.	.	.	.	.	.	.	.	.	.	.	.	.	.	.	.	.	.	.	.	.	.	.	.	.	.	.
Deschampsia ovata	.	.	.	.	.	.	.	.	.	.	.	.	.	.	.	.	.	.	.	.	.	.	.	.	.	.	.	.	.
Cinnagrostis rigescens	.	.	.	.	.	.	.	.	.	.	.	.	.	1	1	1	.	.	.	.	.	.	.	.	.	.	.	.	.
Cinnagrostis spicigera	.	.	.	.	.	.	.	.	.	.	.	.	.	.	.	.	.	.	.	.	.	.	.	.	.	.	.	.	.
Cinnagrostis velutina	.	.	.	.	.	.	.	.	.	.	.	.	.	.	.	.	.	.	.	.	.	.	.	.	.	.	.	.	.
Cinnagrostis vicunarum	.	.	.	.	.	.	.	.	.	.	.	.	.	.	.	.	.	.	.	.	.	.	.	.	.	.	.	.	.
Distichia filamentosa	.	.	.	.	.	.	.	.	.	.	.	.	.	.	.	.	.	.	.	.	.	.	.	.	.	.	.	.	.
Distichia muscoides	.	.	.	.	.	.	.	.	.	.	.	.	.	.	.	.	.	.	.	.	.	.	.	.	.	.	.	.	.

Bog	30	31	32	33	34	35	36	37	38	39	40	41	42	43	44	45	46	47	48	49	50	51	52	53	54	55	56	57	58
Distichlis humilis	.	1	.	1	1	1	1	1	.	1	.	.	1	.	.	.	.	.	1	1	.	.	.	.	.	.	.	.	.
Distichlis scoparia	.	.	.	.	.	.	.	.	.	.	.	.	.	.	.	.	.	.	.	.	.	.	.	.	.	.	.	.	.
Distichlis spicata	.	.	.	.	.	.	.	.	.	.	.	.	.	.	.	.	.	.	.	.	.	.	.	.	.	.	.	.	.
Draba pusilla	.	.	.	.	.	.	.	.	.	.	.	.	.	.	.	.	.	.	.	.	.	.	.	.	.	.	.	.	.
Eleocharis melanomphala	.	1	.	1	.	.	.	1	1	1	.	.	.	.	.	.	1	1	1	1	1	.	1	1	.	.	.	.	.
Eleocharis pseudoalbibracteata	1	1	.	1	1	.	1	1	1	.	.	1	.	1	1	1	1	1	1	.	1	.	1	1	1	1	.	1	1
Elodea potamogeton	.	.	.	.	.	.	.	.	.	.	.	.	.	.	.	.	.	.	.	.	.	.	.	.	.	.	.	.	.
Empetrum rubrum	.	.	.	.	.	.	.	.	.	.	.	.	.	.	.	.	.	.	.	.	.	.	.	.	.	.	.	.	.
Epilobium australe	.	.	.	.	.	.	.	.	.	.	.	.	.	.	.	.	.	.	.	.	.	.	.	.	.	.	.	.	.
Epilobium barbeyanum	.	.	.	.	.	.	.	.	.	.	.	.	.	.	.	.	.	.	.	.	.	.	.	.	.	.	.	.	.
Epilobium ciliatum	.	.	.	.	.	.	.	.	.	.	.	.	.	.	.	.	.	.	.	.	.	.	.	.	.	.	.	.	.
Epilobium denticulatum	.	.	.	.	.	.	.	.	.	.	.	.	.	.	.	.	.	.	.	.	.	.	.	.	.	.	.	.	.
Epilobium fragile	.	.	.	.	.	.	.	.	.	.	.	.	.	.	.	.	.	.	.	.	.	.	.	.	.	.	.	.	.
Epilobium glaucum	.	.	.	.	.	.	.	.	.	.	.	.	.	.	.	.	.	.	.	.	.	.	.	.	.	.	.	.	.
Epilobium nivale	.	.	.	.	.	.	.	.	.	.	.	.	.	.	.	.	.	.	.	.	.	.	.	.	.	.	.	.	.
Erigeron andicola	.	.	.	.	.	.	.	.	.	.	.	.	.	.	.	.	.	.	.	.	.	.	.	.	.	.	.	.	.
Erigeron leptopetalus	.	.	.	.	.	.	.	.	.	.	.	.	.	.	.	.	.	.	.	.	.	.	.	.	.	.	.	.	.
Erigeron myosotis	.	.	.	.	.	.	.	.	.	.	.	.	.	.	.	.	.	.	.	.	.	.	.	.	.	.	.	.	.
Erigeron patagonicus	.	.	.	.	.	.	.	.	.	.	.	.	.	.	.	.	.	.	.	.	.	.	.	.	.	.	.	.	.
Erythranthe cuprea	.	.	.	.	.	.	.	.	.	.	.	.	.	.	.	.	.	.	.	.	.	.	.	.	.	.	.	.	.
Erythranthe depressa	.	.	.	1	.	1	1	.	.	.	.	.	.	1	.	1	1	1	.	1	.	.	1	.	1	.	.	.	.
Erythranthe glabrata	1	.	.	.	.	.	.	.	.	.	.	.	.	.	.	.	.	.	.	.	.	.	.	.	.	.	.	.	.
Erythranthe lutea	.	.	.	.	.	.	.	.	.	.	.	.	.	.	.	.	.	.	.	.	.	.	.	.	.	.	.	.	.
Escallonia virgata	.	.	.	.	.	.	.	.	.	.	.	.	.	.	.	.	.	.	.	.	.	.	.	.	.	.	.	.	.
Euphrasia antarctica	.	.	.	.	.	.	.	.	.	.	.	.	.	.	.	.	.	.	.	.	.	.	.	.	.	.	.	.	.
Euphrasia chrysantha	.	.	.	.	.	.	.	.	.	.	.	.	.	.	.	.	.	.	.	.	.	.	.	.	.	.	.	.	.
Euphrasia subexserta	.	.	.	.	.	.	.	.	.	.	.	.	.	.	.	.	.	.	.	.	.	.	.	.	.	.	.	.	.
Festuca hypsophila	.	.	.	.	.	.	.	.	.	.	.	.	.	.	.	.	.	.	.	.	.	.	.	.	.	.	.	.	.
Festuca kurtziana	.	.	.	.	.	.	.	.	.	.	.	.	.	.	.	.	.	.	.	.	.	.	.	.	.	.	.	.	.

**Bog**	**30**	**31**	**32**	**33**	**34**	**35**	**36**	**37**	**38**	**39**	**40**	**41**	**42**	**43**	**44**	**45**	**46**	**47**	**48**	**49**	**50**	**51**	**52**	**53**	**54**	**55**	**56**	**57**	**58**
Festuca lilloi	.	.	.	.	.	.	.	.	.	.	.	.	.	.	.	.	.	.	.	.	.	.	.	.	.	.	.	.	.
Festuca magellanica	.	.	.	.	.	.	.	.	.	.	.	.	.	.	.	.	.	.	.	.	.	.	.	.	.	.	.	.	.
Festuca nardifolia	.	.	.	.	.	.	.	.	.	.	.	.	.	.	.	.	.	.	.	.	.	.	.	.	.	.	.	.	.
Festuca rigescens	.	.	.	.	.	.	.	.	.	.	.	.	.	.	.	.	.	.	.	.	.	.	.	.	.	.	.	.	.
Festuca werdermannii	.	.	.	.	.	.	.	.	.	.	.	.	.	.	.	.	.	.	.	.	.	.	.	.	.	.	.	.	.
Frankenia triandra	.	.	.	.	.	.	.	.	.	.	.	.	.	.	.	.	.	.	.	.	.	.	.	.	.	.	.	.	.
Gamocarpha graminea	.	.	.	.	.	.	.	.	.	.	.	.	.	.	.	.	.	.	.	.	.	.	.	.	.	.	.	.	.
Gamocarpha ventosa	.	.	.	.	.	.	.	.	.	.	.	.	.	.	.	.	.	.	.	.	.	.	.	.	.	.	.	.	.
Gamochaeta chamissonis	.	.	.	.	.	.	.	.	.	.	.	.	.	.	.	.	.	.	.	.	.	.	.	.	.	.	.	.	.
Gamochaeta longipedicellata	.	.	.	.	.	.	.	.	.	.	.	.	.	.	.	.	.	.	.	.	.	.	.	.	.	.	.	.	.
Gamochaeta neuquensis	.	.	.	.	.	.	.	.	.	.	.	.	.	.	.	.	.	.	.	.	.	.	.	.	.	.	.	.	.
Gaultheria antarctica	.	.	.	.	.	.	.	.	.	.	.	.	.	.	.	.	.	.	.	.	.	.	.	.	.	.	.	.	.
Gaultheria caespitosa	.	.	.	.	.	.	.	.	.	.	.	.	.	.	.	.	.	.	.	.	.	.	.	.	.	.	.	.	.
Gaultheria pumila	.	.	.	.	.	.	.	.	.	.	.	.	.	.	.	.	.	.	.	.	.	.	.	.	.	.	.	.	.
Gavilea chica	.	.	.	.	.	.	.	.	.	.	.	.	.	.	.	.	.	.	.	.	.	.	.	.	.	.	.	.	.
Gentiana prostrata	1	1	.	.	.	.	.	1	1	.	1	1	.	1	1	1	1	1	1	1	1	1	1	1	1	1	1	1	.
Gentianella fiebrigii	.	.	.	.	.	.	.	.	.	.	.	.	.	.	.	.	.	.	.	.	.	.	.	.	1	1	.	.	.
Gentianella magellanica	.	.	.	.	.	.	.	.	.	.	.	.	.	.	.	.	.	.	.	.	.	.	.	.	.	.	.	.	.
Gentianella multicaulis	.	.	.	.	.	.	.	.	.	.	.	.	.	.	.	.	.	.	.	.	.	.	.	.	.	.	.	.	.
Gentianella ottonis	.	.	.	.	.	.	.	.	.	.	.	.	.	.	.	.	.	.	.	.	.	.	.	.	.	.	.	.	.
Gentianella primuloides	.	.	.	.	.	.	.	.	.	.	.	.	.	.	.	.	.	.	.	.	.	.	.	.	.	.	.	.	.
Gentianella pseudocrassula	.	.	.	.	.	.	.	.	.	.	.	.	.	.	.	.	1	1	.	.	.	.	.	.	.	.	.	.	.
Geranium sessiliflorum	.	.	.	.	.	.	.	.	.	.	.	.	.	.	.	.	.	.	.	.	.	.	.	.	.	.	.	.	.
Gunnera magellanica	.	.	.	.	.	.	.	.	.	.	.	.	.	.	.	.	.	.	.	.	.	.	.	.	.	.	.	.	.
Halenia caespitosa	.	.	.	.	.	.	.	.	.	.	.	.	.	.	.	.	.	.	.	.	.	.	.	.	.	.	.	.	.
Halerpestes cymbalaria	.	.	.	1	1	.	1	.	.	.	1	.	.	1	1	1	.	1	1	.	1	1	1	1	1	.	.	.	.
Halerpestes exilis	.	.	1	.	.	.	.	.	.	.	.	.	.	.	.	.	.	.	.	.	.	.	.	.	.	1	.	.	1

Bog	30	31	32	33	34	35	36	37	38	39	40	41	42	43	44	45	46	47	48	49	50	51	52	53	54	55	56	57	58
Hieracium antarcticum	.	.	.	.	.	.	.	.	.	.	.	.	.	.	.	.	.	.	.	.	.	.	.	.	.	.	.	.	.
Hordeum comosum	.	.	.	.	.	.	.	.	.	.	.	.	.	.	.	.	.	.	.	.	.	.	.	.	.	.	.	.	.
Hordeum muticum	.	1	.	1	1	.	.	.	1	.	.	.	.	1	1	1	1	1	1	1	1	1	.	1	1	1	.	.	.
Hypochaeris acaulis	.	.	.	.	.	.	.	.	.	.	.	.	.	.	.	.	.	.	.	.	.	.	.	.	.	.	.	.	.
Hypochaeris chondrilloides	.	.	.	.	.	.	.	.	.	.	.	.	.	.	.	.	.	1	1	.	.	.	1	.	.	.	.	.	.
Hypochaeris meyeniana	.	.	.	.	.	.	.	.	.	.	.	.	.	.	.	.	1	1	.	.	.	1	1	1	.	.	.	.	.
Hypochaeris palustris	.	.	.	.	.	.	.	.	.	.	.	.	.	.	.	.	.	.	.	.	.	.	.	.	.	.	.	.	.
Hypochaeris taraxacoides	.	.	.	.	.	.	.	.	.	.	.	.	.	.	.	.	.	.	.	.	.	.	.	.	.	.	.	.	.
Hypochaeris tenerifolia	.	.	.	.	.	.	.	.	.	.	.	.	.	.	.	.	.	.	.	.	.	.	.	.	.	.	.	.	.
Isolepis nigricans	.	.	.	.	.	.	.	.	.	.	.	.	.	.	.	.	.	.	.	.	.	.	.	.	.	.	.	.	.
Isolepsis inundata	.	.	.	.	.	.	.	.	.	.	.	.	.	.	.	.	.	.	.	.	.	.	.	.	.	.	.	.	.
Juncus balticus	.	1	.	1	1	1	1	1	1	1	.	.	1	1	1	1	1	1	1	1	1	1	1	1	1	1	.	.	.
Juncus stipulatus	.	.	.	.	.	.	1	.	.	.	.	.	.	.	1	1	1	.	1	.	1	1	1	.	1	.	.	1	.
Koeleria kurtzii	.	.	.	.	.	.	.	.	.	.	.	.	.	.	.	.	.	.	.	.	.	.	.	.	.	.	.	.	.
Lachemilla diplophylla	.	.	.	.	.	.	.	.	.	.	.	.	.	.	.	.	.	.	.	.	.	.	.	.	.	.	.	.	.
Lachemilla pinnata	.	.	.	.	.	.	.	.	.	.	.	.	.	.	.	.	.	.	.	.	.	.	.	.	.	.	.	.	.
Lagenophora nudicaulis	.	.	.	.	.	.	.	.	.	.	.	.	.	.	.	.	.	.	.	.	.	.	.	.	.	.	.	.	.
Lemna minuta	.	.	.	.	.	.	.	.	.	1	.	.	.	.	.	.	.	.	.	.	.	.	.	.	.	.	.	.	.
Leptinella scariosa	.	.	.	.	.	.	.	.	.	.	.	.	.	.	.	.	.	.	.	.	.	.	.	.	.	.	.	.	.
Leucheria candidissima	.	.	.	.	.	.	.	.	.	.	.	.	.	.	.	.	.	.	.	.	.	.	.	.	.	.	.	.	.
Leucheria nutans	.	.	.	.	.	.	.	.	.	.	.	.	.	.	.	.	.	.	.	.	.	.	.	.	.	.	.	.	.
Lilaea scilloides	.	.	.	.	.	.	.	.	.	.	.	.	.	.	.	.	.	.	.	.	.	.	.	.	.	.	.	.	.
Lilaeopsis macloviana	.	1	.	1	1	.	1	.	.	1	.	.	.	.	.	1	.	1	1	.	.	1	.	1	.	.	.	.	1
Limosella australis	.	.	.	.	.	.	.	.	.	.	.	.	.	.	.	.	.	.	.	.	.	.	.	.	.	.	.	.	.
Lobelia oligophylla	.	1	.	1	.	.	1	.	.	1	1	.	.	1	1	1	1	1	1	1	1	.	1	1	1	1	.	1	1
Luzula brachyphylla	.	.	.	.	.	.	.	.	.	.	.	.	.	.	.	.	.	.	.	.	.	.	.	.	.	.	.	.	.
Luzula chilensis	.	.	.	.	.	.	.	.	.	.	.	.	.	.	.	.	.	.	.	.	.	.	.	.	.	.	.	.	.
Luzula racemosa	.	.	.	.	.	.	.	.	.	.	.	.	.	.	.	.	.	.	.	.	.	.	.	.	.	.	.	.	.
Luzula vulcanica	.	.	.	.	.	.	.	.	.	.	.	.	.	.	.	.	.	.	.	.	.	.	.	.	.	.	.	.	.

**Bog**	**30**	**31**	**32**	**33**	**34**	**35**	**36**	**37**	**38**	**39**	**40**	**41**	**42**	**43**	**44**	**45**	**46**	**47**	**48**	**49**	**50**	**51**	**52**	**53**	**54**	**55**	**56**	**57**	**58**
Lysipomia pumila	.	.	.	.	.	.	.	.	.	.	.	.	.	.	.	.	.	.	.	.	.	.	.	.	.	.	.	.	.
Marsippospermum philippii	.	.	.	.	.	.	.	.	.	.	.	.	.	.	.	.	.	.	.	.	.	.	.	.	.	.	.	.	.
Marsippospermum reichei	.	.	.	.	.	.	.	.	.	.	.	.	.	.	.	.	.	.	.	.	.	.	.	.	.	.	.	.	.
Montia fontana	.	.	.	.	.	.	.	.	.	.	.	.	.	.	.	.	.	.	.	.	.	.	.	.	.	.	.	.	.
Muhlenbergia asperifolia	.	.	.	.	.	.	.	.	.	.	.	.	.	.	.	.	.	1	1	.	.	.	.	.	.	.	.	.	.
Myriophyllum quitense	.	.	.	.	.	1	1	.	.	.	.	.	.	.	.	.	.	.	.	.	.	.	.	.	1	1	.	.	1
Myrosmodes nervosa	.	.	.	.	.	.	.	.	.	.	.	.	.	.	.	.	.	.	.	.	.	.	.	.	.	.	.	.	.
Myrosmodes paludosa	.	.	.	.	.	.	.	.	.	.	.	.	.	1	.	1	.	.	.	1	.	.	.	.	.	.	.	.	.
Myrteola nummularia	.	.	.	.	.	.	.	.	.	.	.	.	.	.	.	.	.	.	.	.	.	.	.	.	.	.	.	.	.
Nanodea muscosa	.	.	.	.	.	.	.	.	.	.	.	.	.	.	.	.	.	.	.	.	.	.	.	.	.	.	.	.	.
Neobartsia crenoloba	.	.	.	.	.	.	.	.	.	.	.	.	.	.	.	.	.	.	.	.	.	.	.	.	.	.	.	.	.
Neobartsia pedicularoides	.	.	.	.	.	.	.	.	.	.	.	.	.	.	.	.	.	.	.	.	.	.	.	.	.	.	.	.	.
Neobartsia peruviana	.	.	.	.	.	.	.	.	.	.	.	.	.	1	1	1	1	1	.	.	1	.	.	.	.	.	.	.	.
Nertera granadensis	.	.	.	.	.	.	.	.	.	.	.	.	.	.	.	.	.	.	.	.	.	.	.	.	.	.	.	.	.
Nicoraepoa andina	.	.	.	.	.	.	.	.	.	.	.	.	.	.	.	.	.	.	.	.	.	.	.	.	.	.	.	.	.
Nicoraepoa pugionifolia	.	.	.	.	.	.	.	.	.	.	.	.	.	.	.	.	.	.	.	.	.	.	.	.	.	.	.	.	.
Nicoraepoa subenervis	.	.	.	.	.	.	.	.	.	.	.	.	.	.	.	.	.	.	.	.	.	.	.	.	.	.	.	.	.
Nitrophila australis	.	.	.	1	1	.	.	1	.	.	.	.	1	.	.	.	.	.	.	.	.	.	.	1	.	.	.	.	.
Nothofagus antarctica	.	.	.	.	.	.	.	.	.	.	.	.	.	.	.	.	.	.	.	.	.	.	.	.	.	.	.	.	.
Nototriche rugosa	.	.	.	.	.	.	.	.	.	.	.	.	.	.	.	.	.	.	.	.	.	.	.	.	.	.	.	.	.
Ochetophila nana	.	.	.	.	.	.	.	.	.	.	.	.	.	.	.	.	.	.	.	.	.	.	.	.	.	.	.	.	.
Olsynium junceum	.	.	.	.	.	.	.	.	.	.	.	.	.	.	.	.	.	.	.	.	.	.	.	.	.	.	.	.	.
Oreobolus obtusangulus	.	.	.	.	.	.	.	.	.	.	.	.	.	.	.	.	.	.	.	.	.	.	.	.	.	.	.	.	.
Oritrophium limnophilum	.	.	.	.	.	.	.	.	.	.	.	.	.	.	.	.	.	.	.	.	.	.	.	.	.	.	.	.	.
Osmorhiza glabrata	.	.	.	.	.	.	.	.	.	.	.	.	.	.	.	.	.	.	.	.	.	.	.	.	.	.	.	.	.
Ourisia alpina	.	.	.	.	.	.	.	.	.	.	.	.	.	.	.	.	.	.	.	.	.	.	.	.	.	.	.	.	.

Bog	30	31	32	33	34	35	36	37	38	39	40	41	42	43	44	45	46	47	48	49	50	51	52	53	54	55	56	57	58
Ourisia muscosa	.	.	.	.	.	.	.	.	.	.	.	.	.	.	.	.	.	.	.	.	.	.	.	.	.	.	.	.	.
Ourisia ruelloides	.	.	.	.	.	.	.	.	.	.	.	.	.	.	.	.	.	.	.	.	.	.	.	.	.	.	.	.	.
Oxychloe andina	1	1	.	.	.	.	.	1	1	1	1	1	.	1	.	.	.	.	.	1	1	1	1	1	.	.	1	1	1
Oxychloe bisexualis	.	.	.	.	.	.	.	.	.	.	.	.	.	.	.	.	.	.	.	.	.	.	.	.	.	.	.	.	.
Oxychloe castellanosii	.	.	.	.	.	.	.	.	.	.	.	.	.	.	.	.	.	.	.	.	.	.	.	.	.	.	.	.	.
Oxychloe haumaniana	.	.	.	.	.	.	.	.	.	.	.	.	.	.	.	.	.	.	.	.	.	.	.	.	.	.	.	.	.
Oxychloe mendocina	.	.	.	.	.	.	.	.	.	.	.	.	.	.	.	.	.	.	.	.	.	.	.	.	.	.	.	.	.
Patosia clandestina	.	.	.	.	.	.	.	.	.	.	.	.	.	1	1	1	1	1	.	.	.	.	.	.	.	.	.	.	.
Perezia capito	.	.	.	.	.	.	.	.	.	.	.	.	.	.	.	.	.	.	.	.	.	.	.	.	.	.	.	.	.
Perezia delicata	.	.	.	.	.	.	.	.	.	.	.	.	.	.	.	.	.	.	.	.	.	.	.	.	.	.	.	.	.
Perezia fonkii	.	.	.	.	.	.	.	.	.	.	.	.	.	.	.	.	.	.	.	.	.	.	.	.	.	.	.	.	.
Perezia pedicularidifolia	.	.	.	.	.	.	.	.	.	.	.	.	.	.	.	.	.	.	.	.	.	.	.	.	.	.	.	.	.
Perezia pinnatifida	.	.	.	.	.	.	.	.	.	.	.	.	.	.	.	.	.	.	.	.	.	.	.	.	.	.	.	.	.
Petroravenia friesii	.	.	.	.	.	.	.	.	.	.	.	.	.	.	.	.	.	.	.	.	.	.	.	.	.	.	.	.	.
Petroravenia werdermannii	.	.	.	.	.	.	.	.	.	.	.	.	.	.	.	.	.	.	.	.	.	.	.	.	.	.	.	.	.
Phleum alpinum	.	.	.	.	.	.	.	.	.	.	.	.	.	.	.	.	.	.	.	.	.	.	.	.	.	.	.	.	.
Phylloscirpus acaulis	.	1	.	1	.	.	1	.	1	.	.	.	.	1	1	.	1	1	1	1	1	.	.	.	1	.	.	1	.
Phylloscirpus boliviensis	.	.	.	.	.	.	.	.	.	.	.	.	.	.	.	.	.	.	.	.	.	.	.	.	.	.	.	.	.
Phylloscirpus deserticola	.	.	.	.	.	.	.	.	.	.	.	.	.	.	.	1	.	.	.	1	.	1	.	.	.	.	.	.	.
Pinguicula antarctica	.	.	.	.	.	.	.	.	.	.	.	.	.	.	.	.	.	.	.	.	.	.	.	.	.	.	.	.	.
Plantago barbata	.	.	.	.	.	.	.	.	.	.	.	.	.	.	.	.	.	.	.	.	.	.	.	.	.	.	.	.	.
Plantago rigida	.	.	.	.	.	.	.	.	.	.	.	.	.	.	.	.	.	.	.	.	.	.	.	.	.	.	.	.	.
Plantago tubulosa	.	.	.	.	.	.	.	.	.	.	.	.	.	1	1	1	.	.	.	1	.	.	.	.	.	.	.	.	.
Plantago uniglumis	.	.	.	.	.	.	.	.	.	.	.	.	.	.	.	.	.	.	.	.	.	.	.	.	.	.	.	.	.
Poa alopecurus	.	.	.	.	.	.	.	.	.	.	.	.	.	.	.	.	.	.	.	.	.	.	.	.	.	.	.	.	.
Poa hachadoensis	.	.	.	.	.	.	.	.	.	.	.	.	.	.	.	.	.	.	.	.	.	.	.	.	.	.	.	.	.
Poa perligulata	.	.	.	.	.	.	.	.	.	.	.	.	.	.	.	.	.	.	.	.	.	.	.	.	.	.	.	.	.
Polypogon interruptus	.	.	.	.	.	.	.	.	.	.	.	.	.	.	.	.	1	1	1	.	.	.	.	.	.	.	.	.	.

Bog	30	31	32	33	34	35	36	37	38	39	40	41	42	43	44	45	46	47	48	49	50	51	52	53	54	55	56	57	58
Primula magellanica	.	.	.	.	.	.	.	.	.	.	.	.	.	.	.	.	.	.	.	.	.	.	.	.	.	.	.	.	.
Puccinellia frigida	.	.	1	1	1	1	.	1	.	1	1	.	1	.	.	.	.	.	1	.	1	.	.	1	.	.	.	1	.
Quinchamalium chilense	.	.	.	.	.	.	.	.	.	.	.	.	.	.	.	.	.	.	.	.	.	.	.	.	.	.	.	.	.
Ranunculus breviscapus	.	.	.	.	.	.	.	.	.	.	.	.	.	.	.	.	.	.	.	.	.	.	.	.	.	.	.	.	.
Ranunculus fuegianus	.	.	.	.	.	.	.	.	.	.	.	.	.	.	.	.	.	.	.	.	.	.	.	.	.	.	.	.	.
Ranunculus mandonianus	.	.	.	.	.	.	.	.	.	.	.	.	.	.	.	.	.	.	.	.	.	.	.	.	.	.	.	.	.
Ranunculus peduncularis	.	.	.	.	.	.	.	.	.	.	.	.	.	.	.	.	.	.	.	.	.	.	.	.	.	.	.	.	.
Ranunculus trichophyllus	.	.	.	.	.	.	.	.	.	.	.	.	.	.	.	.	.	.	.	.	.	.	.	.	.	.	.	.	.
Halerpestes uniflora	.	1	.	.	1	1	1	1	.	.	.	.	.	.	.	.	.	.	.	.	.	.	.	.	1	1	.	.	.
Rubus geoides	.	.	.	.	.	.	.	.	.	.	.	.	.	.	.	.	.	.	.	.	.	.	.	.	.	.	.	.	.
Rumex magellanicus	.	.	.	.	.	.	.	.	.	.	.	.	.	.	.	.	.	.	.	.	.	.	.	.	.	.	.	.	.
Rytidosperma lechleri	.	.	.	.	.	.	.	.	.	.	.	.	.	.	.	.	.	.	.	.	.	.	.	.	.	.	.	.	.
Sarcocornia pulvinata	.	.	.	.	.	.	.	.	.	.	.	.	.	.	.	.	.	.	.	.	.	.	.	.	.	.	.	.	.
Schoenoplectus pungens	.	.	.	.	.	.	.	.	.	.	.	.	.	.	.	.	.	.	.	.	.	.	.	.	.	.	.	.	.
Schoenus andinus	.	.	.	.	.	.	.	.	.	.	.	.	.	.	.	.	.	.	.	.	.	.	.	.	.	.	.	.	.
Senecio breviscapus	.	.	.	.	.	.	.	.	.	.	.	.	.	.	.	.	.	.	.	.	.	.	.	.	.	.	.	.	.
Senecio diemii	.	.	.	.	.	.	.	.	.	.	.	.	.	.	.	.	.	.	.	.	.	.	.	.	.	.	.	.	.
Senecio fistulosus	.	.	.	.	.	.	.	.	.	.	.	.	.	.	.	.	.	.	.	.	.	.	.	.	.	.	.	.	.
Senecio parodii	.	.	.	.	.	.	.	.	.	.	.	.	.	.	.	.	.	.	.	.	.	.	.	.	.	.	.	.	.
Senecio peteroanus	.	.	.	.	.	.	.	.	.	.	.	.	.	.	.	.	.	.	.	.	.	.	.	.	.	.	.	.	.
Senecio serratifolius	.	.	.	.	.	.	.	.	.	.	.	.	.	.	.	.	.	.	.	.	.	.	.	.	.	.	.	.	.
Senecio trifurcatus	.	.	.	.	.	.	.	.	.	.	.	.	.	.	.	.	.	.	.	.	.	.	.	.	.	.	.	.	.
Sisyrinchium chilense	.	.	.	.	.	.	.	.	.	.	.	.	.	.	.	.	1	1	.	.	.	.	.	.	1	.	.	.	.
Sisyrinchium patagonicum	.	.	.	.	.	.	.	.	.	.	.	.	.	.	.	.	.	.	.	.	.	.	.	.	.	.	.	.	.
Sisyrinchium pearcei	.	.	.	.	.	.	.	.	.	.	.	.	.	.	.	.	.	.	.	.	.	.	.	.	.	.	.	.	.
Stellaria debilis	.	.	.	.	.	.	.	.	.	.	.	.	.	.	.	.	.	.	.	.	.	.	.	.	.	.	.	.	.
Stuckenia filiformis	.	1	.	1	.	1	1	1	.	1	1	1	1	.	.	.	.	.	.	.	.	1	.	1	.	.	.	1	.

Bog	30	31	32	33	34	35	36	37	38	39	40	41	42	43	44	45	46	47	48	49	50	51	52	53	54	55	56	57	58
Stuckenia striata	.	.	.	.	.	.	.	.	.	.	.	.	.	.	.	.	.	.	.	.	.	.	.	.	.	.	.	.	.
Symphyotrichum peteroanum	.	.	.	.	.	.	.	.	.	.	.	.	.	.	.	.	.	.	.	.	.	.	.	.	.	.	.	.	.
Symphyotrichum vahlii	.	.	.	.	.	.	.	.	.	.	.	.	.	.	.	.	.	.	.	.	.	.	.	.	.	.	.	.	.
Tetroncium magellanicum	.	.	.	.	.	.	.	.	.	.	.	.	.	.	.	.	.	.	.	.	.	.	.	.	.	.	.	.	.
Tribeles australis	.	.	.	.	.	.	.	.	.	.	.	.	.	.	.	.	.	.	.	.	.	.	.	.	.	.	.	.	.
Trifolium amabile	.	.	.	.	.	.	.	.	.	.	.	.	.	.	.	.	.	.	.	.	.	.	.	.	.	.	.	.	.
Trifolium polymorphum	.	.	.	.	.	.	.	.	.	.	.	.	.	.	.	.	.	.	.	.	.	.	.	.	.	.	.	.	.
Triglochin concinna	.	1	.	1	1	.	1	1	.	1	.	.	1	.	.	.	.	.	1	1	.	.	1	1	.	.	.	.	.
Triglochin palustris	.	1	.	1	.	.	.	.	.	.	.	.	.	.	.	.	1	.	1	1	.	.	1	1	1	.	.	.	.
Triglochin striata	.	.	.	.	.	.	.	.	.	.	.	.	.	.	.	.	.	.	.	.	.	.	.	.	.	.	.	.	.
Trisetum caudulatum	.	.	.	.	.	.	.	.	.	.	.	.	.	.	.	.	.	.	.	.	.	.	.	.	.	.	.	.	.
Trisetum preslei	.	.	.	.	.	.	.	.	.	.	.	.	.	.	.	.	.	.	.	.	.	.	.	.	.	.	.	.	.
Koeleria spicata	.	.	.	.	.	.	.	.	.	.	.	.	.	.	.	.	.	.	.	.	.	.	.	.	.	.	.	.	.
Utricularia gibba	.	.	.	.	.	.	.	.	.	.	.	.	.	.	.	.	.	.	.	.	.	.	.	.	.	.	.	.	.
Vahlodea atropurpurea	.	.	.	.	.	.	.	.	.	.	.	.	.	.	.	.	.	.	.	.	.	.	.	.	.	.	.	.	.
Valeriana fonckii	.	.	.	.	.	.	.	.	.	.	.	.	.	.	.	.	.	.	.	.	.	.	.	.	.	.	.	.	.
Valeriana macrorhiza	.	.	.	.	.	.	.	.	.	.	.	.	.	.	.	.	.	.	.	.	.	.	.	.	.	.	.	.	.
Viola pygmaea	.	.	.	.	.	.	.	.	.	.	.	.	.	.	.	.	.	.	.	.	.	.	.	.	.	.	.	.	.
Werneria apiculata	.	.	.	.	.	.	.	.	.	.	.	.	.	.	.	.	.	.	.	.	.	.	.	.	.	.	.	.	.
Werneria pinnatifida	.	.	.	.	.	.	.	.	.	.	.	.	.	.	.	.	.	.	.	.	.	.	.	.	.	.	.	1	1
Werneria pygmaea	.	1	.	.	.	.	.	.	1	.	.	.	.	1	1	.	1	.	.	1	.	.	1	.	1	1	.	1	.
Werneria solivifolia	.	.	.	.	.	.	.	.	.	.	.	.	.	.	.	.	.	.	.	.	.	.	.	.	.	.	.	.	.
Werneria spathulata	.	.	.	.	.	.	.	.	.	.	.	.	.	.	.	.	.	.	.	.	.	.	.	.	.	.	.	.	.
Xenophyllum incisum	.	.	.	.	.	.	.	.	.	.	.	.	.	.	.	.	.	.	.	.	.	.	.	.	.	.	.	.	.
Zameioscirpus atacamensis	.	.	.	.	.	.	.	.	.	.	.	.	1	.	.	.	.	.	.	.	.	.	.	.	.	.	.	.	.
Zameioscirpus gaimardiodes	.	.	.	.	.	.	.	.	.	.	.	.	.	.	.	.	.	.	.	.	.	.	.	.	.	.	.	.	.
Zameioscirpus muticus	1	1	1	.	.	.	1	1	1	1	1	1	1	1	1	1	1	.	1	1	1	1	1	1	1	1	1	1	1

Bog	59	60	61	62	63	64	65	66	67	68	69	70	71	72	73	74	75	76	77	78	79	80	81	82	83	84	85	86	87
**Operational zone**	**N**	**N**	**N**	**N**	**N**	**N**	**N**	**N**	**N**	**N**	**N**	**N**	**N**	**N**	**N**	**N**	**N**	**N**	**N**	**N**	**N**	**N**	**N**	**N**	**N**	**N**	**N**	**N**	**N**
**Cluster**	**4**	**2**	**4**	**4**	**4**	**4**	**4**	**2**	**4**	**4**	**4**	**4**	**2**	**4**	**4**	**4**	**2**	**2**	**2**	**2**	**1**	**1**	**1**	**1**	**1**	**1**	**1**	**1**	**1**
**Bioregion**	**T**	**T**	**T**	**T**	**T**	**T**	**T**	**T**	**T**	**T**	**T**	**T**	**T**	**T**	**T**	**T**	**T**	**T**	**T**	**T**	**N**	**N**	**N**	**N**	**N**	**N**	**N**	**N**	**N**
**Longitude**	**-67.51**	**-68.22**	**-67.53**	**-67.48**	**-67.49**	**-67.53**	**-67.50**	**-67.50**	**-67.73**	**-67.73**	**-67.69**	**-67.67**	**-68.22**	**-67.66**	**-67.64**	**-67.27**	**-68.23**	**-68.20**	**-68.22**	**-68.23**	**-66.40**	**-66.38**	**-66.38**	**-66.50**	**-66.50**	**-66.42**	**-66.92**	**-66.69**	**-66.78**
**Latitude**	**-25.77**	**-27.62**	**-25.71**	**-25.84**	**-25.79**	**-25.71**	**-25.61**	**-25.61**	**-25.36**	**-25.36**	**-25.42**	**-25.45**	**-27.62**	**-25.44**	**-25.11**	**-25.56**	**-27.63**	**-27.63**	**-27.62**	**-27.62**	**-17.25**	**-17.22**	**-17.26**	**-17.07**	**-17.07**	**-17.17**	**-17.40**	**-17.58**	**-17.68**
Acaena antarctica	.	.	.	.	.	.	.	.	.	.	.	.	.	.	.	.	.	.	.	.	.	.	.	.	.	.	.	.	.
Acaena macrocephala	.	.	.	.	.	.	.	.	.	.	.	.	.	.	.	.	.	.	.	.	.	.	.	.	.	.	.	.	.
Acaena magellanica	.	.	.	.	.	.	.	1	.	.	.	.	.	1	1	.	.	.	.	.	.	.	.	.	.	.	.	.	.
Acaena ovalifolia	.	.	.	.	.	.	.	.	.	.	.	.	.	.	.	.	.	.	.	.	.	.	.	.	.	.	.	.	.
Acaena pinnatifida	.	.	.	.	.	.	.	.	.	.	.	.	.	.	.	.	.	.	.	.	.	.	.	.	.	.	.	.	.
Adesmia retusa	.	.	.	.	.	.	.	.	.	.	.	.	.	.	.	.	.	.	.	.	.	.	.	.	.	.	.	.	.
Agrostis breviculmis	.	.	.	.	.	.	.	.	.	.	.	.	.	.	.	.	.	.	.	.	.	.	.	.	.	.	.	.	.
Agrostis imberbis	.	.	.	.	.	.	.	.	.	.	.	.	.	.	.	.	.	.	.	.	.	.	.	.	.	.	.	.	.
Agrostis meyenii	.	.	.	.	.	.	.	.	.	.	.	.	.	.	.	.	.	.	.	.	.	.	.	.	.	.	.	.	.
Agrostis perennans	.	.	.	.	.	.	.	.	.	.	.	.	.	.	.	.	.	.	.	.	.	.	.	.	.	.	.	.	.
Alchemilla pinnata	.	.	.	.	.	.	.	.	.	.	.	.	.	.	.	.	.	.	.	.	.	.	.	.	.	.	.	.	.
Alopecurus magellanicus	.	.	.	.	.	.	.	.	.	.	.	.	.	.	.	.	.	.	.	.	.	.	.	.	.	.	.	.	.
Amphiscirpus nevadensis	.	.	.	.	.	.	.	1	.	.	.	.	.	.	.	.	.	.	.	.	.	.	.	.	.	.	.	.	.
Anagallis alternifolia	.	.	.	.	.	.	.	.	.	.	.	.	.	.	.	.	.	.	.	.	.	.	.	.	.	.	.	.	.
Antennaria chilensis	.	.	.	.	.	.	.	.	.	.	.	.	.	.	.	.	.	.	.	.	.	.	.	.	.	.	.	.	.
Anthoxanthum redolens	.	.	.	.	.	.	.	.	.	.	.	.	.	.	.	.	.	.	.	.	.	.	.	.	.	.	.	.	.
Apium panul	.	.	.	.	.	.	.	.	.	.	.	.	.	.	.	.	.	.	.	.	.	.	.	.	.	.	.	.	.
Arenaria rivularis	.	.	.	.	.	.	.	.	.	.	.	.	.	.	.	.	.	.	.	.	.	.	.	.	.	.	.	.	.
Arenaria serpens	1	.	.	.	.	1	.	1	.	1	1	.	.	.	.	.	.	.	.	.	.	.	.	.	.	.	.	.	.
Arjona pusilla	.	.	.	.	.	.	.	.	.	.	.	.	.	.	.	.	.	.	.	.	1	1	.	.	.	.	1	.	1
Astragalus bustillosii	.	.	.	.	.	.	.	.	.	.	.	.	.	.	.	.	.	.	.	.	.	.	.	.	.	.	.	.	.
Astragalus micranthellus	.	.	.	.	.	.	.	.	.	.	.	.	.	.	.	.	.	.	.	.	.	.	.	.	.	.	.	.	.
Azolla filiculoides	.	.	.	.	.	.	.	.	.	.	.	.	.	.	.	.	.	.	.	.	.	.	.	.	.	.	.	.	.
Azorella boelckei	.	.	.	.	.	.	.	.	.	.	.	.	.	.	.	.	.	.	.	.	.	.	.	.	.	.	.	.	.
Azorella burkartii	.	.	.	.	.	.	.	.	.	.	.	.	.	.	.	.	.	.	.	.	.	.	.	.	.	.	.	.	.

Bog	59	60	61	62	63	64	65	66	67	68	69	70	71	72	73	74	75	76	77	78	79	80	81	82	83	84	85	86	87
Azorella cryptantha	.	.	.	.	.	.	.	.	.	.	.	.	.	.	.	.	.	.	.	.	.	.	.	.	.	.	.	.	.
Azorella lycopodioides	.	.	.	.	.	.	.	.	.	.	.	.	.	.	.	.	.	.	.	.	.	.	.	.	.	.	.	.	.
Azorella trifoliolata	.	.	.	.	.	.	.	.	.	.	.	.	.	.	.	.	.	.	.	.	.	.	.	.	.	.	.	.	.
Baccharis acaulis	.	.	.	.	.	.	.	1	.	.	.	.	.	.	.	.	.	.	.	.	.	.	.	.	.	.	.	.	.
Baccharis caespitosa	.	.	.	.	.	.	.	.	.	.	.	.	.	.	.	.	.	.	.	.	1	.	1	1	1	.	1	.	.
Baccharis magellanica	.	.	.	.	.	.	.	.	.	.	.	.	.	.	.	.	.	.	.	.	.	.	.	.	.	.	.	.	.
Belloa chilensis	.	.	.	.	.	.	.	.	.	.	.	.	.	.	.	.	.	.	.	.	.	.	.	.	.	.	.	.	.
Bromus catharticus	.	.	.	.	.	.	.	.	.	.	.	.	.	.	.	.	.	.	.	.	.	.	.	.	.	.	.	.	.
Calandrinia acaulis	.	.	.	.	.	.	.	.	.	.	.	.	.	.	.	.	.	.	.	.	.	.	.	.	.	.	.	.	.
Calandrinia compacta	.	.	.	.	.	.	.	.	.	.	.	.	.	.	.	.	.	.	.	.	.	.	.	.	.	.	.	1	.
Calceolaria biflora	.	.	.	.	.	.	.	.	.	.	.	.	.	.	.	.	.	.	.	.	.	.	.	.	.	.	.	.	.
Calceolaria cana	.	.	.	.	.	.	.	.	.	.	.	.	.	.	.	.	.	.	.	.	.	.	.	.	.	.	.	.	.
Calceolaria corymbosa	.	.	.	.	.	.	.	.	.	.	.	.	.	.	.	.	.	.	.	.	.	.	.	.	.	.	.	.	.
Calceolaria filicaulis	.	.	.	.	.	.	.	.	.	.	.	.	.	.	.	.	.	.	.	.	.	.	.	.	.	.	.	.	.
Callitriche lechleri	.	.	.	.	.	.	.	.	.	.	.	.	.	.	.	.	.	.	.	.	.	.	.	.	.	.	.	.	.
Caltha appendiculata	.	.	.	.	.	.	.	.	.	.	.	.	.	.	.	.	.	.	.	.	.	.	.	.	.	.	.	.	.
Caltha sagittata	.	.	.	.	.	.	.	.	.	.	.	.	.	.	.	.	.	.	.	.	.	.	.	.	.	.	1	.	.
Cardamine cordata	.	.	.	.	.	.	.	.	.	.	.	.	.	.	.	.	.	.	.	.	.	.	.	.	.	.	.	.	.
Cardamine glacialis	.	.	.	.	.	.	.	.	.	.	.	.	.	.	.	.	.	.	.	.	.	.	.	.	.	.	.	.	.
Cardamine tenuirostris	.	.	.	.	.	.	.	.	.	.	.	.	.	.	.	.	.	.	.	.	.	.	.	.	.	.	.	.	.
Cardamine volckmannii	.	.	.	.	.	.	.	.	.	.	.	.	.	.	.	.	.	.	.	.	.	.	.	.	.	.	.	.	.
Carex acaulis	.	.	.	.	.	.	.	.	.	.	.	.	.	.	.	.	.	.	.	.	.	.	.	.	.	.	.	.	.
Carex atropicta	.	.	.	.	.	.	.	.	.	.	.	.	.	.	.	.	.	.	.	.	.	.	.	.	.	.	.	.	.
Carex banksii	.	.	.	.	.	.	.	.	.	.	.	.	.	.	.	.	.	.	.	.	.	.	.	.	.	.	.	.	.
Carex caduca	.	.	.	.	.	.	.	.	.	.	.	.	.	.	.	.	.	.	.	.	.	.	.	.	.	.	.	.	.
Carex decidua	.	.	.	.	.	.	.	.	.	.	.	.	.	.	.	.	.	.	.	.	.	.	.	.	.	.	.	.	.
Carex fuscula	.	.	.	.	.	.	.	.	.	.	.	.	.	.	.	.	.	.	.	.	.	.	.	.	.	.	.	.	.
Carex gayana	.	.	.	.	.	1	1	1	1	1	1	1	1	1	.	1	.	.	.	1	.	.	.	.	.	.	.	.	.
Carex hypoleucos	.	.	.	.	.	.	.	.	.	.	.	.	.	.	.	.	.	.	.	.	.	.	.	.	.	.	.	.	.
Carex macloviana	.	.	.	.	.	.	.	.	.	.	.	.	.	.	.	.	.	.	.	.	.	.	.	.	.	.	.	.	.
Carex magellanica	.	.	.	.	.	.	.	.	.	.	.	.	.	.	.	.	.	.	.	.	.	.	.	.	.	.	.	.	.
Carex malmei	.	.	.	.	.	.	.	.	.	.	.	.	.	.	.	.	.	.	.	.	.	.	.	.	.	.	.	.	.
Carex maritima	1	.	1	1	1	.	.	1	.	.	.	.	.	.	.	1	1	.	.	.	1	.	1	.	.	1	1	1	.
Carex microglochin	.	.	.	.	.	.	.	.	.	.	.	.	.	.	.	.	.	.	.	1	1	1	1	.	.	.	.	.	.
Carex pleioneura	.	.	.	.	.	.	.	.	.	.	.	.	.	.	.	.	.	.	.	.	.	.	.	.	.	.	.	.	.
Carex ruthsatzae	.	.	.	.	.	.	.	.	.	.	.	.	.	.	.	.	.	.	.	.	.	.	.	.	.	.	.	.	.
Carex vallis-pulchrae	1	.	.	.	.	.	.	.	.	.	.	.	.	.	.	.	.	.	.	.	.	.	.	.	.	.	.	.	.
Carpha schoenoides	.	.	.	.	.	.	.	.	.	.	.	.	.	.	.	.	.	.	.	.	.	.	.	.	.	.	.	.	.

Bog	59	60	61	62	63	64	65	66	67	68	69	70	71	72	73	74	75	76	77	78	79	80	81	82	83	84	85	86	87
Castilleja pumila	.	.	.	.	.	.	.	.	.	.	.	.	.	.	.	.	.	.	.	.	1	1	1	1	1	.	1	1	1
Catabrosa werdermannii	1	.	.	1	1	.	.	1	.	.	.	.	.	.	.	.	.	.	.	.	.	.	.	.	.	.	.	.	.
Cerastium humifusum	.	.	.	.	.	.	.	.	.	.	.	.	.	.	.	.	.	.	.	.	.	.	.	.	.	.	.	.	.
Cerastium montioides	.	.	.	.	.	.	.	.	.	.	.	.	.	.	.	.	.	.	.	.	.	.	.	.	.	.	.	.	.
Chiliotrichum diffusum	.	.	.	.	.	.	.	.	.	.	.	.	.	.	.	.	.	.	.	.	.	.	.	.	.	.	.	.	.
Chusquea culeou	.	.	.	.	.	.	.	.	.	.	.	.	.	.	.	.	.	.	.	.	.	.	.	.	.	.	.	.	.
Colobanthus quitensis	1	.	.	1	.	1	.	1	.	.	.	.	.	.	.	.	1	.	.	.	1	.	.	.	.	.	1	1	1
Cortaderia egmontiana	.	.	.	.	.	.	.	.	.	.	.	.	.	.	.	.	.	.	.	.	.	.	.	.	.	.	.	.	.
Cotula mexicana	.	.	.	.	.	.	.	1	.	.	.	.	.	.	.	.	.	.	.	.	.	1	1	1	1	.	1	1	1
Crassula peduncularis	.	.	.	.	.	.	.	.	.	.	.	.	.	.	.	.	.	.	.	.	.	.	.	.	.	.	.	.	.
Cuatrecasasiella argentina	.	.	.	.	.	.	.	.	.	.	.	.	.	.	.	.	.	.	.	.	1	1	1	1	1	.	1	1	.
Deschampsia antarctica	.	.	.	.	.	.	.	.	.	.	.	.	.	.	.	.	.	.	.	.	.	.	.	.	.	.	.	.	.
Deschampsia caespitosa	.	.	.	.	.	.	.	.	.	.	.	.	.	.	.	.	.	.	.	.	.	.	.	.	.	.	.	.	.
Deschampsia patula	.	.	.	.	.	.	.	.	.	.	.	.	.	.	.	.	.	.	.	.	.	.	.	.	.	.	.	.	.
Cinnagrostis brevifolia	.	.	.	.	.	.	.	.	.	.	.	.	.	.	.	.	.	.	.	.	.	.	.	.	.	.	.	.	.
Deschampsia chrysantha	.	.	.	.	.	.	.	.	.	.	.	.	.	.	.	.	.	.	.	.	.	.	.	.	.	.	.	.	.
Cinnagrostis chrysophylla	.	.	.	.	.	.	.	.	.	.	.	.	.	.	.	.	.	.	.	.	.	.	.	.	.	.	.	1	.
Deschampsia chrysostachya	.	.	.	.	.	.	.	.	.	.	.	.	.	.	.	.	.	.	.	.	.	.	.	.	.	.	.	.	.
Deschampsia eminens	.	.	.	.	.	.	.	1	.	.	1	1	1	.	.	1	1	1	.	1	.	.	.	.	.	.	.	.	.
Deschampsia hackelii	1	.	1	.	.	1	.	.	1	1	.	.	.	.	.	.	.	.	.	.	.	.	.	.	.	.	.	.	.
Cinnagrostis minima	.	.	.	.	.	.	.	.	.	.	.	.	.	.	.	.	.	.	.	.	.	.	.	.	.	.	.	.	.
Deschampsia ovata	.	.	.	.	.	.	.	.	.	.	.	.	.	.	.	.	.	.	.	.	.	.	.	.	.	.	.	1	.
Cinnagrostis rigescens	.	.	.	.	.	.	.	.	.	.	.	.	.	.	.	.	.	.	.	.	1	1	1	1	.	1	1	1	1
Cinnagrostis spicigera	.	.	.	.	.	.	.	.	.	.	.	.	.	.	.	.	.	.	.	.	.	.	.	.	.	.	.	.	.
Cinnagrostis velutina	.	.	.	.	.	.	.	.	.	.	.	.	.	.	.	.	.	.	.	.	.	.	.	.	.	.	.	.	.
Cinnagrostis vicunarum	.	.	.	.	.	.	.	.	.	.	.	.	.	.	.	.	.	.	.	.	.	.	.	.	.	.	.	.	.
Distichia filamentosa	.	.	.	.	.	.	.	.	.	.	.	.	.	.	.	.	.	.	.	.	.	.	.	.	.	.	.	.	.
Distichia muscoides	.	.	.	.	.	.	.	.	.	.	.	.	.	.	.	.	.	.	.	.	1	1	1	1	1	1	1	1	1
Distichlis humilis	.	.	.	1	1	.	.	1	.	.	1	.	.	.	.	.	.	.	.	.	.	.	.	.	.	.	.	.	.
Distichlis scoparia	.	.	.	.	.	.	.	.	.	.	.	.	.	.	.	.	.	.	.	.	.	.	.	.	.	.	.	.	.
Distichlis spicata	.	.	.	.	.	.	.	.	.	.	.	.	.	.	.	.	.	.	.	.	.	.	.	.	.	.	.	.	.
Draba pusilla	.	.	.	.	.	.	.	.	.	.	.	.	.	.	.	.	.	.	.	.	.	.	.	.	.	.	.	.	.

Bog	59	60	61	62	63	64	65	66	67	68	69	70	71	72	73	74	75	76	77	78	79	80	81	82	83	84	85	86	87
Eleocharis melanomphala	.	.	.	.	.	.	.	1	.	.	.	.	1	.	.	.	1	.	1	.	.	.	.	.	.	.	.	.	.
Eleocharis pseudoalbibracteata	1	1	.	1	1	1	1	1	.	.	.	.	.	.	1	.	1	1	1	1	.	.	.	.	.	.	.	.	.
Elodea potamogeton	.	.	.	.	.	.	.	.	.	.	.	.	.	.	.	.	.	.	.	.	.	.	.	.	.	.	.	.	.
Empetrum rubrum	.	.	.	.	.	.	.	.	.	.	.	.	.	.	.	.	.	.	.	.	.	.	.	.	.	.	.	.	.
Epilobium australe	.	.	.	.	.	.	.	.	.	.	.	.	.	.	.	.	.	.	.	.	.	.	.	.	.	.	.	.	.
Epilobium barbeyanum	.	.	.	.	.	.	.	.	.	.	.	.	.	.	.	.	.	.	.	.	.	.	.	.	.	.	.	.	.
Epilobium ciliatum	.	.	.	.	.	.	.	.	.	.	.	.	.	.	.	.	.	.	.	.	.	.	.	.	.	.	.	.	.
Epilobium denticulatum	.	.	.	.	.	.	.	.	.	.	.	.	.	.	.	.	.	.	.	.	.	.	.	.	.	.	.	.	.
Epilobium fragile	.	.	.	.	.	.	.	.	.	.	.	.	.	.	.	.	.	.	.	.	.	.	.	.	.	.	.	.	.
Epilobium glaucum	.	.	.	.	.	.	.	.	.	.	.	.	.	.	.	.	.	.	.	.	.	.	.	.	.	.	.	.	.
Epilobium nivale	.	.	.	.	.	.	.	.	.	.	.	.	.	.	.	.	.	.	.	.	.	.	.	.	.	.	.	.	.
Erigeron andicola	.	.	.	.	.	.	.	.	.	.	.	.	.	.	.	.	.	.	.	.	.	.	.	.	.	.	.	.	.
Erigeron leptopetalus	.	.	.	.	.	.	.	.	.	.	.	.	.	.	.	.	.	.	.	.	.	.	.	.	.	.	.	.	.
Erigeron myosotis	.	.	.	.	.	.	.	.	.	.	.	.	.	.	.	.	.	.	.	.	.	.	.	.	.	.	.	.	.
Erigeron patagonicus	.	.	.	.	.	.	.	.	.	.	.	.	.	.	.	.	.	.	.	.	.	.	.	.	.	.	.	.	.
Erythranthe cuprea	.	.	.	.	.	.	.	.	.	.	.	.	.	.	.	.	.	.	.	.	.	.	.	.	.	.	.	.	.
Erythranthe depressa	.	.	.	.	.	1	.	1	.	.	.	.	.	.	.	.	.	1	1	.	.	.	.	.	.	.	.	.	.
Erythranthe glabrata	.	.	.	.	.	.	.	.	.	.	.	.	.	.	.	.	.	.	.	.	.	.	.	.	.	.	.	.	.
Erythranthe lutea	.	.	.	.	.	.	.	.	.	.	.	.	.	.	.	.	.	.	.	.	.	.	.	.	.	.	.	.	.
Escallonia virgata	.	.	.	.	.	.	.	.	.	.	.	.	.	.	.	.	.	.	.	.	.	.	.	.	.	.	.	.	.
Euphrasia antarctica	.	.	.	.	.	.	.	.	.	.	.	.	.	.	.	.	.	.	.	.	.	.	.	.	.	.	.	.	.
Euphrasia chrysantha	.	.	.	.	.	.	.	.	.	.	.	.	.	.	.	.	.	.	.	.	.	.	.	.	.	.	.	.	.
Euphrasia subexserta	.	.	.	.	.	.	.	.	.	.	.	.	.	.	.	.	.	.	.	.	.	.	.	.	.	.	.	.	.
Festuca hypsophila	.	.	.	.	.	.	.	.	.	.	.	.	.	.	.	.	.	.	.	.	.	.	.	.	.	.	.	.	.
Festuca kurtziana	.	.	.	.	.	.	.	.	.	.	.	.	.	.	.	.	.	.	.	.	.	.	.	.	.	.	.	.	.
Festuca lilloi	.	.	.	.	.	.	.	.	.	.	.	.	.	.	.	.	.	.	.	.	.	.	.	.	.	.	.	.	.
Festuca magellanica	.	.	.	.	.	.	.	.	.	.	.	.	.	.	.	.	.	.	.	.	.	.	.	.	.	.	.	.	.
Festuca nardifolia	.	.	.	.	.	.	.	.	.	.	.	.	.	.	.	.	.	.	.	.	.	.	.	.	.	.	.	.	.
Festuca rigescens	.	.	.	.	.	.	.	.	.	.	.	.	.	.	.	.	.	.	.	.	.	.	.	.	.	.	.	.	.
Festuca werdermannii	.	.	.	.	.	.	.	.	.	.	.	.	.	.	.	.	.	.	.	.	.	.	.	.	.	.	.	.	.
Frankenia triandra	.	.	.	.	.	.	.	.	.	.	.	.	.	.	.	.	.	.	.	.	.	.	.	.	.	.	.	.	.
Gamocarpha graminea	.	.	.	.	.	.	.	.	.	.	.	.	.	.	.	.	.	.	.	.	.	.	.	.	.	.	.	.	.
Gamocarpha ventosa	.	.	.	.	.	.	.	.	.	.	.	.	.	.	.	.	.	.	.	.	.	.	.	.	.	.	.	.	.
Gamochaeta chamissonis	.	.	.	.	.	.	.	.	.	.	.	.	.	.	.	.	.	.	.	.	.	.	.	.	.	.	.	.	.

**Bog**	**59**	**60**	**61**	**62**	**63**	**64**	**65**	**66**	**67**	**68**	**69**	**70**	**71**	**72**	**73**	**74**	**75**	**76**	**77**	**78**	**79**	**80**	**81**	**82**	**83**	**84**	**85**	**86**	**87**
Gamochaeta longipedicellata	.	.	.	.	.	.	.	.	.	.	.	.	.	.	.	.	.	.	.	.	.	.	.	.	.	.	.	.	.
Gamochaeta neuquensis	.	.	.	.	.	.	.	.	.	.	.	.	.	.	.	.	.	.	.	.	.	.	.	.	.	.	.	.	.
Gaultheria antarctica	.	.	.	.	.	.	.	.	.	.	.	.	.	.	.	.	.	.	.	.	.	.	.	.	.	.	.	.	.
Gaultheria caespitosa	.	.	.	.	.	.	.	.	.	.	.	.	.	.	.	.	.	.	.	.	.	.	.	.	.	.	.	.	.
Gaultheria pumila	.	.	.	.	.	.	.	.	.	.	.	.	.	.	.	.	.	.	.	.	.	.	.	.	.	.	.	.	.
Gavilea chica	.	.	.	.	.	.	.	.	.	.	.	.	.	.	.	.	.	.	.	.	.	.	.	.	.	.	.	.	.
Gentiana prostrata	1	1	.	.	1	.	.	1	.	.	1	1	1	1	1	1	1	1	1	1	1	1	1	1	1	1	1	1	1
Gentianella fiebrigii	.	.	.	.	.	.	.	.	.	.	.	.	.	.	.	.	.	.	.	.	.	.	.	.	.	.	.	.	.
Gentianella magellanica	.	.	.	.	.	.	.	.	.	.	.	.	.	.	.	.	.	.	.	.	.	.	.	.	.	.	.	.	.
Gentianella multicaulis	.	.	.	.	.	.	.	.	.	.	.	.	.	.	.	.	.	.	.	.	.	.	.	.	.	.	.	.	.
Gentianella ottonis	.	.	.	.	.	.	.	.	.	.	.	.	.	.	.	.	.	.	.	.	.	.	.	.	.	.	.	.	.
Gentianella primuloides	.	.	.	.	.	.	.	.	.	.	.	.	.	.	.	.	.	.	.	.	1	1	1	1	.	1	1	1	1
Gentianella pseudocrassula	.	.	.	.	.	.	.	.	.	.	.	.	.	.	.	.	.	.	.	.	.	.	.	.	.	.	.	.	.
Geranium sessiliflorum	.	.	.	.	.	.	.	.	.	.	.	.	.	.	.	.	.	.	.	.	.	.	.	.	.	.	.	.	.
Gunnera magellanica	.	.	.	.	.	.	.	.	.	.	.	.	.	.	.	.	.	.	.	.	.	.	.	.	.	.	.	.	.
Halenia caespitosa	.	.	.	.	.	.	.	.	.	.	.	.	.	.	.	.	.	.	.	.	1	1	1	.	.	.	.	.	.
Halerpestes cymbalaria	.	.	.	1	1	.	1	1	.	.	.	.	.	.	.	.	.	.	.	.	.	.	.	.	.	.	.	.	.
Halerpestes exilis	1	.	1	.	.	1	1	1	.	1	1	.	.	1	.	.	.	.	.	.	.	.	.	.	.	.	.	.	.
Hieracium antarcticum	.	.	.	.	.	.	.	.	.	.	.	.	.	.	.	.	.	.	.	.	.	.	.	.	.	.	.	.	.
Hordeum comosum	.	.	.	.	.	.	.	.	.	.	.	.	.	.	.	.	.	.	.	.	.	.	.	.	.	.	.	.	.
Hordeum muticum	.	.	.	1	1	.	.	1	.	.	.	1	.	1	1	.	1	.	1	1	.	.	.	.	.	.	.	.	.
Hypochaeris acaulis	.	.	.	.	.	.	.	.	.	.	.	.	.	.	.	.	.	.	.	.	.	.	.	.	.	.	.	.	.
Hypochaeris chondrilloides	.	.	.	.	.	.	.	1	.	.	.	.	.	.	.	.	.	.	.	.	.	.	.	.	.	.	.	.	.
Hypochaeris meyeniana	.	.	.	.	.	.	.	1	.	.	.	.	.	.	1	1	.	.	.	.	.	.	.	.	.	.	.	.	.
Hypochaeris palustris	.	.	.	.	.	.	.	.	.	.	.	.	.	.	.	.	.	.	.	.	.	.	.	.	.	.	.	.	.
Hypochaeris taraxacoides	.	.	.	.	.	.	.	.	.	.	.	.	.	.	.	.	.	.	.	.	1	1	1	1	1	1	1	1	1
Hypochaeris tenerifolia	.	.	.	.	.	.	.	.	.	.	.	.	.	.	.	.	.	.	.	.	.	.	.	.	.	.	.	.	.
Isolepis nigricans	.	.	.	.	.	.	.	.	.	.	.	.	.	.	.	.	.	.	.	.	.	.	.	.	.	.	.	.	.
Isolepsis inundata	.	.	.	.	.	.	.	.	.	.	.	.	.	.	.	.	.	.	.	.	.	.	.	.	.	.	.	.	.
Juncus balticus	.	1	.	1	1	.	.	1	.	.	.	.	1	1	1	.	1	1	1	1	.	.	.	.	.	.	.	.	.
Juncus stipulatus	.	.	.	.	.	1	.	1	.	.	.	.	.	.	.	.	1	.	1	1	1	1	1	1	1	1	1	1	1
Koeleria kurtzii	.	.	.	.	.	.	.	.	.	.	.	.	.	.	.	.	.	.	.	.	.	.	.	.	.	.	.	.	.

Bog	59	60	61	62	63	64	65	66	67	68	69	70	71	72	73	74	75	76	77	78	79	80	81	82	83	84	85	86	87
Lachemilla diplophylla	.	.	.	.	.	.	.	.	.	.	.	.	.	.	.	.	.	.	.	.	1	1	1	1	.	1	1	1	1
Lachemilla pinnata	.	.	.	.	.	.	.	.	.	.	.	.	.	.	.	.	.	.	.	.	1	1	1	1	1	1	1	1	1
Lagenophora nudicaulis	.	.	.	.	.	.	.	.	.	.	.	.	.	.	.	.	.	.	.	.	.	.	.	.	.	.	.	.	.
Lemna minuta	.	.	.	.	.	1	.	1	.	.	1	.	.	.	.	.	.	.	.	.	.	.	.	.	.	.	.	.	.
Leptinella scariosa	.	.	.	.	.	.	.	.	.	.	.	.	.	.	.	.	.	.	.	.	.	.	.	.	.	.	.	.	.
Leucheria candidissima	.	.	.	.	.	.	.	.	.	.	.	.	.	.	.	.	.	.	.	.	.	.	.	.	.	.	.	.	.
Leucheria nutans	.	.	.	.	.	.	.	.	.	.	.	.	.	.	.	.	.	.	.	.	.	.	.	.	.	.	.	.	.
Lilaea scilloides	.	.	.	.	.	.	.	.	.	.	.	.	.	.	.	.	.	.	.	.	.	.	.	.	.	.	.	.	.
Lilaeopsis macloviana	.	.	1	1	1	1	1	.	.	1	.	.	.	1	.	.	.	.	.	.	.	1	.	1	.	.	.	.	1
Limosella australis	.	.	.	.	.	.	.	.	.	.	.	.	.	.	.	.	.	.	.	.	.	.	.	.	.	.	.	.	.
Lobelia oligophylla	1	.	.	1	1	1	1	1	.	.	1	1	1	.	1	.	1	1	1	.	.	.	.	.	.	.	1	1	1
Luzula brachyphylla	.	.	.	.	.	.	.	.	.	.	.	.	.	.	.	.	.	.	.	.	.	.	.	.	.	.	.	.	.
Luzula chilensis	.	.	.	.	.	.	.	.	.	.	.	.	.	.	.	.	.	.	.	.	.	.	.	.	.	.	.	.	.
Luzula racemosa	.	.	.	.	.	.	.	.	.	.	.	.	.	.	.	.	.	.	.	.	.	.	.	.	.	.	.	.	.
Luzula vulcanica	.	.	.	.	.	.	.	.	.	.	.	.	.	.	.	.	.	.	.	.	.	.	.	.	.	.	.	.	.
Lysipomia pumila	.	.	.	.	.	.	.	.	.	.	.	.	.	.	.	.	.	.	.	.	.	1	.	.	.	1	.	.	.
Marsippospermum philippii	.	.	.	.	.	.	.	.	.	.	.	.	.	.	.	.	.	.	.	.	.	.	.	.	.	.	.	.	.
Marsippospermum reichei	.	.	.	.	.	.	.	.	.	.	.	.	.	.	.	.	.	.	.	.	.	.	.	.	.	.	.	.	.
Montia fontana	.	.	.	.	.	.	.	.	.	.	.	.	.	.	.	.	.	.	.	.	.	.	.	.	.	1	.	.	.
Muhlenbergia asperifolia	.	.	.	.	.	.	.	.	.	.	.	.	.	.	.	.	.	.	.	.	.	.	.	.	.	.	.	.	.
Myriophyllum quitense	.	.	.	1	1	.	.	1	.	.	1	1	.	.	.	.	.	.	.	.	.	.	.	.	.	.	.	.	.
Myrosmodes nervosa	.	.	.	.	.	.	.	.	.	.	.	.	.	.	.	.	.	.	.	.	.	.	.	.	.	.	.	.	.
Myrosmodes paludosa	.	1	.	.	.	.	.	.	.	.	.	.	.	.	.	.	.	1	.	.	.	.	.	.	.	.	.	.	.
Myrteola nummularia	.	.	.	.	.	.	.	.	.	.	.	.	.	.	.	.	.	.	.	.	.	.	.	.	.	.	.	.	.
Nanodea muscosa	.	.	.	.	.	.	.	.	.	.	.	.	.	.	.	.	.	.	.	.	.	.	.	.	.	.	.	.	.
Neobartsia crenoloba	.	.	.	.	.	.	.	.	.	.	.	.	.	.	.	.	.	.	.	.	.	.	.	.	.	.	.	.	.
Neobartsia pedicularoides	.	.	.	.	.	.	.	.	.	.	.	.	.	.	.	.	.	.	.	.	.	1	.	.	.	.	.	.	.
Neobartsia peruviana	.	.	.	.	.	.	.	.	.	.	.	.	.	.	.	.	.	.	.	.	.	.	.	.	.	.	.	.	.
Nertera granadensis	.	.	.	.	.	.	.	.	.	.	.	.	.	.	.	.	.	.	.	.	.	.	.	.	.	.	.	.	.
Nicoraepoa andina	.	.	.	.	.	.	.	.	.	.	.	.	.	.	.	.	.	.	.	.	.	.	.	.	.	.	.	.	.
Nicoraepoa pugionifolia	.	.	.	.	.	.	.	.	.	.	.	.	.	.	.	.	.	.	.	.	.	.	.	.	.	.	.	.	.
Nicoraepoa subenervis	.	.	.	.	.	.	.	.	.	.	.	.	.	.	.	.	.	.	.	.	.	.	.	.	.	.	.	.	.

**Bog**	**59**	**60**	**61**	**62**	**63**	**64**	**65**	**66**	**67**	**68**	**69**	**70**	**71**	**72**	**73**	**74**	**75**	**76**	**77**	**78**	**79**	**80**	**81**	**82**	**83**	**84**	**85**	**86**	**87**
Nitrophila australis	.	.	.	1	1	.	.	1	.	.	.	.	.	.	1	.	.	.	.	.	.	.	.	.	.	.	.	.	.
Nothofagus antarctica	.	.	.	.	.	.	.	.	.	.	.	.	.	.	.	.	.	.	.	.	.	.	.	.	.	.	.	.	.
Nototriche rugosa	.	.	.	.	.	.	.	.	.	.	.	.	.	.	.	.	.	.	.	.	.	.	.	.	.	.	.	.	.
Ochetophila nana	.	.	.	.	.	.	.	.	.	.	.	.	.	.	.	.	.	.	.	.	.	.	.	.	.	.	.	.	.
Olsynium junceum	.	.	.	.	.	.	.	.	.	.	.	.	.	.	.	.	.	.	.	.	.	.	.	.	.	.	.	.	.
Oreobolus obtusangulus	.	.	.	.	.	.	.	.	.	.	.	.	.	.	.	.	.	.	.	.	.	.	.	.	.	.	.	.	.
Oritrophium limnophilum	.	.	.	.	.	.	.	.	.	.	.	.	.	.	.	.	.	.	.	.	1	1	1	1	1	.	.	.	.
Osmorhiza glabrata	.	.	.	.	.	.	.	.	.	.	.	.	.	.	.	.	.	.	.	.	.	.	.	.	.	.	.	.	.
Ourisia alpina	.	.	.	.	.	.	.	.	.	.	.	.	.	.	.	.	.	.	.	.	.	.	.	.	.	.	.	.	.
Ourisia muscosa	.	.	.	.	.	.	.	.	.	.	.	.	.	.	.	.	.	.	.	.	1	.	.	.	.	1	.	1	.
Ourisia ruelloides	.	.	.	.	.	.	.	.	.	.	.	.	.	.	.	.	.	.	.	.	.	.	.	.	.	.	.	.	.
Oxychloe andina	1	1	.	.	.	1	.	1	1	1	1	1	1	.	.	.	1	1	1	1	.	.	.	.	.	.	.	.	.
Oxychloe bisexualis	.	.	.	.	.	.	.	.	.	.	.	.	.	.	.	.	.	.	.	.	.	.	.	.	.	.	.	.	.
Oxychloe castellanosii	.	.	.	.	.	.	.	.	.	.	.	.	.	.	.	.	.	.	.	.	.	.	.	.	.	.	.	.	.
Oxychloe haumaniana	.	.	.	.	.	.	.	.	.	.	.	.	.	.	.	.	.	.	.	.	.	.	.	.	.	.	.	.	.
Oxychloe mendocina	.	.	.	.	.	.	.	.	.	.	.	.	.	.	.	.	.	.	.	.	.	.	.	.	.	.	.	.	.
Patosia clandestina	.	.	.	.	.	.	.	.	.	.	.	.	.	.	.	.	.	.	.	.	.	.	.	.	.	.	.	.	.
Perezia capito	.	.	.	.	.	.	.	.	.	.	.	.	.	.	.	.	.	.	.	.	.	.	.	.	.	.	.	.	.
Perezia delicata	.	.	.	.	.	.	.	.	.	.	.	.	.	.	.	.	.	.	.	.	.	.	.	.	.	.	.	.	.
Perezia fonkii	.	.	.	.	.	.	.	.	.	.	.	.	.	.	.	.	.	.	.	.	.	.	.	.	.	.	.	.	.
Perezia pedicularidifolia	.	.	.	.	.	.	.	.	.	.	.	.	.	.	.	.	.	.	.	.	.	.	.	.	.	.	.	.	.
Perezia pinnatifida	.	.	.	.	.	.	.	.	.	.	.	.	.	.	.	.	.	.	.	.	.	.	1	.	.	1	.	.	.
Petroravenia friesii	.	.	.	.	.	.	.	.	.	.	.	.	.	.	.	.	.	.	.	.	.	.	.	.	.	.	.	.	.
Petroravenia werdermannii	.	.	.	.	.	.	.	.	.	.	.	.	.	.	.	.	.	.	.	.	.	.	.	.	.	.	.	.	.
Phleum alpinum	.	.	.	.	.	.	.	.	.	.	.	.	.	.	.	.	.	.	.	.	.	.	.	.	.	.	.	.	.
Phylloscirpus acaulis	.	1	.	1	.	.	.	1	.	.	1	1	1	.	.	.	1	1	1	1	.	.	.	.	.	.	.	.	.
Phylloscirpus boliviensis	.	.	.	.	.	.	.	.	.	.	.	.	.	.	.	.	.	.	.	.	.	.	.	.	.	.	.	.	.
Phylloscirpus deserticola	.	.	.	.	.	.	.	.	.	.	.	.	1	.	.	.	.	.	.	1	1	1	1	1	1	.	1	1	1
Pinguicula antarctica	.	.	.	.	.	.	.	.	.	.	.	.	.	.	.	.	.	.	.	.	.	.	.	.	.	.	.	.	.
Plantago barbata	.	.	.	.	.	.	.	.	.	.	.	.	.	.	.	.	.	.	.	.	.	.	.	.	.	.	.	.	.
Plantago rigida	.	.	.	.	.	.	.	.	.	.	.	.	.	.	.	.	.	.	.	.	1	1	1	1	1	.	.	.	.
Plantago tubulosa	.	.	.	.	.	.	.	.	.	.	.	.	.	.	.	.	.	.	.	.	1	1	1	.	1	1	1	1	1

Bog	59	60	61	62	63	64	65	66	67	68	69	70	71	72	73	74	75	76	77	78	79	80	81	82	83	84	85	86	87
Plantago uniglumis	.	.	.	.	.	.	.	.	.	.	.	.	.	.	.	.	.	.	.	.	.	.	.	.	.	.	.	.	.
Poa alopecurus	.	.	.	.	.	.	.	.	.	.	.	.	.	.	.	.	.	.	.	.	.	.	.	.	.	.	.	.	.
Poa hachadoensis	.	.	.	.	.	.	.	.	.	.	.	.	.	.	.	.	.	.	.	.	.	.	.	.	.	.	.	.	.
Poa perligulata	.	.	.	.	.	.	.	.	.	.	.	.	.	.	.	.	.	.	.	.	.	.	.	.	.	1	.	.	.
Polypogon interruptus	.	.	.	.	.	.	.	.	.	.	.	.	.	.	.	.	.	.	.	.	.	.	.	.	.	.	.	.	.
Primula magellanica	.	.	.	.	.	.	.	.	.	.	.	.	.	.	.	.	.	.	.	.	.	.	.	.	.	.	.	.	.
Puccinellia frigida	.	.	1	1	1	1	1	1	1	1	1	.	.	.	1	1	.	.	.	.	.	.	.	.	.	.	.	.	.
Quinchamalium chilense	.	.	.	.	.	.	.	.	.	.	.	.	.	.	.	.	.	.	.	.	.	.	.	.	.	.	.	.	.
Ranunculus breviscapus	.	.	.	.	.	.	.	.	.	.	.	.	.	.	.	.	.	.	.	.	.	.	.	.	.	.	.	.	.
Ranunculus fuegianus	.	.	.	.	.	1	.	.	.	.	.	.	.	.	.	.	.	.	.	.	.	.	.	.	.	.	.	.	.
Ranunculus mandonianus	.	.	.	.	.	.	.	.	.	.	.	.	.	.	.	.	.	.	.	.	.	.	.	.	.	.	.	.	.
Ranunculus peduncularis	.	.	.	.	.	.	.	.	.	.	.	.	.	.	.	.	.	.	.	.	.	.	.	.	.	.	.	.	.
Ranunculus trichophyllus	.	.	.	.	.	.	.	.	.	.	.	.	.	.	.	.	.	.	.	.	.	.	.	.	.	.	.	.	.
Halerpestes uniflora	.	.	.	.	.	.	.	.	.	.	.	.	.	.	.	.	.	.	1	.	.	.	.	.	.	.	.	.	.
Rubus geoides	.	.	.	.	.	.	.	.	.	.	.	.	.	.	.	.	.	.	.	.	.	.	.	.	.	.	.	.	.
Rumex magellanicus	.	.	.	.	.	.	.	.	.	.	.	.	.	.	.	.	.	.	.	.	.	.	.	.	.	.	.	.	.
Rytidosperma lechleri	.	.	.	.	.	.	.	.	.	.	.	.	.	.	.	.	.	.	.	.	.	.	.	.	.	.	.	.	.
Sarcocornia pulvinata	.	.	.	.	.	.	.	.	.	.	.	.	.	.	.	.	.	.	.	.	.	.	.	.	.	.	.	.	.
Schoenoplectus pungens	.	.	.	.	.	.	.	.	.	.	.	.	.	.	.	.	.	.	.	.	.	.	.	.	.	.	.	.	.
Schoenus andinus	.	.	.	.	.	.	.	.	.	.	.	.	.	.	.	.	.	.	.	.	.	.	.	.	.	.	.	.	.
Senecio breviscapus	.	.	.	.	.	.	.	.	.	.	.	.	.	.	.	.	.	.	.	.	.	.	.	.	.	.	.	.	.
Senecio diemii	.	.	.	.	.	.	.	.	.	.	.	.	.	.	.	.	.	.	.	.	.	.	.	.	.	.	.	.	.
Senecio fistulosus	.	.	.	.	.	.	.	.	.	.	.	.	.	.	.	.	.	.	.	.	.	.	.	.	.	.	.	.	.
Senecio parodii	.	.	.	.	.	.	.	.	.	.	.	.	.	.	.	.	.	.	.	.	.	.	.	.	.	.	.	.	.
Senecio peteroanus	.	.	.	.	.	.	.	.	.	.	.	.	.	.	.	.	.	.	.	.	.	.	.	.	.	.	.	.	.
Senecio serratifolius	.	.	.	.	.	.	.	.	.	.	.	.	.	.	.	.	.	.	.	.	.	1	.	.	.	1	.	.	.
Senecio trifurcatus	.	.	.	.	.	.	.	.	.	.	.	.	.	.	.	.	.	.	.	.	.	.	.	.	.	.	.	.	.
Sisyrinchium chilense	.	.	.	.	.	.	.	.	.	.	.	.	.	.	.	.	.	.	.	.	.	.	.	.	.	.	.	.	.
Sisyrinchium patagonicum	.	.	.	.	.	.	.	.	.	.	.	.	.	.	.	.	.	.	.	.	.	.	.	.	.	.	.	.	.
Sisyrinchium pearcei	.	.	.	.	.	.	.	.	.	.	.	.	.	.	.	.	.	.	.	.	.	.	.	.	.	.	.	.	.
Stellaria debilis	.	.	.	.	.	.	.	.	.	.	.	.	.	.	.	.	.	.	.	.	.	.	.	.	.	.	.	.	.
Stuckenia filiformis	1	.	1	1	1	1	1	1	1	1	1	.	.	1	1	.	.	.	.	.	.	.	.	.	.	.	.	.	.

Bog	59	60	61	62	63	64	65	66	67	68	69	70	71	72	73	74	75	76	77	78	79	80	81	82	83	84	85	86	87
Stuckenia striata	.	.	.	.	.	.	.	.	.	.	.	.	.	.	.	.	.	.	.	.	.	.	.	.	.	.	.	.	.
Symphyotrichum peteroanum	.	.	.	.	.	.	.	.	.	.	.	.	.	.	.	.	.	.	.	.	.	.	.	.	.	.	.	.	.
Symphyotrichum vahlii	.	.	.	.	.	.	.	.	.	.	.	.	.	.	.	.	.	.	.	.	.	.	.	.	.	.	.	.	.
Tetroncium magellanicum	.	.	.	.	.	.	.	.	.	.	.	.	.	.	.	.	.	.	.	.	.	.	.	.	.	.	.	.	.
Tribeles australis	.	.	.	.	.	.	.	.	.	.	.	.	.	.	.	.	.	.	.	.	.	.	.	.	.	.	.	.	.
Trifolium amabile	.	.	.	.	.	.	.	.	.	.	.	.	.	.	.	.	.	.	.	.	.	.	.	.	.	.	.	.	.
Trifolium polymorphum	.	.	.	.	.	.	.	.	.	.	.	.	.	.	.	.	.	.	.	.	.	.	.	.	.	.	.	.	.
Triglochin concinna	.	.	1	1	1	1	1	1	.	.	.	.	.	.	1	1	.	.	.	.	.	.	.	.	.	.	.	.	.
Triglochin palustris	.	1	.	.	.	.	1	1	.	.	1	1	1	.	1	1	.	1	1	1	.	.	.	.	.	.	.	.	.
Triglochin striata	.	.	.	.	.	.	.	.	.	.	.	.	.	.	.	.	.	.	.	.	.	.	.	.	.	.	.	.	.
Trisetum caudulatum	.	.	.	.	.	.	.	.	.	.	.	.	.	.	.	.	.	.	.	.	.	.	.	.	.	.	.	.	.
Trisetum preslei	.	.	.	.	.	.	.	.	.	.	.	.	.	.	.	.	.	.	.	.	.	.	.	.	.	.	.	.	.
Koeleria spicata	.	.	.	.	.	.	.	.	.	.	.	.	.	.	.	.	.	.	.	.	.	.	.	.	.	.	.	.	.
Utricularia gibba	.	.	.	.	.	.	.	.	.	.	.	.	.	.	.	.	.	.	.	.	.	.	.	.	.	.	.	.	.
Vahlodea atropurpurea	.	.	.	.	.	.	.	.	.	.	.	.	.	.	.	.	.	.	.	.	.	.	.	.	.	.	.	.	.
Valeriana fonckii	.	.	.	.	.	.	.	.	.	.	.	.	.	.	.	.	.	.	.	.	.	.	.	.	.	.	.	.	.
Valeriana macrorhiza	.	.	.	.	.	.	.	.	.	.	.	.	.	.	.	.	.	.	.	.	.	.	.	.	.	.	.	.	.
Viola pygmaea	.	.	.	.	.	.	.	.	.	.	.	.	.	.	.	.	.	.	.	.	1	.	1	1	1	.	1	1	1
Werneria apiculata	.	.	.	.	.	.	.	.	.	.	.	.	.	.	.	.	.	.	.	.	.	.	.	.	.	.	.	.	.
Werneria pinnatifida	1	.	.	.	.	1	.	.	1	1	.	.	.	1	.	.	.	.	.	.	1	1	.	1	1	1	.	1	1
Werneria pygmaea	1	1	.	1	1	.	1	1	.	.	.	.	.	.	.	1	1	1	1	1	1	1	1	1	1	1	1	1	1
Werneria solivifolia	.	.	.	.	.	.	.	.	.	.	.	.	.	.	.	.	.	.	.	.	1	1	.	.	.	.	1	.	.
Werneria spathulata	.	.	.	.	.	.	.	.	.	.	.	.	.	.	.	.	.	.	.	.	1	1	.	.	.	.	.	.	.
Xenophyllum incisum	.	.	.	.	.	.	.	.	.	.	.	.	.	.	.	.	.	.	.	.	.	.	.	.	.	.	.	.	.
Zameioscirpus atacamensis	.	.	.	.	.	1	1	.	1	1	1	1	.	1	1	1	.	.	.	.	.	.	.	.	.	.	.	.	.
Zameioscirpus gaimardiodes	.	.	.	.	.	.	.	.	.	.	.	.	.	.	.	.	.	.	.	.	.	.	.	.	.	.	.	.	.
Zameioscirpus muticus	1	1	1	1	1	.	.	1	.	.	.	.	1	.	.	.	1	1	1	1	1	1	1	1	1	1	1	1	1

Bog	88	89	90	91	92	93	94	95	96	97	98	99	100	101	102	103	104	105	106	107	108	109	110	111	112	113	114	115	116
**Operational zone**	**N**	**N**	**N**	**N**	**N**	**T**	**T**	**T**	**T**	**T**	**T**	**T**	**T**	**T**	**T**	**T**	**N**	**N**	**N**	**N**	**N**	**N**	**N**	**N**	**N**	**T**	**T**	**T**	**T**
**Cluster**	**4**	**4**	**4**	**4**	**4**	**2**	**2**	**2**	**2**	**2**	**2**	**2**	**2**	**2**	**2**	**2**	**1**	**1**	**1**	**1**	**1**	**1**	**1**	**1**	**1**	**2**	**2**	**2**	**2**
**Bioregion**	**T**	**T**	**T**	**T**	**T**	**S**	**T**	**S**	**S**	**T**	**T**	**S**	**T**	**T**	**T**	**T**	**N**	**T**	**N**	**T**	**T**	**T**	**T**	**T**	**N**	**S**	**S**	**S**	**S**
**Longitude**	**-69.01**	**-69.00**	**-69.06**	**-69.09**	**-69.22**	**-70.34**	**-70.10**	**-70.34**	**-70.31**	**-70.28**	**-69.96**	**-69.96**	**-70.48**	**-70.06**	**-70.21**	**-69.89**	**-68.81**	**-68.64**	**-68.55**	**-68.88**	**-68.63**	**-68.72**	**-68.65**	**-68.88**	**-69.19**	**-70.55**	**-70.53**	**-70.31**	**-70.31**
**Latitude**	**-27.15**	**-27.17**	**-27.22**	**-27.48**	**-27.20**	**-30.37**	**-29.58**	**-30.38**	**-30.38**	**-30.40**	**-29.88**	**-29.87**	**-29.75**	**-29.88**	**-29.58**	**-28.72**	**-20.05**	**-20.98**	**-20.98**	**-20.27**	**-19.90**	**-19.52**	**-19.62**	**-19.22**	**-19.18**	**-30.73**	**-30.73**	**-30.57**	**-30.57**
Acaena antarctica	.	.	.	.	.	.	.	.	.	.	.	.	.	.	.	.	.	.	.	.	.	.	.	.	.	.	.	.	.
Acaena macrocephala	.	.	.	.	.	.	.	.	.	.	.	.	.	.	.	.	.	.	.	.	.	.	.	.	.	.	.	.	.
Acaena magellanica	.	.	.	.	.	1	.	1	.	.	1	1	.	.	1	.	.	.	.	.	.	.	.	.	.	.	1	.	1
Acaena ovalifolia	.	.	.	.	.	.	.	.	.	.	.	.	.	.	.	.	.	.	.	.	.	.	.	.	.	.	.	.	.
Acaena pinnatifida	.	.	.	.	.	.	.	.	.	.	.	.	.	.	.	.	.	.	.	.	.	.	.	.	.	.	.	.	.
Adesmia retusa	.	.	.	.	.	.	.	.	.	.	.	.	.	.	.	.	.	.	.	.	.	.	.	.	.	.	.	.	.
Agrostis breviculmis	.	.	.	.	.	.	.	.	.	.	.	.	.	.	.	.	.	.	.	.	.	.	.	.	.	.	.	.	.
Agrostis imberbis	.	.	.	.	.	.	.	.	.	.	.	.	.	.	.	.	.	.	.	.	.	.	.	.	.	.	.	.	.
Agrostis meyenii	.	.	.	.	.	.	.	.	.	.	.	.	.	.	.	.	.	.	.	.	.	.	.	.	.	.	.	.	.
Agrostis perennans	.	.	.	.	.	.	.	.	.	.	.	.	.	.	.	.	.	.	.	.	.	.	.	.	.	.	.	.	.
Alchemilla pinnata	.	.	.	.	.	.	.	.	.	.	.	.	.	.	.	.	.	.	.	.	.	.	.	.	.	.	.	.	.
Alopecurus magellanicus	.	.	.	.	.	.	.	.	.	.	.	.	.	.	.	.	.	.	.	.	.	.	.	.	.	.	.	.	.
Amphiscirpus nevadensis	.	.	.	.	.	.	.	.	.	.	.	.	.	.	.	.	.	.	.	.	.	.	.	.	.	.	.	.	.
Anagallis alternifolia	.	.	.	.	.	.	.	.	.	.	.	.	.	.	.	1	.	.	.	.	.	.	.	.	.	.	.	1	1
Antennaria chilensis	.	.	.	.	.	.	.	.	.	.	.	.	.	.	.	.	.	.	.	.	.	.	.	.	.	.	.	.	.
Anthoxanthum redolens	.	.	.	.	.	.	.	.	.	.	.	.	.	.	.	.	.	.	.	.	.	.	.	.	.	.	.	.	.
Apium panul	.	.	.	.	.	.	.	.	.	.	1	1	.	.	.	1	.	.	.	.	.	.	.	.	.	1	.	1	.
Arenaria rivularis	.	.	.	.	.	.	.	.	.	.	.	.	.	.	.	.	.	.	.	.	.	.	.	.	.	.	.	.	.
Arenaria serpens	.	1	1	1	1	.	1	1	1	.	.	.	1	1	1	.	1	1	1	1	1	1	1	1	1	.	.	.	1
Arjona pusilla	.	.	.	.	.	.	.	.	.	.	.	.	.	.	.	.	.	.	.	.	.	.	.	.	.	.	.	.	.
Astragalus bustillosii	.	.	.	.	.	.	.	1	1	.	.	.	.	1	1	1	.	.	.	.	.	1	.	.	.	.	.	.	1
Astragalus micranthellus	.	.	.	.	.	.	.	.	.	.	.	.	.	.	.	.	.	.	.	.	.	.	.	.	.	.	.	.	.
Azolla filiculoides	.	.	.	.	.	.	.	.	.	.	.	.	.	.	.	.	.	.	.	1	.	.	1	1	.	.	.	.	.
Azorella boelckei	.	.	.	.	.	.	.	.	.	.	.	.	.	.	.	.	.	.	.	.	.	.	.	.	.	.	.	.	.

Bog	88	89	90	91	92	93	94	95	96	97	98	99	100	101	102	103	104	105	106	107	108	109	110	111	112	113	114	115	116
Azorella burkartii	.	.	.	.	.	.	.	.	.	.	.	.	.	.	.	.	.	.	.	.	.	.	.	.	.	.	.	.	.
Azorella cryptantha	.	.	.	.	.	.	.	.	.	.	.	.	.	.	.	.	.	.	.	.	.	.	.	.	.	.	.	.	.
Azorella lycopodioides	.	.	.	.	.	.	.	.	.	.	.	.	.	.	.	.	.	.	.	.	.	.	.	.	.	.	.	.	.
Azorella trifoliolata	.	.	.	.	.	1	1	1	1	.	.	1	.	.	.	1	.	.	.	.	.	.	.	.	.	.	1	1	1
Baccharis acaulis	.	.	.	.	.	.	.	.	.	.	.	.	.	.	.	.	.	.	.	1	1	.	1	1	.	.	.	.	.
Baccharis caespitosa	.	.	.	.	.	.	.	.	.	.	.	.	.	.	.	.	.	.	.	.	.	.	.	.	.	.	.	.	.
Baccharis magellanica	.	.	.	.	.	.	.	.	.	.	.	.	.	.	.	.	.	.	.	.	.	.	.	.	.	.	.	.	.
Belloa chilensis	.	.	.	.	.	.	.	.	.	.	.	.	.	.	.	.	.	.	.	.	.	.	.	.	.	.	.	.	.
Bromus catharticus	.	.	.	.	.	.	.	.	.	.	.	.	.	.	.	.	.	.	.	.	.	.	.	.	.	.	.	.	.
Calandrinia acaulis	.	.	.	.	.	.	.	.	.	.	.	.	.	.	.	.	.	.	.	.	.	.	.	.	.	.	.	.	.
Calandrinia compacta	.	.	.	.	.	.	.	.	.	.	.	.	.	.	.	.	.	.	.	.	.	.	.	.	.	.	.	.	.
Calceolaria biflora	.	.	.	.	.	1	.	1	.	.	.	.	.	.	.	.	.	.	.	.	.	.	.	.	.	.	1	1	1
Calceolaria cana	.	.	.	.	.	.	.	.	.	.	.	.	.	.	.	.	.	.	.	.	.	.	.	.	.	.	.	.	.
Calceolaria corymbosa	.	.	.	.	.	.	.	.	.	.	.	.	.	.	.	.	.	.	.	.	.	.	.	.	.	.	.	.	.
Calceolaria filicaulis	.	.	.	.	.	.	.	.	.	.	.	.	.	.	.	.	.	.	.	.	.	.	.	.	.	.	.	.	.
Callitriche lechleri	.	.	.	.	.	.	.	.	.	.	.	.	.	.	.	.	.	.	.	.	.	.	.	.	.	.	.	.	.
Caltha appendiculata	.	.	.	.	.	.	.	.	.	.	.	.	.	.	.	.	.	.	.	.	.	.	.	.	.	.	.	.	.
Caltha sagittata	.	.	.	.	.	1	.	1	1	.	.	.	.	.	.	.	.	.	.	.	.	.	.	.	.	.	1	1	1
Cardamine cordata	.	.	.	.	.	.	.	.	.	.	.	.	.	.	.	.	.	.	.	.	.	.	.	.	.	.	.	.	.
Cardamine glacialis	.	.	.	.	.	.	.	.	.	.	.	.	.	.	.	1	.	.	.	.	.	.	.	.	.	.	.	.	.
Cardamine tenuirostris	.	.	.	.	.	.	.	.	.	.	.	.	.	.	.	.	.	.	.	.	.	.	.	.	.	.	1	.	.
Cardamine volckmannii	.	.	.	.	.	.	.	.	.	.	.	.	.	.	.	.	.	.	.	.	.	.	.	.	.	.	.	.	.
Carex acaulis	.	.	.	.	.	.	.	.	.	.	.	.	.	.	.	.	.	.	.	.	.	.	.	.	.	.	.	.	.
Carex atropicta	.	.	.	.	.	.	.	1	.	.	.	.	.	.	.	.	.	.	.	.	.	.	.	.	.	.	.	1	1
Carex banksii	.	.	.	.	.	.	.	.	.	.	.	.	.	.	.	.	.	.	.	.	.	.	.	.	.	.	.	.	.
Carex caduca	.	.	.	.	.	.	.	.	.	.	.	.	.	.	.	.	.	.	.	.	.	.	.	.	.	.	.	.	.
Carex decidua	.	.	.	.	.	.	.	.	.	.	.	.	.	.	.	.	.	.	.	.	.	.	.	.	.	.	.	.	.
Carex fuscula	.	.	.	.	.	.	.	.	.	.	.	.	.	.	.	.	.	.	.	.	.	.	.	.	.	1	1	1	.
Carex gayana	1	1	1	.	.	1	1	1	1	1	1	1	1	1	1	1	.	.	.	.	.	.	.	.	.	1	1	1	1
Carex hypoleucos	.	.	.	.	.	.	.	.	.	.	.	.	.	.	.	.	.	.	.	.	.	.	.	.	.	.	.	.	.
Carex macloviana	.	.	.	.	.	.	.	.	.	.	.	.	.	.	.	.	.	.	.	.	.	.	.	.	.	.	.	1	.
Carex magellanica	.	.	.	.	.	.	.	.	.	.	.	.	.	.	.	.	.	.	.	.	.	.	.	.	.	.	.	.	.
Carex malmei	.	.	.	.	.	.	.	.	.	.	.	.	.	.	.	.	.	.	.	.	.	.	.	.	.	.	.	.	.
Carex maritima	1	1	1	1	1	.	1	.	.	.	.	.	1	1	1	1	.	.	.	.	.	.	.	.	.	.	.	.	.
Carex microglochin	.	.	.	.	.	.	.	.	.	.	.	.	.	.	.	.	.	.	.	.	.	.	.	.	.	.	.	1	.
Carex pleioneura	.	.	.	.	.	.	.	.	1	.	.	.	.	.	.	.	.	.	.	.	.	.	.	.	.	.	.	1	.
Carex ruthsatzae	.	.	.	.	.	.	.	.	.	.	.	.	.	.	.	.	.	.	.	.	.	.	.	.	.	.	.	.	.

**Bog**	**88**	**89**	**90**	**91**	**92**	**93**	**94**	**95**	**96**	**97**	**98**	**99**	**100**	**101**	**102**	**103**	**104**	**105**	**106**	**107**	**108**	**109**	**110**	**111**	**112**	**113**	**114**	**115**	**116**
Carex vallis-pulchrae	.	.	.	.	.	.	.	.	.	.	.	1	1	1	.	1	.	.	.	.	.	.	.	.	.	.	.	.	1
Carpha schoenoides	.	.	.	.	.	.	.	.	.	.	.	.	.	.	.	.	.	.	.	.	.	.	.	.	.	.	.	.	.
Castilleja pumila	.	.	.	.	.	.	.	.	.	.	.	.	.	.	.	.	.	.	.	.	.	.	.	.	.	.	.	.	.
Catabrosa werdermannii	1	1	.	.	.	.	1	.	1	1	.	.	1	1	1	.	.	.	.	.	.	1	.	.	.	.	.	.	.
Cerastium humifusum	.	.	.	.	.	.	.	.	.	.	.	.	.	.	.	.	.	.	.	.	.	.	.	.	.	.	.	.	.
Cerastium montioides	.	.	.	.	.	.	1	.	1	1	.	.	1	1	.	.	.	.	.	.	.	.	.	.	.	1	1	1	1
Chiliotrichum diffusum	.	.	.	.	.	.	.	.	.	.	.	.	.	.	.	.	.	.	.	.	.	.	.	.	.	.	.	.	.
Chusquea culeou	.	.	.	.	.	.	.	.	.	.	.	.	.	.	.	.	.	.	.	.	.	.	.	.	.	.	.	.	.
Colobanthus quitensis	.	.	.	.	.	.	1	1	1	.	.	.	1	1	1	1	.	.	1	.	.	1	.	.	.	.	.	.	1
Cortaderia egmontiana	.	.	.	.	.	.	.	.	.	.	.	.	.	.	.	.	.	.	.	.	.	.	.	.	.	.	.	.	.
Cotula mexicana	.	.	.	.	.	.	.	.	.	.	.	.	.	.	.	.	.	.	.	.	.	.	.	.	.	.	.	.	.
Crassula peduncularis	.	.	.	.	.	.	.	.	.	.	.	.	.	.	.	.	.	.	.	.	.	.	.	.	.	.	.	.	.
Cuatrecasasiella argentina	.	.	.	.	.	.	.	.	.	.	.	.	.	.	.	.	.	.	.	.	.	1	.	.	.	.	.	.	.
Deschampsia antarctica	.	.	.	.	.	.	.	.	.	.	.	.	.	.	.	.	.	.	.	.	.	.	.	.	.	.	.	.	.
Deschampsia caespitosa	.	.	.	.	.	.	.	.	.	.	.	.	.	.	.	.	.	.	.	.	.	.	.	.	.	.	.	.	.
Deschampsia patula	.	.	.	.	.	.	.	.	.	.	.	.	.	.	.	.	.	.	.	.	.	.	.	.	.	.	.	.	.
Cinnagrostis brevifolia	.	.	.	.	.	.	.	.	.	.	.	.	.	.	.	.	.	.	.	.	.	.	.	.	.	.	.	.	.
Deschampsia chrysantha	.	.	.	.	.	.	.	.	.	.	.	.	.	.	.	.	1	1	1	.	1	1	.	.	.	.	.	.	.
Cinnagrostis chrysophylla	.	.	.	.	.	.	.	.	.	.	.	.	.	.	.	.	.	.	.	.	.	.	.	.	.	.	.	.	.
Deschampsia chrysostachya	.	.	.	.	.	.	.	.	.	.	.	.	.	.	.	.	.	.	.	.	.	.	.	.	.	.	.	.	.
Deschampsia eminens	.	.	.	.	.	.	.	.	.	.	.	.	.	.	.	.	.	.	.	.	.	.	.	.	.	.	.	.	.
Deschampsia hackelii	.	.	.	.	.	.	.	.	.	.	.	.	.	.	.	.	.	.	.	.	.	.	.	.	.	.	.	.	.
Cinnagrostis minima	.	.	.	.	.	.	.	.	.	.	.	.	.	.	.	.	.	.	.	.	.	.	.	.	.	.	.	.	.
Deschampsia ovata	.	.	.	.	.	.	.	.	.	.	.	.	.	.	.	.	.	.	.	.	.	.	.	.	.	.	.	.	.
Cinnagrostis rigescens	.	.	.	.	.	.	.	.	.	.	.	.	.	.	.	.	.	.	.	.	.	.	.	.	.	.	.	.	.
Cinnagrostis spicigera	.	.	.	.	.	.	.	.	.	.	.	.	.	.	.	.	.	.	.	.	.	.	.	.	.	.	.	.	.
Cinnagrostis velutina	1	1	1	1	1	.	1	1	1	1	.	.	1	1	1	1	1	1	1	1	1	1	1	1	.	.	.	.	1
Cinnagrostis vicunarum	.	.	.	.	.	.	.	.	.	.	.	.	.	.	.	.	.	.	.	.	.	.	.	.	.	.	.	.	.
Distichia filamentosa	.	.	.	.	.	.	.	.	.	.	.	.	.	.	.	.	.	.	.	.	.	.	.	.	.	.	.	.	.
Distichia muscoides	.	.	.	.	.	.	.	.	.	.	.	.	.	.	.	.	1	.	1	.	.	1	.	.	1	.	.	.	.
Distichlis humilis	.	.	.	.	.	.	.	.	.	.	.	.	.	.	.	.	.	.	.	1	1	.	1	1	1	.	.	.	.

Bog	88	89	90	91	92	93	94	95	96	97	98	99	100	101	102	103	104	105	106	107	108	109	110	111	112	113	114	115	116
Distichlis scoparia	.	.	.	.	.	.	.	.	.	.	.	.	.	.	.	.	.	.	.	1	.	.	.	.	.	.	.	.	.
Distichlis spicata	.	.	.	.	.	.	.	.	.	.	.	.	.	.	.	.	.	.	.	.	.	.	.	.	.	.	.	.	.
Draba pusilla	.	.	.	.	.	.	.	.	1	1	.	.	.	.	.	.	.	.	.	.	.	.	.	.	.	.	.	.	.
Eleocharis melanomphala	.	.	.	.	.	.	.	.	.	.	.	.	.	.	.	.	.	.	.	.	.	.	.	.	.	.	.	.	.
Eleocharis pseudoalbibracteata	.	.	.	.	.	.	.	.	.	.	.	.	.	.	.	.	.	.	.	.	.	.	.	.	.	.	.	.	.
Elodea potamogeton	.	.	.	.	.	.	.	.	.	.	.	.	.	.	.	.	.	.	.	.	.	.	.	.	.	.	.	.	.
Empetrum rubrum	.	.	.	.	.	.	.	.	.	.	.	.	.	.	.	.	.	.	.	.	.	.	.	.	.	.	.	.	.
Epilobium australe	.	.	.	.	.	.	.	.	.	.	.	.	.	.	.	.	.	.	.	.	.	.	.	.	.	.	.	.	.
Epilobium barbeyanum	.	.	.	.	.	.	.	.	.	.	.	.	1	.	.	1	.	.	.	.	.	.	.	.	.	.	1	1	.
Epilobium ciliatum	.	.	.	.	.	.	.	.	.	.	.	.	.	.	.	1	.	.	.	.	.	.	.	.	.	.	1	.	.
Epilobium denticulatum	.	.	.	.	.	.	.	.	.	.	.	.	.	.	.	.	1	.	.	.	.	.	.	.	.	1	.	.	.
Epilobium fragile	.	.	.	.	.	.	.	.	.	.	.	.	.	.	.	.	1	.	.	.	.	.	.	.	.	.	.	.	.
Epilobium glaucum	.	.	.	.	.	.	.	.	.	.	.	.	.	.	.	.	.	.	.	.	.	.	.	.	.	.	1	1	.
Epilobium nivale	.	.	.	.	.	.	.	.	.	.	.	1	1	.	.	.	.	.	.	.	.	.	.	.	.	.	1	.	.
Erigeron andicola	.	.	.	.	.	.	.	.	.	.	.	.	.	.	.	.	.	.	.	.	.	.	.	.	.	.	.	.	.
Erigeron leptopetalus	.	.	.	.	.	.	.	.	.	.	.	.	.	.	.	.	.	.	.	.	.	.	.	.	.	.	.	.	.
Erigeron myosotis	.	.	.	.	.	.	.	.	.	.	.	.	.	.	.	.	.	.	.	.	.	.	.	.	.	.	.	.	.
Erigeron patagonicus	.	.	.	.	.	.	.	.	.	.	.	.	.	.	.	.	.	.	.	.	.	.	.	.	.	.	.	.	.
Erythranthe cuprea	.	.	.	.	.	.	.	.	.	.	.	.	.	.	.	.	.	.	.	.	.	.	.	.	.	.	.	.	.
Erythranthe depressa	.	1	.	.	.	.	.	1	1	1	1	1	1	1	1	1	.	.	.	1	.	1	1	.	.	.	1	1	.
Erythranthe glabrata	.	.	.	.	.	.	.	.	.	.	.	.	.	.	.	.	.	.	.	.	.	.	.	.	.	.	.	.	.
Erythranthe lutea	.	.	.	.	.	.	.	.	.	.	.	.	.	.	.	1	.	.	.	.	.	.	.	.	.	.	.	.	.
Escallonia virgata	.	.	.	.	.	.	.	.	.	.	.	.	.	.	.	.	.	.	.	.	.	.	.	.	.	.	.	.	.
Euphrasia antarctica	.	.	.	.	.	.	.	.	.	.	.	.	.	.	.	.	.	.	.	.	.	.	.	.	.	.	.	.	.
Euphrasia chrysantha	.	.	.	.	.	.	.	.	.	.	.	.	.	.	.	.	.	.	.	.	.	.	.	.	.	.	.	.	.
Euphrasia subexserta	.	.	.	.	.	.	.	.	.	.	.	.	.	.	.	.	.	.	.	.	.	.	.	.	.	.	.	.	.
Festuca hypsophila	.	.	.	.	.	.	.	.	.	.	.	.	.	.	.	1	.	.	.	.	.	.	1	1	.	.	.	.	.
Festuca kurtziana	.	.	.	.	.	.	.	.	.	.	.	.	.	.	.	.	.	.	.	.	.	.	.	.	.	.	.	.	.
Festuca lilloi	.	.	.	.	.	.	.	.	.	.	.	.	.	.	.	.	.	.	.	.	.	.	.	.	.	.	.	.	.
Festuca magellanica	.	.	.	.	.	.	.	1	1	.	.	.	.	1	.	.	.	.	.	.	.	.	.	.	.	.	1	.	1
Festuca nardifolia	.	.	.	.	.	.	.	.	.	.	.	.	.	.	.	.	.	.	.	.	.	.	.	.	.	.	.	.	.
Festuca rigescens	.	.	.	.	.	.	.	.	.	.	.	.	.	.	.	.	1	1	1	1	1	1	1	1	1	.	.	.	.
Festuca werdermannii	1	.	1	.	.	.	1	.	.	.	1	1	1	1	1	1	.	.	.	.	.	.	.	.	.	.	.	.	.
Frankenia triandra	.	.	.	.	.	.	.	.	.	.	.	.	.	.	.	.	.	1	.	1	1	.	1	1	.	.	.	.	.
Gamocarpha graminea	.	.	.	.	.	.	.	.	.	.	.	.	.	.	.	.	.	.	.	.	.	.	.	.	.	.	.	.	.

Bog	88	89	90	91	92	93	94	95	96	97	98	99	100	101	102	103	104	105	106	107	108	109	110	111	112	113	114	115	116
Gamocarpha ventosa	.	.	.	.	.	.	.	.	.	.	.	.	.	.	.	.	.	.	.	.	.	.	.	.	.	.	.	.	.
Gamochaeta chamissonis	.	.	.	.	.	.	.	.	.	.	.	.	.	.	.	.	.	.	.	.	.	.	.	.	.	.	.	.	.
Gamochaeta longipedicellata	.	.	.	.	.	.	.	.	.	.	.	.	.	.	.	.	.	.	.	.	.	.	.	.	.	.	.	.	.
Gamochaeta neuquensis	.	.	.	.	.	.	.	.	.	.	.	.	.	.	.	.	.	.	.	.	.	.	.	.	.	.	.	.	.
Gaultheria antarctica	.	.	.	.	.	.	.	.	.	.	.	.	.	.	.	.	.	.	.	.	.	.	.	.	.	.	.	.	.
Gaultheria caespitosa	.	.	.	.	.	.	.	.	.	.	.	.	.	.	.	.	.	.	.	.	.	.	.	.	.	.	.	.	.
Gaultheria pumila	.	.	.	.	.	.	.	.	.	.	.	.	.	.	.	.	.	.	.	.	.	.	.	.	.	.	.	.	.
Gavilea chica	.	.	.	.	.	.	.	.	.	.	.	.	.	.	.	.	.	.	.	.	.	.	.	.	.	.	.	.	.
Gentiana prostrata	.	.	.	.	.	.	1	1	1	1	.	.	.	1	1	1	1	1	1	1	1	1	1	1	1	.	1	.	1
Gentianella fiebrigii	.	.	.	.	.	.	.	.	.	.	.	.	.	.	.	.	.	.	.	.	.	.	.	.	.	.	.	.	.
Gentianella magellanica	.	.	.	.	.	.	.	.	.	.	.	.	.	.	.	.	.	.	.	.	.	.	.	.	.	.	.	.	.
Gentianella multicaulis	.	.	.	.	.	.	.	.	.	.	.	.	.	.	.	.	.	.	.	.	.	.	.	.	.	.	.	.	.
Gentianella ottonis	.	.	.	.	.	.	.	.	.	.	.	.	.	.	.	.	.	.	.	.	.	.	.	.	.	.	.	.	.
Gentianella primuloides	.	.	.	.	.	.	.	.	.	.	.	.	.	.	.	.	.	.	.	.	.	.	.	.	.	.	.	.	.
Gentianella pseudocrassula	.	.	.	.	.	.	.	.	.	.	.	.	.	.	.	.	.	.	.	.	.	.	.	.	.	.	.	.	.
Geranium sessiliflorum	.	.	.	.	.	.	.	.	.	.	.	.	.	.	.	.	.	.	.	.	.	.	.	.	.	.	.	.	.
Gunnera magellanica	.	.	.	.	.	.	.	.	.	.	.	.	.	.	.	.	.	.	.	.	.	.	.	.	.	.	.	.	.
Halenia caespitosa	.	.	.	.	.	.	.	.	.	.	.	.	.	.	.	.	.	.	.	.	.	.	.	.	.	.	.	.	.
Halerpestes cymbalaria	1	1	.	1	1	1	1	.	1	.	.	.	1	1	1	1	.	.	.	.	.	.	1	1	.	.	1	.	.
Halerpestes exilis	.	.	.	.	.	.	.	.	.	.	.	.	.	.	.	.	.	.	.	.	.	.	.	.	.	.	.	.	.
Hieracium antarcticum	.	.	.	.	.	.	.	.	.	.	.	.	.	.	.	.	.	.	.	.	.	.	.	.	.	.	.	.	.
Hordeum comosum	.	.	.	.	.	.	.	.	.	.	.	.	.	.	.	.	.	.	.	.	.	.	.	.	.	.	.	.	.
Hordeum muticum	.	.	.	.	.	.	.	.	.	.	.	.	.	.	.	.	.	.	.	.	.	.	.	.	.	.	.	.	.
Hypochaeris acaulis	.	.	.	.	.	.	1	1	1	1	.	.	.	.	1	.	.	.	.	.	.	.	.	.	.	.	.	.	.
Hypochaeris chondrilloides	.	.	.	.	.	.	.	.	.	.	.	.	.	.	.	.	.	.	.	.	.	.	.	.	.	.	.	.	.
Hypochaeris meyeniana	.	.	.	.	.	.	.	.	.	.	.	.	.	.	.	.	.	.	.	.	.	.	.	.	.	.	.	.	.
Hypochaeris palustris	.	.	.	.	.	.	.	.	.	.	.	.	.	.	.	.	.	.	.	.	.	.	.	.	.	.	.	.	.
Hypochaeris taraxacoides	.	.	.	.	.	.	.	.	.	.	.	.	.	.	.	.	.	.	.	1	1	1	1	1	1	.	.	.	.
Hypochaeris tenerifolia	.	.	.	.	.	.	.	.	.	.	.	.	.	.	.	.	.	.	.	.	.	.	.	.	.	.	.	.	.
Isolepis nigricans	.	.	.	.	.	.	.	.	.	.	.	.	.	.	.	.	.	.	.	.	.	.	.	.	.	.	.	.	.

**Bog**	**88**	**89**	**90**	**91**	**92**	**93**	**94**	**95**	**96**	**97**	**98**	**99**	**100**	**101**	**102**	**103**	**104**	**105**	**106**	**107**	**108**	**109**	**110**	**111**	**112**	**113**	**114**	**115**	**116**
Isolepsis inundata	.	.	.	.	.	.	.	.	.	.	.	.	.	.	.	.	.	.	.	.	.	.	.	.	.	.	.	.	.
Juncus balticus	1	1	1	.	.	1	.	1	1	.	1	1	.	1	1	1	.	.	.	.	.	.	.	.	.	1	1	1	1
Juncus stipulatus	.	.	1	.	.	1	.	1	1	.	1	1	1	1	.	1	1	.	1	1	1	1	1	1	1	1	1	1	1
Koeleria kurtzii	.	.	.	.	.	.	.	.	.	.	.	.	.	.	.	.	.	.	.	.	.	.	.	.	.	.	.	.	.
Lachemilla diplophylla	.	.	.	.	.	.	.	.	.	.	.	.	.	.	.	.	.	.	.	.	.	1	.	.	1	.	.	.	.
Lachemilla pinnata	.	.	.	.	.	.	.	.	.	.	.	.	.	.	.	.	.	.	.	.	.	.	.	.	.	.	.	.	.
Lagenophora nudicaulis	.	.	.	.	.	.	.	.	.	.	.	.	.	.	.	.	.	.	.	.	.	.	.	.	.	.	.	.	.
Lemna minuta	.	.	.	.	.	.	.	.	.	.	.	.	.	.	.	.	.	.	.	.	.	.	.	.	.	.	.	.	.
Leptinella scariosa	.	.	.	.	.	.	.	.	.	.	.	.	.	.	.	.	.	.	.	.	.	.	.	.	.	.	.	1	.
Leucheria candidissima	.	.	.	.	.	.	.	.	.	.	.	.	.	.	.	.	.	.	.	.	.	.	.	.	.	.	.	.	.
Leucheria nutans	.	.	.	.	.	.	.	.	.	.	.	.	.	.	.	.	.	.	.	.	.	.	.	.	.	.	.	.	.
Lilaea scilloides	.	.	.	.	.	.	.	.	.	.	.	.	.	.	.	.	.	.	.	.	.	.	.	.	.	.	.	.	.
Lilaeopsis macloviana	.	1	.	.	.	1	.	1	.	.	1	1	1	1	1	1	1	1	.	1	1	1	1	1	1	1	1	1	1
Limosella australis	.	.	.	.	.	.	.	.	.	.	.	.	.	.	.	.	.	.	.	.	.	.	.	.	.	.	.	.	.
Lobelia oligophylla	1	1	.	.	.	1	.	1	1	.	1	1	1	1	1	1	1	.	1	1	1	1	1	1	1	1	1	1	1
Luzula brachyphylla	.	.	.	.	.	.	.	.	.	.	.	.	.	.	.	.	.	.	.	.	.	.	.	.	.	.	.	.	.
Luzula chilensis	.	.	.	.	.	.	.	.	.	.	.	.	.	.	.	.	.	.	.	.	.	.	.	.	.	.	.	.	.
Luzula racemosa	.	.	.	.	.	.	.	.	.	.	.	.	.	.	.	.	.	.	.	.	.	.	.	.	.	.	.	.	.
Luzula vulcanica	.	.	.	.	.	.	.	.	.	.	.	.	.	.	.	.	.	.	.	.	.	.	.	.	.	.	.	.	.
Lysipomia pumila	.	.	.	.	.	.	.	.	.	.	.	.	.	.	.	.	.	.	.	.	.	.	.	.	.	.	.	.	.
Marsippospermum philippii	.	.	.	.	.	.	.	.	.	.	.	.	.	.	.	.	.	.	.	.	.	.	.	.	.	.	.	.	.
Marsippospermum reichei	.	.	.	.	.	.	.	.	.	.	.	.	.	.	.	.	.	.	.	.	.	.	.	.	.	.	.	.	.
Montia fontana	.	.	.	.	.	.	.	.	.	.	.	.	.	.	.	.	.	.	.	.	.	.	.	.	.	.	.	.	.
Muhlenbergia asperifolia	.	.	.	.	.	.	.	.	.	.	1	1	.	1	.	1	.	.	.	.	.	.	.	.	.	1	.	.	.
Myriophyllum quitense	.	1	.	.	.	1	1	1	1	.	.	.	.	1	.	1	.	.	.	1	.	1	1	1	1	.	1	.	1
Myrosmodes nervosa	.	.	.	.	.	.	.	.	.	.	.	.	.	.	.	.	.	.	.	.	.	1	1	1	1	.	.	.	.
Myrosmodes paludosa	.	.	.	.	.	.	.	.	.	.	.	.	.	.	.	.	.	.	.	.	.	.	.	.	.	.	.	.	.
Myrteola nummularia	.	.	.	.	.	.	.	.	.	.	.	.	.	.	.	.	.	.	.	.	.	.	.	.	.	.	.	.	.
Nanodea muscosa	.	.	.	.	.	.	.	.	.	.	.	.	.	.	.	.	.	.	.	.	.	.	.	.	.	.	.	.	.
Neobartsia crenoloba	.	.	.	.	.	.	.	.	.	.	.	.	.	.	.	.	.	.	.	.	.	.	.	.	.	.	.	.	.
Neobartsia pedicularoides	.	.	.	.	.	.	.	.	.	.	.	.	.	.	.	.	.	.	.	.	.	.	.	.	.	.	.	.	.
Neobartsia peruviana	.	.	.	.	.	.	.	.	.	.	.	.	.	.	.	.	.	.	.	.	.	.	.	.	.	.	.	.	.

Bog	88	89	90	91	92	93	94	95	96	97	98	99	100	101	102	103	104	105	106	107	108	109	110	111	112	113	114	115	116
Nertera granadensis	.	.	.	.	.	.	.	.	.	.	.	.	.	.	.	.	.	.	.	.	.	.	.	.	.	.	.	.	.
Nicoraepoa andina	.	.	.	.	.	.	.	.	.	.	.	.	.	.	.	.	.	.	.	.	.	.	.	.	.	.	.	.	.
Nicoraepoa pugionifolia	.	.	.	.	.	.	.	.	.	.	.	.	.	.	.	.	.	.	.	.	.	.	.	.	.	.	.	.	.
Nicoraepoa subenervis	.	.	.	.	.	.	.	.	.	.	.	.	.	.	.	.	.	.	.	.	.	.	.	.	.	.	.	.	.
Nitrophila australis	.	.	.	.	.	.	.	.	.	.	.	.	.	.	.	.	.	.	.	.	.	.	.	.	.	.	.	.	.
Nothofagus antarctica	.	.	.	.	.	.	.	.	.	.	.	.	.	.	.	.	.	.	.	.	.	.	.	.	.	.	.	.	.
Nototriche rugosa	.	.	.	.	.	.	.	.	.	.	.	.	.	.	.	.	.	.	.	.	.	.	.	.	.	.	.	.	.
Ochetophila nana	.	.	.	.	.	1	.	1	1	.	.	.	.	.	.	.	.	.	.	.	.	.	.	.	.	.	.	.	.
Olsynium junceum	.	.	.	.	.	.	.	.	.	.	.	.	.	.	.	1	.	.	.	.	.	.	.	.	.	.	.	.	.
Oreobolus obtusangulus	.	.	.	.	.	.	.	.	.	.	.	.	.	.	.	.	.	.	.	.	.	.	.	.	.	.	.	.	.
Oritrophium limnophilum	.	.	.	.	.	.	.	.	.	.	.	.	.	.	.	.	.	.	.	.	.	.	.	.	.	.	.	.	.
Osmorhiza glabrata	.	.	.	.	.	.	.	.	.	.	.	.	.	.	.	.	.	.	.	.	.	.	.	.	.	.	.	.	.
Ourisia alpina	.	.	.	.	.	.	.	.	.	.	.	.	.	.	.	.	.	.	.	.	.	.	.	.	.	.	.	.	.
Ourisia muscosa	.	.	.	.	.	.	.	.	.	.	.	.	.	.	.	.	.	.	.	.	.	.	.	.	.	.	.	.	.
Ourisia ruelloides	.	.	.	.	.	.	.	.	.	.	.	.	.	.	.	.	.	.	.	.	.	.	.	.	.	.	.	.	.
Oxychloe andina	.	1	1	.	1	.	1	.	1	.	.	.	.	1	1	1	1	.	1	1	1	1	1	1	1	.	.	.	.
Oxychloe bisexualis	.	.	.	.	.	.	.	.	.	.	.	.	.	.	.	.	.	.	.	.	.	.	.	.	.	.	.	.	.
Oxychloe castellanosii	.	.	.	.	.	.	.	.	.	.	.	.	.	.	.	.	.	.	.	.	.	.	.	.	.	.	.	.	.
Oxychloe haumaniana	.	.	.	.	.	.	.	.	.	.	.	.	.	.	.	.	.	.	.	.	.	.	.	.	.	.	.	.	.
Oxychloe mendocina	.	.	.	.	.	.	.	.	.	.	.	.	.	.	.	.	.	.	.	.	.	.	.	.	.	.	.	.	.
Patosia clandestina	.	1	.	.	.	.	1	1	1	1	.	1	1	1	1	1	.	.	.	.	.	.	.	.	.	.	.	.	1
Perezia capito	.	.	.	.	.	.	.	.	.	.	.	.	.	.	.	.	.	.	.	.	.	.	.	.	.	.	.	.	.
Perezia delicata	.	.	.	.	.	.	.	.	.	.	.	.	.	.	.	.	.	.	.	.	.	.	.	.	.	.	.	.	.
Perezia fonkii	.	.	.	.	.	.	.	.	.	.	.	.	.	.	.	.	.	.	.	.	.	.	.	.	.	.	.	.	.
Perezia pedicularidifolia	.	.	.	.	.	.	.	.	.	.	.	.	.	.	.	.	.	.	.	.	.	.	.	.	.	.	.	.	.
Perezia pinnatifida	.	.	.	.	.	.	.	.	.	.	.	.	.	.	.	.	.	.	.	.	.	.	.	.	.	.	.	.	.
Petroravenia friesii	.	.	.	.	.	.	.	.	.	.	.	.	.	.	.	.	.	.	.	.	.	.	.	.	.	.	.	.	.
Petroravenia werdermannii	.	.	.	.	.	.	.	.	.	.	.	.	.	.	.	.	.	.	.	.	.	.	.	.	.	.	.	.	.
Phleum alpinum	.	.	.	.	.	.	.	1	1	.	.	.	.	.	.	.	.	.	.	.	.	.	.	.	.	.	1	.	.
Phylloscirpus acaulis	1	.	.	.	.	1	.	1	.	.	1	1	1	1	1	.	.	.	.	.	.	1	1	1	.	1	1	1	1
Phylloscirpus boliviensis	.	.	.	.	.	.	.	.	.	.	.	.	.	.	.	.	.	.	.	.	.	.	.	.	.	.	.	.	.

**Bog**	**88**	**89**	**90**	**91**	**92**	**93**	**94**	**95**	**96**	**97**	**98**	**99**	**100**	**101**	**102**	**103**	**104**	**105**	**106**	**107**	**108**	**109**	**110**	**111**	**112**	**113**	**114**	**115**	**116**
Phylloscirpus deserticola	.	.	.	.	.	.	.	.	.	.	.	.	.	.	.	1	1	.	1	1	1	1	1	1	1	.	.	.	.
Pinguicula antarctica	.	.	.	.	.	.	.	.	.	.	.	.	.	.	.	.	.	.	.	.	.	.	.	.	.	.	.	.	.
Plantago barbata	.	.	.	.	.	1	1	1	1	1	.	1	1	1	1	1	.	.	.	.	.	.	.	.	.	.	1	1	1
Plantago rigida	.	.	.	.	.	.	.	.	.	.	.	.	.	.	.	.	.	.	.	.	.	.	.	.	.	.	.	.	.
Plantago tubulosa	.	.	.	.	.	.	.	.	.	.	.	.	.	.	.	.	.	.	.	.	.	.	.	.	.	.	.	.	.
Plantago uniglumis	.	.	.	.	.	1	.	1	1	1	.	.	.	.	.	.	.	.	.	.	.	.	.	.	.	.	.	.	1
Poa alopecurus	.	.	.	.	.	.	.	.	.	.	.	.	.	.	.	.	.	.	.	.	.	.	.	.	.	.	.	.	.
Poa hachadoensis	.	.	.	.	.	.	.	.	.	.	.	.	.	.	.	.	.	.	.	.	.	.	.	.	.	.	.	.	.
Poa perligulata	.	.	.	.	.	.	.	.	.	.	.	.	.	.	.	.	.	.	1	.	.	.	.	.	1	.	.	.	.
Polypogon interruptus	.	.	.	.	.	.	.	1	.	.	1	1	1	.	.	1	.	.	.	.	.	.	1	.	.	1	1	1	1
Primula magellanica	.	.	.	.	.	.	.	.	.	.	.	.	.	.	.	.	.	.	.	.	.	.	.	.	.	.	.	.	.
Puccinellia frigida	1	1	1	1	1	.	.	.	.	1	.	.	1	1	1	1	1	1	.	1	1	.	1	1	.	.	.	.	.
Quinchamalium chilense	.	.	.	.	.	.	.	.	.	.	.	.	.	.	.	.	.	.	.	.	.	.	.	.	.	.	.	.	.
Ranunculus breviscapus	.	.	.	.	.	.	.	.	.	.	.	.	.	.	.	.	.	.	.	.	.	.	.	.	.	.	.	.	.
Ranunculus fuegianus	.	.	.	.	.	.	.	.	.	.	.	.	.	.	.	.	.	.	.	.	.	.	.	.	.	.	.	.	.
Ranunculus mandonianus	.	.	.	.	.	.	.	.	.	.	.	.	.	.	.	.	.	.	.	.	.	.	.	.	.	.	.	.	.
Ranunculus peduncularis	.	.	.	.	.	.	.	1	.	.	.	.	.	.	.	.	.	.	.	.	.	.	.	.	.	.	1	1	.
Ranunculus trichophyllus	.	.	.	.	.	.	.	.	.	.	.	.	.	.	.	.	.	.	.	.	.	1	1	.	.	.	.	.	.
Halerpestes uniflora	.	.	.	.	.	.	.	.	.	.	.	.	.	.	.	.	.	.	.	.	.	.	.	.	.	.	.	.	.
Rubus geoides	.	.	.	.	.	.	.	.	.	.	.	.	.	.	.	.	.	.	.	.	.	.	.	.	.	.	.	.	.
Rumex magellanicus	.	.	.	.	.	.	.	1	.	.	1	.	.	.	.	.	.	.	.	.	.	.	.	.	.	1	1	1	.
Rytidosperma lechleri	.	.	.	.	.	.	.	.	.	.	.	.	.	.	.	.	.	.	.	.	.	.	.	.	.	.	.	.	.
Sarcocornia pulvinata	.	.	.	.	.	.	.	.	.	.	.	.	.	.	.	.	.	.	.	1	1	.	1	.	.	.	.	.	.
Schoenoplectus pungens	.	.	.	.	.	.	.	.	.	.	.	1	.	.	.	1	.	.	.	.	.	.	.	.	.	1	.	.	.
Schoenus andinus	.	.	.	.	.	.	.	.	.	.	.	.	.	.	.	.	.	.	.	.	.	.	.	.	.	.	.	.	.
Senecio breviscapus	.	.	.	.	.	.	.	.	.	.	.	.	.	.	.	.	.	.	.	.	.	.	.	.	.	.	.	.	.
Senecio diemii	.	.	.	.	.	.	.	.	.	.	.	.	.	.	.	.	.	.	.	.	.	.	.	.	.	.	.	.	.
Senecio fistulosus	.	.	.	.	.	.	.	.	.	.	.	.	.	.	.	.	.	.	.	.	.	.	.	.	.	.	.	.	.
Senecio parodii	.	.	.	.	.	.	.	.	.	.	.	.	.	.	.	.	.	.	.	.	.	.	.	.	.	.	.	.	.
Senecio peteroanus	.	.	.	.	.	.	.	.	.	.	.	.	.	.	.	.	.	.	.	.	.	.	.	.	.	.	.	.	.
Senecio serratifolius	.	.	.	.	.	.	.	.	.	.	.	.	.	.	.	.	1	.	.	.	.	.	.	.	.	.	.	.	.

**Bog**	**88**	**89**	**90**	**91**	**92**	**93**	**94**	**95**	**96**	**97**	**98**	**99**	**100**	**101**	**102**	**103**	**104**	**105**	**106**	**107**	**108**	**109**	**110**	**111**	**112**	**113**	**114**	**115**	**116**
Senecio trifurcatus	.	.	.	.	.	.	.	.	.	.	.	.	.	.	.	.	.	.	.	.	.	.	.	.	.	.	.	.	.
Sisyrinchium chilense	.	.	.	.	.	.	.	.	.	.	.	.	.	.	.	.	.	.	.	.	.	.	.	.	.	.	.	.	.
Sisyrinchium patagonicum	.	.	.	.	.	.	.	.	.	.	.	.	.	.	.	.	.	.	.	.	.	.	.	.	.	.	.	.	.
Sisyrinchium pearcei	.	.	.	.	.	.	.	.	.	.	.	.	.	.	.	.	.	.	.	.	.	.	.	.	.	.	.	.	.
Stellaria debilis	.	.	.	.	.	.	.	.	.	.	.	.	.	.	.	.	.	.	.	.	.	.	.	.	.	.	.	.	.
Stuckenia filiformis	.	1	.	.	1	.	1	.	1	.	.	.	.	1	1	1	.	.	.	1	.	1	1	1	.	.	.	.	.
Stuckenia striata	.	.	.	.	.	.	.	.	.	.	.	.	.	.	.	.	.	.	.	.	.	.	.	.	.	.	.	.	.
Symphyotrichum peteroanum	.	.	.	.	.	.	.	.	.	.	.	.	.	.	.	.	.	.	.	.	.	.	.	.	.	.	.	.	.
Symphyotrichum vahlii	.	.	.	.	.	.	.	.	.	.	.	.	.	.	.	.	.	.	.	.	.	.	.	.	.	.	.	.	.
Tetroncium magellanicum	.	.	.	.	.	.	.	.	.	.	.	.	.	.	.	.	.	.	.	.	.	.	.	.	.	.	.	.	.
Tribeles australis	.	.	.	.	.	.	.	.	.	.	.	.	.	.	.	.	.	.	.	.	.	.	.	.	.	.	.	.	.
Trifolium amabile	.	.	.	.	.	.	.	.	.	.	.	.	.	.	.	.	.	.	.	.	.	.	.	.	.	.	.	.	.
Trifolium polymorphum	.	.	.	.	.	1	.	1	.	.	.	.	.	.	.	1	.	.	.	.	.	.	.	.	.	1	1	1	1
Triglochin concinna	.	.	.	.	.	.	.	.	.	.	.	.	.	.	.	.	.	.	.	.	.	.	.	.	.	.	.	.	.
Triglochin palustris	1	1	.	.	.	.	.	.	.	.	1	.	.	.	.	1	.	.	.	.	.	.	.	.	.	.	.	.	.
Triglochin striata	1	1	.	.	1	.	.	.	.	.	1	1	.	.	1	.	1	.	.	1	1	.	.	1	.	.	.	.	1
Trisetum caudulatum	.	.	.	.	.	1	.	.	.	.	.	.	.	.	.	.	.	.	.	.	.	.	.	.	.	.	.	1	.
Trisetum preslei	.	.	.	.	.	.	.	.	.	.	.	.	.	.	.	1	.	.	.	.	.	.	.	.	.	.	.	.	.
Koeleria spicata	.	.	.	.	.	.	.	.	.	.	.	.	.	.	.	.	.	.	.	.	.	.	.	.	.	.	.	.	.
Utricularia gibba	.	.	.	.	.	.	.	.	.	.	.	.	.	.	.	1	.	.	.	.	.	.	.	.	.	1	.	.	.
Vahlodea atropurpurea	.	.	.	.	.	.	.	.	.	.	.	.	.	.	.	.	.	.	.	.	.	.	.	.	.	.	.	.	.
Valeriana fonckii	.	.	.	.	.	.	.	.	.	.	.	.	.	.	.	.	.	.	.	.	.	.	.	.	.	.	.	.	.
Valeriana macrorhiza	.	.	.	.	.	.	.	.	.	.	.	.	.	.	.	.	.	.	.	.	.	.	.	.	.	.	.	.	.
Viola pygmaea	.	.	.	.	.	.	.	.	.	.	.	.	.	.	.	.	.	.	.	.	.	.	.	.	.	.	.	.	.
Werneria apiculata	.	.	.	.	.	.	.	.	.	.	.	.	.	.	.	.	1	.	.	.	.	1	.	.	.	.	.	.	.
Werneria pinnatifida	.	.	.	.	.	.	.	.	.	.	.	.	.	.	.	.	.	.	.	.	.	.	.	.	.	.	.	1	.
Werneria pygmaea	.	.	.	.	.	.	1	1	1	1	.	1	1	1	1	1	1	1	1	.	.	1	1	1	1	.	.	1	1
Werneria solivifolia	.	.	.	.	.	.	.	.	.	.	.	.	.	.	.	.	.	.	.	.	.	1	.	1	1	.	.	.	.
Werneria spathulata	.	.	.	.	.	.	.	.	.	.	.	.	.	.	.	.	1	.	1	.	.	1	1	.	.	.	.	.	.
Xenophyllum incisum	.	.	.	.	.	.	.	.	.	.	.	.	.	.	.	.	.	.	.	.	.	.	.	.	.	.	.	.	.
Zameioscirpus atacamensis	.	.	.	.	.	.	.	.	.	.	.	.	.	.	.	.	.	.	.	.	.	.	.	.	.	.	.	.	.
Zameioscirpus gaimardiodes	.	.	.	.	.	.	.	.	.	.	.	.	.	.	.	.	.	.	.	.	.	.	.	.	.	.	.	.	.
Zameioscirpus muticus	.	.	.	.	.	.	.	.	.	.	.	.	.	.	.	.	.	.	.	.	.	.	.	.	.	.	.	.	.

Bog	117	118	119	120	121	122	123	124	125	126	127	128	129	130	131	132	133	134	135	136	137	138	139	140	141	142	143	144	145
**Operational zone**	**T**	**S**	**S**	**N**	**N**	**N**	**N**	**N**	**N**	**N**	**N**	**N**	**N**	**N**	**N**	**N**	**N**	**N**	**N**	**N**	**N**	**N**	**N**	**N**	**N**	**N**	**N**	**N**	**N**
**Cluster**	**2**	**9**	**9**	**1**	**1**	**1**	**1**	**1**	**1**	**1**	**1**	**1**	**1**	**1**	**1**	**1**	**1**	**1**	**1**	**1**	**1**	**1**	**1**	**1**	**1**	**1**	**1**	**1**	**1**
**Bioregion**	**S**	**S**	**S**	**N**	**N**	**N**	**N**	**N**	**N**	**N**	**N**	**N**	**N**	**N**	**N**	**N**	**N**	**N**	**N**	**N**	**N**	**N**	**N**	**N**	**N**	**N**	**N**	**N**	**N**
**Longitude**	**-70.28**	**-71.88**	**-72.03**	**-68.45**	**-69.29**	**-69.18**	**-68.09**	**-68.15**	**-68.12**	**-68.29**	**-68.97**	**-67.38**	**-69.34**	**-69.37**	**-68.44**	**-69.31**	**-69.17**	**-69.19**	**-67.38**	**-69.19**	**-67.38**	**-67.38**	**-68.35**	**-68.36**	**-68.44**	**-67.38**	**-67.38**	**-69.39**	**-69.44**
**Latitude**	**-30.58**	**-41.08**	**-41.08**	**-17.03**	**-17.36**	**-17.38**	**-16.45**	**-16.44**	**-16.45**	**-16.29**	**-18.36**	**-17.02**	**-17.24**	**-17.3**	**-17.03**	**-17.25**	**-17.94**	**-17.93**	**-16.97**	**-17.96**	**-16.97**	**-16.97**	**-16.14**	**-16.12**	**-17.03**	**-16.97**	**-16.97**	**-17.31**	**-17.32**
Acaena antarctica	.	1	.	.	.	.	.	.	.	.	.	.	.	.	.	.	.	.	.	.	.	.	.	.	.	.	.	.	.
Acaena macrocephala	.	.	.	.	.	.	.	.	.	.	.	.	.	.	.	.	.	.	.	.	.	.	.	.	.	.	.	.	.
Acaena magellanica	.	.	.	.	.	.	.	.	.	.	.	.	.	.	.	.	.	.	.	.	.	.	.	.	.	.	.	.	.
Acaena ovalifolia	.	1	.	.	.	.	.	.	.	.	.	.	.	.	.	.	.	.	.	.	.	.	.	.	.	.	.	.	.
Acaena pinnatifida	.	.	.	.	.	.	.	.	.	.	.	.	.	.	.	.	.	.	.	.	.	.	.	.	.	.	.	.	.
Adesmia retusa	.	.	1	.	.	.	.	.	.	.	.	.	.	.	.	.	.	.	.	.	.	.	.	.	.	.	.	.	.
Agrostis breviculmis	.	.	.	.	.	.	.	.	.	.	.	.	.	.	.	.	.	.	.	.	.	.	.	.	.	.	.	.	.
Agrostis imberbis	.	.	.	.	.	.	.	.	.	.	.	.	.	.	.	.	.	.	.	.	.	.	.	.	.	.	.	.	.
Agrostis meyenii	.	.	.	.	.	.	.	.	.	.	.	.	.	.	.	.	.	.	.	.	.	.	.	.	.	.	.	.	.
Agrostis perennans	.	.	.	.	.	.	.	.	.	.	.	.	.	.	.	.	.	.	.	.	.	.	.	.	.	.	.	.	.
Alchemilla pinnata	.	.	.	.	.	.	.	.	.	.	.	.	.	.	.	.	.	.	.	.	.	.	.	.	.	.	.	.	.
Alopecurus magellanicus	.	.	.	.	.	.	.	.	.	.	.	.	.	.	.	.	.	.	.	.	.	.	.	.	.	.	.	.	.
Amphiscirpus nevadensis	.	.	.	.	.	.	.	.	.	.	.	.	.	.	.	.	.	.	.	.	.	.	.	.	.	.	.	.	.
Anagallis alternifolia	.	.	.	.	.	.	.	.	.	.	.	.	.	.	.	.	.	.	.	.	.	.	.	.	.	.	.	.	.
Antennaria chilensis	.	.	.	.	.	.	.	.	.	.	.	.	.	.	.	.	.	.	.	.	.	.	.	.	.	.	.	.	.
Anthoxanthum redolens	.	.	.	.	.	.	.	.	.	.	.	.	.	.	.	.	.	.	.	.	.	.	.	.	.	.	.	.	.
Apium panul	.	.	.	.	.	.	.	.	.	.	.	.	.	.	.	.	.	.	.	.	.	.	.	.	.	.	.	.	.
Arenaria rivularis	.	.	.	.	.	.	.	.	.	.	.	.	.	.	.	.	.	.	.	.	.	.	.	.	.	.	.	.	.
Arenaria serpens	.	.	.	.	.	.	.	.	.	.	.	.	.	.	.	.	.	.	.	.	.	.	.	.	.	.	.	.	.
Arjona pusilla	.	.	.	.	.	.	1	1	1	1	1	.	.	.	.	.	.	.	.	.	.	.	.	.	.	.	.	.	.
Astragalus bustillosii	1	.	.	.	.	.	.	.	.	.	.	.	.	.	.	.	.	.	.	.	.	.	.	.	.	.	.	.	.
Astragalus micranthellus	.	.	.	.	.	.	.	.	.	.	.	.	.	.	.	.	.	.	.	.	.	.	.	.	.	.	.	.	.
Azolla filiculoides	.	.	.	1	.	.	.	.	.	.	.	.	.	.	.	.	1	.	.	.	.	.	.	.	1	.	.	.	.

Bog	117	118	119	120	121	122	123	124	125	126	127	128	129	130	131	132	133	134	135	136	137	138	139	140	141	142	143	144	145
Azorella boelckei	.	.	.	.	.	.	.	.	.	.	.	.	.	.	.	.	.	.	.	.	.	.	.	.	.	.	.	.	.
Azorella burkartii	.	.	.	.	.	.	.	.	.	.	.	.	.	.	.	.	.	.	.	.	.	.	.	.	.	.	.	.	.
Azorella cryptantha	.	.	.	.	.	.	.	.	.	.	.	.	.	.	.	.	.	.	.	.	.	.	.	.	.	.	.	.	.
Azorella lycopodioides	.	.	1	.	.	.	.	.	.	.	.	.	.	.	.	.	.	.	.	.	.	.	.	.	.	.	.	.	.
Azorella trifoliolata	1	.	.	.	.	.	.	.	.	.	.	.	.	.	.	.	.	.	.	.	.	.	.	.	.	.	.	.	.
Baccharis acaulis	.	.	.	.	.	1	.	.	.	.	.	.	.	.	.	.	.	.	.	.	.	.	.	.	.	.	.	.	.
Baccharis caespitosa	.	.	.	.	.	.	1	1	1	.	1	.	1	.	.	.	.	.	.	.	.	.	.	1	.	.	.	.	.
Baccharis magellanica	.	1	1	.	.	.	.	.	.	.	.	.	.	.	.	.	.	.	.	.	.	.	.	.	.	.	.	.	.
Belloa chilensis	.	.	1	.	.	.	.	.	.	.	.	.	.	.	.	.	.	.	.	.	.	.	.	.	.	.	.	.	.
Bromus catharticus	.	.	.	.	.	.	.	.	.	.	.	.	.	.	.	.	.	.	.	.	.	.	.	.	.	.	.	.	.
Calandrinia acaulis	.	.	.	.	.	.	.	.	.	.	.	.	.	.	.	.	.	.	.	.	.	.	.	.	.	.	.	.	.
Calandrinia compacta	.	.	.	1	.	1	.	.	.	1	.	.	.	.	.	.	1	1	.	1	.	.	.	.	1	.	1	.	.
Calceolaria biflora	1	.	.	.	.	.	.	.	.	.	.	.	.	.	.	.	.	.	.	.	.	.	.	.	.	.	.	.	.
Calceolaria cana	.	.	.	.	.	.	.	.	.	.	.	.	.	.	.	.	.	.	.	.	.	.	.	.	.	.	.	.	.
Calceolaria corymbosa	.	.	.	.	.	.	.	.	.	.	.	.	.	.	.	.	.	.	.	.	.	.	.	.	.	.	.	.	.
Calceolaria filicaulis	.	.	.	.	.	.	.	.	.	.	.	.	.	.	.	.	.	.	.	.	.	.	.	.	.	.	.	.	.
Callitriche lechleri	.	.	.	.	.	.	.	.	.	.	.	.	.	.	.	.	.	.	.	.	.	.	.	.	.	.	.	.	.
Caltha appendiculata	.	1	1	.	.	.	.	.	.	.	.	.	.	.	.	.	.	.	.	.	.	.	.	.	.	.	.	.	.
Caltha sagittata	.	.	.	1	.	.	1	.	.	.	1	1	.	.	1	.	.	.	.	.	.	.	1	.	1	.	.	.	.
Cardamine cordata	.	.	.	.	.	.	.	.	.	.	.	.	.	.	.	.	.	.	.	.	.	.	.	.	.	.	.	.	.
Cardamine glacialis	.	.	.	.	.	.	.	.	.	.	.	.	.	.	.	.	.	.	.	.	.	.	.	.	.	.	.	.	.
Cardamine tenuirostris	.	.	.	.	.	.	.	.	.	.	.	.	.	.	.	.	.	.	.	.	.	.	.	.	.	.	.	.	.
Cardamine volckmannii	.	.	.	.	.	.	.	.	.	.	.	.	.	.	.	.	.	.	.	.	.	.	.	.	.	.	.	.	.
Carex acaulis	.	.	.	.	.	.	.	.	.	.	.	.	.	.	.	.	.	.	.	.	.	.	.	.	.	.	.	.	.
Carex atropicta	.	.	.	.	.	.	.	.	.	.	.	.	.	.	.	.	.	.	.	.	.	.	.	.	.	.	.	.	.
Carex banksii	.	1	.	.	.	.	.	.	.	.	.	.	.	.	.	.	.	.	.	.	.	.	.	.	.	.	.	.	.
Carex caduca	.	.	.	.	.	.	.	.	.	.	.	.	.	.	.	.	.	.	.	.	.	.	.	.	.	.	.	.	.
Carex decidua	.	1	.	.	.	.	.	.	.	.	.	.	.	.	.	.	.	.	.	.	.	.	.	.	.	.	.	.	.
Carex fuscula	.	1	.	.	.	.	.	.	.	.	.	.	.	.	.	.	.	.	.	.	.	.	.	.	.	.	.	.	.
Carex gayana	1	.	.	.	.	.	.	.	.	.	.	.	.	.	.	.	.	.	.	.	1	.	.	.	.	.	.	.	.
Carex hypoleucos	.	.	.	.	.	.	.	.	.	.	.	.	.	.	.	.	.	.	.	.	.	.	.	.	.	.	.	.	.
Carex macloviana	.	.	1	.	.	.	.	.	.	.	.	.	.	.	.	.	.	.	.	.	.	.	.	.	.	.	.	.	.
Carex magellanica	.	1	.	.	.	.	.	.	.	.	.	.	.	.	.	.	.	.	.	.	.	.	.	.	.	.	.	.	.
Carex malmei	.	.	.	.	.	.	.	.	.	.	.	.	.	.	.	.	.	.	.	.	.	.	.	.	.	.	.	.	.
Carex maritima	.	.	.	.	.	1	1	.	.	.	1	.	1	1	.	1	1	1	.	1	.	.	.	.	1	.	.	.	.
Carex microglochin	.	.	.	.	.	.	1	.	1	.	.	.	.	.	.	.	.	.	.	.	.	.	.	.	.	.	.	.	.
Carex pleioneura	1	.	.	.	.	.	.	.	.	.	.	.	.	.	.	.	.	.	.	.	.	.	.	.	.	.	.	.	.
Carex ruthsatzae	.	.	.	.	.	.	.	.	.	.	.	.	.	.	.	.	.	.	.	.	.	.	.	1	.	.	.	.	.

**Bog**	**117**	**118**	**119**	**120**	**121**	**122**	**123**	**124**	**125**	**126**	**127**	**128**	**129**	**130**	**131**	**132**	**133**	**134**	**135**	**136**	**137**	**138**	**139**	**140**	**141**	**142**	**143**	**144**	**145**
Carex vallis-pulchrae	1	.	.	.	.	.	.	1	.	.	.	.	.	.	.	.	.	.	.	.	.	.	.	.	.	.	.	.	.
Carpha schoenoides	.	1	.	.	.	.	.	.	.	.	.	.	.	.	.	.	.	.	.	.	.	.	.	.	.	.	.	.	.
Castilleja pumila	.	.	.	1	1	.	1	1	1	.	.	.	1	1	1	1	1	.	.	.	.	.	.	.	1	.	.	1	1
Catabrosa werdermannii	.	.	.	.	.	.	.	.	.	.	.	.	.	.	.	.	.	.	.	.	.	.	.	.	.	.	.	.	.
Cerastium humifusum	.	.	.	.	.	.	.	.	.	.	.	.	.	.	.	.	.	.	.	.	.	.	.	.	.	.	.	.	.
Cerastium montioides	1	.	.	.	.	.	.	.	.	.	.	.	.	.	.	.	.	.	.	.	.	.	.	.	.	.	.	.	.
Chiliotrichum diffusum	.	.	.	.	.	.	.	.	.	.	.	.	.	.	.	.	.	.	.	.	.	.	.	.	.	.	.	.	.
Chusquea culeou	.	.	.	.	.	.	.	.	.	.	.	.	.	.	.	.	.	.	.	.	.	.	.	.	.	.	.	.	.
Colobanthus quitensis	1	.	.	1	.	1	.	.	.	.	1	.	.	.	.	.	1	1	.	1	.	1	.	.	.	.	1	.	.
Cortaderia egmontiana	.	.	.	.	.	.	.	.	.	.	.	.	.	.	.	.	.	.	.	.	.	.	.	.	.	.	.	.	.
Cotula mexicana	.	.	.	1	1	.	1	.	.	.	1	.	1	1	1	1	1	1	.	.	.	.	1	1	1	.	.	1	1
Crassula peduncularis	.	.	.	.	.	.	.	.	.	.	.	.	.	.	.	.	.	.	.	.	.	.	.	.	.	.	.	.	.
Cuatrecasasiella argentina	.	.	.	1	.	1	1	1	1	1	1	1	1	1	1	1	1	.	.	.	.	.	1	.	1	.	.	.	.
Deschampsia antarctica	.	.	.	.	.	.	.	.	.	.	.	.	.	.	.	.	.	.	.	.	.	.	.	.	.	.	.	.	.
Deschampsia caespitosa	.	.	.	.	.	.	.	.	.	.	.	.	.	.	.	.	.	.	.	.	.	.	.	.	.	.	.	.	.
Deschampsia patula	.	.	.	.	.	.	.	.	.	.	.	.	.	.	.	.	.	.	.	.	.	.	.	.	.	.	.	.	.
Cinnagrostis brevifolia	.	.	.	.	.	.	.	.	.	.	.	.	.	.	.	.	.	.	.	.	.	.	.	.	.	.	.	.	.
Deschampsia chrysantha	.	.	.	.	.	.	1	.	.	.	.	.	.	.	.	.	1	.	.	.	1	.	.	.	.	.	.	.	.
Cinnagrostis chrysophylla	.	.	.	.	.	.	.	.	.	.	.	.	.	.	.	.	.	.	.	.	.	.	.	.	.	.	.	.	.
Deschampsia chrysostachya	.	.	.	.	.	.	.	.	.	.	.	.	.	.	.	.	.	.	.	.	.	.	.	.	.	.	.	.	.
Deschampsia eminens	.	.	.	.	.	.	.	.	.	.	.	.	.	1	.	.	.	1	.	.	.	.	.	.	.	.	.	.	.
Deschampsia hackelii	.	.	.	.	.	.	.	.	.	.	.	.	.	.	.	.	.	.	.	.	.	.	.	.	.	.	.	.	.
Cinnagrostis minima	.	.	.	.	.	.	.	.	.	.	.	.	.	.	.	.	.	.	.	.	.	.	.	.	.	.	.	.	.
Deschampsia ovata	.	.	.	.	.	.	.	.	.	.	.	.	.	.	.	.	1	.	.	.	.	.	.	.	.	.	.	.	.
Cinnagrostis rigescens	.	.	.	1	1	1	1	1	.	.	1	1	1	1	1	1	1	1	.	1	.	.	1	1	1	.	.	1	1
Cinnagrostis spicigera	.	.	.	.	.	.	.	1	.	.	.	.	.	.	.	.	.	.	1	.	1	1	.	.	.	.	1	1	.
Cinnagrostis velutina	1	.	.	.	.	.	.	.	.	.	.	.	.	.	.	.	.	.	.	.	.	.	.	.	.	.	.	.	.
Cinnagrostis vicunarum	.	.	.	.	.	.	.	.	.	.	.	.	.	.	.	.	.	.	1	.	.	.	.	.	.	.	.	.	.
Distichia filamentosa	.	.	.	.	.	.	.	.	.	.	.	.	.	.	.	.	.	.	.	.	.	.	.	.	.	.	.	.	.
Distichia muscoides	.	.	.	.	1	1	1	1	1	1	1	1	1	1	.	1	1	1	1	1	1	1	.	.	.	1	1	1	1
Distichlis humilis	.	.	.	.	.	.	.	.	.	.	.	.	.	.	.	.	.	.	.	.	.	.	.	.	.	.	.	.	.
Distichlis scoparia	.	.	.	.	.	.	.	.	.	.	.	.	.	.	.	.	.	.	.	.	.	.	.	.	.	.	.	.	.

Bog	117	118	119	120	121	122	123	124	125	126	127	128	129	130	131	132	133	134	135	136	137	138	139	140	141	142	143	144	145
Distichlis spicata	.	.	.	.	.	.	.	.	.	.	.	.	.	.	.	.	.	.	.	.	.	.	.	.	.	.	.	.	.
Draba pusilla	.	.	.	.	.	.	.	.	.	.	.	.	.	.	.	.	.	.	.	.	.	.	.	.	.	.	.	.	.
Eleocharis melanomphala	.	.	.	.	.	.	.	.	.	.	.	.	.	.	.	.	.	.	.	.	.	.	.	.	.	.	.	.	.
Eleocharis pseudoalbibracteata	.	.	.	.	.	.	.	.	.	.	.	.	.	.	.	.	.	.	.	.	.	.	.	.	.	.	.	.	.
Elodea potamogeton	.	.	.	.	1	.	.	.	.	.	.	.	1	.	.	.	1	.	.	.	.	.	.	.	.	.	.	.	.
Empetrum rubrum	.	1	1	.	.	.	.	.	.	.	.	.	.	.	.	.	.	.	.	.	.	.	.	.	.	.	.	.	.
Epilobium australe	.	.	.	.	.	.	.	.	.	.	.	.	.	.	.	.	.	.	.	.	.	.	.	.	.	.	.	.	.
Epilobium barbeyanum	.	.	.	.	.	.	.	.	.	.	.	.	.	.	.	.	.	.	.	.	.	.	.	.	.	.	.	.	.
Epilobium ciliatum	.	.	.	.	.	.	.	.	.	.	.	.	.	.	.	.	.	.	.	.	.	.	.	.	.	.	.	.	.
Epilobium denticulatum	.	.	.	.	.	.	.	.	.	.	.	.	.	.	.	.	.	.	.	.	.	.	1	.	.	.	.	.	.
Epilobium fragile	.	.	.	.	.	.	.	.	.	.	.	.	.	.	.	.	.	.	.	.	.	.	.	.	1	.	.	.	.
Epilobium glaucum	.	.	.	.	.	.	.	.	.	.	.	.	.	.	.	.	.	.	.	.	.	.	.	.	.	.	.	.	.
Epilobium nivale	.	.	.	.	.	.	.	.	.	.	.	.	.	.	.	.	.	.	.	.	.	.	.	.	.	.	.	.	.
Erigeron andicola	.	.	.	.	.	.	.	.	.	.	.	.	.	.	.	.	.	.	.	.	.	.	.	.	.	.	.	.	.
Erigeron leptopetalus	.	.	.	.	.	.	.	.	.	.	.	.	.	.	.	.	.	.	.	.	.	.	.	.	.	.	.	.	.
Erigeron myosotis	.	.	.	.	.	.	.	.	.	.	.	.	.	.	.	.	.	.	.	.	.	.	.	.	.	.	.	.	.
Erigeron patagonicus	.	.	.	.	.	.	.	.	.	.	.	.	.	.	.	.	.	.	.	.	.	.	.	.	.	.	.	.	.
Erythranthe cuprea	.	.	.	.	.	.	.	.	.	.	.	.	.	.	.	.	.	.	.	.	.	.	.	.	.	.	.	.	.
Erythranthe depressa	1	.	.	.	.	.	.	.	.	.	.	.	.	.	.	.	.	.	.	.	.	.	.	.	.	.	.	.	.
Erythranthe glabrata	.	.	.	1	.	1	.	.	.	.	.	.	1	.	1	1	1	.	.	.	.	.	.	.	1	.	.	.	.
Erythranthe lutea	.	.	.	.	.	.	.	.	.	.	.	.	.	.	.	.	.	.	.	.	.	.	.	.	.	.	.	.	.
Escallonia virgata	.	1	.	.	.	.	.	.	.	.	.	.	.	.	.	.	.	.	.	.	.	.	.	.	.	.	.	.	.
Euphrasia antarctica	.	.	.	.	.	.	.	.	.	.	.	.	.	.	.	.	.	.	.	.	.	.	.	.	.	.	.	.	.
Euphrasia chrysantha	.	.	.	.	.	.	.	.	.	.	.	.	.	.	.	.	.	.	.	.	.	.	.	.	.	.	.	.	.
Euphrasia subexserta	.	.	.	.	.	.	.	.	.	.	.	.	.	.	.	.	.	.	.	.	.	.	.	.	.	.	.	.	.
Festuca hypsophila	.	.	.	.	.	.	.	.	.	.	.	.	.	.	.	.	.	.	.	.	.	.	.	.	.	.	.	.	.
Festuca kurtziana	.	.	.	.	.	.	.	.	.	.	.	.	.	.	.	.	.	.	.	.	.	.	.	.	.	.	.	.	.
Festuca lilloi	.	.	.	.	.	.	.	.	.	.	.	.	.	.	.	.	.	.	.	.	.	.	.	.	.	.	.	.	.
Festuca magellanica	1	.	.	.	.	.	.	.	.	.	.	.	.	.	.	.	.	.	.	.	.	.	.	.	.	.	.	.	.
Festuca nardifolia	.	.	.	.	.	.	.	.	.	.	.	.	.	.	.	.	.	.	.	.	.	.	.	.	.	.	.	.	.
Festuca rigescens	.	.	.	.	.	.	.	.	.	.	.	.	.	.	.	.	.	.	.	.	.	.	.	.	.	.	.	.	.
Festuca werdermannii	.	.	.	.	.	.	.	.	.	.	.	.	.	.	.	.	.	.	.	.	.	.	.	.	.	.	.	.	.
Frankenia triandra	.	.	.	.	.	.	.	.	.	.	.	.	.	.	.	.	.	.	.	.	.	.	.	.	.	.	.	.	.
Gamocarpha graminea	.	.	.	.	.	.	.	.	.	.	.	.	.	.	.	.	.	.	.	.	.	.	.	.	.	.	.	.	.
Gamocarpha ventosa	.	.	.	.	.	.	.	.	.	.	.	.	.	.	.	.	.	.	.	.	.	.	.	.	.	.	.	.	.

Bog	117	118	119	120	121	122	123	124	125	126	127	128	129	130	131	132	133	134	135	136	137	138	139	140	141	142	143	144	145
Gamochaeta chamissonis	.	.	.	.	.	.	.	.	.	.	.	.	.	.	.	.	.	.	.	.	.	.	.	.	.	.	.	.	.
Gamochaeta longipedicellata	.	.	.	.	.	.	.	.	.	.	.	.	.	.	.	.	.	.	.	.	.	.	.	.	.	.	.	.	.
Gamochaeta neuquensis	.	.	.	.	.	.	.	.	.	.	.	.	.	.	.	.	.	.	.	.	.	.	.	.	.	.	.	.	.
Gaultheria antarctica	.	1	1	.	.	.	.	.	.	.	.	.	.	.	.	.	.	.	.	.	.	.	.	.	.	.	.	.	.
Gaultheria caespitosa	.	.	1	.	.	.	.	.	.	.	.	.	.	.	.	.	.	.	.	.	.	.	.	.	.	.	.	.	.
Gaultheria pumila	.	.	1	.	.	.	.	.	.	.	.	.	.	.	.	.	.	.	.	.	.	.	.	.	.	.	.	.	.
Gavilea chica	.	.	.	.	.	.	.	.	.	.	.	.	.	.	.	.	.	.	.	.	.	.	.	.	.	.	.	.	.
Gentiana prostrata	1	.	.	1	1	1	1	1	1	1	1	1	1	1	1	1	1	1	.	1	.	1	.	1	1	.	1	1	1
Gentianella fiebrigii	.	.	.	.	.	.	.	.	.	.	.	.	.	.	.	.	.	.	.	.	.	.	.	.	.	.	.	.	.
Gentianella magellanica	.	1	.	.	.	.	.	.	.	.	.	.	.	.	.	.	.	.	.	.	.	.	.	.	.	.	.	.	.
Gentianella multicaulis	.	.	.	.	.	.	.	.	.	.	.	.	.	.	.	.	.	.	.	.	.	.	.	.	.	.	.	.	.
Gentianella ottonis	.	.	.	.	.	.	.	.	.	.	.	.	.	.	.	.	.	.	.	.	.	.	.	.	.	.	.	.	.
Gentianella primuloides	.	.	.	.	.	.	.	.	.	.	.	1	.	.	.	.	.	.	.	.	.	.	.	.	.	.	.	.	.
Gentianella pseudocrassula	.	.	.	.	.	.	.	.	.	.	.	.	.	.	.	.	.	.	.	.	.	.	.	.	.	.	.	.	.
Geranium sessiliflorum	.	.	.	.	.	.	.	.	.	.	.	.	.	.	.	.	.	.	.	.	.	.	.	.	.	.	.	.	.
Gunnera magellanica	.	1	1	.	.	.	.	.	.	.	.	.	.	.	.	.	.	.	.	.	.	.	.	.	.	.	.	.	.
Halenia caespitosa	.	.	.	.	.	.	1	.	1	.	.	.	.	.	.	.	.	.	.	.	.	.	.	.	.	.	.	.	.
Halerpestes cymbalaria	1	.	.	.	.	.	1	.	.	.	.	.	.	.	.	.	.	.	.	.	.	.	.	.	.	.	.	.	.
Halerpestes exilis	.	.	.	.	.	.	.	.	.	.	.	.	.	1	.	.	.	.	.	.	.	.	.	.	.	.	.	.	.
Hieracium antarcticum	.	.	.	.	.	.	.	.	.	.	.	.	.	.	.	.	.	.	.	.	.	.	.	.	.	.	.	.	.
Hordeum comosum	.	.	.	.	.	.	.	.	.	.	.	.	.	.	.	.	.	.	.	.	.	.	.	.	.	.	.	.	.
Hordeum muticum	.	.	.	.	.	.	.	.	.	.	.	.	.	.	.	.	.	.	.	.	.	.	.	.	.	.	.	.	.
Hypochaeris acaulis	.	.	.	.	.	.	.	.	.	.	.	.	.	.	.	.	.	.	.	.	.	.	.	.	.	.	.	.	.
Hypochaeris chondrilloides	.	.	.	.	.	.	.	.	.	.	.	.	.	.	.	.	.	.	.	.	.	.	.	.	.	.	.	.	.
Hypochaeris meyeniana	.	.	.	.	.	.	.	.	.	.	.	.	.	.	.	.	.	.	.	.	.	.	.	.	.	.	.	.	.
Hypochaeris palustris	.	1	.	.	.	.	.	.	.	.	.	.	.	.	.	.	.	.	.	.	.	.	.	.	.	.	.	.	.
Hypochaeris taraxacoides	.	.	.	1	1	1	1	1	1	.	.	.	1	1	1	1	1	1	.	1	.	.	1	1	1	.	.	1	1
Hypochaeris tenerifolia	.	.	.	.	.	.	.	.	.	.	.	.	.	.	.	.	.	.	.	.	.	.	.	.	.	.	.	.	.
Isolepis nigricans	.	.	.	.	.	.	.	.	.	.	.	.	.	.	.	.	.	.	.	.	.	.	.	.	.	.	.	.	.
Isolepsis inundata	.	1	.	.	.	.	.	.	.	.	.	.	.	.	.	.	.	.	.	.	.	.	.	.	.	.	.	.	.
Juncus balticus	1	1	.	.	.	.	.	.	.	.	.	.	.	.	.	.	.	.	.	.	.	.	.	.	.	.	.	.	.

Bog	117	118	119	120	121	122	123	124	125	126	127	128	129	130	131	132	133	134	135	136	137	138	139	140	141	142	143	144	145
Juncus stipulatus	1	1	.	1	1	1	1	.	.	.	1	1	1	1	.	1	1	1	.	1	.	.	.	.	1	.	.	1	1
Koeleria kurtzii	.	.	.	.	.	.	.	.	.	.	.	.	.	.	.	.	.	.	.	.	.	.	.	.	.	.	.	.	.
Lachemilla diplophylla	.	.	.	1	1	1	1	1	.	1	1	1	1	1	1	1	.	1	.	1	.	.	1	1	1	.	.	1	1
Lachemilla pinnata	.	.	.	.	1	1	.	1	.	.	1	1	1	1	1	1	1	1	.	1	1	.	1	1	1	.	.	1	1
Lagenophora nudicaulis	.	.	.	.	.	.	.	.	.	.	.	.	.	.	.	.	.	.	.	.	.	.	.	.	.	.	.	.	.
Lemna minuta	.	.	.	1	.	.	.	.	.	.	.	.	1	.	1	.	1	.	.	.	.	.	.	.	.	.	.	.	.
Leptinella scariosa	.	.	.	.	.	.	.	.	.	.	.	.	.	.	.	.	.	.	.	.	.	.	.	.	.	.	.	.	.
Leucheria candidissima	.	.	.	.	.	.	.	.	.	.	.	.	.	.	.	.	.	.	.	.	.	.	.	.	.	.	.	.	.
Leucheria nutans	.	.	.	.	.	.	.	.	.	.	.	.	.	.	.	.	.	.	.	.	.	.	.	.	.	.	.	.	.
Lilaea scilloides	.	.	.	.	.	.	.	.	.	.	.	.	.	.	.	.	.	.	.	.	.	.	.	.	.	.	.	.	.
Lilaeopsis macloviana	.	.	.	1	1	1	1	.	.	.	1	1	1	1	1	1	1	1	.	1	.	.	.	1	1	.	.	1	1
Limosella australis	.	.	.	.	.	.	.	.	.	.	.	.	.	.	.	.	.	.	.	.	.	.	.	.	.	.	.	.	.
Lobelia oligophylla	1	.	.	1	1	1	1	.	.	.	1	1	1	1	1	1	1	1	.	1	.	.	1	.	1	.	.	1	1
Luzula brachyphylla	.	.	.	.	.	.	.	.	.	.	.	.	.	.	.	.	.	.	.	.	.	.	.	.	.	.	.	.	.
Luzula chilensis	.	.	.	.	.	.	.	.	.	.	.	.	.	.	.	.	.	.	.	.	.	.	.	.	.	.	.	.	.
Luzula racemosa	.	.	.	.	.	.	.	.	.	.	.	.	.	.	.	.	.	.	.	.	.	.	.	.	.	.	.	.	.
Luzula vulcanica	.	.	.	.	.	.	.	.	.	.	.	.	.	.	.	.	.	.	.	.	.	.	.	.	.	.	.	.	.
Lysipomia pumila	.	.	.	.	.	.	.	.	.	1	.	.	.	.	.	.	.	.	.	.	.	.	.	.	.	.	.	.	.
Marsippospermum philippii	.	1	.	.	.	.	.	.	.	.	.	.	.	.	.	.	.	.	.	.	.	.	.	.	.	.	.	.	.
Marsippospermum reichei	.	.	1	.	.	.	.	.	.	.	.	.	.	.	.	.	.	.	.	.	.	.	.	.	.	.	.	.	.
Montia fontana	.	.	.	.	.	.	.	.	.	.	.	.	1	.	.	.	.	.	.	.	.	.	.	.	.	.	.	1	1
Muhlenbergia asperifolia	.	.	.	.	.	.	.	.	.	.	.	.	.	.	.	.	.	.	.	.	.	.	.	.	.	.	.	.	.
Myriophyllum quitense	.	.	.	.	1	.	1	.	.	.	.	.	.	.	.	.	.	1	.	.	.	.	.	.	.	.	.	.	.
Myrosmodes nervosa	.	.	.	.	.	.	.	.	.	.	.	.	.	.	.	.	.	.	.	.	.	.	.	.	.	.	.	.	.
Myrosmodes paludosa	.	.	.	.	.	.	.	.	.	.	.	.	.	.	.	.	.	.	.	.	.	.	.	.	.	.	.	.	.
Myrteola nummularia	.	1	.	.	.	.	.	.	.	.	.	.	.	.	.	.	.	.	.	.	.	.	.	.	.	.	.	.	.
Nanodea muscosa	.	1	.	.	.	.	.	.	.	.	.	.	.	.	.	.	.	.	.	.	.	.	.	.	.	.	.	.	.
Neobartsia crenoloba	.	.	.	.	.	.	.	.	.	.	.	.	.	.	.	.	.	.	.	.	.	.	.	.	.	.	.	.	.
Neobartsia pedicularoides	.	.	.	.	.	.	.	1	.	.	.	.	.	.	.	.	.	.	.	.	.	.	.	.	.	.	.	.	.
Neobartsia peruviana	.	.	.	.	.	.	.	.	.	.	.	.	.	.	.	.	.	.	.	.	.	.	.	.	.	.	.	.	.
Nertera granadensis	.	1	.	.	.	.	.	.	.	.	.	.	.	.	.	.	.	.	.	.	.	.	.	.	.	.	.	.	.
Nicoraepoa andina	.	.	.	.	.	.	.	.	.	.	.	.	.	.	.	.	.	.	.	.	.	.	.	.	.	.	.	.	.

**Bog**	**117**	**118**	**119**	**120**	**121**	**122**	**123**	**124**	**125**	**126**	**127**	**128**	**129**	**130**	**131**	**132**	**133**	**134**	**135**	**136**	**137**	**138**	**139**	**140**	**141**	**142**	**143**	**144**	**145**
Nicoraepoa pugionifolia	.	.	.	.	.	.	.	.	.	.	.	.	.	.	.	.	.	.	.	.	.	.	.	.	.	.	.	.	.
Nicoraepoa subenervis	.	.	.	.	.	.	.	.	.	.	.	.	.	.	.	.	.	.	.	.	.	.	.	.	.	.	.	.	.
Nitrophila australis	.	.	.	.	.	.	.	.	.	.	.	.	.	.	.	.	.	.	.	.	.	.	.	.	.	.	.	.	.
Nothofagus antarctica	.	1	.	.	.	.	.	.	.	.	.	.	.	.	.	.	.	.	.	.	.	.	.	.	.	.	.	.	.
Nototriche rugosa	.	.	.	.	.	.	.	.	.	.	.	.	.	.	.	.	.	.	.	.	.	.	.	.	.	.	.	.	.
Ochetophila nana	1	.	.	.	.	.	.	.	.	.	.	.	.	.	.	.	.	.	.	.	.	.	.	.	.	.	.	.	.
Olsynium junceum	.	.	.	.	.	.	.	.	.	.	.	.	.	.	.	.	.	.	.	.	.	.	.	.	.	.	.	.	.
Oreobolus obtusangulus	.	.	1	.	.	.	.	.	.	.	.	.	.	.	.	.	.	.	.	.	.	.	.	.	.	.	.	.	.
Oritrophium limnophilum	.	.	.	.	.	.	1	1	.	1	.	.	.	.	.	.	.	.	.	.	.	.	.	.	.	.	.	.	.
Osmorhiza glabrata	.	.	.	.	.	.	.	.	.	.	.	.	.	.	.	.	.	.	.	.	.	.	.	.	.	.	.	.	.
Ourisia alpina	.	.	.	.	.	.	.	.	.	.	.	.	.	.	.	.	.	.	.	.	.	.	.	.	.	.	.	.	.
Ourisia muscosa	.	.	.	.	.	.	1	1	.	1	1	1	1	1	.	1	1	1	1	.	.	.	.	1	.	1	.	1	1
Ourisia ruelloides	.	.	.	.	.	.	.	.	.	.	.	.	.	.	.	.	.	.	.	.	.	.	.	.	.	.	.	.	.
Oxychloe andina	1	.	.	.	.	.	1	1	1	1	1	1	1	1	.	1	1	1	1	1	1	1	1	1	.	.	.	1	1
Oxychloe bisexualis	.	.	.	.	.	.	.	.	.	.	.	.	.	.	.	.	.	.	.	.	.	.	.	.	.	.	.	.	.
Oxychloe castellanosii	.	.	.	.	.	.	.	.	.	.	.	.	.	.	.	.	.	.	.	.	.	.	.	.	.	.	.	.	.
Oxychloe haumaniana	.	.	.	.	.	.	.	.	.	.	.	.	.	.	.	.	.	.	.	.	.	.	.	.	.	.	.	.	.
Oxychloe mendocina	.	.	.	.	.	.	.	.	.	.	.	.	.	.	.	.	.	.	.	.	.	.	.	.	.	.	.	.	.
Patosia clandestina	1	.	.	1	.	.	.	.	.	.	.	.	.	.	1	.	.	.	.	.	.	.	.	.	1	.	.	.	.
Perezia capito	.	.	.	.	.	.	.	.	.	.	.	.	.	.	.	.	.	.	.	.	.	.	.	.	.	.	.	.	.
Perezia delicata	.	.	.	.	.	.	.	.	.	.	.	.	.	.	.	.	.	.	.	.	.	.	.	.	.	.	.	.	.
Perezia fonkii	.	.	.	.	.	.	.	.	.	.	.	.	.	.	.	.	.	.	.	.	.	.	.	.	.	.	.	.	.
Perezia pedicularidifolia	.	.	1	.	.	.	.	.	.	.	.	.	.	.	.	.	.	.	.	.	.	.	.	.	.	.	.	.	.
Perezia pinnatifida	.	.	.	.	.	.	.	1	.	.	.	.	.	.	.	.	.	.	.	.	.	.	.	1	.	.	.	.	.
Petroravenia friesii	.	.	.	.	.	.	.	.	.	.	.	.	.	.	.	.	.	1	.	.	.	.	.	.	.	.	.	1	.
Petroravenia werdermannii	.	.	.	.	.	.	.	.	.	.	.	.	.	.	.	.	.	.	.	.	.	.	.	.	.	.	.	.	.
Phleum alpinum	1	.	.	.	.	.	.	.	.	.	.	.	.	.	.	.	.	.	.	.	.	.	.	.	.	.	.	.	.
Phylloscirpus acaulis	1	.	.	.	.	.	.	.	.	.	.	.	.	.	.	.	.	.	.	.	.	.	.	.	.	.	.	.	.
Phylloscirpus boliviensis	.	.	.	.	.	.	1	.	.	.	1	1	.	.	.	1	1	1	1	1	1	1	.	.	.	1	1	.	.
Phylloscirpus deserticola	.	.	.	1	1	1	1	1	1	.	.	1	1	1	1	1	1	1	.	1	1	.	1	1	1	.	.	1	1
Pinguicula antarctica	.	1	1	.	.	.	.	.	.	.	.	.	.	.	.	.	.	.	.	.	.	.	.	.	.	.	.	.	.

**Bog**	**117**	**118**	**119**	**120**	**121**	**122**	**123**	**124**	**125**	**126**	**127**	**128**	**129**	**130**	**131**	**132**	**133**	**134**	**135**	**136**	**137**	**138**	**139**	**140**	**141**	**142**	**143**	**144**	**145**
Plantago barbata	1	.	1	.	.	.	.	.	.	.	.	.	.	.	.	.	.	.	.	.	.	.	.	.	.	.	.	.	.
Plantago rigida	.	.	.	.	.	.	.	.	.	.	.	.	.	.	.	.	.	.	.	.	.	.	.	.	.	.	.	.	.
Plantago tubulosa	.	.	.	1	1	1	1	1	1	1	1	1	1	1	1	1	1	1	.	1	.	.	1	1	1	.	.	1	1
Plantago uniglumis	1	.	.	.	.	.	.	.	.	.	.	.	.	.	.	.	.	.	.	.	.	.	.	.	.	.	.	.	.
Poa alopecurus	.	.	.	.	.	.	.	.	.	.	.	.	.	.	.	.	.	.	.	.	.	.	.	.	.	.	.	.	.
Poa hachadoensis	.	.	.	.	.	.	.	.	.	.	.	.	.	.	.	.	.	.	.	.	.	.	.	.	.	.	.	.	.
Poa perligulata	.	.	.	.	.	.	.	1	.	1	.	.	1	1	.	.	.	1	1	1	1	1	.	.	.	1	1	1	1
Polypogon interruptus	.	.	.	.	.	.	.	.	.	.	.	.	.	.	.	.	.	.	.	.	.	.	.	.	.	.	.	.	.
Primula magellanica	.	1	.	.	.	.	.	.	.	.	.	.	.	.	.	.	.	.	.	.	.	.	.	.	.	.	.	.	.
Puccinellia frigida	.	.	.	.	.	.	.	.	.	.	.	.	.	.	.	.	.	.	.	.	.	.	.	.	.	.	.	.	.
Quinchamalium chilense	.	.	1	.	.	.	.	.	.	.	.	.	.	.	.	.	.	.	.	.	.	.	.	.	.	.	.	.	.
Ranunculus breviscapus	.	.	.	.	.	.	.	.	.	.	.	.	.	.	1	.	.	.	.	.	.	.	.	.	1	.	.	.	.
Ranunculus fuegianus	.	.	.	.	.	.	.	.	.	.	.	.	.	.	.	.	.	.	.	.	.	.	.	.	.	.	.	.	.
Ranunculus mandonianus	.	.	.	.	.	.	.	.	.	.	.	.	.	.	.	.	.	.	.	.	.	.	.	.	.	.	.	.	.
Ranunculus peduncularis	.	.	.	.	.	.	.	.	.	.	.	.	.	.	.	.	.	.	.	.	.	.	.	.	.	.	.	.	.
Ranunculus trichophyllus	.	.	.	.	.	.	.	.	.	.	.	.	1	.	.	.	1	.	.	1	.	.	.	.	.	.	.	1	.
Halerpestes uniflora	.	.	.	.	.	.	.	.	.	.	.	.	.	.	.	.	.	.	.	.	.	.	.	.	.	.	.	.	.
Rubus geoides	.	.	.	.	.	.	.	.	.	.	.	.	.	.	.	.	.	.	.	.	.	.	.	.	.	.	.	.	.
Rumex magellanicus	.	.	.	.	.	.	.	.	.	.	.	.	.	.	.	.	.	.	.	.	.	.	.	.	.	.	.	.	.
Rytidosperma lechleri	.	.	.	.	.	.	.	.	.	.	.	.	.	.	.	.	.	.	.	.	.	.	.	.	.	.	.	.	.
Sarcocornia pulvinata	.	.	.	.	.	.	.	.	.	.	.	.	.	.	.	.	.	.	.	.	.	.	.	.	.	.	.	.	.
Schoenoplectus pungens	.	.	.	.	.	.	.	.	.	.	.	.	.	.	.	.	.	.	.	.	.	.	.	.	.	.	.	.	.
Schoenus andinus	.	1	1	.	.	.	.	.	.	.	.	.	.	.	.	.	.	.	.	.	.	.	.	.	.	.	.	.	.
Senecio breviscapus	.	.	.	.	.	.	.	.	.	.	.	.	.	1	.	1	.	.	.	.	.	.	1	.	1	.	.	.	.
Senecio diemii	.	.	.	.	.	.	.	.	.	.	.	.	.	.	.	.	.	.	.	.	.	.	.	.	.	.	.	.	.
Senecio fistulosus	.	.	.	.	.	.	.	.	.	.	.	.	.	.	.	.	.	.	.	.	.	.	.	.	.	.	.	.	.
Senecio parodii	.	1	.	.	.	.	.	.	.	.	.	.	.	.	.	.	.	.	.	.	.	.	.	.	.	.	.	.	.
Senecio peteroanus	.	.	.	.	.	.	.	.	.	.	.	.	.	.	.	.	.	.	.	.	.	.	.	.	.	.	.	.	.
Senecio serratifolius	.	.	.	.	.	.	.	1	.	1	.	.	.	.	.	1	.	.	1	.	.	1	1	.	.	1	.	.	.
Senecio trifurcatus	.	1	1	.	.	.	.	.	.	.	.	.	.	.	.	.	.	.	.	.	.	.	.	.	.	.	.	.	.
Sisyrinchium chilense	.	.	.	.	.	.	.	.	.	.	.	.	.	.	.	.	.	.	.	.	.	.	.	.	.	.	.	.	.
Sisyrinchium patagonicum	.	1	.	.	.	.	.	.	.	.	.	.	.	.	.	.	.	.	.	.	.	.	.	.	.	.	.	.	.

**Bog**	**117**	**118**	**119**	**120**	**121**	**122**	**123**	**124**	**125**	**126**	**127**	**128**	**129**	**130**	**131**	**132**	**133**	**134**	**135**	**136**	**137**	**138**	**139**	**140**	**141**	**142**	**143**	**144**	**145**
Sisyrinchium pearcei	.	.	.	.	.	.	.	.	.	.	.	.	.	.	.	.	.	.	.	.	.	.	.	.	.	.	.	.	.
Stellaria debilis	.	.	.	.	.	.	.	.	.	.	.	.	.	.	.	.	.	.	.	.	.	.	.	.	.	.	.	.	.
Stuckenia filiformis	1	.	.	.	.	1	.	.	.	.	.	.	.	.	.	.	.	.	.	.	.	.	.	.	.	.	.	.	.
Stuckenia striata	.	.	.	.	.	.	.	.	.	.	.	.	.	.	.	.	.	.	.	.	.	.	.	.	.	.	.	.	.
Symphyotrichum peteroanum	.	.	.	.	.	.	.	.	.	.	.	.	.	.	.	.	.	.	.	.	.	.	.	.	.	.	.	.	.
Symphyotrichum vahlii	.	1	1	.	.	.	.	.	.	.	.	.	.	.	.	.	.	.	.	.	.	.	.	.	.	.	.	.	.
Tetroncium magellanicum	.	1	.	.	.	.	.	.	.	.	.	.	.	.	.	.	.	.	.	.	.	.	.	.	.	.	.	.	.
Tribeles australis	.	.	1	.	.	.	.	.	.	.	.	.	.	.	.	.	.	.	.	.	.	.	.	.	.	.	.	.	.
Trifolium amabile	.	.	.	.	.	.	.	.	.	.	.	.	.	.	.	.	.	.	.	.	.	.	.	.	.	.	.	.	.
Trifolium polymorphum	.	.	.	.	.	.	.	.	.	.	.	.	.	.	.	.	.	.	.	.	.	.	.	.	.	.	.	.	.
Triglochin concinna	.	.	.	.	.	.	.	.	.	.	.	.	.	.	.	.	.	.	.	.	.	.	.	.	.	.	.	.	.
Triglochin palustris	.	1	.	.	.	.	.	.	.	.	.	.	.	.	.	.	.	.	.	.	.	.	.	.	.	.	.	.	.
Triglochin striata	1	.	.	.	.	.	.	.	.	.	.	.	.	.	.	.	.	.	.	.	.	.	.	.	.	.	.	.	.
Trisetum caudulatum	.	.	.	.	.	.	.	.	.	.	.	.	.	.	.	.	.	.	.	.	.	.	.	.	.	.	.	.	.
Trisetum preslei	.	.	.	.	.	.	.	.	.	.	.	.	.	.	.	.	.	.	.	.	.	.	.	.	.	.	.	.	.
Koeleria spicata	.	.	.	.	.	.	.	.	.	.	.	.	.	.	.	.	.	.	.	.	.	.	.	.	.	.	.	.	.
Utricularia gibba	.	.	.	.	.	.	.	.	.	.	.	.	.	.	.	.	.	.	.	.	.	.	.	.	.	.	.	.	.
Vahlodea atropurpurea	.	.	.	.	.	.	.	.	.	.	.	.	.	.	.	.	.	.	.	.	.	.	.	.	.	.	.	.	.
Valeriana fonckii	.	.	1	.	.	.	.	.	.	.	.	.	.	.	.	.	.	.	.	.	.	.	.	.	.	.	.	.	.
Valeriana macrorhiza	.	.	.	.	.	.	.	.	.	.	.	.	.	.	.	.	.	.	.	.	.	.	.	.	.	.	.	.	.
Viola pygmaea	.	.	.	.	.	.	.	.	.	.	.	.	.	.	.	.	.	.	.	.	.	.	.	.	.	.	.	.	.
Werneria apiculata	.	.	.	.	.	.	.	.	.	.	.	.	.	.	.	.	.	.	.	.	.	.	.	.	.	.	.	.	.
Werneria pinnatifida	.	.	.	1	1	1	.	1	.	1	1	1	.	1	1	.	1	1	1	.	.	1	1	1	1	.	1	1	1
Werneria pygmaea	1	.	.	1	1	1	1	1	1	1	1	1	1	1	1	1	1	1	1	.	1	1	1	1	1	1	.	1	.
Werneria solivifolia	.	.	.	.	.	.	1	1	.	.	.	.	1	1	.	1	1	1	.	.	.	.	.	.	.	.	.	1	.
Werneria spathulata	.	.	.	.	.	.	1	1	1	1	1	1	.	.	.	.	.	.	.	.	.	1	1	.	1	.	.	.	.
Xenophyllum incisum	.	.	.	.	.	.	.	.	.	.	.	.	.	.	.	.	.	.	.	.	.	.	.	.	.	.	.	.	.
Zameioscirpus atacamensis	.	.	.	.	.	.	.	.	.	.	.	.	.	.	.	.	.	.	.	.	.	.	.	.	.	.	.	.	.
Zameioscirpus gaimardiodes	.	.	.	.	.	.	.	.	.	.	.	.	.	.	.	.	.	.	.	.	.	.	.	.	.	.	.	.	.
Zameioscirpus muticus	.	.	.	.	1	1	1	1	.	1	.	.	1	1	.	1	1	1	1	1	1	1	1	.	1	1	1	1	1

Bog	146	147	148	149	150	151	152	153	154	155	156	157	158	159	160	161	162	163	164	165	166	167	168	169	170	171	172	173	174
**Operational zone**	N	N	T	T	T	T	T	T	T	T	T	T	T	T	T	T	T	T	T	T	T	T	T	T	T	T	S	S	S
**Cluster**	1	1	2	4	4	4	2	2	2	2	1	1	4	2	4	1	4	4	4	2	2	2	2	2	2	4	7	7	7
**Bioregion**	N	N	T	T	T	T	T	T	T	T	T	T	T	T	T	T	T	T	T	T	T	T	N	T	T	T	S	S	S
**Longitude**	-67.18	-69.22	-67.71	-68.84	-68.84	-68.85	-68.84	-68.84	-68.85	-68.85	-68.99	-68.82	-68.82	-67.71	-68.74	-69.23	-69.11	-69.13	-68.99	-67.7	-67.69	-67.7	-67.7	-67.72	-67.78	-68.79	-71.19	-71.04	-70.99
**Latitude**	-17.04	-17.29	-28.98	-28.48	-28.48	-28.45	-28.48	-28.48	-28.44	-28.44	-28.59	-28.19	-28.28	-28.98	-28.29	-28.09	-28.19	-28.39	-28.72	-28.97	-28.97	-28.98	-28.98	-28.98	-28.88	-28.51	-38.4	-38.63	-38.63
Acaena antarctica	.	.	.	.	.	.	.	.	.	.	.	.	.	.	.	.	.	.	.	.	.	.	.	.	.	.	.	.	.
Acaena macrocephala	.	.	.	.	.	.	.	.	.	.	.	.	.	.	.	.	.	.	.	.	.	.	.	.	.	.	.	.	.
Acaena magellanica	.	.	.	.	.	.	.	.	.	.	.	.	.	.	.	.	.	.	.	.	.	.	.	.	1	1	.	.	.
Acaena ovalifolia	.	.	.	.	.	.	.	.	.	.	.	.	.	.	.	.	.	.	.	.	.	.	.	.	.	.	.	.	1
Acaena pinnatifida	.	.	.	.	.	.	.	.	.	.	.	.	.	.	.	.	.	.	.	.	.	.	.	.	.	.	.	.	.
Adesmia retusa	.	.	.	.	.	.	.	.	.	.	.	.	.	.	.	.	.	.	.	.	.	.	.	.	.	.	.	.	.
Agrostis breviculmis	.	.	.	.	.	.	.	.	.	.	.	.	.	.	.	.	.	.	.	.	.	.	.	.	.	.	.	.	.
Agrostis imberbis	.	.	.	.	.	.	.	.	.	.	.	.	.	.	.	.	.	.	.	.	.	.	.	.	.	.	.	.	.
Agrostis meyenii	.	.	.	.	.	.	.	.	.	.	.	.	.	.	.	.	.	.	.	.	.	.	.	.	.	.	1	1	1
Agrostis perennans	.	.	.	.	.	.	.	.	.	.	.	.	.	.	.	.	.	.	.	.	.	.	.	.	.	.	.	.	.
Alchemilla pinnata	.	.	.	.	.	.	.	.	.	.	.	.	.	.	.	.	.	.	.	.	.	.	.	.	.	.	.	.	.
Alopecurus magellanicus	.	.	.	.	.	.	.	.	.	.	.	.	.	.	.	.	.	.	.	.	.	.	.	.	.	.	.	.	1
Amphiscirpus nevadensis	.	.	.	.	.	.	.	.	.	.	.	.	.	.	.	.	.	.	.	.	.	.	.	.	.	.	.	.	.
Anagallis alternifolia	.	.	.	.	.	.	.	.	.	.	.	.	.	.	.	.	.	.	.	.	.	.	.	.	1	.	.	1	1
Antennaria chilensis	.	.	.	.	.	.	.	.	.	.	.	.	.	.	.	.	.	.	.	.	.	.	.	.	.	.	.	.	.
Anthoxanthum redolens	.	.	.	.	.	.	.	.	.	.	.	.	.	.	.	.	.	.	.	.	.	.	.	.	.	.	.	.	.
Apium panul	.	.	.	.	.	.	.	.	.	.	.	.	.	.	.	.	.	.	.	.	.	.	.	.	.	.	.	.	.
Arenaria rivularis	.	.	.	.	.	.	.	.	.	.	.	.	.	.	.	.	.	.	.	.	.	.	.	.	.	.	.	.	.
Arenaria serpens	.	.	.	.	.	.	.	.	.	.	.	.	.	.	.	.	.	.	.	.	.	.	.	.	.	.	.	.	.
Arjona pusilla	.	.	.	.	.	.	.	.	.	.	.	.	.	.	.	.	.	.	.	.	.	.	.	.	.	.	.	.	.
Astragalus bustillosii	.	.	.	.	.	.	.	.	.	.	.	.	.	.	.	.	.	.	.	.	.	.	.	.	.	.	.	.	.
Astragalus micranthellus	.	.	.	.	.	.	.	.	.	.	.	.	.	.	.	.	.	.	.	.	.	.	.	.	.	.	.	.	.
Azolla filiculoides	.	.	.	.	.	.	.	.	.	.	.	.	.	.	.	.	.	.	.	.	.	.	.	.	.	.	.	.	.
Azorella boelckei	.	.	.	.	.	.	.	.	.	.	.	.	.	.	.	.	.	.	.	.	.	.	.	.	.	.	.	.	.

Bog	146	147	148	149	150	151	152	153	154	155	156	157	158	159	160	161	162	163	164	165	166	167	168	169	170	171	172	173	174
Azorella burkartii	.	.	.	.	.	.	.	.	.	.	.	.	.	.	.	.	.	.	.	.	.	.	.	.	.	.	.	.	1
Azorella cryptantha	.	.	.	.	.	.	.	.	.	.	.	.	.	.	.	.	.	.	.	.	.	.	.	.	.	.	.	.	.
Azorella lycopodioides	.	.	.	.	.	.	.	.	.	.	.	.	.	.	.	.	.	.	.	.	.	.	.	.	.	.	1	.	.
Azorella trifoliolata	.	.	.	.	.	.	.	.	.	.	.	.	.	.	.	.	.	.	.	.	.	.	.	.	.	.	.	.	.
Baccharis acaulis	.	.	.	.	.	.	.	.	.	.	.	.	.	.	.	.	.	.	.	.	.	.	.	.	.	.	.	.	.
Baccharis caespitosa	.	.	.	.	.	.	.	.	.	.	.	.	.	.	.	.	.	.	.	.	.	.	.	.	.	.	.	.	.
Baccharis magellanica	.	.	.	.	.	.	.	.	.	.	.	.	.	.	.	.	.	.	.	.	.	.	.	.	.	.	.	.	.
Belloa chilensis	.	.	.	.	.	.	.	.	.	.	.	.	.	.	.	.	.	.	.	.	.	.	.	.	.	.	.	.	.
Bromus catharticus	.	.	1	.	.	.	.	.	.	.	.	.	.	1	.	.	.	.	.	1	.	1	.	1	.	.	.	.	.
Calandrinia acaulis	.	.	.	.	.	.	.	.	.	.	.	.	.	.	.	.	.	.	.	.	.	.	.	.	.	.	.	.	.
Calandrinia compacta	.	1	.	.	.	.	.	.	.	.	1	1	1	.	1	1	1	.	.	.	.	.	.	.	.	.	.	.	.
Calceolaria biflora	.	.	.	.	.	.	.	.	.	.	.	.	.	.	.	.	.	.	.	.	.	.	.	.	.	.	.	.	.
Calceolaria cana	.	.	.	.	.	.	.	.	.	.	.	.	.	.	.	.	.	.	.	.	.	.	.	.	.	.	.	.	1
Calceolaria corymbosa	.	.	.	.	.	.	.	.	.	.	.	.	.	.	.	.	.	.	.	.	.	.	.	.	.	.	.	.	.
Calceolaria filicaulis	.	.	.	.	.	.	.	.	.	.	.	.	.	.	.	.	.	.	.	.	.	.	.	.	.	.	.	.	.
Callitriche lechleri	.	.	.	.	.	.	.	.	.	.	.	.	.	.	.	.	.	.	.	.	.	.	.	.	.	.	.	.	.
Caltha appendiculata	.	.	.	.	.	.	.	.	.	.	.	.	.	.	.	.	.	.	.	.	.	.	.	.	.	.	.	.	1
Caltha sagittata	.	.	.	.	.	.	.	.	.	.	.	.	.	.	.	.	.	.	.	.	.	.	.	.	.	.	.	1	1
Cardamine cordata	.	.	.	.	.	.	.	.	.	.	.	.	.	.	.	.	.	.	.	.	.	.	.	.	.	.	.	.	.
Cardamine glacialis	.	.	.	.	.	.	.	.	.	.	.	.	.	.	.	.	.	.	.	.	.	.	.	.	.	.	.	.	.
Cardamine tenuirostris	.	.	.	.	.	.	.	.	.	.	.	.	.	.	.	.	.	.	.	.	.	.	.	.	.	.	.	.	.
Cardamine volckmannii	.	.	.	.	.	.	.	.	.	.	.	.	.	.	.	.	.	.	.	.	.	.	.	.	.	.	.	.	.
Carex acaulis	.	.	.	.	.	.	.	.	.	.	.	.	.	.	.	.	.	.	.	.	.	.	.	.	.	.	.	.	.
Carex atropicta	.	.	.	.	.	.	.	.	.	.	.	.	.	.	.	.	.	.	.	.	.	.	.	.	.	.	.	.	1
Carex banksii	.	.	.	.	.	.	.	.	.	.	.	.	.	.	.	.	.	.	.	.	.	.	.	.	.	.	1	1	1
Carex caduca	.	.	.	.	.	.	.	.	.	.	.	.	.	.	.	.	.	.	.	.	.	.	.	.	.	.	.	.	.
Carex decidua	.	.	.	.	.	.	.	.	.	.	.	.	.	.	.	.	.	.	.	.	.	.	.	.	.	.	.	.	.
Carex fuscula	.	.	.	.	.	.	.	.	.	.	.	.	.	.	.	.	.	.	.	.	.	.	.	.	.	.	.	1	1
Carex gayana	.	.	1	.	1	.	.	.	.	.	.	.	.	1	.	.	1	.	.	1	1	1	1	.	1	.	.	1	1
Carex hypoleucos	.	.	.	.	.	.	.	.	.	.	.	.	.	.	.	.	.	.	.	.	.	.	.	.	.	.	.	1	.
Carex macloviana	.	.	.	.	.	.	.	.	.	.	.	.	.	.	.	.	.	.	.	.	.	.	.	.	.	.	1	.	.
Carex magellanica	.	.	.	.	.	.	.	.	.	.	.	.	.	.	.	.	.	.	.	.	.	.	.	.	.	.	.	1	1
Carex malmei	.	.	.	.	.	.	.	.	.	.	.	.	.	.	.	.	.	.	.	.	.	.	.	.	.	.	.	.	.
Carex maritima	1	1	.	1	.	.	1	1	1	1	1	1	1	.	1	1	1	1	1	.	.	.	.	.	.	.	.	.	.
Carex microglochin	.	.	1	.	.	.	1	1	1	1	.	.	.	1	.	.	.	.	.	.	1	1	1	.	1	.	.	.	.
Carex pleioneura	.	.	.	.	.	.	.	.	.	.	.	.	.	.	.	.	.	.	.	.	.	.	.	.	.	.	.	.	.
Carex ruthsatzae	.	.	.	.	.	.	.	.	.	.	.	.	.	.	.	.	.	.	.	.	.	.	.	.	.	.	.	.	.
Carex vallis-pulchrae	.	.	1	1	.	.	1	1	.	.	.	.	.	1	.	.	.	.	.	.	.	.	.	1	.	.	.	.	.

Bog	146	147	148	149	150	151	152	153	154	155	156	157	158	159	160	161	162	163	164	165	166	167	168	169	170	171	172	173	174
Carpha schoenoides	.	.	.	.	.	.	.	.	.	.	.	.	.	.	.	.	.	.	.	.	.	.	.	.	.	.	.	.	.
Castilleja pumila	1	.	.	.	.	.	.	.	.	.	.	.	.	.	.	.	.	.	.	.	.	.	.	.	.	.	.	.	.
Catabrosa werdermannii	.	.	.	.	.	.	.	.	.	.	.	.	1	.	.	.	1	.	.	.	.	.	.	.	.	.	.	.	.
Cerastium humifusum	.	.	.	.	.	.	.	.	.	.	.	.	.	.	.	.	.	.	.	.	.	.	.	.	.	.	.	.	.
Cerastium montioides	.	.	.	.	.	.	.	.	.	.	.	.	.	.	.	.	.	.	.	.	.	.	.	.	.	.	.	.	.
Chiliotrichum diffusum	.	.	.	.	.	.	.	.	.	.	.	.	.	.	.	.	.	.	.	.	.	.	.	.	.	.	1	1	1
Chusquea culeou	.	.	.	.	.	.	.	.	.	.	.	.	.	.	.	.	.	.	.	.	.	.	.	.	.	.	.	.	.
Colobanthus quitensis	1	1	1	1	.	.	1	1	1	1	.	.	.	.	.	.	.	.	.	1	1	.	1	1	.	.	.	1	1
Cortaderia egmontiana	.	.	.	.	.	.	.	.	.	.	.	.	.	.	.	.	.	.	.	.	.	.	.	.	.	.	1	1	1
Cotula mexicana	.	1	1	.	.	.	.	.	.	.	.	.	.	1	.	.	.	.	.	1	1	1	.	1	1	.	.	.	.
Crassula peduncularis	.	.	.	.	.	.	.	.	.	.	.	.	.	.	.	.	.	.	.	.	.	.	.	.	.	.	.	.	.
Cuatrecasasiella argentina	.	.	1	.	.	.	.	.	.	.	.	.	.	.	.	.	.	.	.	.	1	1	1	1	1	.	.	.	.
Deschampsia antarctica	.	.	.	.	.	.	.	.	.	.	.	.	.	.	.	.	.	.	.	.	.	.	.	.	.	.	.	.	.
Deschampsia caespitosa	.	.	.	.	.	.	.	.	.	.	.	.	.	.	.	.	.	.	.	.	.	.	.	.	.	.	.	.	.
Deschampsia patula	.	.	.	.	.	.	.	.	.	.	.	.	.	.	.	.	.	.	.	.	.	.	.	.	.	.	.	.	.
Cinnagrostis brevifolia	.	.	.	.	.	.	.	.	.	.	.	.	.	.	.	.	.	.	.	.	.	.	.	.	.	.	.	.	.
Deschampsia chrysantha	.	.	.	.	.	.	.	.	.	.	.	.	.	.	.	.	.	.	.	.	.	.	.	.	.	.	.	.	.
Cinnagrostis chrysophylla	.	.	.	.	.	.	.	.	.	.	.	.	.	.	.	.	.	.	.	.	.	.	.	.	.	.	.	.	.
Deschampsia chrysostachya	.	.	.	.	.	.	.	.	.	.	.	.	.	.	.	.	.	.	.	.	.	.	.	.	.	.	.	.	.
Deschampsia eminens	.	.	1	.	1	1	1	1	1	1	.	1	.	1	.	1	1	.	.	.	.	.	1	1	1	.	.	.	.
Deschampsia hackelii	.	.	.	.	.	.	.	.	.	.	.	.	.	.	.	.	.	.	.	.	.	.	.	.	.	.	.	.	.
Cinnagrostis minima	.	.	.	.	.	.	.	.	.	.	.	.	.	.	.	.	.	.	.	.	.	.	.	.	.	.	.	.	.
Deschampsia ovata	.	.	.	.	.	.	.	.	.	.	.	.	.	.	.	.	.	.	.	.	.	.	.	.	.	.	.	.	.
Cinnagrostis rigescens	1	1	1	.	.	.	.	.	.	.	.	.	.	1	.	.	.	.	.	1	1	1	1	1	1	.	.	.	.
Cinnagrostis spicigera	.	.	.	.	.	.	.	.	.	.	.	.	.	.	.	.	.	.	.	.	.	.	.	.	.	.	.	.	.
Cinnagrostis velutina	.	.	.	.	.	.	.	.	.	.	.	.	.	.	.	.	.	.	.	.	.	.	.	.	.	.	.	.	.
Cinnagrostis vicunarum	.	.	.	.	.	.	.	.	.	.	.	.	.	.	.	.	.	.	.	.	.	.	.	.	.	.	.	.	.
Distichia filamentosa	.	.	.	.	.	.	.	.	.	.	.	.	.	.	.	.	.	.	.	.	.	.	.	.	.	.	.	.	.
Distichia muscoides	1	1	.	.	.	.	.	.	.	.	.	.	.	.	.	.	.	.	.	.	.	.	.	.	.	.	.	.	.
Distichlis humilis	.	.	.	.	.	.	.	.	.	.	.	.	.	.	.	.	.	.	.	.	.	.	.	.	.	.	.	.	.
Distichlis scoparia	.	.	.	.	.	.	.	.	.	.	.	.	.	.	.	.	.	.	.	.	.	.	.	.	.	.	.	.	.
Distichlis spicata	.	.	.	.	.	.	1	.	.	.	.	.	.	.	.	.	.	.	.	.	.	.	.	.	.	1	.	.	.

Bog	146	147	148	149	150	151	152	153	154	155	156	157	158	159	160	161	162	163	164	165	166	167	168	169	170	171	172	173	174
Draba pusilla	.	.	.	.	.	.	.	.	.	.	.	.	.	.	.	.	.	.	.	.	.	.	.	.	.	.	.	.	.
Eleocharis melanomphala	.	.	.	.	.	.	.	.	.	.	.	.	.	.	.	.	.	.	.	.	.	.	.	.	.	.	.	.	.
Eleocharis pseudoalbibracteata	.	.	1	1	1	.	1	1	1	1	.	1	.	1	.	.	.	.	.	1	1	.	.	1	.	.	.	.	.
Elodea potamogeton	.	.	.	.	.	.	.	.	.	.	.	.	.	.	.	.	.	.	.	.	.	.	.	.	.	.	.	.	.
Empetrum rubrum	.	.	.	.	.	.	.	.	.	.	.	.	.	.	.	.	.	.	.	.	.	.	.	.	.	.	1	.	1
Epilobium australe	.	.	.	.	.	.	.	.	.	.	.	.	.	.	.	.	.	.	.	.	.	.	.	.	.	.	.	.	.
Epilobium barbeyanum	.	.	.	.	.	.	.	.	.	.	.	.	.	.	.	.	.	.	.	.	.	.	.	.	.	.	.	.	.
Epilobium ciliatum	.	.	.	.	.	.	.	.	.	.	.	.	.	.	.	.	.	.	.	.	.	.	.	.	.	.	.	1	1
Epilobium denticulatum	.	.	.	.	.	.	.	.	.	.	.	.	.	.	.	.	.	.	.	.	.	.	.	.	.	.	.	.	.
Epilobium fragile	.	.	.	.	.	.	.	.	.	.	.	.	.	.	.	.	.	.	.	.	.	.	.	.	.	.	.	.	.
Epilobium glaucum	.	.	.	.	.	.	.	.	.	.	.	.	.	.	.	.	.	.	.	.	.	.	.	.	.	.	.	.	.
Epilobium nivale	.	.	.	.	.	.	.	.	.	.	.	.	.	.	.	.	.	.	.	.	.	.	.	.	.	.	.	.	1
Erigeron andicola	.	.	.	.	.	.	.	.	.	.	.	.	.	.	.	.	.	.	.	.	.	.	.	.	.	.	.	.	.
Erigeron leptopetalus	.	.	.	.	.	.	.	.	.	.	.	.	.	.	.	.	.	.	.	.	.	.	.	.	.	.	.	.	.
Erigeron myosotis	.	.	.	.	.	.	.	.	.	.	.	.	.	.	.	.	.	.	.	.	.	.	.	.	.	.	.	1	1
Erigeron patagonicus	.	.	.	.	.	.	.	.	.	.	.	.	.	.	.	.	.	.	.	.	.	.	.	.	.	.	.	.	.
Erythranthe cuprea	.	.	.	.	.	.	.	.	.	.	.	.	.	.	.	.	.	.	.	.	.	.	.	.	.	.	.	.	.
Erythranthe depressa	.	.	1	.	.	.	.	1	.	.	.	.	.	.	.	.	1	.	.	.	1	.	1	.	.	.	.	.	.
Erythranthe glabrata	.	.	.	.	.	.	.	.	.	.	.	.	.	.	.	.	.	.	.	.	.	.	.	.	.	.	.	.	.
Erythranthe lutea	.	.	.	.	.	.	.	.	.	.	.	.	.	.	.	.	.	.	.	.	.	.	.	.	.	.	.	1	.
Escallonia virgata	.	.	.	.	.	.	.	.	.	.	.	.	.	.	.	.	.	.	.	.	.	.	.	.	.	.	1	1	1
Euphrasia antarctica	.	.	.	.	.	.	.	.	.	.	.	.	.	.	.	.	.	.	.	.	.	.	.	.	.	.	.	.	.
Euphrasia chrysantha	.	.	.	.	.	.	.	.	.	.	.	.	.	.	.	.	.	.	.	.	.	.	.	.	.	.	.	.	.
Euphrasia subexserta	.	.	.	.	.	.	.	.	.	.	.	.	.	.	.	.	.	.	.	.	.	.	.	.	.	.	1	1	1
Festuca hypsophila	.	.	.	.	.	.	.	.	.	.	.	.	.	.	.	.	.	.	.	.	.	.	.	.	.	.	.	.	.
Festuca kurtziana	.	.	.	.	.	.	.	.	.	.	.	.	.	.	.	.	.	.	.	.	.	.	.	.	.	.	.	.	.
Festuca lilloi	.	.	.	.	.	.	.	.	.	.	.	.	.	.	.	.	.	.	.	.	.	.	.	.	.	.	.	.	.
Festuca magellanica	.	.	.	.	.	.	.	.	.	.	.	.	.	.	.	.	.	.	.	.	.	.	.	.	.	.	.	.	1
Festuca nardifolia	.	.	.	.	.	.	.	.	.	.	.	.	.	.	.	.	.	.	.	.	.	.	.	.	.	.	.	.	.
Festuca rigescens	.	.	.	.	.	.	.	.	.	.	.	.	.	.	.	.	.	.	.	.	.	.	.	.	.	.	.	.	.
Festuca werdermannii	.	.	.	.	.	.	.	.	.	.	.	.	.	.	.	.	.	.	.	.	.	.	.	.	.	.	.	.	.
Frankenia triandra	.	.	.	.	.	.	.	.	.	.	.	.	.	.	.	.	.	.	.	.	.	.	.	.	.	.	.	.	.
Gamocarpha graminea	.	.	.	.	.	.	.	.	.	.	.	.	.	.	.	.	.	.	.	.	.	.	.	.	.	.	.	.	1
Gamocarpha ventosa	.	.	.	.	.	.	.	.	.	.	.	.	.	.	.	.	.	.	.	.	.	.	.	.	.	.	.	.	.
Gamochaeta chamissonis	.	.	.	.	.	.	.	.	.	.	.	.	.	.	.	.	.	.	.	.	.	.	.	.	.	.	.	.	.

Bog	146	147	148	149	150	151	152	153	154	155	156	157	158	159	160	161	162	163	164	165	166	167	168	169	170	171	172	173	174
Gamochaeta longipedicellata	.	.	.	.	.	.	.	.	.	.	.	.	.	.	.	.	.	.	.	.	.	.	.	.	.	.	.	.	.
Gamochaeta neuquensis	.	.	.	.	.	.	.	.	.	.	.	.	.	.	.	.	.	.	.	.	.	.	.	.	.	.	.	.	.
Gaultheria antarctica	.	.	.	.	.	.	.	.	.	.	.	.	.	.	.	.	.	.	.	.	.	.	.	.	.	.	.	.	.
Gaultheria caespitosa	.	.	.	.	.	.	.	.	.	.	.	.	.	.	.	.	.	.	.	.	.	.	.	.	.	.	.	.	1
Gaultheria pumila	.	.	.	.	.	.	.	.	.	.	.	.	.	.	.	.	.	.	.	.	.	.	.	.	.	.	1	1	1
Gavilea chica	.	.	.	.	.	.	.	.	.	.	.	.	.	.	.	.	.	.	.	.	.	.	.	.	.	.	.	.	.
Gentiana prostrata	1	1	1	.	.	.	1	1	1	1	.	.	.	1	.	.	.	.	.	1	1	1	1	1	1	.	.	1	1
Gentianella fiebrigii	.	.	.	.	.	.	.	.	.	.	.	.	.	.	.	.	.	.	.	.	.	.	.	.	.	.	.	.	.
Gentianella magellanica	.	.	.	.	.	.	.	.	.	.	.	.	.	.	.	.	.	.	.	.	.	.	.	.	.	.	1	1	1
Gentianella multicaulis	.	.	.	.	.	.	.	.	.	.	.	.	.	.	.	.	.	.	.	.	.	.	.	.	.	.	.	.	.
Gentianella ottonis	.	.	.	.	.	.	.	.	.	.	.	.	.	.	.	.	.	.	.	.	.	.	.	.	.	.	.	.	.
Gentianella primuloides	1	.	.	.	.	.	.	.	.	.	.	.	.	.	.	.	.	.	.	.	.	.	.	.	.	.	.	.	.
Gentianella pseudocrassula	.	.	.	.	.	.	.	.	.	.	.	.	.	.	.	.	.	.	.	.	.	.	.	.	.	.	.	.	.
Geranium sessiliflorum	.	.	1	.	.	.	.	.	.	.	.	.	.	.	.	.	.	.	.	.	1	1	1	1	.	.	.	.	.
Gunnera magellanica	.	.	.	.	.	.	.	.	.	.	.	.	.	.	.	.	.	.	.	.	.	.	.	.	.	.	1	1	1
Halenia caespitosa	.	.	.	.	.	.	.	.	.	.	.	.	.	.	.	.	.	.	.	.	.	.	.	.	.	.	.	.	.
Halerpestes cymbalaria	.	.	1	1	1	.	.	1	.	.	.	.	.	.	.	.	.	1	.	1	.	1	.	1	.	1	.	.	.
Halerpestes exilis	.	.	.	1	.	1	.	.	.	.	.	1	1	.	.	.	1	1	.	.	.	.	.	.	.	.	.	.	.
Hieracium antarcticum	.	.	.	.	.	.	.	.	.	.	.	.	.	.	.	.	.	.	.	.	.	.	.	.	.	.	.	.	.
Hordeum comosum	.	.	.	.	.	.	.	.	.	.	.	.	.	.	.	.	.	.	.	.	.	.	.	.	.	.	.	.	.
Hordeum muticum	.	.	1	1	1	1	1	1	1	1	.	.	.	.	.	.	.	.	.	1	1	.	.	.	.	1	.	.	.
Hypochaeris acaulis	.	.	.	.	.	.	.	.	.	.	.	.	.	.	.	.	.	.	.	.	.	.	.	.	.	.	.	.	1
Hypochaeris chondrilloides	.	.	.	.	.	.	.	.	.	.	.	.	.	.	.	.	.	.	.	.	.	.	.	.	.	.	.	.	.
Hypochaeris meyeniana	.	.	.	.	.	.	.	.	.	.	.	.	.	.	.	.	.	.	.	.	.	.	.	.	.	.	.	.	.
Hypochaeris palustris	.	.	.	.	.	.	.	.	.	.	.	.	.	.	.	.	.	.	.	.	.	.	.	.	.	.	.	.	.
Hypochaeris taraxacoides	1	.	.	.	.	.	.	.	.	.	.	.	.	.	.	.	.	.	.	.	.	.	.	.	.	.	.	.	.
Hypochaeris tenerifolia	.	.	.	.	.	.	.	.	.	.	.	.	.	.	.	.	.	.	.	.	.	.	.	.	.	.	.	.	1
Isolepis nigricans	.	.	.	.	.	.	.	.	.	.	.	.	.	.	.	.	.	.	.	.	.	.	.	.	.	.	.	.	.
Isolepsis inundata	.	.	.	.	.	.	.	.	.	.	.	.	.	.	.	.	.	.	.	.	.	.	.	.	.	.	.	1	1
Juncus balticus	.	.	1	1	1	1	1	1	1	1	1	.	.	.	.	.	.	1	1	1	.	.	.	.	1	1	1	1	1
Juncus stipulatus	.	1	1	.	.	.	.	1	.	.	.	.	.	1	.	.	.	1	.	1	1	1	1	1	1	.	1	1	1
Koeleria kurtzii	.	.	.	.	.	.	.	.	.	.	.	.	.	.	.	.	.	.	.	.	.	.	.	.	.	.	.	.	.

Bog	146	147	148	149	150	151	152	153	154	155	156	157	158	159	160	161	162	163	164	165	166	167	168	169	170	171	172	173	174
Lachemilla diplophylla	1	1	.	.	.	.	.	.	.	.	.	.	.	.	.	.	.	.	.	.	.	.	.	.	.	.	.	.	.
Lachemilla pinnata	.	1	1	.	.	.	.	.	.	.	.	.	.	1	.	.	.	.	.	1	1	1	1	1	.	.	.	.	.
Lagenophora nudicaulis	.	.	.	.	.	.	.	.	.	.	.	.	.	.	.	.	.	.	.	.	.	.	.	.	.	.	.	.	.
Lemna minuta	.	.	.	.	.	.	.	.	.	.	.	.	.	.	.	.	.	.	.	.	.	.	.	.	.	.	.	.	.
Leptinella scariosa	.	.	.	.	.	.	.	.	.	.	.	.	.	.	.	.	.	.	.	.	.	.	.	.	.	.	.	.	.
Leucheria candidissima	.	.	.	.	.	.	.	.	.	.	.	.	.	.	.	.	.	.	.	.	.	.	.	.	.	.	.	.	.
Leucheria nutans	.	.	.	.	.	.	.	.	.	.	.	.	.	.	.	.	.	.	.	.	.	.	.	.	.	.	.	.	.
Lilaea scilloides	.	.	.	.	.	.	.	.	.	.	.	.	.	.	.	.	.	.	.	.	.	.	.	.	.	.	.	.	.
Lilaeopsis macloviana	.	1	1	1	1	.	.	1	1	.	.	.	.	.	.	.	.	.	.	1	.	.	.	.	.	1	.	.	.
Limosella australis	.	.	.	.	.	.	.	.	.	.	.	.	.	.	.	.	.	.	.	.	.	.	.	.	.	.	.	.	.
Lobelia oligophylla	1	1	.	1	1	.	1	1	1	.	.	.	.	.	.	.	.	.	.	1	.	.	.	1	.	1	.	1	1
Luzula brachyphylla	.	.	.	.	.	.	.	.	.	.	.	.	.	.	.	.	.	.	.	.	.	.	.	.	.	.	.	.	.
Luzula chilensis	.	.	.	.	.	.	.	.	.	.	.	.	.	.	.	.	.	.	.	.	.	.	.	.	.	.	1	1	1
Luzula racemosa	.	.	1	.	.	.	.	.	.	.	.	.	.	1	.	.	.	.	.	1	1	.	1	.	.	.	.	.	.
Luzula vulcanica	.	.	.	.	.	.	.	.	.	.	.	.	.	.	.	.	.	.	.	.	.	.	.	.	.	.	.	.	.
Lysipomia pumila	.	.	.	.	.	.	.	.	.	.	.	.	.	.	.	.	.	.	.	.	.	.	.	.	.	.	.	.	.
Marsippospermum philippii	.	.	.	.	.	.	.	.	.	.	.	.	.	.	.	.	.	.	.	.	.	.	.	.	.	.	.	.	1
Marsippospermum reichei	.	.	.	.	.	.	.	.	.	.	.	.	.	.	.	.	.	.	.	.	.	.	.	.	.	.	.	.	.
Montia fontana	.	.	.	.	.	.	.	.	.	.	.	.	.	.	.	.	.	.	.	.	.	.	.	.	.	.	.	.	.
Muhlenbergia asperifolia	.	.	.	.	.	.	.	.	.	.	.	.	.	.	.	.	.	.	.	.	.	.	.	.	.	.	.	.	.
Myriophyllum quitense	.	.	.	.	.	.	.	.	.	.	.	.	.	.	.	.	.	.	.	.	.	.	.	.	.	.	.	.	.
Myrosmodes nervosa	.	.	.	.	.	.	.	.	.	.	.	.	.	.	.	.	.	.	.	.	.	.	.	.	.	.	.	.	.
Myrosmodes paludosa	.	.	1	.	.	.	.	.	.	.	.	.	.	1	.	.	.	.	.	1	1	1	1	1	.	.	.	.	.
Myrteola nummularia	.	.	.	.	.	.	.	.	.	.	.	.	.	.	.	.	.	.	.	.	.	.	.	.	.	.	.	.	.
Nanodea muscosa	.	.	.	.	.	.	.	.	.	.	.	.	.	.	.	.	.	.	.	.	.	.	.	.	.	.	.	.	.
Neobartsia crenoloba	.	.	1	.	.	.	.	.	.	.	.	.	.	1	.	.	.	.	.	.	.	1	1	.	.	.	.	.	.
Neobartsia pedicularoides	.	.	.	.	.	.	.	.	.	.	.	.	.	.	.	.	.	.	.	.	.	.	.	.	.	.	.	.	.
Neobartsia peruviana	.	.	.	.	.	.	.	.	.	.	.	.	.	.	.	.	.	.	.	.	.	.	.	.	.	.	.	.	.
Nertera granadensis	.	.	.	.	.	.	.	.	.	.	.	.	.	.	.	.	.	.	.	.	.	.	.	.	.	.	.	.	.
Nicoraepoa andina	.	.	.	.	.	.	.	.	.	.	.	.	.	.	.	.	.	.	.	.	.	.	.	.	.	.	.	1	1
Nicoraepoa pugionifolia	.	.	.	.	.	.	.	.	.	.	.	.	.	.	.	.	.	.	.	.	.	.	.	.	.	.	.	.	.
Nicoraepoa subenervis	.	.	.	.	.	.	.	.	.	.	.	.	.	.	.	.	.	.	.	.	.	.	.	.	.	.	.	.	.

Bog	146	147	148	149	150	151	152	153	154	155	156	157	158	159	160	161	162	163	164	165	166	167	168	169	170	171	172	173	174
Nitrophila australis	.	.	.	.	.	.	.	.	.	.	.	.	.	.	.	.	.	.	.	.	.	.	.	.	.	.	.	.	.
Nothofagus antarctica	.	.	.	.	.	.	.	.	.	.	.	.	.	.	.	.	.	.	.	.	.	.	.	.	.	.	1	1	1
Nototriche rugosa	.	.	.	.	.	.	.	.	.	.	.	.	.	.	.	.	.	.	.	.	.	.	.	.	.	.	.	.	.
Ochetophila nana	.	.	.	.	.	.	.	.	.	.	.	.	.	.	.	.	.	.	.	.	.	.	.	.	.	.	1	.	1
Olsynium junceum	.	.	.	.	.	.	.	.	.	.	.	.	.	.	.	.	.	.	.	.	.	.	.	.	.	.	.	.	.
Oreobolus obtusangulus	.	.	.	.	.	.	.	.	.	.	.	.	.	.	.	.	.	.	.	.	.	.	.	.	.	.	1	.	1
Oritrophium limnophilum	.	.	.	.	.	.	.	.	.	.	.	.	.	.	.	.	.	.	.	.	.	.	.	.	.	.	.	.	.
Osmorhiza glabrata	.	.	.	.	.	.	.	.	.	.	.	.	.	.	.	.	.	.	.	.	.	.	.	.	.	.	.	.	.
Ourisia alpina	.	.	.	.	.	.	.	.	.	.	.	.	.	.	.	.	.	.	.	.	.	.	.	.	.	.	1	1	1
Ourisia muscosa	.	.	.	.	.	.	.	.	.	.	.	.	.	.	.	.	.	.	.	.	.	.	.	.	.	.	.	.	.
Ourisia ruelloides	.	.	.	.	.	.	.	.	.	.	.	.	.	.	.	.	.	.	.	.	.	.	.	.	.	.	.	.	.
Oxychloe andina	.	.	.	.	.	.	1	1	1	1	1	1	.	.	.	.	.	1	1	.	.	.	1	1	1	.	.	.	.
Oxychloe bisexualis	.	.	.	.	.	.	.	.	.	.	.	.	.	.	.	.	.	.	.	.	.	.	.	.	.	.	.	.	.
Oxychloe castellanosii	.	.	.	.	.	.	.	.	.	.	.	.	.	.	.	1	1	.	.	.	.	.	.	.	.	.	.	.	.
Oxychloe haumaniana	.	.	.	.	.	.	.	.	.	.	.	.	.	.	.	.	.	.	.	.	.	.	.	.	.	.	.	.	.
Oxychloe mendocina	.	.	.	.	.	.	.	.	.	.	.	.	.	.	.	.	.	.	.	.	.	.	.	.	.	.	.	.	.
Patosia clandestina	.	.	.	.	.	.	.	.	.	.	.	.	.	.	.	.	.	.	.	.	.	.	.	.	.	.	.	.	1
Perezia capito	.	.	.	.	.	.	.	.	.	.	.	.	.	.	.	.	.	.	.	.	.	.	.	.	.	.	.	.	.
Perezia delicata	.	.	.	.	.	.	.	.	.	.	.	.	.	.	.	.	.	.	.	.	.	.	.	.	.	.	.	.	.
Perezia fonkii	.	.	.	.	.	.	.	.	.	.	.	.	.	.	.	.	.	.	.	.	.	.	.	.	.	.	.	.	1
Perezia pedicularidifolia	.	.	.	.	.	.	.	.	.	.	.	.	.	.	.	.	.	.	.	.	.	.	.	.	.	.	.	.	1
Perezia pinnatifida	.	.	.	.	.	.	.	.	.	.	.	.	.	.	.	.	.	.	.	.	.	.	.	.	.	.	.	.	.
Petroravenia friesii	.	.	.	.	.	.	.	.	.	.	.	.	.	.	.	.	.	.	.	.	.	.	.	.	.	.	.	.	.
Petroravenia werdermannii	.	.	.	.	.	.	.	.	.	.	.	.	.	.	.	.	.	.	.	.	.	.	.	.	.	.	.	.	.
Phleum alpinum	.	.	.	.	.	.	.	.	.	.	.	.	.	.	.	.	.	.	.	.	.	.	.	.	.	.	.	.	1
Phylloscirpus acaulis	.	.	1	1	1	.	1	1	1	.	.	.	.	1	.	.	.	.	.	1	1	1	1	1	1	1	1	1	1
Phylloscirpus boliviensis	.	.	.	.	.	.	.	.	.	.	.	.	.	.	.	.	.	.	.	.	.	.	.	.	.	.	.	.	.
Phylloscirpus deserticola	1	.	1	.	.	.	.	.	.	.	.	.	.	.	.	.	.	.	.	.	.	.	1	1	.	.	.	.	.
Pinguicula antarctica	.	.	.	.	.	.	.	.	.	.	.	.	.	.	.	.	.	.	.	.	.	.	.	.	.	.	.	.	.
Plantago barbata	.	.	1	1	.	.	1	1	1	1	.	.	.	.	.	.	.	.	.	1	1	1	.	.	.	.	.	.	1
Plantago rigida	.	.	.	.	.	.	.	.	.	.	.	.	.	.	.	.	.	.	.	.	.	.	.	.	.	.	.	.	.
Plantago tubulosa	1	1	.	.	.	.	.	.	.	.	.	.	.	1	.	.	.	.	.	1	1	1	1	1	.	.	.	.	.

Bog	146	147	148	149	150	151	152	153	154	155	156	157	158	159	160	161	162	163	164	165	166	167	168	169	170	171	172	173	174
Plantago uniglumis	.	.	.	.	.	.	.	.	.	.	.	.	.	.	.	.	.	.	.	.	.	.	.	.	.	.	.	.	.
Poa alopecurus	.	.	.	.	.	.	.	.	.	.	.	.	.	.	.	.	.	.	.	.	.	.	.	.	.	.	.	.	.
Poa hachadoensis	.	.	.	.	.	.	.	.	.	.	.	.	.	.	.	.	.	.	.	.	.	.	.	.	.	.	.	.	.
Poa perligulata	1	1	.	.	.	.	.	.	.	.	.	.	.	.	.	.	.	.	.	.	.	.	.	.	.	.	.	.	.
Polypogon interruptus	.	.	1	.	.	.	.	.	.	.	.	.	.	.	.	.	.	.	.	1	1	1	.	.	.	1	.	1	.
Primula magellanica	.	.	.	.	.	.	.	.	.	.	.	.	.	.	.	.	.	.	.	.	.	.	.	.	.	.	1	.	.
Puccinellia frigida	.	.	.	1	1	1	.	.	.	.	.	.	1	.	1	.	1	.	.	.	.	.	.	.	.	1	.	.	.
Quinchamalium chilense	.	.	.	.	.	.	.	.	.	.	.	.	.	.	.	.	.	.	.	.	.	.	.	.	.	.	.	.	.
Ranunculus breviscapus	.	.	.	.	.	.	.	.	.	.	.	.	.	.	.	.	.	.	.	.	.	.	.	.	.	.	.	.	.
Ranunculus fuegianus	.	.	.	.	.	.	.	.	.	.	.	.	.	.	.	.	.	.	.	.	.	.	.	.	.	.	.	.	.
Ranunculus mandonianus	.	.	.	.	.	.	.	.	.	.	.	.	.	.	.	.	.	.	.	.	.	.	.	.	.	.	.	.	.
Ranunculus peduncularis	.	.	.	.	.	.	.	.	.	.	.	.	.	.	.	.	.	.	.	.	.	.	.	.	.	.	1	.	1
Ranunculus trichophyllus	.	.	.	.	.	.	.	.	.	.	.	.	.	.	.	.	.	.	.	.	.	.	.	.	.	.	.	.	.
Halerpestes uniflora	.	.	.	1	1	.	1	1	.	.	.	.	.	.	.	.	.	1	.	.	.	.	.	.	.	1	.	.	.
Rubus geoides	.	.	.	.	.	.	.	.	.	.	.	.	.	.	.	.	.	.	.	.	.	.	.	.	.	.	.	.	.
Rumex magellanicus	.	.	.	.	.	.	.	.	.	.	.	.	.	.	.	.	.	.	.	.	.	.	.	.	.	.	.	.	.
Rytidosperma lechleri	.	.	.	.	.	.	.	.	.	.	.	.	.	.	.	.	.	.	.	.	.	.	.	.	.	.	.	.	.
Sarcocornia pulvinata	.	.	.	.	.	.	.	.	.	.	.	.	.	.	.	.	.	.	.	.	.	.	.	.	.	.	.	.	.
Schoenoplectus pungens	.	.	.	.	.	.	.	.	.	.	.	.	.	.	.	.	.	.	.	.	.	.	.	.	.	.	.	.	.
Schoenus andinus	.	.	.	.	.	.	.	.	.	.	.	.	.	.	.	.	.	.	.	.	.	.	.	.	.	.	1	1	1
Senecio breviscapus	.	.	.	.	.	.	.	.	.	.	.	.	.	.	.	.	.	.	.	.	.	.	.	.	.	.	.	.	.
Senecio diemii	.	.	.	.	.	.	.	.	.	.	.	.	.	.	.	.	.	.	.	.	.	.	.	.	.	.	.	.	1
Senecio fistulosus	.	.	.	.	.	.	.	.	.	.	.	.	.	.	.	.	.	.	.	.	.	.	.	.	.	.	.	1	1
Senecio parodii	.	.	.	.	.	.	.	.	.	.	.	.	.	.	.	.	.	.	.	.	.	.	.	.	.	.	.	.	1
Senecio peteroanus	.	.	.	.	.	.	.	.	.	.	.	.	.	.	.	.	.	.	.	.	.	.	.	.	.	.	.	.	.
Senecio serratifolius	.	.	.	.	.	.	.	.	.	.	.	.	.	.	.	.	.	.	.	.	.	.	.	.	.	.	.	.	.
Senecio trifurcatus	.	.	.	.	.	.	.	.	.	.	.	.	.	.	.	.	.	.	.	.	.	.	.	.	.	.	1	1	1
Sisyrinchium chilense	.	.	1	.	.	.	.	.	.	.	.	.	.	1	.	.	.	.	.	1	1	1	1	1	1	.	.	.	.
Sisyrinchium patagonicum	.	.	.	.	.	.	.	.	.	.	.	.	.	.	.	.	.	.	.	.	.	.	.	.	.	.	1	.	1
Sisyrinchium pearcei	.	.	.	.	.	.	.	.	.	.	.	.	.	.	.	.	.	.	.	.	.	.	.	.	.	.	.	.	.
Stellaria debilis	.	.	.	.	.	.	.	.	.	.	.	.	.	.	.	.	.	.	.	.	.	.	.	.	.	.	.	.	.
Stuckenia filiformis	.	.	.	.	.	.	.	.	.	.	.	.	.	.	.	.	.	.	.	.	.	.	.	.	.	.	.	.	.

**Bog**	**146**	**147**	**148**	**149**	**150**	**151**	**152**	**153**	**154**	**155**	**156**	**157**	**158**	**159**	**160**	**161**	**162**	**163**	**164**	**165**	**166**	**167**	**168**	**169**	**170**	**171**	**172**	**173**	**174**
Stuckenia striata	.	.	.	.	.	.	.	.	.	.	.	.	.	.	.	.	.	.	.	.	.	.	.	.	.	.	.	.	.
Symphyotrichum peteroanum	.	.	.	.	.	.	.	.	.	.	.	.	.	.	.	.	.	.	.	.	.	.	.	.	.	.	.	.	.
Symphyotrichum vahlii	.	.	.	.	.	.	.	.	.	.	.	.	.	.	.	.	.	.	.	.	.	.	.	.	.	.	1	1	1
Tetroncium magellanicum	.	.	.	.	.	.	.	.	.	.	.	.	.	.	.	.	.	.	.	.	.	.	.	.	.	.	.	.	.
Tribeles australis	.	.	.	.	.	.	.	.	.	.	.	.	.	.	.	.	.	.	.	.	.	.	.	.	.	.	.	.	.
Trifolium amabile	.	.	1	.	.	.	.	.	.	.	.	.	.	.	.	.	.	.	.	.	1	.	.	1	.	.	.	.	.
Trifolium polymorphum	.	.	.	.	.	.	.	.	.	.	.	.	.	.	.	.	.	.	.	.	.	.	.	.	.	.	.	.	.
Triglochin concinna	.	.	.	1	1	1	.	.	1	.	.	.	.	.	.	.	.	.	1	.	.	.	.	.	.	1	.	.	.
Triglochin palustris	.	.	1	1	1	1	.	.	.	.	.	.	.	.	.	.	.	1	1	1	1	.	.	.	1	1	.	.	.
Triglochin striata	.	.	.	.	.	.	.	.	.	.	.	.	.	.	.	.	.	.	.	.	.	.	.	.	.	.	.	.	.
Trisetum caudulatum	.	.	.	.	.	.	.	.	.	.	.	.	.	.	.	.	.	.	.	.	.	.	.	.	.	.	.	.	.
Trisetum preslei	.	.	.	.	.	.	.	.	.	.	.	.	.	.	.	.	.	.	.	.	.	.	.	.	.	.	.	.	.
Koeleria spicata	.	.	.	.	.	.	.	.	.	.	.	.	.	.	.	.	.	.	.	.	.	.	.	.	.	.	.	.	.
Utricularia gibba	.	.	.	.	.	.	.	.	.	.	.	.	.	.	.	.	.	.	.	.	.	.	.	.	.	.	.	.	.
Vahlodea atropurpurea	.	.	.	.	.	.	.	.	.	.	.	.	.	.	.	.	.	.	.	.	.	.	.	.	.	.	.	.	.
Valeriana fonckii	.	.	.	.	.	.	.	.	.	.	.	.	.	.	.	.	.	.	.	.	.	.	.	.	.	.	.	.	.
Valeriana macrorhiza	.	.	.	.	.	.	.	.	.	.	.	.	.	.	.	.	.	.	.	.	.	.	.	.	.	.	1	.	1
Viola pygmaea	.	.	.	.	.	.	.	.	.	.	.	.	.	.	.	.	.	.	.	.	.	.	.	.	.	.	.	.	.
Werneria apiculata	.	.	.	.	.	.	.	.	.	.	.	.	.	.	.	.	.	.	.	.	.	.	.	.	.	.	.	.	.
Werneria pinnatifida	1	1	.	.	.	.	1	.	.	.	.	.	.	.	.	.	.	.	.	.	.	.	.	.	.	.	.	.	.
Werneria pygmaea	1	.	1	.	.	.	1	1	1	1	.	.	.	1	.	.	.	.	.	1	1	1	1	1	1	.	.	.	.
Werneria solivifolia	.	1	.	.	.	.	.	.	.	.	.	.	.	.	.	.	.	.	.	.	.	.	.	.	.	.	.	.	.
Werneria spathulata	.	.	1	.	.	.	.	.	.	.	.	.	.	.	.	.	.	.	.	1	.	.	1	.	.	.	.	.	.
Xenophyllum incisum	.	.	.	.	.	.	.	.	.	.	.	.	.	.	.	.	.	.	.	.	.	.	.	.	.	.	.	.	.
Zameioscirpus atacamensis	.	.	.	1	1	1	.	.	.	.	.	.	.	.	1	.	.	1	1	.	.	.	.	.	.	1	.	.	.
Zameioscirpus gaimardiodes	.	.	.	.	.	.	.	.	.	.	.	.	.	.	.	.	.	.	.	.	.	.	.	.	.	.	.	.	.
Zameioscirpus muticus	1	.	1	.	.	.	1	1	1	1	1	1	.	1	.	1	1	.	.	1	1	1	1	1	.	.	.	.	.

Bog	175	176	177	178	179	180	181	182	183	184	185	186	187	188	189	190	191	192	193	194	195	196	197	198	199	200	201	202	203
**Operational zone**	**S**	**S**	**S**	**S**	**S**	**S**	**S**	**S**	**S**	**S**	**S**	**S**	**S**	**T**	**T**	**T**	**T**	**T**	**T**	**T**	**T**	**T**	**S**	**T**	**T**	**T**	**T**	**T**	**T**
**Cluster**	**7**	**7**	**7**	**7**	**2**	**2**	**2**	**2**	**2**	**2**	**2**	**7**	**2**	**2**	**2**	**2**	**2**	**2**	**2**	**2**	**2**	**2**	**7**	**2**	**2**	**2**	**2**	**6**	**2**
**Bioregion**	**S**	**S**	**S**	**S**	**S**	**S**	**S**	**S**	**S**	**S**	**S**	**S**	**S**	**S**	**S**	**S**	**S**	**S**	**S**	**S**	**S**	**S**	**S**	**S**	**S**	**S**	**S**	**S**	**S**
**Longitude**	**-70.39**	**-70.52**	**-70.52**	**-70.52**	**-70.19**	**-70.14**	**-70.13**	**-70.13**	**-70.14**	**-70.21**	**-70.14**	**-70.4**	**-70.1**	**-70.1**	**-70.1**	**-70.05**	**-69.66**	**-69.66**	**-69.65**	**-69.64**	**-69.65**	**-69.54**	**-70.39**	**-69.68**	**-69.67**	**-69.41**	**-69.62**	**-69.55**	**-69.98**
**Latitude**	**-35.98**	**-35.2**	**-35.2**	**-35.2**	**-35.13**	**-35.11**	**-35.09**	**-35.09**	**-35.1**	**-35.1**	**-35.12**	**-35.98**	**-35.11**	**-34.69**	**-34.76**	**-34.77**	**-34.74**	**-34.74**	**-34.73**	**-34.72**	**-34.73**	**-34.18**	**-35.99**	**-34.18**	**-34.16**	**-34.23**	**-33.62**	**-33.61**	**-32.76**
Acaena antarctica	.	.	.	.	.	.	.	.	.	.	.	.	.	.	.	.	.	.	.	.	.	.	.	.	.	.	.	.	.
Acaena macrocephala	.	.	.	.	.	.	.	.	.	.	.	.	.	.	.	.	.	.	.	.	.	.	.	.	.	.	.	.	.
Acaena magellanica	.	.	.	.	.	.	1	1	.	.	.	1	.	1	1	1	.	.	.	.	.	.	1	.	.	.	.	.	1
Acaena ovalifolia	.	.	.	.	.	.	.	.	.	.	.	.	.	.	.	.	.	.	.	.	.	.	.	.	.	.	.	.	.
Acaena pinnatifida	.	.	.	.	.	.	.	.	.	.	.	.	.	.	.	.	.	.	.	.	.	.	.	.	.	.	.	.	.
Adesmia retusa	.	.	.	.	.	.	.	.	.	.	.	.	.	.	.	.	.	.	.	.	.	.	.	.	.	.	.	.	.
Agrostis breviculmis	.	.	.	.	.	.	.	.	.	.	.	.	.	.	.	.	.	.	.	.	.	.	.	.	.	.	.	.	.
Agrostis imberbis	.	.	.	.	.	.	.	.	.	.	.	.	.	.	.	.	.	.	.	.	.	.	.	.	.	.	.	.	.
Agrostis meyenii	.	.	.	.	.	.	.	.	.	.	.	.	.	.	.	.	.	.	.	.	.	.	.	.	.	.	.	.	.
Agrostis perennans	.	.	.	.	.	.	.	.	.	.	.	.	.	.	.	.	.	.	.	.	.	.	.	.	.	.	.	.	.
Alchemilla pinnata	.	.	.	.	.	.	.	.	.	.	.	.	.	.	.	.	.	.	.	.	.	.	.	.	.	.	.	.	.
Alopecurus magellanicus	.	.	.	.	.	1	1	1	1	.	.	.	.	.	.	.	.	.	.	.	.	.	1	.	.	.	.	.	.
Amphiscirpus nevadensis	.	.	.	.	.	.	.	.	.	.	.	.	.	.	.	.	.	.	.	.	.	.	.	.	.	.	.	.	.
Anagallis alternifolia	.	1	1	1	.	.	1	1	.	.	.	1	1	.	.	.	.	.	.	.	.	.	1	.	.	.	.	.	.
Antennaria chilensis	.	.	.	.	.	.	.	.	.	.	.	.	.	.	.	.	.	.	.	.	.	.	.	.	.	.	.	.	.
Anthoxanthum redolens	.	.	.	.	.	.	.	.	.	.	.	.	.	.	.	.	.	.	.	.	.	.	.	.	.	.	.	.	.
Apium panul	.	.	.	.	.	.	.	.	.	.	.	.	.	.	.	.	.	.	.	.	.	.	.	.	.	.	.	.	.
Arenaria rivularis	.	.	.	.	.	.	.	.	.	.	.	.	.	.	.	.	.	.	.	.	.	.	.	.	.	.	.	.	.
Arenaria serpens	.	.	.	.	.	.	.	.	.	.	.	.	.	.	.	.	.	.	.	.	.	.	.	.	.	.	.	.	.
Arjona pusilla	.	.	.	.	.	.	.	.	.	.	.	.	.	.	.	.	.	.	.	.	.	.	.	.	.	.	.	.	.
Astragalus bustillosii	.	.	.	.	.	.	.	.	.	.	.	.	.	.	.	.	.	.	.	.	.	.	.	.	.	.	.	.	.
Astragalus micranthellus	.	.	.	.	.	.	.	.	.	.	.	.	.	.	.	.	.	.	.	.	.	.	.	.	.	.	.	.	.
Azolla filiculoides	.	.	.	.	.	.	.	.	.	.	.	.	.	.	.	.	.	.	.	.	.	.	.	.	.	.	.	.	.
Azorella boelckei	.	.	.	.	.	.	.	.	.	.	.	.	.	.	.	.	.	.	.	.	.	.	.	.	.	.	.	.	.

Bog	175	176	177	178	179	180	181	182	183	184	185	186	187	188	189	190	191	192	193	194	195	196	197	198	199	200	201	202	203
Azorella burkartii	.	.	.	.	.	.	.	.	.	.	.	.	.	.	.	.	.	.	.	.	.	.	.	.	.	.	.	.	.
Azorella cryptantha	.	.	.	.	.	.	.	.	.	.	.	.	.	.	.	.	.	.	.	.	.	.	.	.	.	.	.	.	.
Azorella lycopodioides	.	.	.	.	.	.	.	.	.	.	.	.	.	.	.	.	.	.	.	.	.	.	.	.	.	.	.	.	.
Azorella trifoliolata	.	.	.	.	.	.	.	.	.	.	.	.	.	.	.	.	.	.	.	.	.	.	.	.	.	.	.	.	.
Baccharis acaulis	.	.	.	.	.	.	.	.	.	.	.	.	.	.	.	.	.	.	.	.	.	.	.	.	.	.	.	.	.
Baccharis caespitosa	.	.	.	.	.	.	.	.	.	.	.	.	.	.	.	.	.	.	.	.	.	.	.	.	.	.	.	.	.
Baccharis magellanica	.	.	.	.	.	.	.	.	.	.	.	.	.	.	.	.	.	.	.	.	.	.	.	.	.	.	.	.	.
Belloa chilensis	.	.	.	.	.	.	.	.	.	.	.	.	.	.	.	.	.	.	.	.	.	.	.	.	.	.	.	.	.
Bromus catharticus	.	.	.	.	.	.	.	.	.	.	.	.	.	.	.	.	.	.	.	.	.	.	.	.	.	.	.	.	.
Calandrinia acaulis	.	.	.	.	.	.	.	.	.	.	.	.	.	.	.	.	.	.	.	.	.	.	.	.	.	.	.	.	.
Calandrinia compacta	.	.	.	.	.	.	.	.	.	.	.	.	.	.	.	.	.	.	.	.	.	.	.	.	.	.	.	.	.
Calceolaria biflora	.	.	.	.	.	.	.	.	.	.	.	.	.	.	.	.	.	.	.	.	.	.	.	.	.	.	.	.	.
Calceolaria cana	.	.	.	.	.	.	.	.	.	.	.	.	.	.	.	.	.	.	.	.	.	.	.	.	.	.	.	.	.
Calceolaria corymbosa	.	.	.	.	.	.	.	.	.	.	.	.	.	.	.	.	.	.	.	.	.	.	.	.	.	.	.	.	.
Calceolaria filicaulis	.	.	.	.	.	.	.	.	.	.	.	.	.	.	.	.	.	.	.	.	.	.	.	.	.	.	.	.	.
Callitriche lechleri	.	.	.	.	.	.	.	.	.	.	.	.	.	.	.	.	.	.	.	.	.	.	.	.	.	.	.	.	.
Caltha appendiculata	.	.	.	.	.	.	.	.	.	.	.	.	.	.	.	.	.	.	.	.	.	.	.	.	.	.	.	.	.
Caltha sagittata	1	1	1	1	.	.	1	1	.	1	.	1	.	.	.	.	.	.	.	.	.	.	1	.	.	.	.	.	.
Cardamine cordata	.	.	.	1	1	1	.	.	.	.	.	1	.	.	.	.	.	.	.	.	.	.	1	.	.	.	.	.	.
Cardamine glacialis	.	.	.	.	.	.	.	.	.	.	.	.	.	.	.	.	.	.	.	.	.	.	.	.	.	.	.	.	.
Cardamine tenuirostris	.	.	.	.	.	.	.	.	.	.	.	.	.	.	.	.	.	.	.	.	.	.	.	.	.	.	.	.	.
Cardamine volckmannii	.	.	.	.	.	.	.	.	.	.	.	.	.	.	.	.	.	.	.	.	.	.	.	.	.	.	.	.	.
Carex acaulis	.	.	.	.	.	.	.	.	.	.	.	.	.	.	.	.	.	.	.	.	.	.	.	.	.	.	.	.	.
Carex atropicta	1	1	.	1	.	.	.	.	.	.	.	1	.	.	.	.	.	.	.	.	.	.	.	.	.	.	.	.	.
Carex banksii	.	1	1	.	.	.	.	.	.	.	.	1	.	.	.	.	.	.	.	.	.	.	1	.	.	.	.	.	.
Carex caduca	.	.	.	.	.	.	.	.	.	.	.	.	.	.	.	.	.	.	.	.	.	.	.	.	.	.	.	.	.
Carex decidua	.	.	.	.	.	.	.	.	.	.	.	.	.	.	.	.	.	.	.	.	.	.	.	.	.	.	.	.	.
Carex fuscula	.	.	.	.	.	.	.	.	.	.	.	.	.	.	.	.	.	.	.	.	.	.	.	.	.	.	.	.	.
Carex gayana	.	1	1	1	1	1	1	1	1	1	1	.	1	1	1	1	1	1	1	1	1	.	1	.	.	1	.	1	1
Carex hypoleucos	.	.	.	.	.	.	.	.	.	.	.	.	.	.	.	.	.	.	.	.	.	.	.	.	.	.	.	.	.
Carex macloviana	1	1	.	1	.	.	.	.	.	1	.	1	.	.	.	.	.	.	.	.	.	.	1	1	.	.	1	.	.
Carex magellanica	.	.	.	.	.	.	.	.	.	.	.	.	.	.	.	.	.	.	.	.	.	.	.	.	.	.	.	.	.
Carex malmei	.	.	.	.	.	.	.	.	.	.	.	.	.	.	.	.	.	.	.	.	.	.	.	.	.	.	.	.	.
Carex maritima	.	.	.	.	.	.	.	.	.	.	.	.	.	.	.	.	.	.	.	.	.	1	.	1	1	.	1	1	.
Carex microglochin	.	.	.	.	.	.	.	.	.	.	.	.	.	.	.	.	.	.	.	.	.	.	.	.	.	.	.	.	.
Carex pleioneura	.	.	.	.	.	.	.	.	.	.	.	.	.	.	.	.	.	.	.	.	.	.	.	.	.	.	.	.	.
Carex ruthsatzae	.	.	.	.	.	.	.	.	.	.	.	.	.	.	.	.	.	.	.	.	.	.	.	.	.	.	.	.	.
Carex vallis-pulchrae	.	.	.	.	.	1	1	1	1	1	1	.	.	1	.	.	.	.	1	1	.	.	1	.	.	.	.	.	.

Bog	175	176	177	178	179	180	181	182	183	184	185	186	187	188	189	190	191	192	193	194	195	196	197	198	199	200	201	202	203
Carpha schoenoides	.	.	.	.	.	.	.	.	.	.	.	.	.	.	.	.	.	.	.	.	.	.	.	.	.	.	.	.	.
Castilleja pumila	.	.	.	.	.	.	.	.	.	.	.	.	.	.	.	.	.	.	.	.	.	.	.	.	.	.	.	.	.
Catabrosa werdermannii	.	.	.	.	.	.	.	.	.	.	.	.	.	.	.	.	.	.	.	.	.	.	.	.	.	.	.	.	.
Cerastium humifusum	.	.	.	.	.	.	.	.	.	.	.	.	.	.	.	.	.	.	.	.	.	.	.	.	.	.	.	.	.
Cerastium montioides	.	.	.	.	.	.	.	.	.	.	.	.	.	.	.	.	.	.	.	.	.	.	.	.	.	.	.	.	.
Chiliotrichum diffusum	.	1	.	.	.	.	.	.	.	.	.	1	.	.	.	.	.	.	.	.	.	.	1	.	.	.	.	.	.
Chusquea culeou	.	.	.	.	.	.	.	.	.	.	.	.	.	.	.	.	.	.	.	.	.	.	.	.	.	.	.	.	.
Colobanthus quitensis	1	.	.	.	.	.	1	.	.	.	.	.	1	1	.	1	1	1	1	1	1	1	.	1	1	1	1	.	1
Cortaderia egmontiana	.	.	.	.	.	.	.	.	.	.	.	.	.	.	.	.	.	.	.	.	.	.	.	.	.	.	.	.	.
Cotula mexicana	.	.	.	.	.	.	.	.	.	.	.	.	.	.	.	.	.	.	.	.	.	.	.	.	.	.	.	.	.
Crassula peduncularis	.	.	.	.	.	.	.	.	.	.	.	.	.	.	.	.	.	.	.	.	.	.	.	.	.	.	.	.	.
Cuatrecasasiella argentina	.	.	.	.	.	.	.	.	.	.	.	.	.	.	.	.	.	.	.	.	.	.	.	.	.	.	.	.	.
Deschampsia antarctica	.	.	.	.	.	.	.	.	.	.	.	.	.	.	.	.	.	.	.	.	.	.	.	.	.	.	.	.	.
Deschampsia caespitosa	.	1	1	1	.	1	1	1	1	1	1	1	1	.	.	.	.	.	.	.	.	.	1	.	.	.	.	.	.
Deschampsia patula	1	.	.	.	.	.	.	.	.	.	.	.	.	.	.	.	.	.	.	.	.	.	1	1	.	.	.	.	.
Cinnagrostis brevifolia	.	.	.	.	.	.	.	.	.	.	.	.	.	.	.	.	.	.	.	.	.	.	.	.	.	.	.	.	.
Deschampsia chrysantha	.	.	.	.	.	.	.	.	.	.	.	.	.	.	.	.	.	.	.	.	.	.	.	.	.	.	.	.	.
Cinnagrostis chrysophylla	.	.	.	.	.	.	.	.	.	.	.	.	.	.	.	.	.	.	.	.	.	.	.	.	.	.	.	.	.
Deschampsia chrysostachya	.	.	.	.	.	.	.	.	.	.	.	.	.	.	.	.	.	.	.	.	.	.	.	.	.	.	.	.	.
Deschampsia eminens	.	.	.	.	.	.	.	.	.	.	.	.	.	.	.	.	.	.	.	.	.	.	.	.	.	.	.	.	.
Deschampsia hackelii	.	.	.	.	.	.	.	.	.	.	.	.	.	.	.	.	.	.	.	.	.	.	.	.	.	.	.	.	.
Cinnagrostis minima	.	.	.	.	.	.	.	.	.	.	.	.	.	.	.	.	.	.	.	.	.	.	.	.	.	.	.	.	.
Deschampsia ovata	.	.	.	.	.	.	.	.	.	.	.	.	.	.	.	.	.	.	.	.	.	.	.	.	.	.	.	.	.
Cinnagrostis rigescens	.	.	.	.	.	.	.	.	.	.	.	.	.	.	.	.	.	.	.	.	.	.	.	.	.	.	.	.	.
Cinnagrostis spicigera	.	.	.	.	.	.	.	.	.	.	.	.	.	.	.	.	.	.	.	.	.	.	.	.	.	.	.	.	.
Cinnagrostis velutina	.	.	.	.	.	.	.	.	.	.	.	.	.	.	.	.	.	.	.	.	.	.	.	.	.	.	.	.	.
Cinnagrostis vicunarum	.	.	.	.	.	.	.	.	.	.	.	.	.	.	.	.	.	.	.	.	.	.	.	.	.	.	.	.	.
Distichia filamentosa	.	.	.	.	.	.	.	.	.	.	.	.	.	.	.	.	.	.	.	.	.	.	.	.	.	.	.	.	.
Distichia muscoides	.	.	.	.	.	.	.	.	.	.	.	.	.	.	.	.	.	.	.	.	.	.	.	.	.	.	.	.	.
Distichlis humilis	.	.	.	.	.	.	.	.	.	.	.	.	.	.	.	.	.	.	.	.	.	.	.	.	.	.	.	.	.
Distichlis scoparia	.	.	.	.	.	.	.	.	.	.	.	.	.	.	.	.	.	.	.	.	.	.	.	.	.	.	.	.	.
Distichlis spicata	.	.	.	.	.	.	.	.	.	.	.	.	.	.	.	.	.	.	.	.	.	.	.	.	.	.	.	.	.

Bog	175	176	177	178	179	180	181	182	183	184	185	186	187	188	189	190	191	192	193	194	195	196	197	198	199	200	201	202	203
Draba pusilla	.	.	.	.	.	.	.	.	.	.	.	.	.	.	.	.	.	.	.	.	.	.	.	.	.	.	.	.	.
Eleocharis melanomphala	.	.	.	.	.	.	.	.	.	.	.	.	.	.	.	.	.	.	.	.	.	.	.	.	.	.	.	.	.
Eleocharis pseudoalbibracteata	.	.	.	.	.	.	.	.	.	.	.	.	.	.	.	.	.	.	.	.	.	.	.	.	.	.	.	.	.
Elodea potamogeton	.	.	.	.	.	.	.	.	.	.	.	.	.	.	.	.	.	.	.	.	.	.	.	.	.	.	.	.	.
Empetrum rubrum	1	.	.	.	.	.	.	.	.	.	.	1	.	.	.	.	.	.	.	.	.	.	1	.	.	.	.	.	.
Epilobium australe	.	.	.	.	.	.	.	.	.	.	.	.	.	.	.	.	.	.	.	.	.	.	.	.	.	.	.	.	.
Epilobium barbeyanum	.	1	1	1	.	.	.	.	1	1	.	.	.	.	.	.	.	.	.	.	.	.	1	.	.	.	.	.	.
Epilobium ciliatum	.	.	.	.	.	.	.	.	.	.	.	.	.	.	.	.	.	.	.	.	.	.	.	.	.	.	.	.	.
Epilobium denticulatum	.	.	.	.	.	.	.	.	.	.	.	.	.	.	.	.	.	.	.	.	.	.	.	.	.	.	.	.	.
Epilobium fragile	.	.	.	.	.	.	.	.	.	.	.	.	.	.	.	.	.	.	.	.	.	.	.	.	.	.	.	.	.
Epilobium glaucum	.	.	.	.	.	.	.	.	.	.	.	.	.	.	.	.	.	.	.	.	.	.	.	.	.	.	.	.	.
Epilobium nivale	.	.	.	.	.	.	.	.	.	.	.	.	.	.	.	.	.	.	.	.	.	.	.	.	.	.	.	.	.
Erigeron andicola	.	.	.	.	.	.	.	.	.	.	.	.	.	.	.	.	.	.	.	.	.	.	.	.	.	.	.	.	.
Erigeron leptopetalus	.	.	.	.	.	.	.	.	.	.	.	.	.	.	.	.	.	.	.	.	.	.	.	.	.	.	.	.	.
Erigeron myosotis	.	.	.	.	.	.	.	.	.	.	.	.	.	.	.	.	.	.	.	.	.	.	.	.	.	.	.	.	.
Erigeron patagonicus	.	.	.	.	.	.	.	.	.	.	.	.	.	.	.	.	.	.	.	.	.	.	.	.	.	.	.	.	.
Erythranthe cuprea	.	.	.	.	.	.	.	.	.	.	.	.	.	.	.	.	.	.	.	.	.	.	.	.	.	.	.	.	.
Erythranthe depressa	.	.	.	.	.	.	.	.	.	.	.	.	.	.	.	.	.	.	.	.	.	.	.	.	.	.	.	.	.
Erythranthe glabrata	.	.	.	.	.	.	.	.	.	.	.	.	.	.	.	.	.	.	.	.	.	.	.	.	.	.	.	.	.
Erythranthe lutea	.	1	1	1	.	.	.	.	1	.	1	.	1	.	.	1	.	.	.	.	.	.	1	.	.	.	.	.	.
Escallonia virgata	.	.	.	.	.	.	.	.	.	.	.	.	.	.	.	.	.	.	.	.	.	.	.	.	.	.	.	.	.
Euphrasia antarctica	.	.	.	.	.	.	.	.	.	.	.	.	.	.	.	.	.	.	.	.	.	.	.	.	.	.	.	.	.
Euphrasia chrysantha	.	.	.	.	.	.	.	.	.	.	.	.	.	.	.	.	.	.	.	.	.	.	.	.	.	.	.	.	.
Euphrasia subexserta	.	1	1	1	.	.	.	.	.	.	.	1	.	.	.	.	.	.	.	.	.	.	1	.	.	.	.	.	.
Festuca hypsophila	.	.	.	.	.	.	.	.	.	.	.	.	.	.	.	.	.	.	.	.	.	.	.	.	.	.	.	.	.
Festuca kurtziana	.	.	.	.	.	.	.	.	.	.	.	.	.	.	.	.	.	.	.	.	.	.	.	.	.	.	.	.	.
Festuca lilloi	.	.	.	.	.	.	.	.	.	.	.	.	.	.	.	.	.	.	.	.	.	.	.	.	.	.	.	.	.
Festuca magellanica	.	.	.	.	.	.	.	.	.	.	.	.	.	.	.	.	.	.	.	.	.	.	.	.	.	.	.	.	.
Festuca nardifolia	.	.	.	.	.	.	.	.	.	.	.	.	.	.	.	.	.	.	.	.	.	.	.	.	.	.	.	.	.
Festuca rigescens	.	.	.	.	.	.	.	.	.	.	.	.	.	.	.	.	.	.	.	.	.	.	.	.	.	.	.	.	.
Festuca werdermannii	.	.	.	.	.	.	.	.	.	.	.	.	.	.	.	.	.	.	.	.	.	.	.	.	.	.	.	.	.
Frankenia triandra	.	.	.	.	.	.	.	.	.	.	.	.	.	.	.	.	.	.	.	.	.	.	.	.	.	.	.	.	.
Gamocarpha graminea	.	.	.	.	.	.	.	.	.	.	.	.	.	.	.	.	.	.	.	.	.	.	.	.	.	.	.	.	.
Gamocarpha ventosa	.	.	.	.	.	.	.	.	.	.	.	.	.	.	.	.	.	.	.	.	.	.	.	.	.	.	.	.	.
Gamochaeta chamissonis	.	.	.	.	.	.	.	.	.	.	.	.	.	.	.	.	.	.	.	.	.	.	.	.	.	.	.	.	.

Bog	175	176	177	178	179	180	181	182	183	184	185	186	187	188	189	190	191	192	193	194	195	196	197	198	199	200	201	202	203
Gamochaeta longipedicellata	.	.	.	.	.	.	.	.	.	.	.	.	.	.	.	.	.	.	.	.	.	.	.	.	.	.	.	.	.
Gamochaeta neuquensis	.	.	.	.	.	.	.	.	.	.	.	.	.	.	.	.	.	.	.	.	.	.	.	.	.	.	.	.	.
Gaultheria antarctica	.	.	.	.	.	.	.	.	.	.	.	.	.	.	.	.	.	.	.	.	.	.	.	.	.	.	.	.	.
Gaultheria caespitosa	.	.	.	.	.	.	.	.	.	.	.	.	.	.	.	.	.	.	.	.	.	.	.	.	.	.	.	.	.
Gaultheria pumila	.	.	.	1	.	.	.	.	.	.	.	1	.	.	.	.	.	.	.	.	.	.	1	.	.	.	.	.	.
Gavilea chica	.	.	.	.	.	.	.	.	.	.	.	.	.	.	.	.	.	.	.	.	.	.	.	.	.	.	.	.	.
Gentiana prostrata	1	1	.	1	.	.	.	.	1	1	.	1	.	.	.	.	1	1	.	.	1	1	1	.	.	.	1	.	.
Gentianella fiebrigii	.	.	.	.	.	.	.	.	.	.	.	.	.	.	.	.	.	.	.	.	.	.	.	.	.	.	.	.	.
Gentianella magellanica	.	.	.	.	.	.	1	.	.	.	.	1	.	1	.	1	1	.	.	.	.	.	1	.	.	.	.	.	1
Gentianella multicaulis	.	.	.	.	.	.	.	.	.	.	.	.	.	.	.	.	.	.	.	.	.	.	.	.	.	.	.	.	.
Gentianella ottonis	.	.	.	.	.	.	.	.	.	.	.	.	.	.	.	.	.	.	.	.	.	.	.	.	.	.	.	.	.
Gentianella primuloides	.	.	.	.	.	.	.	.	.	.	.	.	.	.	.	.	.	.	.	.	.	.	.	.	.	.	.	.	.
Gentianella pseudocrassula	.	.	.	.	.	.	.	.	.	.	.	.	.	.	.	.	.	.	.	.	.	.	.	.	.	.	.	.	.
Geranium sessiliflorum	.	.	.	.	.	.	.	.	.	.	.	.	.	.	.	.	.	.	.	.	.	.	.	.	.	.	.	.	.
Gunnera magellanica	.	1	1	1	.	.	.	.	.	.	.	.	.	.	.	.	.	.	.	.	.	.	1	.	.	.	.	.	.
Halenia caespitosa	.	.	.	.	.	.	.	.	.	.	.	.	.	.	.	.	.	.	.	.	.	.	.	.	.	.	.	.	.
Halerpestes cymbalaria	.	.	.	.	.	.	.	.	.	.	.	.	.	.	.	.	.	.	.	.	.	.	.	.	.	.	.	.	.
Halerpestes exilis	.	.	.	.	.	.	.	.	.	.	.	.	.	.	.	.	.	.	.	.	.	.	.	.	.	.	.	.	.
Hieracium antarcticum	.	.	.	.	.	.	.	.	.	.	.	.	.	.	.	.	.	.	.	.	.	.	.	.	.	.	.	.	.
Hordeum comosum	1	1	.	1	.	1	1	1	1	1	.	1	1	1	.	1	1	1	.	1	1	1	1	1	1	.	.	.	1
Hordeum muticum	.	.	.	.	.	.	.	.	.	.	.	.	.	.	.	.	.	.	.	.	.	.	.	.	.	.	.	.	.
Hypochaeris acaulis	.	.	.	.	.	.	.	.	.	.	.	.	.	.	.	.	.	.	.	.	.	.	.	.	.	.	.	.	.
Hypochaeris chondrilloides	.	.	.	.	.	.	.	.	.	.	.	.	.	.	.	.	.	.	.	.	.	.	.	.	.	.	.	.	.
Hypochaeris meyeniana	.	.	.	.	.	.	.	.	.	.	.	.	.	.	.	.	.	.	.	.	.	.	.	.	.	.	.	.	.
Hypochaeris palustris	.	.	.	.	.	.	.	.	.	.	.	.	.	.	.	.	.	.	.	.	.	.	.	.	.	.	.	.	.
Hypochaeris taraxacoides	.	.	.	.	.	.	.	.	.	.	.	.	.	.	.	.	.	.	.	.	.	.	.	.	.	.	.	.	.
Hypochaeris tenerifolia	.	.	.	.	.	.	.	.	.	.	.	.	.	.	.	.	.	.	.	.	.	.	.	.	.	.	.	.	.
Isolepis nigricans	.	.	.	.	.	.	.	.	.	.	.	.	.	.	.	.	.	.	.	.	.	.	.	.	.	.	.	.	.
Isolepsis inundata	.	.	.	.	.	.	.	.	.	.	.	.	.	.	.	.	.	.	.	.	.	.	.	.	.	.	.	.	.
Juncus balticus	.	.	.	1	1	.	.	.	1	1	.	.	1	.	1	1	1	1	1	1	1	.	1	1	1	1	.	.	1
Juncus stipulatus	.	.	.	.	.	.	.	.	.	.	.	.	.	.	.	.	.	.	.	.	.	.	.	.	.	.	.	.	.
Koeleria kurtzii	.	.	.	.	.	.	.	.	.	.	.	.	.	.	.	.	.	.	.	.	.	.	.	.	.	.	.	.	.

Bog	175	176	177	178	179	180	181	182	183	184	185	186	187	188	189	190	191	192	193	194	195	196	197	198	199	200	201	202	203
Lachemilla diplophylla	.	.	.	.	.	.	.	.	.	.	.	.	.	.	.	.	.	.	.	.	.	.	.	.	.	.	.	.	.
Lachemilla pinnata	.	.	.	.	.	.	.	.	.	.	.	.	.	.	.	.	.	.	.	.	.	.	.	.	.	.	.	.	.
Lagenophora nudicaulis	.	.	.	.	.	.	.	.	.	.	.	.	.	.	.	.	.	.	.	.	.	.	.	.	.	.	.	.	.
Lemna minuta	.	.	.	.	.	.	.	.	.	.	.	.	.	.	.	.	.	.	.	.	.	.	.	.	.	.	.	.	.
Leptinella scariosa	.	.	.	.	.	.	.	.	.	.	.	.	.	.	.	.	.	.	.	.	.	.	.	.	.	.	.	.	.
Leucheria candidissima	.	.	.	.	.	.	.	.	.	.	.	.	.	.	.	.	.	.	.	.	.	.	.	.	.	.	.	.	.
Leucheria nutans	.	.	.	1	.	.	.	.	.	.	.	.	.	.	.	.	.	.	.	.	.	.	1	.	.	.	.	.	.
Lilaea scilloides	.	.	.	.	.	.	.	.	.	.	.	.	.	.	.	.	.	.	.	.	.	.	.	.	.	.	.	.	.
Lilaeopsis macloviana	.	.	.	.	.	.	.	.	.	.	.	.	.	.	.	.	.	.	.	.	.	.	.	.	.	.	.	.	.
Limosella australis	.	.	.	.	.	.	.	.	.	.	.	.	.	.	.	.	.	.	.	.	.	.	.	.	.	.	.	.	.
Lobelia oligophylla	.	1	.	.	1	.	.	.	1	1	.	.	1	1	1	1	1	1	1	1	1	.	1	.	.	1	1	.	1
Luzula brachyphylla	.	.	.	.	.	.	.	.	.	.	.	.	.	.	.	.	.	.	.	.	.	.	.	.	.	.	.	.	.
Luzula chilensis	.	.	.	.	.	.	.	.	.	.	.	.	.	.	.	.	.	.	.	.	.	.	.	.	.	.	.	.	.
Luzula racemosa	1	.	1	1	.	.	.	.	1	.	.	1	.	.	.	.	.	.	.	.	.	.	1	.	.	.	.	.	.
Luzula vulcanica	.	.	.	.	.	.	.	.	.	.	.	.	.	.	.	.	.	.	.	.	.	.	.	.	.	.	.	.	.
Lysipomia pumila	.	.	.	.	.	.	.	.	.	.	.	.	.	.	.	.	.	.	.	.	.	.	.	.	.	.	.	.	.
Marsippospermum philippii	.	.	.	.	.	.	.	.	.	.	.	.	.	.	.	.	.	.	.	.	.	.	.	.	.	.	.	.	.
Marsippospermum reichei	.	.	.	.	.	.	.	.	.	.	.	.	.	.	.	.	.	.	.	.	.	.	.	.	.	.	.	.	.
Montia fontana	.	.	.	.	.	.	.	.	.	.	.	.	.	.	.	.	.	.	.	.	.	.	.	.	.	.	.	.	.
Muhlenbergia asperifolia	.	.	.	.	.	.	.	.	.	.	.	.	.	.	.	.	.	.	.	.	.	.	.	.	.	.	.	.	.
Myriophyllum quitense	.	.	.	.	.	.	1	.	1	1	.	.	.	.	.	.	.	.	.	.	.	.	.	.	.	.	.	.	.
Myrosmodes nervosa	.	.	.	.	.	.	.	.	.	.	.	.	.	.	.	.	.	.	.	.	.	.	.	.	.	.	.	.	.
Myrosmodes paludosa	.	.	.	.	.	.	.	.	.	.	.	.	.	.	.	.	.	.	.	.	.	.	.	.	.	.	.	.	.
Myrteola nummularia	.	.	.	.	.	.	.	.	.	.	.	.	.	.	.	.	.	.	.	.	.	.	.	.	.	.	.	.	.
Nanodea muscosa	.	.	.	.	.	.	.	.	.	.	.	.	.	.	.	.	.	.	.	.	.	.	.	.	.	.	.	.	.
Neobartsia crenoloba	.	.	.	.	.	.	.	.	.	.	.	.	.	.	.	.	.	.	.	.	.	.	.	.	.	.	.	.	.
Neobartsia pedicularoides	.	.	.	.	.	.	.	.	.	.	.	.	.	.	.	.	.	.	.	.	.	.	.	.	.	.	.	.	.
Neobartsia peruviana	.	.	.	.	.	.	.	.	.	.	.	.	.	.	.	.	.	.	.	.	.	.	.	.	.	.	.	.	.
Nertera granadensis	.	.	.	.	.	.	.	.	.	.	.	.	.	.	.	.	.	.	.	.	.	.	.	.	.	.	.	.	.
Nicoraepoa andina	.	.	.	.	.	.	.	.	.	.	.	.	.	.	.	.	.	.	.	.	.	.	.	.	.	.	.	.	.
Nicoraepoa pugionifolia	.	.	.	.	.	.	.	.	.	.	.	.	.	.	.	.	.	.	.	.	.	.	.	.	.	.	.	.	.
Nicoraepoa subenervis	.	.	.	.	.	.	.	.	.	.	.	.	.	.	.	.	.	.	.	.	.	.	.	.	.	.	.	.	.

Bog	175	176	177	178	179	180	181	182	183	184	185	186	187	188	189	190	191	192	193	194	195	196	197	198	199	200	201	202	203
Nitrophila australis	.	.	.	.	.	.	.	.	.	.	.	.	.	.	.	.	.	.	.	.	.	.	.	.	.	.	.	.	.
Nothofagus antarctica	.	.	.	.	.	.	.	.	.	.	.	.	.	.	.	.	.	.	.	.	.	.	.	.	.	.	.	.	.
Nototriche rugosa	.	.	.	.	.	.	.	.	.	.	.	.	.	.	.	.	.	.	.	.	.	.	.	.	.	.	.	.	.
Ochetophila nana	1	1	1	1	.	1	1	1	.	.	1	1	.	1	1	.	.	.	1	1	1	1	1	.	1	1	.	.	.
Olsynium junceum	.	.	.	.	.	.	.	.	.	.	.	.	.	.	.	.	.	.	.	.	.	.	.	.	.	.	.	.	.
Oreobolus obtusangulus	.	.	.	.	.	.	.	.	.	.	.	1	.	.	.	.	.	.	.	.	.	.	.	.	.	.	.	.	.
Oritrophium limnophilum	.	.	.	.	.	.	.	.	.	.	.	.	.	.	.	.	.	.	.	.	.	.	.	.	.	.	.	.	.
Osmorhiza glabrata	.	.	.	.	.	.	.	.	.	.	.	.	.	.	.	.	.	.	.	.	.	.	.	.	.	.	.	.	.
Ourisia alpina	.	.	.	.	.	.	.	.	.	.	.	.	.	.	.	.	.	.	.	.	.	.	.	.	.	.	.	.	.
Ourisia muscosa	.	.	.	.	.	.	.	.	.	.	.	.	.	.	.	.	.	.	.	.	.	.	.	.	.	.	.	.	.
Ourisia ruelloides	.	.	.	.	.	.	.	.	.	.	.	.	.	.	.	.	.	.	.	.	.	.	.	.	.	.	.	.	.
Oxychloe andina	.	.	.	.	.	.	.	.	.	.	.	.	.	.	.	.	.	.	.	.	.	.	.	.	.	.	.	.	.
Oxychloe bisexualis	.	.	.	.	.	.	.	.	.	.	.	.	.	.	.	.	.	.	.	.	.	.	.	.	.	.	.	.	.
Oxychloe castellanosii	.	.	.	.	.	.	.	.	.	.	.	.	.	.	.	.	.	.	.	.	.	.	.	.	.	.	.	.	.
Oxychloe haumaniana	.	.	.	.	.	.	.	.	.	.	.	.	.	.	.	.	.	.	.	.	.	.	.	.	.	.	.	.	.
Oxychloe mendocina	.	.	.	.	.	1	1	.	.	.	.	.	.	.	.	.	.	.	.	.	1	.	.	1	.	.	.	1	.
Patosia clandestina	1	1	1	1	1	1	1	1	1	1	1	1	1	1	1	.	1	1	1	1	1	1	1	1	1	1	.	.	1
Perezia capito	.	.	.	.	.	.	.	.	.	.	.	.	.	.	.	.	.	.	.	.	.	.	.	.	.	.	.	.	.
Perezia delicata	.	.	.	.	.	.	.	.	.	.	.	.	.	.	.	.	.	.	.	.	.	.	.	.	.	.	.	.	.
Perezia fonkii	.	.	.	.	.	.	.	.	.	.	.	.	.	.	.	.	.	.	.	.	.	.	.	.	.	.	.	.	.
Perezia pedicularidifolia	.	.	.	.	.	.	.	.	.	.	.	.	.	.	.	.	.	.	.	.	.	.	.	.	.	.	.	.	.
Perezia pinnatifida	.	.	.	.	.	.	.	.	.	.	.	.	.	.	.	.	.	.	.	.	.	.	.	.	.	.	.	.	.
Petroravenia friesii	.	.	.	.	.	.	.	.	.	.	.	.	.	.	.	.	.	.	.	.	.	.	.	.	.	.	.	.	.
Petroravenia werdermannii	.	.	.	.	.	.	.	.	.	.	.	.	.	.	.	.	.	.	.	.	.	.	.	.	.	.	.	.	.
Phleum alpinum	1	1	.	1	1	1	1	1	.	.	1	1	.	.	.	.	.	.	.	.	.	1	1	.	.	.	.	.	.
Phylloscirpus acaulis	.	.	.	.	.	.	1	1	.	.	.	.	1	1	1	1	1	1	1	1	1	.	.	.	.	1	.	1	1
Phylloscirpus boliviensis	.	.	.	.	.	.	.	.	.	.	.	.	.	.	.	.	.	.	.	.	.	.	.	.	.	.	.	.	.
Phylloscirpus deserticola	.	.	.	.	.	.	.	.	.	.	.	.	.	.	.	.	.	.	.	.	.	.	.	.	.	.	.	.	.
Pinguicula antarctica	.	.	.	.	.	.	.	.	.	.	.	.	.	.	.	.	.	.	.	.	.	.	.	.	.	.	.	.	.
Plantago barbata	1	1	1	1	1	1	1	1	1	1	.	1	1	1	.	1	1	1	.	1	.	1	1	.	.	1	1	1	1
Plantago rigida	.	.	.	.	.	.	.	.	.	.	.	.	.	.	.	.	.	.	.	.	.	.	.	.	.	.	.	.	.
Plantago tubulosa	.	.	.	.	.	.	.	.	.	.	.	.	.	.	.	.	.	.	.	.	.	.	.	.	.	.	.	.	.

Bog	175	176	177	178	179	180	181	182	183	184	185	186	187	188	189	190	191	192	193	194	195	196	197	198	199	200	201	202	203
Plantago uniglumis	.	.	.	.	.	.	.	.	.	.	.	.	.	.	.	.	.	.	.	.	.	.	.	.	.	.	.	.	.
Poa alopecurus	.	.	.	.	.	.	.	.	.	.	.	.	.	.	.	.	.	.	.	.	.	.	.	.	.	.	.	.	.
Poa hachadoensis	.	.	.	.	.	.	.	.	.	.	.	.	.	.	.	.	.	.	.	.	.	.	.	.	.	.	.	.	.
Poa perligulata	.	.	.	.	.	.	.	.	.	.	.	.	.	.	.	.	.	.	.	.	.	.	.	.	.	.	.	.	.
Polypogon interruptus	.	.	.	.	.	.	.	.	.	.	.	.	.	.	.	.	.	.	.	.	.	.	.	.	.	.	.	.	.
Primula magellanica	.	.	.	.	.	.	.	.	.	.	.	.	.	.	.	.	.	.	.	.	.	.	.	.	.	.	.	.	.
Puccinellia frigida	.	.	.	.	.	.	.	.	.	.	.	.	.	.	.	.	.	.	.	.	.	.	.	.	.	.	.	.	.
Quinchamalium chilense	.	.	.	.	.	.	.	.	.	.	.	.	.	.	.	.	.	.	.	.	.	.	.	.	.	.	.	.	.
Ranunculus breviscapus	.	.	.	.	.	.	.	.	.	.	.	.	.	.	.	.	.	.	.	.	.	.	.	.	.	.	.	.	.
Ranunculus fuegianus	.	.	.	.	.	.	.	.	.	.	.	.	.	.	.	.	.	.	.	.	.	.	.	.	.	.	.	.	.
Ranunculus mandonianus	.	.	.	.	.	.	.	.	.	.	.	.	.	.	.	.	.	.	.	.	.	.	.	.	.	.	.	.	.
Ranunculus peduncularis	1	1	.	1	1	1	1	1	1	1	1	1	.	.	.	.	.	.	.	.	.	.	1	.	.	.	.	.	.
Ranunculus trichophyllus	.	.	.	.	.	.	.	.	.	.	.	.	.	.	.	.	.	.	.	.	.	.	.	.	.	.	.	.	.
Halerpestes uniflora	.	.	.	.	.	.	.	.	1	1	.	.	1	.	.	1	1	1	1	1	.	1	.	.	1	.	.	.	.
Rubus geoides	.	.	.	.	.	.	.	.	.	.	.	.	.	.	.	.	.	.	.	.	.	.	.	.	.	.	.	.	.
Rumex magellanicus	.	.	.	.	.	.	.	.	.	.	.	.	.	.	.	.	.	.	.	.	.	.	.	.	.	.	.	.	.
Rytidosperma lechleri	.	.	.	.	.	.	.	.	.	.	.	.	.	.	.	.	.	.	.	.	.	.	.	.	.	.	.	.	.
Sarcocornia pulvinata	.	.	.	.	.	.	.	.	.	.	.	.	.	.	.	.	.	.	.	.	.	.	.	.	.	.	.	.	.
Schoenoplectus pungens	.	.	.	.	.	.	.	.	.	.	.	.	.	.	.	.	.	.	.	.	.	.	.	.	.	.	.	.	.
Schoenus andinus	.	.	.	.	.	.	.	.	.	.	.	.	.	.	.	.	.	.	.	.	.	.	.	.	.	.	.	.	.
Senecio breviscapus	1	.	.	1	.	.	1	1	1	.	.	1	.	.	.	.	.	.	.	.	.	.	1	.	.	.	.	.	.
Senecio diemii	.	.	.	.	.	.	.	.	.	.	.	.	.	.	.	.	.	.	.	.	.	.	.	.	.	.	.	.	.
Senecio fistulosus	.	.	.	.	.	.	.	.	.	.	.	.	.	.	.	.	.	.	.	.	.	.	.	.	.	.	.	.	.
Senecio parodii	.	.	.	.	.	.	.	.	.	.	.	.	.	.	.	.	.	.	.	.	.	.	.	.	.	.	.	.	.
Senecio peteroanus	.	.	.	.	.	.	.	.	.	.	.	.	.	.	.	.	.	.	.	.	.	.	.	.	.	.	.	.	.
Senecio serratifolius	.	.	.	.	.	.	.	.	.	.	.	.	.	.	.	.	.	.	.	.	.	.	.	.	.	.	.	.	.
Senecio trifurcatus	.	.	.	.	.	.	.	.	.	.	.	.	.	.	.	.	.	.	.	.	.	.	.	.	.	.	.	.	.
Sisyrinchium chilense	.	.	.	.	.	.	.	.	.	.	.	.	.	.	.	.	.	.	.	.	.	.	.	.	.	.	.	.	.
Sisyrinchium patagonicum	.	.	.	.	.	.	.	.	.	.	.	.	.	.	.	.	.	.	.	.	.	.	.	.	.	.	.	.	.
Sisyrinchium pearcei	.	.	.	.	.	.	.	.	.	.	.	.	.	.	.	.	.	.	.	.	.	.	.	.	.	.	.	.	.
Stellaria debilis	.	1	1	.	.	.	.	.	1	1	1	.	1	.	.	1	.	.	1	1	.	1	.	.	1	.	1	.	.
Stuckenia filiformis	.	.	.	.	.	.	.	.	.	.	.	.	.	.	.	.	.	.	.	.	.	.	.	.	.	.	.	.	.

Bog	175	176	177	178	179	180	181	182	183	184	185	186	187	188	189	190	191	192	193	194	195	196	197	198	199	200	201	202	203
Stuckenia striata	.	.	.	.	.	.	.	.	.	.	.	.	.	.	.	.	.	.	.	.	.	.	.	.	.	.	.	.	.
Symphyotrichum peteroanum	.	.	.	.	.	.	.	.	.	.	.	.	.	.	.	.	.	.	.	.	.	.	.	.	.	.	.	.	.
Symphyotrichum vahlii	.	1	1	1	.	.	.	.	.	.	.	1	.	.	.	1	.	.	.	.	.	.	1	.	.	.	.	.	.
Tetroncium magellanicum	.	.	.	.	.	.	.	.	.	.	.	.	.	.	.	.	.	.	.	.	.	.	.	.	.	.	.	.	.
Tribeles australis	.	.	.	.	.	.	.	.	.	.	.	.	.	.	.	.	.	.	.	.	.	.	.	.	.	.	.	.	.
Trifolium amabile	.	.	.	.	.	.	.	.	.	.	.	.	.	.	.	.	.	.	.	.	.	.	.	.	.	.	.	.	.
Trifolium polymorphum	.	.	.	.	.	.	.	.	.	.	.	.	.	.	.	.	.	.	.	.	.	.	.	.	.	.	.	.	.
Triglochin concinna	.	.	.	.	.	.	.	.	.	.	.	.	.	.	.	.	.	.	.	.	.	.	.	.	.	.	.	.	.
Triglochin palustris	.	.	.	.	.	.	.	.	.	.	.	.	.	1	.	1	1	1	.	.	.	.	.	.	.	.	.	.	1
Triglochin striata	.	.	.	.	.	.	.	.	.	.	.	.	.	.	.	.	.	.	.	.	.	.	.	.	.	.	.	.	.
Trisetum caudulatum	.	.	.	.	.	.	.	.	.	.	.	.	.	.	.	.	.	.	.	.	.	.	.	.	.	.	.	.	.
Trisetum preslei	.	.	.	.	.	.	.	.	.	.	.	.	.	.	.	.	.	.	.	.	.	.	.	.	.	.	.	.	.
Koeleria spicata	.	.	.	.	.	.	.	.	.	.	.	.	.	.	.	.	.	.	.	.	.	.	.	.	.	.	.	.	.
Utricularia gibba	.	.	.	.	.	.	.	.	.	.	.	.	.	.	.	.	.	.	.	.	.	.	.	.	.	.	.	.	.
Vahlodea atropurpurea	.	.	.	.	.	.	.	.	.	.	.	.	.	.	.	.	.	.	.	.	.	.	.	.	.	.	.	.	.
Valeriana fonckii	.	.	.	.	.	.	.	.	.	.	.	.	.	.	.	.	.	.	.	.	.	.	.	.	.	.	.	.	.
Valeriana macrorhiza	1	1	.	1	.	1	.	.	.	1	.	1	.	.	.	.	.	.	.	.	.	.	1	.	.	.	.	.	.
Viola pygmaea	.	.	.	.	.	.	.	.	.	.	.	.	.	.	.	.	.	.	.	.	.	.	.	.	.	.	.	.	.
Werneria apiculata	.	.	.	.	.	.	.	.	.	.	.	.	.	.	.	.	.	.	.	.	.	.	.	.	.	.	.	.	.
Werneria pinnatifida	.	.	.	.	.	.	.	.	.	.	.	.	.	.	.	.	.	.	.	.	.	.	.	.	.	.	.	.	.
Werneria pygmaea	.	.	.	1	.	1	1	1	1	1	.	.	1	1	1	1	1	1	1	1	1	1	.	1	.	1	1	.	1
Werneria solivifolia	.	.	.	.	.	.	.	.	.	.	.	.	.	.	.	.	.	.	.	.	.	.	.	.	.	.	.	.	.
Werneria spathulata	.	.	.	.	.	.	.	.	.	.	.	.	.	.	.	.	.	.	.	.	.	.	.	.	.	.	.	.	.
Xenophyllum incisum	.	.	.	.	.	.	.	.	.	.	.	.	.	.	.	.	.	.	.	.	.	.	.	.	.	.	.	.	.
Zameioscirpus atacamensis	.	.	.	.	.	.	.	.	.	.	.	.	.	.	.	.	.	.	.	.	.	.	.	.	.	.	.	.	.
Zameioscirpus gaimardiodes	.	.	.	.	.	.	.	.	.	.	.	.	.	.	.	.	.	.	.	.	.	.	.	.	.	.	.	.	.
Zameioscirpus muticus	1	1	1	1	.	1	1	1	1	1	1	1	.	.	.	.	.	.	.	.	.	1	1	1	1	.	1	.	.

Bog	204	205	206	207	208	209	210	211	212	213	214	215	216	217	218	219	220	221	222	223	224	225	226	227	228	229	230	231	232
**Operational zone**	**S**	**S**	**S**	**S**	**S**	**S**	**S**	**S**	**S**	**S**	**S**	**S**	**S**	**S**	**S**	**S**	**S**	**S**	**S**	**S**	**S**	**S**	**S**	**S**	**S**	**N**	**N**	**N**	**N**
**Cluster**	**7**	**7**	**7**	**7**	**7**	**2**	**2**	**7**	**7**	**7**	**2**	**2**	**2**	**6**	**7**	**2**	**7**	**7**	**7**	**7**	**7**	**7**	**7**	**7**	**7**	**1**	**1**	**1**	**1**
**Bioregion**	**S**	**S**	**S**	**S**	**S**	**S**	**S**	**S**	**S**	**S**	**S**	**S**	**S**	**S**	**S**	**S**	**S**	**S**	**S**	**S**	**S**	**S**	**S**	**S**	**S**	**N**	**N**	**N**	**N**
**Longitude**	**-70.39**	**-70.4**	**-70.4**	**-70.41**	**-70.53**	**-70.52**	**-70.85**	**-71.12**	**-71.1**	**-71.12**	**-70.12**	**-70.1**	**-70.12**	**-70.12**	**-70.55**	**-70.54**	**-70.52**	**-70.85**	**-70.88**	**-70.83**	**-70.83**	**-70.83**	**-70.84**	**-71.1**	**-71.12**	**-69.01**	**-66.61**	**-68.94**	**-68.94**
**Latitude**	**-35.98**	**-35.95**	**-35.98**	**-35.97**	**-35.3**	**-35.2**	**-38.72**	**-37.85**	**-37.83**	**-37.45**	**-37.08**	**-37.12**	**-37.08**	**-37.07**	**-36.69**	**-36.54**	**-36.68**	**-38.72**	**-38.67**	**-38.6**	**-38.6**	**-38.63**	**-38.62**	**-37.84**	**-37.85**	**-18.12**	**-18.87**	**-17.94**	**-17.95**
Acaena antarctica	.	.	.	.	.	.	.	.	.	.	.	.	.	.	.	.	.	.	.	.	.	.	.	.	.	.	.	.	.
Acaena macrocephala	.	.	.	.	.	.	.	.	.	.	.	.	.	.	.	.	.	.	.	.	.	.	.	.	.	.	.	.	.
Acaena magellanica	.	1	.	1	1	.	.	1	.	.	.	.	.	.	.	.	.	1	.	.	.	1	1	.	1	.	.	.	.
Acaena ovalifolia	.	.	.	.	.	.	.	.	.	.	.	.	.	.	.	.	.	.	.	.	.	.	.	.	.	.	.	.	.
Acaena pinnatifida	.	.	.	.	.	.	.	.	.	.	.	.	.	.	.	.	.	.	.	.	.	.	.	.	.	.	.	.	.
Adesmia retusa	.	.	.	.	.	.	.	.	.	.	.	.	.	.	.	.	.	.	.	.	.	.	.	.	.	.	.	.	.
Agrostis breviculmis	.	.	.	.	.	.	.	.	.	.	.	.	.	.	.	.	.	.	.	.	.	.	.	.	.	.	.	.	.
Agrostis imberbis	.	.	.	.	.	.	.	.	.	.	.	.	.	.	.	.	.	.	.	.	.	.	.	.	.	.	.	.	.
Agrostis meyenii	.	.	.	.	.	.	.	.	.	.	.	.	.	.	.	.	.	.	.	.	.	.	.	.	.	.	.	.	.
Agrostis perennans	.	.	.	.	.	.	.	.	.	.	.	.	.	.	.	.	.	.	.	.	.	.	.	.	.	.	.	.	.
Alchemilla pinnata	.	.	.	.	.	.	.	.	.	.	.	.	.	.	.	.	.	.	.	.	.	.	.	.	.	.	.	.	.
Alopecurus magellanicus	.	.	.	1	1	.	.	.	.	.	.	.	.	.	.	.	1	.	.	.	.	.	.	.	.	.	.	.	.
Amphiscirpus nevadensis	.	.	.	.	.	.	.	.	.	.	.	.	.	.	.	.	.	.	.	.	.	.	.	.	.	.	.	.	.
Anagallis alternifolia	1	1	.	1	.	.	.	.	1	1	.	.	.	.	1	1	.	1	1	1	.	1	1	.	1	.	.	.	.
Antennaria chilensis	.	.	.	.	.	.	.	.	.	.	.	.	.	.	.	.	.	.	.	.	.	.	.	.	.	.	.	.	.
Anthoxanthum redolens	.	.	.	.	.	.	.	.	.	.	.	.	.	.	.	.	.	.	.	.	.	.	.	.	.	.	.	.	.
Apium panul	.	.	.	.	.	.	.	.	.	.	.	.	.	.	.	.	.	.	.	.	.	.	.	.	.	.	.	.	.
Arenaria rivularis	.	.	.	.	.	.	.	.	.	.	.	.	.	.	.	.	.	.	.	.	.	.	.	.	.	.	.	.	.
Arenaria serpens	.	.	.	.	.	.	.	.	.	.	.	.	.	.	.	.	.	.	.	.	.	.	.	.	.	.	.	.	.
Arjona pusilla	.	.	.	.	.	.	.	.	.	.	.	.	.	.	.	.	.	.	.	.	.	.	.	.	.	.	.	.	.
Astragalus bustillosii	.	.	.	.	.	.	.	.	.	.	.	.	.	.	.	.	.	.	.	.	.	.	.	.	.	.	.	.	.
Astragalus micranthellus	.	.	.	.	.	.	.	.	.	.	.	.	.	.	.	.	.	.	.	.	.	.	.	.	.	.	.	.	.
Azolla filiculoides	.	.	.	.	.	.	.	.	.	.	.	.	.	.	.	.	.	.	.	.	.	.	.	.	.	.	.	.	.
Azorella boelckei	.	.	.	.	.	.	.	.	.	.	.	.	.	.	.	.	.	.	.	1	.	.	1	.	.	.	.	.	.

**Bog**	**204**	**205**	**206**	**207**	**208**	**209**	**210**	**211**	**212**	**213**	**214**	**215**	**216**	**217**	**218**	**219**	**220**	**221**	**222**	**223**	**224**	**225**	**226**	**227**	**228**	**229**	**230**	**231**	**232**
Azorella burkartii	.	.	.	.	.	.	.	.	.	.	.	.	.	.	.	.	.	.	.	.	.	.	.	.	.	.	.	.	.
Azorella cryptantha	.	.	.	.	.	.	.	.	.	.	.	.	.	.	.	.	.	.	.	.	.	.	.	.	.	.	.	.	.
Azorella lycopodioides	.	.	.	.	.	.	.	.	.	.	.	.	.	.	.	.	.	.	.	.	.	.	.	.	.	.	.	.	.
Azorella trifoliolata	.	.	.	.	.	.	1	.	.	.	.	.	.	.	.	.	.	1	.	.	.	.	.	.	.	.	.	.	.
Baccharis acaulis	.	.	.	.	.	.	.	.	.	.	.	.	.	.	.	.	.	.	.	.	.	.	.	.	.	.	.	.	.
Baccharis caespitosa	.	.	.	.	.	.	.	.	.	.	.	.	.	.	.	.	.	.	.	.	.	.	.	.	.	.	.	.	.
Baccharis magellanica	.	.	.	.	.	.	.	.	.	.	.	.	.	.	.	.	.	.	.	.	.	.	.	.	.	.	.	.	.
Belloa chilensis	.	.	.	.	.	.	.	.	.	.	.	.	.	.	.	.	.	.	.	.	.	.	.	.	.	.	.	.	.
Bromus catharticus	.	.	.	.	.	.	.	.	.	.	.	.	.	.	.	.	.	.	.	.	.	.	.	.	.	.	.	.	.
Calandrinia acaulis	.	.	.	.	.	.	.	.	.	.	.	.	.	.	.	.	.	.	.	.	.	.	.	.	.	.	.	.	.
Calandrinia compacta	.	.	.	.	.	.	.	.	.	.	.	.	.	.	.	.	.	.	.	.	.	.	.	.	.	1	1	1	.
Calceolaria biflora	.	.	.	.	.	.	.	.	.	.	.	.	.	.	.	.	.	.	.	.	.	.	.	.	.	.	.	.	.
Calceolaria cana	.	.	.	.	.	.	.	.	.	.	.	.	.	.	.	.	.	.	.	.	.	.	.	.	.	.	.	.	.
Calceolaria corymbosa	.	.	.	.	.	.	.	.	.	.	.	.	.	.	.	.	.	.	.	.	.	.	.	.	.	.	.	.	.
Calceolaria filicaulis	.	.	.	.	.	.	.	.	.	.	.	.	.	.	.	.	.	.	.	.	.	.	.	.	.	.	.	.	.
Callitriche lechleri	.	.	.	.	.	.	.	.	.	.	.	.	.	.	.	.	.	.	.	.	.	.	.	.	.	.	.	.	.
Caltha appendiculata	.	.	.	.	.	.	.	.	1	.	.	.	.	.	.	1	1	.	.	1	.	.	.	1	.	.	.	.	.
Caltha sagittata	1	1	1	1	1	1	1	1	1	1	.	.	.	.	1	.	.	1	1	1	1	1	1	1	1	.	.	.	.
Cardamine cordata	.	.	.	1	.	.	.	.	.	1	.	.	.	.	.	.	1	.	1	.	.	.	1	.	.	.	.	.	.
Cardamine glacialis	.	.	.	.	.	.	.	.	.	.	.	.	.	.	.	.	.	.	.	.	.	.	.	.	.	.	.	.	.
Cardamine tenuirostris	.	.	.	.	.	.	.	.	.	.	.	.	.	.	.	.	.	.	.	.	.	.	.	.	.	.	.	.	.
Cardamine volckmannii	.	.	.	.	.	.	.	.	.	.	.	.	.	.	.	.	.	.	.	.	.	.	.	.	.	.	.	.	.
Carex acaulis	.	.	.	.	.	.	1	.	.	.	.	.	.	.	.	.	.	1	.	.	.	.	.	.	.	.	.	.	.
Carex atropicta	1	.	.	.	1	.	.	.	.	.	.	.	1	1	.	.	.	.	1	.	.	1	1	.	1	.	.	.	.
Carex banksii	.	.	.	.	1	.	.	.	.	1	.	.	.	.	.	.	.	.	1	1	1	1	1	1	.	.	.	.	.
Carex caduca	.	.	.	.	.	.	.	.	.	.	.	.	.	.	.	.	.	.	.	.	.	.	.	.	.	.	.	.	.
Carex decidua	.	.	.	.	.	.	.	.	.	.	.	.	.	.	.	.	.	.	.	.	.	.	.	.	.	.	.	.	.
Carex fuscula	.	.	.	.	.	.	.	.	.	.	.	.	.	.	.	.	.	1	1	.	1	1	.	.	.	.	.	.	.
Carex gayana	.	.	.	.	1	1	.	1	.	1	1	1	1	.	1	.	.	1	1	1	1	1	1	.	.	.	.	.	.
Carex hypoleucos	.	.	.	.	.	.	.	.	.	.	.	.	.	.	.	.	.	.	.	.	.	.	.	.	.	.	.	.	.
Carex macloviana	.	1	1	1	1	1	.	.	.	.	.	.	1	1	.	.	1	.	.	.	.	.	.	.	.	.	.	.	.
Carex magellanica	.	.	.	.	.	.	.	.	.	1	.	.	.	.	.	.	.	.	.	.	.	.	.	.	.	.	.	.	.
Carex malmei	.	.	.	.	.	.	.	.	.	.	.	.	.	.	.	.	.	.	.	.	.	.	.	.	.	.	.	.	.
Carex maritima	.	.	.	.	.	.	.	.	.	.	.	.	.	.	.	.	.	.	.	.	.	.	.	.	.	1	.	1	1
Carex microglochin	.	.	.	.	.	.	.	.	.	.	.	.	.	.	.	.	.	.	.	.	.	.	.	.	.	.	.	.	.
Carex pleioneura	.	.	.	.	.	.	.	.	.	.	.	.	.	.	.	.	.	.	.	.	.	.	.	.	.	.	.	.	.
Carex ruthsatzae	.	.	.	.	.	.	.	.	.	.	.	.	.	.	.	.	.	.	.	.	.	.	.	.	.	.	.	.	.
Carex vallis-pulchrae	.	.	.	.	.	.	1	.	.	.	.	.	.	1	.	1	.	1	.	.	.	.	.	.	.	.	.	.	1

Bog	204	205	206	207	208	209	210	211	212	213	214	215	216	217	218	219	220	221	222	223	224	225	226	227	228	229	230	231	232
Carpha schoenoides	.	.	.	.	.	.	.	.	.	.	.	.	.	.	.	.	.	.	.	.	.	.	.	.	.	.	.	.	.
Castilleja pumila	.	.	.	.	.	.	.	.	.	.	.	.	.	.	.	.	.	.	.	.	.	.	.	.	.	1	1	1	.
Catabrosa werdermannii	.	.	.	.	.	.	.	.	.	.	.	.	.	.	.	.	.	.	.	.	.	.	.	.	.	.	.	.	.
Cerastium humifusum	.	.	.	.	.	.	.	.	.	.	.	.	.	.	.	.	.	.	.	.	.	.	.	.	.	.	.	.	.
Cerastium montioides	.	.	.	.	.	.	.	.	.	.	.	.	.	.	.	.	.	.	.	.	.	.	.	.	.	.	.	.	.
Chiliotrichum diffusum	1	1	1	1	1	.	.	1	1	.	.	.	1	.	.	1	.	1	1	1	1	1	1	1	1	.	.	.	.
Chusquea culeou	.	.	.	.	.	.	.	.	.	.	.	.	.	.	.	.	.	.	1	.	.	.	.	.	.	.	.	.	.
Colobanthus quitensis	.	.	.	.	.	.	.	.	.	.	1	.	1	.	.	.	1	.	.	.	.	.	1	.	.	1	1	1	.
Cortaderia egmontiana	.	.	.	.	.	.	.	.	.	.	.	.	.	.	.	.	.	.	.	.	.	.	.	.	.	.	.	.	.
Cotula mexicana	.	.	.	.	.	.	.	.	.	.	.	.	.	.	.	.	.	.	.	.	.	.	.	.	.	.	1	1	.
Crassula peduncularis	.	.	.	.	.	.	.	.	.	.	.	.	.	.	.	.	.	.	.	.	.	.	.	.	.	.	.	.	.
Cuatrecasasiella argentina	.	.	.	.	.	.	.	.	.	.	.	.	.	.	.	.	.	.	.	.	.	.	.	.	.	1	1	.	.
Deschampsia antarctica	.	.	.	.	.	.	.	.	.	.	.	.	.	.	.	.	.	.	.	.	.	.	.	.	.	.	.	.	.
Deschampsia caespitosa	.	1	.	.	1	1	.	1	.	1	1	.	1	.	1	1	1	1	.	1	1	1	1	.	.	.	.	.	.
Deschampsia patula	1	.	1	.	.	.	1	.	.	.	.	.	.	.	.	.	.	1	.	.	.	1	1	.	.	.	.	.	.
Cinnagrostis brevifolia	.	.	.	.	.	.	.	.	.	.	.	.	.	.	.	.	.	.	.	.	.	.	.	.	.	.	.	.	.
Deschampsia chrysantha	.	.	.	.	.	.	.	.	.	.	.	.	.	.	.	.	.	.	.	.	.	.	.	.	.	1	.	.	1
Cinnagrostis chrysophylla	.	.	.	.	.	.	.	.	.	.	.	.	.	.	.	.	.	.	.	.	.	.	.	.	.	.	.	.	1
Deschampsia chrysostachya	.	.	.	.	.	.	.	.	.	.	.	.	.	.	.	.	.	.	.	.	.	.	.	.	.	.	.	.	.
Deschampsia eminens	.	.	.	.	.	.	.	.	.	.	.	.	.	.	.	.	.	.	.	.	.	.	.	.	.	.	.	.	1
Deschampsia hackelii	.	.	.	.	.	.	.	.	.	.	.	.	.	.	.	.	.	.	.	.	.	.	.	.	.	.	.	.	.
Cinnagrostis minima	.	.	.	.	.	.	.	.	.	.	.	.	.	.	.	.	.	.	.	.	.	.	.	.	.	.	.	.	.
Deschampsia ovata	.	.	.	.	.	.	.	.	.	.	.	.	.	.	.	.	.	.	.	.	.	.	.	.	.	.	.	.	.
Cinnagrostis rigescens	.	.	.	.	.	.	.	.	.	.	.	.	.	.	.	.	.	.	.	.	.	.	.	.	.	.	1	1	1
Cinnagrostis spicigera	.	.	.	.	.	.	.	.	.	.	.	.	.	.	.	.	.	.	.	.	.	.	.	.	.	1	.	.	.
Cinnagrostis velutina	.	.	.	.	.	.	.	.	.	.	.	.	.	.	.	.	.	.	.	.	.	.	.	.	.	.	.	.	.
Cinnagrostis vicunarum	.	.	.	.	.	.	.	.	.	.	.	.	.	.	.	.	.	.	.	.	.	.	.	.	.	.	.	.	.
Distichia filamentosa	.	.	.	.	.	.	.	.	.	.	.	.	.	.	.	.	.	.	.	.	.	.	.	.	.	.	.	.	.
Distichia muscoides	.	.	.	.	.	.	.	.	.	.	.	.	.	.	.	.	.	.	.	.	.	.	.	.	.	1	.	1	1
Distichlis humilis	.	.	.	.	.	.	.	.	.	.	.	.	.	.	.	.	.	.	.	.	.	.	.	.	.	.	.	.	.
Distichlis scoparia	.	.	.	.	.	.	.	.	.	.	.	.	.	.	.	.	.	.	.	.	.	.	.	.	.	.	.	.	.
Distichlis spicata	.	.	.	.	.	.	.	.	.	.	.	.	.	.	.	.	.	.	.	.	.	.	.	.	.	.	.	.	.

**Bog**	**204**	**205**	**206**	**207**	**208**	**209**	**210**	**211**	**212**	**213**	**214**	**215**	**216**	**217**	**218**	**219**	**220**	**221**	**222**	**223**	**224**	**225**	**226**	**227**	**228**	**229**	**230**	**231**	**232**
Draba pusilla	.	.	.	.	.	.	.	.	.	.	.	.	.	.	.	.	.	.	.	.	.	.	.	.	.	.	.	.	.
Eleocharis melanomphala	.	.	.	.	.	.	.	.	.	.	.	.	.	.	.	.	.	.	.	.	.	.	.	.	.	.	.	.	.
Eleocharis pseudoalbibracteata	.	.	.	.	.	.	.	.	.	.	.	.	.	.	.	.	.	.	.	.	.	.	.	.	.	.	.	.	.
Elodea potamogeton	.	.	.	.	.	.	.	.	.	.	.	.	.	.	.	.	.	.	.	.	.	.	.	.	.	.	.	.	.
Empetrum rubrum	1	1	1	1	.	.	.	.	1	.	.	.	.	.	.	.	.	.	.	1	.	.	1	1	1	.	.	.	.
Epilobium australe	.	.	.	.	.	.	.	.	.	.	.	.	.	.	.	.	.	.	.	.	.	.	.	.	.	.	.	.	.
Epilobium barbeyanum	1	.	.	.	1	1	.	.	.	.	.	.	.	.	.	.	1	.	.	.	.	.	1	.	.	.	.	.	.
Epilobium ciliatum	.	.	.	.	.	.	.	.	.	.	.	.	.	.	.	.	.	.	.	.	.	.	.	.	.	.	.	.	.
Epilobium denticulatum	.	.	.	.	.	.	.	.	.	.	.	.	.	.	.	.	.	.	.	.	.	.	.	.	.	.	.	.	.
Epilobium fragile	.	.	.	.	.	.	.	.	.	.	.	.	.	.	.	.	.	.	.	.	.	.	.	.	.	.	.	.	.
Epilobium glaucum	.	.	.	.	.	.	.	.	.	.	.	.	.	.	.	.	.	.	.	.	.	.	.	.	.	.	.	.	.
Epilobium nivale	.	.	.	.	.	.	.	.	.	.	.	.	.	.	.	.	.	.	.	.	.	.	.	.	.	.	.	.	.
Erigeron andicola	.	.	.	.	.	.	.	.	.	.	.	.	.	.	.	.	.	.	.	.	.	.	.	.	.	.	.	.	.
Erigeron leptopetalus	.	.	.	.	.	.	.	.	.	.	.	.	.	.	.	.	.	.	.	.	.	.	.	.	.	.	.	.	.
Erigeron myosotis	.	.	.	.	.	.	.	.	.	.	.	.	.	.	.	.	.	.	.	.	.	.	.	.	.	.	.	.	.
Erigeron patagonicus	.	.	.	.	.	.	.	.	.	.	.	.	.	.	.	.	.	.	.	.	.	.	.	.	.	.	.	.	.
Erythranthe cuprea	.	.	.	.	.	.	1	.	.	1	.	.	.	.	.	.	.	1	.	1	.	1	1	.	.	.	.	.	.
Erythranthe depressa	.	.	.	.	.	.	.	.	.	.	.	.	.	.	.	.	.	.	.	.	.	.	.	.	.	.	.	.	.
Erythranthe glabrata	.	.	.	.	.	.	.	.	.	.	.	.	.	.	.	.	.	.	.	.	.	.	.	.	.	.	1	.	.
Erythranthe lutea	.	.	.	.	1	.	.	.	.	1	.	.	.	.	.	.	.	.	1	.	.	.	.	.	.	.	.	.	.
Escallonia virgata	.	.	.	.	.	.	.	.	.	.	.	.	.	.	.	.	.	.	.	.	.	.	.	.	.	.	.	.	.
Euphrasia antarctica	.	.	.	.	.	.	.	.	.	.	.	.	.	.	.	.	.	.	.	.	.	.	.	.	.	.	.	.	.
Euphrasia chrysantha	.	.	.	.	.	.	.	.	.	.	.	.	.	.	.	.	.	.	.	.	.	.	.	.	.	.	.	.	.
Euphrasia subexserta	1	1	1	1	1	.	.	1	1	.	.	.	.	.	1	.	1	.	.	1	.	1	1	1	1	.	.	.	.
Festuca hypsophila	.	.	.	.	.	.	.	.	.	.	.	.	.	.	.	.	.	.	.	.	.	.	.	.	.	.	.	.	.
Festuca kurtziana	.	.	.	.	.	.	.	.	.	.	.	.	.	.	.	.	.	.	.	.	.	.	.	.	.	.	.	.	.
Festuca lilloi	.	.	.	.	.	.	.	.	.	.	.	.	.	.	.	.	.	.	.	.	.	.	.	.	.	.	.	.	.
Festuca magellanica	.	.	.	.	.	.	.	.	.	.	.	.	.	.	.	.	.	.	.	.	.	.	.	.	.	.	.	.	.
Festuca nardifolia	.	.	.	.	.	.	.	.	.	.	.	.	.	.	.	.	.	.	.	.	.	.	.	.	.	.	.	.	.
Festuca rigescens	.	.	.	.	.	.	.	.	.	.	.	.	.	.	.	.	.	.	.	.	.	.	.	.	.	.	.	.	.
Festuca werdermannii	.	.	.	.	.	.	.	.	.	.	.	.	.	.	.	.	.	.	.	.	.	.	.	.	.	.	.	.	.
Frankenia triandra	.	.	.	.	.	.	.	.	.	.	.	.	.	.	.	.	.	.	.	.	.	.	.	.	.	.	.	.	.
Gamocarpha graminea	.	.	.	.	.	.	.	1	1	1	.	.	.	.	.	.	.	1	1	1	1	1	1	.	1	.	.	.	.
Gamocarpha ventosa	.	.	.	.	.	.	.	.	.	.	.	.	.	.	.	.	.	.	.	.	.	.	.	.	.	.	.	.	.
Gamochaeta chamissonis	.	.	.	.	.	.	.	.	.	.	.	.	.	.	.	.	.	.	.	.	.	.	.	.	.	.	.	.	.

Bog	204	205	206	207	208	209	210	211	212	213	214	215	216	217	218	219	220	221	222	223	224	225	226	227	228	229	230	231	232
Gamochaeta longipedicellata	.	.	.	.	.	.	.	.	.	.	.	.	.	.	.	.	.	.	.	.	.	.	.	.	.	.	.	.	.
Gamochaeta neuquensis	.	.	.	.	.	.	.	.	.	.	.	.	.	.	.	.	.	.	.	.	.	.	.	.	.	.	.	.	.
Gaultheria antarctica	.	.	.	.	.	.	.	.	.	.	.	.	.	.	.	.	.	.	.	.	.	.	.	.	.	.	.	.	.
Gaultheria caespitosa	.	.	.	.	.	.	.	.	.	.	.	.	.	.	.	.	.	.	.	.	.	.	.	.	.	.	.	.	.
Gaultheria pumila	1	1	1	1	1	.	.	.	1	.	.	.	.	.	.	.	1	1	1	1	.	.	1	1	1	.	.	.	.
Gavilea chica	.	.	.	.	.	.	.	.	.	1	.	.	.	.	.	.	.	.	.	1	1	.	.	.	.	.	.	.	.
Gentiana prostrata	.	1	.	.	.	1	1	1	1	.	.	.	.	.	.	.	.	1	1	.	.	1	1	.	1	1	.	1	1
Gentianella fiebrigii	.	.	.	.	.	.	.	.	.	.	.	.	.	.	.	.	.	.	.	.	.	.	.	.	.	.	.	.	.
Gentianella magellanica	1	1	1	1	.	.	.	1	1	1	.	.	.	.	.	.	.	.	1	1	1	1	1	.	1	.	.	.	.
Gentianella multicaulis	.	.	.	.	.	.	.	.	.	.	.	.	.	.	.	.	.	.	.	.	.	.	.	.	.	.	.	.	.
Gentianella ottonis	.	.	.	.	.	.	.	.	.	.	.	.	.	.	.	.	.	.	.	.	.	.	.	.	.	.	.	.	.
Gentianella primuloides	.	.	.	.	.	.	.	.	.	.	.	.	.	.	.	.	.	.	.	.	.	.	.	.	.	.	.	.	.
Gentianella pseudocrassula	.	.	.	.	.	.	.	.	.	.	.	.	.	.	.	.	.	.	.	.	.	.	.	.	.	.	.	.	.
Geranium sessiliflorum	.	.	.	.	.	.	.	.	.	.	.	.	.	.	.	.	.	.	.	.	.	.	.	.	.	.	.	.	.
Gunnera magellanica	.	.	.	.	1	.	.	1	.	1	.	.	.	.	1	.	1	.	1	1	1	1	1	1	.	.	.	.	.
Halenia caespitosa	.	.	.	.	.	.	.	.	.	.	.	.	.	.	.	.	.	.	.	.	.	.	.	.	.	.	.	.	.
Halerpestes cymbalaria	.	.	.	.	.	.	.	.	.	.	.	.	.	.	.	.	.	.	.	.	.	.	.	.	.	.	1	.	.
Halerpestes exilis	.	.	.	.	.	.	.	.	.	.	.	.	.	.	.	.	.	.	.	.	.	.	.	.	.	.	.	.	.
Hieracium antarcticum	.	.	.	.	.	.	.	.	.	.	.	.	.	.	.	.	.	.	.	.	.	.	.	.	.	.	.	.	.
Hordeum comosum	1	1	1	.	1	1	.	.	.	.	1	1	1	.	1	1	1	1	1	.	1	1	.	.	1	.	.	.	.
Hordeum muticum	.	.	.	.	.	.	.	.	.	.	.	.	.	.	.	.	.	.	.	.	.	.	.	.	.	.	.	.	.
Hypochaeris acaulis	.	.	.	.	.	.	.	.	.	.	.	.	.	.	.	.	.	.	.	.	.	.	.	.	.	.	.	.	.
Hypochaeris chondrilloides	.	.	.	.	.	.	.	.	.	.	.	.	.	.	.	.	.	.	.	.	.	.	.	.	.	.	.	.	.
Hypochaeris meyeniana	.	.	.	.	.	.	.	.	.	.	.	.	.	.	.	.	.	.	.	.	.	.	.	.	.	.	.	.	.
Hypochaeris palustris	.	.	.	.	.	.	.	.	.	.	.	.	.	.	.	.	.	.	.	.	.	.	.	.	.	.	.	.	.
Hypochaeris taraxacoides	.	.	.	.	.	.	.	.	.	.	.	.	.	.	.	.	.	.	.	.	.	.	.	.	.	1	1	1	1
Hypochaeris tenerifolia	.	.	.	.	.	.	.	.	.	.	.	.	.	.	.	.	.	.	.	.	.	.	.	.	.	.	.	.	.
Isolepis nigricans	.	.	.	.	.	.	.	.	.	.	.	.	.	.	.	.	.	.	.	.	.	1	1	.	.	.	.	.	.
Isolepsis inundata	.	.	.	.	.	.	.	.	.	.	.	.	.	.	.	.	.	.	.	.	.	.	.	.	.	.	.	.	.
Juncus balticus	1	.	.	.	1	1	.	1	.	1	1	1	1	.	1	1	1	1	.	.	.	.	.	.	1	.	.	.	.
Juncus stipulatus	.	.	.	.	.	.	.	.	.	.	.	.	.	.	.	.	.	.	.	.	.	.	.	.	.	.	1	1	.
Koeleria kurtzii	.	.	.	.	.	.	.	.	.	.	.	.	.	.	.	.	.	.	.	.	.	.	.	.	.	.	.	.	.

**Bog**	**204**	**205**	**206**	**207**	**208**	**209**	**210**	**211**	**212**	**213**	**214**	**215**	**216**	**217**	**218**	**219**	**220**	**221**	**222**	**223**	**224**	**225**	**226**	**227**	**228**	**229**	**230**	**231**	**232**
Lachemilla diplophylla	.	.	.	.	.	.	.	.	.	.	.	.	.	.	.	.	.	.	.	.	.	.	.	.	.	.	1	1	.
Lachemilla pinnata	.	.	.	.	.	.	.	.	.	.	.	.	.	.	.	.	.	.	.	.	.	.	.	.	.	.	1	1	1
Lagenophora nudicaulis	.	.	.	.	.	.	.	.	.	.	.	.	.	.	.	.	.	.	.	.	.	.	.	.	.	.	.	.	.
Lemna minuta	.	.	.	.	.	.	.	.	.	.	.	.	.	.	.	.	.	.	.	.	.	.	.	.	.	.	.	.	.
Leptinella scariosa	.	.	.	.	.	.	.	.	.	.	.	.	.	.	.	.	.	.	.	.	.	.	.	.	.	.	.	.	.
Leucheria candidissima	.	.	.	.	.	.	.	.	.	.	.	.	.	.	.	.	.	.	.	.	.	.	.	.	.	.	.	.	.
Leucheria nutans	.	.	.	.	.	.	.	1	1	1	.	.	.	.	.	.	.	.	.	1	.	.	.	1	.	.	.	.	.
Lilaea scilloides	.	.	.	.	.	.	.	.	.	.	.	.	.	.	.	.	.	.	.	.	.	.	.	.	.	.	.	.	.
Lilaeopsis macloviana	.	.	.	.	.	.	.	.	.	.	.	.	.	.	.	.	.	.	.	.	.	.	.	.	.	1	1	1	.
Limosella australis	.	.	.	.	.	.	.	.	.	.	.	.	.	.	.	.	.	.	.	.	.	.	.	.	.	.	.	.	.
Lobelia oligophylla	.	.	.	.	1	1	.	1	.	1	1	1	1	.	1	1	.	1	1	.	.	1	.	.	.	1	1	1	1
Luzula brachyphylla	.	.	.	.	.	.	.	.	.	.	.	.	.	.	.	.	.	.	.	.	.	.	.	.	.	.	.	.	.
Luzula chilensis	.	.	.	.	.	.	.	.	.	.	.	.	.	.	.	.	.	.	.	.	.	.	.	.	.	.	.	.	.
Luzula racemosa	.	1	1	1	1	.	.	.	.	1	.	.	1	1	.	.	1	.	1	1	.	1	.	1	1	.	.	.	.
Luzula vulcanica	.	.	.	.	.	.	.	.	.	.	.	.	.	.	.	.	.	.	.	.	.	.	.	.	.	.	.	.	.
Lysipomia pumila	.	.	.	.	.	.	.	.	.	.	.	.	.	.	.	.	.	.	.	.	.	.	.	.	.	.	.	.	.
Marsippospermum philippii	.	.	.	.	.	.	.	.	1	.	.	.	.	.	.	.	.	.	.	1	.	.	.	1	.	.	.	.	.
Marsippospermum reichei	.	.	.	.	.	.	.	.	.	.	.	.	.	.	.	.	.	.	.	.	.	.	.	.	.	.	.	.	.
Montia fontana	.	.	.	.	.	.	.	.	.	.	.	.	.	.	.	.	.	.	.	.	.	.	.	.	.	.	.	.	1
Muhlenbergia asperifolia	.	.	.	.	.	.	.	.	.	.	.	.	.	.	.	.	.	.	.	.	.	.	.	.	.	.	.	.	.
Myriophyllum quitense	.	.	.	.	.	1	.	.	.	.	.	.	.	.	.	.	.	1	1	.	.	1	.	.	.	.	.	1	.
Myrosmodes nervosa	.	.	.	.	.	.	.	.	.	.	.	.	.	.	.	.	.	.	.	.	.	.	.	.	.	.	.	.	.
Myrosmodes paludosa	.	.	.	.	.	.	.	.	.	.	.	.	.	.	.	.	.	.	.	.	.	.	.	.	.	.	.	.	.
Myrteola nummularia	.	.	.	.	.	.	.	.	.	.	.	.	.	.	.	.	.	.	.	.	.	.	.	.	.	.	.	.	.
Nanodea muscosa	.	.	.	.	.	.	.	.	.	.	.	.	.	.	.	.	.	.	.	.	.	.	.	.	.	.	.	.	.
Neobartsia crenoloba	.	.	.	.	.	.	.	.	.	.	.	.	.	.	.	.	.	.	.	.	.	.	.	.	.	.	.	.	.
Neobartsia pedicularoides	.	.	.	.	.	.	.	.	.	.	.	.	.	.	.	.	.	.	.	.	.	.	.	.	.	.	.	.	.
Neobartsia peruviana	.	.	.	.	.	.	.	.	.	.	.	.	.	.	.	.	.	.	.	.	.	.	.	.	.	.	.	.	.
Nertera granadensis	.	.	.	.	.	.	.	.	.	.	.	.	.	.	.	.	.	.	.	.	.	.	.	.	.	.	.	.	.
Nicoraepoa andina	.	.	.	.	.	.	.	.	.	.	.	.	.	.	.	.	.	.	.	.	.	.	.	.	.	.	.	.	.
Nicoraepoa pugionifolia	.	.	.	.	.	.	.	.	.	.	.	.	.	.	.	.	.	.	.	.	.	.	.	.	.	.	.	.	.
Nicoraepoa subenervis	.	.	.	.	.	.	.	.	.	.	.	.	.	.	.	.	.	.	.	.	.	.	.	.	.	.	.	.	.

Bog	204	205	206	207	208	209	210	211	212	213	214	215	216	217	218	219	220	221	222	223	224	225	226	227	228	229	230	231	232
Nitrophila australis	.	.	.	.	.	.	.	.	.	.	.	.	.	.	.	.	.	.	.	.	.	.	.	.	.	.	.	.	.
Nothofagus antarctica	.	.	.	.	.	.	.	.	.	1	.	.	.	.	.	.	.	.	1	1	1	.	.	.	.	.	.	.	.
Nototriche rugosa	.	.	.	.	.	.	.	.	.	.	.	.	.	.	.	.	.	.	.	.	.	.	.	.	.	.	.	.	.
Ochetophila nana	1	1	1	1	1	.	.	1	.	.	.	.	1	.	.	1	.	.	1	1	.	1	1	1	1	.	.	.	.
Olsynium junceum	.	.	.	.	.	.	.	.	.	.	.	.	.	.	.	.	.	.	.	.	.	.	.	.	.	.	.	.	.
Oreobolus obtusangulus	.	.	.	.	.	.	.	.	1	.	.	.	.	.	.	.	.	.	.	1	.	.	.	1	.	.	.	.	.
Oritrophium limnophilum	.	.	.	.	.	.	.	.	.	.	.	.	.	.	.	.	.	.	.	.	.	.	.	.	.	.	.	.	.
Osmorhiza glabrata	.	.	.	.	.	.	.	.	.	.	.	.	.	.	.	.	.	.	.	.	.	.	.	.	.	.	.	.	.
Ourisia alpina	.	.	.	.	.	.	.	.	.	.	.	.	.	.	.	.	.	.	.	.	.	.	.	.	.	.	.	.	.
Ourisia muscosa	.	.	.	.	.	.	.	.	.	.	.	.	.	.	.	.	.	.	.	.	.	.	.	.	.	.	.	1	1
Ourisia ruelloides	.	.	.	.	.	.	.	.	.	.	.	.	.	.	.	.	.	.	.	.	.	1	.	.	.	.	.	.	.
Oxychloe andina	.	.	.	.	.	.	.	.	.	.	.	.	.	.	.	.	.	.	.	.	.	.	.	.	.	1	.	1	1
Oxychloe bisexualis	.	.	.	.	.	.	.	.	.	.	.	.	.	.	.	.	.	.	.	.	.	.	.	.	.	.	.	.	.
Oxychloe castellanosii	.	.	.	.	.	.	.	.	.	.	.	.	.	.	.	.	.	.	.	.	.	.	.	.	.	.	.	.	.
Oxychloe haumaniana	.	.	.	.	.	.	.	.	.	.	.	.	.	.	.	.	.	.	.	.	.	.	.	.	.	.	.	.	.
Oxychloe mendocina	.	.	.	.	.	.	.	.	.	.	.	.	1	1	.	1	.	.	.	.	.	.	.	.	.	.	.	.	.
Patosia clandestina	1	1	1	1	1	1	1	1	1	.	1	1	1	.	1	1	1	1	1	1	1	1	1	1	1	.	1	.	.
Perezia capito	.	.	.	.	.	.	.	.	.	.	.	.	.	.	.	.	.	.	.	.	.	.	.	.	.	.	.	.	.
Perezia delicata	.	.	.	.	.	.	.	.	.	.	.	.	.	.	.	.	.	.	.	.	.	.	.	.	.	.	.	.	.
Perezia fonkii	.	.	.	.	.	.	.	.	.	.	.	.	.	.	.	.	.	.	.	.	.	.	.	.	.	.	.	.	.
Perezia pedicularidifolia	.	.	.	.	.	.	.	.	.	.	.	.	.	.	.	.	.	.	.	.	.	.	.	.	.	.	.	.	.
Perezia pinnatifida	.	.	.	.	.	.	.	.	.	.	.	.	.	.	.	.	.	.	.	.	.	.	.	.	.	.	.	.	.
Petroravenia friesii	.	.	.	.	.	.	.	.	.	.	.	.	.	.	.	.	.	.	.	.	.	.	.	.	.	1	.	1	1
Petroravenia werdermannii	.	.	.	.	.	.	.	.	.	.	.	.	.	.	.	.	.	.	.	.	.	.	.	.	.	.	.	.	.
Phleum alpinum	.	1	1	1	.	1	.	1	.	1	.	.	1	.	.	.	1	.	1	1	1	1	.	.	1	.	.	.	.
Phylloscirpus acaulis	.	.	.	.	.	.	1	.	.	1	1	.	1	.	.	1	.	1	1	1	1	1	1	.	1	.	.	.	.
Phylloscirpus boliviensis	.	.	.	.	.	.	.	.	.	.	.	.	.	.	.	.	.	.	.	.	.	.	.	.	.	1	.	1	.
Phylloscirpus deserticola	.	.	.	.	.	.	.	.	.	.	.	.	.	.	.	.	.	.	.	.	.	.	.	.	.	1	1	1	1
Pinguicula antarctica	.	.	.	.	.	.	.	.	.	.	.	.	.	.	.	.	.	.	.	.	.	.	.	.	.	.	.	.	.
Plantago barbata	1	.	1	1	1	1	1	1	1	.	.	.	1	1	.	.	1	1	1	1	1	1	1	1	1	.	.	.	.
Plantago rigida	.	.	.	.	.	.	.	.	.	.	.	.	.	.	.	.	.	.	.	.	.	.	.	.	.	.	.	.	.
Plantago tubulosa	.	.	.	.	.	.	.	.	.	.	.	.	.	.	.	.	.	.	.	.	.	.	.	.	.	1	1	1	1

**Bog**	**204**	**205**	**206**	**207**	**208**	**209**	**210**	**211**	**212**	**213**	**214**	**215**	**216**	**217**	**218**	**219**	**220**	**221**	**222**	**223**	**224**	**225**	**226**	**227**	**228**	**229**	**230**	**231**	**232**
Plantago uniglumis	.	.	.	.	.	.	.	.	.	.	.	.	.	.	.	.	.	.	.	.	.	.	.	.	.	.	.	.	.
Poa alopecurus	.	.	.	.	.	.	.	.	.	.	.	.	.	.	.	.	.	.	.	.	.	.	.	.	.	.	.	.	.
Poa hachadoensis	.	.	.	.	.	.	.	.	.	.	.	.	.	.	.	.	.	.	.	.	.	.	.	.	.	.	.	.	.
Poa perligulata	.	.	.	.	.	.	.	.	.	.	.	.	.	.	.	.	.	.	.	.	.	.	.	.	.	1	.	1	1
Polypogon interruptus	.	.	.	.	.	.	.	.	.	.	.	.	.	.	.	.	.	.	.	.	.	.	.	.	.	.	.	.	.
Primula magellanica	.	.	.	.	.	.	.	.	.	.	.	.	.	.	.	.	.	.	.	.	.	.	.	.	.	.	.	.	.
Puccinellia frigida	.	.	.	.	.	.	.	.	.	.	.	.	.	.	.	.	.	.	.	.	.	.	.	.	.	.	.	.	.
Quinchamalium chilense	.	.	.	.	.	.	.	.	.	.	.	.	.	.	.	.	.	.	.	.	.	.	.	.	.	.	.	.	.
Ranunculus breviscapus	.	.	.	.	.	.	.	.	.	.	.	.	.	.	.	.	.	.	.	.	.	.	.	.	.	.	.	.	.
Ranunculus fuegianus	.	.	.	.	.	.	.	.	.	.	.	.	.	.	.	.	.	.	.	.	.	.	.	.	.	.	.	.	.
Ranunculus mandonianus	.	.	.	.	.	.	.	.	.	.	.	.	.	.	.	.	.	.	.	.	.	.	.	.	.	.	.	.	.
Ranunculus peduncularis	1	1	1	1	1	1	.	1	.	1	.	.	1	.	.	.	.	.	1	1	1	.	.	1	1	.	.	.	.
Ranunculus trichophyllus	.	.	.	.	.	.	.	.	.	.	.	.	.	.	.	.	.	.	.	.	.	.	.	.	.	1	.	1	.
Halerpestes uniflora	.	.	.	.	1	.	.	.	.	.	.	1	.	1	.	.	.	.	.	.	.	.	.	.	.	.	.	.	.
Rubus geoides	.	.	.	.	.	.	.	.	.	.	.	.	.	.	.	.	.	.	.	.	.	.	.	.	.	.	.	.	.
Rumex magellanicus	.	.	.	.	.	.	.	.	.	.	.	.	.	.	.	.	.	.	.	.	.	.	.	.	.	.	.	.	.
Rytidosperma lechleri	.	.	.	.	.	.	.	.	.	.	.	.	.	.	.	.	.	.	.	.	.	.	.	.	.	.	.	.	.
Sarcocornia pulvinata	.	.	.	.	.	.	.	.	.	.	.	.	.	.	.	.	.	.	.	.	.	.	.	.	.	.	.	.	.
Schoenoplectus pungens	.	.	.	.	.	.	.	.	.	.	.	.	.	.	.	.	.	.	.	.	.	.	.	.	.	.	.	.	.
Schoenus andinus	.	.	.	.	.	.	.	.	1	1	.	.	.	.	.	.	.	1	1	1	1	.	1	1	1	.	.	.	.
Senecio breviscapus	.	.	.	.	1	1	.	.	1	.	.	.	.	.	1	.	1	1	.	1	.	.	.	1	.	.	.	.	.
Senecio diemii	.	.	.	.	.	.	.	.	.	.	.	.	.	.	.	.	.	.	.	.	.	.	.	.	.	.	.	.	.
Senecio fistulosus	.	.	.	.	.	.	.	1	.	1	.	.	.	.	.	.	.	.	.	1	1	.	.	.	.	.	.	.	.
Senecio parodii	.	.	.	.	.	.	.	.	.	.	.	.	.	.	.	.	.	.	.	.	.	.	.	.	.	.	.	.	.
Senecio peteroanus	.	.	.	.	.	.	.	.	.	.	.	.	.	.	.	.	.	.	.	.	.	.	.	.	.	.	.	.	.
Senecio serratifolius	.	.	.	.	.	.	.	.	.	.	.	.	.	.	.	.	.	.	.	.	.	.	.	.	.	.	.	.	1
Senecio trifurcatus	.	.	.	.	.	.	.	.	.	.	.	.	.	.	.	.	.	.	.	.	.	.	.	.	.	.	.	.	.
Sisyrinchium chilense	.	.	.	.	.	.	.	.	.	.	.	.	.	.	.	.	.	.	.	.	.	.	.	.	.	.	.	.	.
Sisyrinchium patagonicum	.	.	.	.	.	.	.	.	.	.	.	.	.	.	.	.	.	.	.	.	.	.	.	.	.	.	.	.	.
Sisyrinchium pearcei	.	.	.	.	.	.	.	.	.	.	.	.	.	.	.	.	.	.	.	.	.	.	.	.	.	.	.	.	.
Stellaria debilis	.	.	.	.	1	1	.	.	.	.	1	1	1	.	.	.	.	.	1	.	.	.	.	.	.	.	.	.	.
Stuckenia filiformis	.	.	.	.	.	.	.	.	.	.	.	.	.	.	.	.	.	.	.	.	.	.	.	.	.	.	.	.	.

Bog	204	205	206	207	208	209	210	211	212	213	214	215	216	217	218	219	220	221	222	223	224	225	226	227	228	229	230	231	232
Stuckenia striata	.	.	.	.	.	.	.	.	.	.	.	.	.	.	.	.	.	.	.	.	.	.	.	.	.	.	.	.	.
Symphyotrichum peteroanum	.	.	.	.	.	.	.	.	.	.	.	.	.	.	.	.	.	.	.	.	.	.	.	.	.	.	.	.	.
Symphyotrichum vahlii	1	1	.	.	1	1	.	1	1	1	.	.	.	.	.	1	.	1	1	1	1	1	1	1	1	.	.	.	.
Tetroncium magellanicum	.	.	.	.	.	.	.	.	.	.	.	.	.	.	.	.	.	.	.	.	.	.	.	.	.	.	.	.	.
Tribeles australis	.	.	.	.	.	.	.	.	.	.	.	.	.	.	.	.	.	.	.	.	.	.	.	.	.	.	.	.	.
Trifolium amabile	.	.	.	.	.	.	.	.	.	.	.	.	.	.	.	.	.	.	.	.	.	.	.	.	.	.	.	.	.
Trifolium polymorphum	.	.	.	.	.	.	.	.	.	.	.	.	.	.	.	.	.	.	.	.	.	.	.	.	.	.	.	.	.
Triglochin concinna	.	.	.	.	.	.	.	.	.	.	.	.	.	.	.	.	.	.	.	.	.	.	.	.	.	.	.	.	.
Triglochin palustris	.	.	.	.	.	.	.	1	.	.	.	.	.	.	.	.	.	.	.	.	.	.	.	.	.	.	.	.	.
Triglochin striata	.	.	.	.	.	.	.	.	.	.	.	.	.	.	.	.	.	.	.	.	.	.	.	.	.	.	.	.	.
Trisetum caudulatum	.	.	.	.	.	.	.	.	.	.	.	.	.	.	.	.	.	.	.	.	.	.	.	.	.	.	.	.	.
Trisetum preslei	.	.	.	.	.	.	.	.	.	.	.	.	.	.	.	.	.	.	.	.	.	.	.	.	.	.	.	.	.
Koeleria spicata	.	.	.	.	.	.	.	.	.	.	.	.	.	.	.	.	.	.	.	.	.	.	.	.	.	.	.	.	.
Utricularia gibba	.	.	.	.	.	.	.	.	.	.	.	.	.	.	.	.	.	.	.	.	.	.	.	.	.	.	.	.	.
Vahlodea atropurpurea	.	.	.	.	.	.	.	.	.	.	.	.	.	.	.	.	.	.	.	.	.	.	.	.	.	.	.	.	.
Valeriana fonckii	.	.	.	.	.	.	.	.	.	.	.	.	.	.	.	.	.	.	.	.	.	.	.	.	.	.	.	.	.
Valeriana macrorhiza	.	1	1	1	.	.	.	1	1	.	.	.	.	1	.	.	.	.	1	1	.	.	1	.	1	.	.	.	.
Viola pygmaea	.	.	.	.	.	.	.	.	.	.	.	.	.	.	.	.	.	.	.	.	.	.	.	.	.	.	.	.	.
Werneria apiculata	.	.	.	.	.	.	.	.	.	.	.	.	.	.	.	.	.	.	.	.	.	.	.	.	.	.	.	.	.
Werneria pinnatifida	.	.	.	.	.	.	.	.	.	.	.	.	.	.	.	.	.	.	.	.	.	.	.	.	.	1	.	1	1
Werneria pygmaea	.	.	.	.	.	.	1	.	.	.	.	.	.	.	.	.	.	1	.	.	.	.	.	.	.	1	1	1	1
Werneria solivifolia	.	.	.	.	.	.	.	.	.	.	.	.	.	.	.	.	.	.	.	.	.	.	.	.	.	1	1	1	1
Werneria spathulata	.	.	.	.	.	.	.	.	.	.	.	.	.	.	.	.	.	.	.	.	.	.	.	.	.	1	1	.	1
Xenophyllum incisum	.	.	.	.	.	.	.	.	.	.	.	.	.	.	.	.	.	.	.	.	.	.	.	.	.	.	.	.	.
Zameioscirpus atacamensis	.	.	.	.	.	.	.	.	.	.	.	.	.	.	.	.	.	.	.	.	.	.	.	.	.	.	.	.	.
Zameioscirpus gaimardiodes	.	.	.	.	.	.	.	.	.	.	.	.	.	.	.	.	.	.	.	.	.	.	.	.	.	.	.	.	.
Zameioscirpus muticus	1	1	1	1	1	1	.	1	.	.	.	.	.	.	.	1	1	.	.	1	.	.	.	1	1	1	.	1	1

Bog	233	234	235	236	237	238	239	240	241	242	243	244	245	246	247	248	249	250	251	252	253	254	255	256	257	258	259	260	261
**Operational zone**	**N**	**N**	**N**	**N**	**N**	**N**	**N**	**N**	**N**	**N**	**N**	**N**	**N**	**N**	**N**	**N**	**N**	**N**	**N**	**N**	**N**	**N**	**N**	**S**	**N**	**N**	**N**	**N**	**N**
**Cluster**	**1**	**1**	**1**	**1**	**1**	**1**	**1**	**1**	**1**	**1**	**1**	**1**	**1**	**1**	**1**	**1**	**1**	**1**	**1**	**1**	**1**	**1**	**1**	**9**	**1**	**1**	**1**	**1**	**1**
**Bioregion**	**N**	**N**	**N**	**N**	**N**	**N**	**N**	**N**	**N**	**N**	**N**	**N**	**N**	**N**	**N**	**N**	**N**	**N**	**N**	**N**	**N**	**N**	**N**	**S**	**N**	**N**	**N**	**N**	**N**
**Longitude**	**-69.07**	**-68.88**	**-69**	**-69.02**	**-68.97**	**-68.96**	**-68.97**	**-69**	**-66.61**	**-68.98**	**-68.98**	**-68.97**	**-68.91**	**-68.64**	**-69.01**	**-69.03**	**-68.99**	**-66.62**	**-67.12**	**-67.13**	**-66.78**	**-67.13**	**-66.73**	**-72.15**	**-71.63**	**-71.63**	**-71.63**	**-71.58**	**-71.58**
**Latitude**	**-18.07**	**-18.18**	**-18.12**	**-18.1**	**-18.34**	**-18.34**	**-18.36**	**-18.54**	**-18.87**	**-18.34**	**-18.34**	**-18.53**	**-18.41**	**-18.74**	**-18.11**	**-18.17**	**-18.11**	**-18.87**	**-17.47**	**-17.45**	**-18.28**	**-17.45**	**-18.34**	**-40.75**	**-15.71**	**-15.71**	**-15.71**	**-15.55**	**-15.55**
Acaena antarctica	.	.	.	.	.	.	.	.	.	.	.	.	.	.	.	.	.	.	.	.	.	.	.	1	.	.	.	.	.
Acaena macrocephala	.	.	.	.	.	.	.	.	.	.	.	.	.	.	.	.	.	.	.	.	.	.	.	.	.	.	.	.	.
Acaena magellanica	.	.	.	.	.	.	.	.	.	.	.	.	.	.	.	.	.	.	.	.	.	.	.	.	.	.	.	.	.
Acaena ovalifolia	.	.	.	.	.	.	.	.	.	.	.	.	.	.	.	.	.	.	.	.	.	.	.	.	.	.	.	.	.
Acaena pinnatifida	.	.	.	.	.	.	.	.	.	.	.	.	.	.	.	.	.	.	.	.	.	.	.	.	.	.	.	.	.
Adesmia retusa	.	.	.	.	.	.	.	.	.	.	.	.	.	.	.	.	.	.	.	.	.	.	.	1	.	.	.	.	.
Agrostis breviculmis	.	.	.	.	.	.	.	.	.	.	.	.	.	.	.	.	.	.	.	.	.	.	.	.	.	.	.	.	.
Agrostis imberbis	.	.	.	.	.	.	.	.	.	.	.	.	.	.	.	.	.	.	.	.	.	.	.	.	.	.	.	.	.
Agrostis meyenii	.	.	.	.	.	.	.	.	.	.	.	.	.	.	.	.	.	.	.	.	.	.	.	1	.	.	.	.	.
Agrostis perennans	.	.	.	.	.	.	.	.	.	.	.	.	.	.	.	.	.	.	.	.	.	.	.	.	.	.	.	.	.
Alchemilla pinnata	.	.	.	.	.	.	.	.	.	.	.	.	.	.	.	.	.	.	.	.	.	.	.	.	.	.	.	.	.
Alopecurus magellanicus	.	.	.	.	.	.	.	.	.	.	.	.	.	.	.	.	.	.	.	.	.	.	.	.	.	.	.	.	.
Amphiscirpus nevadensis	.	.	.	.	.	.	.	.	.	.	.	.	.	.	.	.	.	.	.	.	.	.	.	.	.	.	.	.	.
Anagallis alternifolia	.	.	.	.	.	.	.	.	.	.	.	.	.	.	.	.	.	.	.	.	.	.	.	.	.	.	.	.	.
Antennaria chilensis	.	.	.	.	.	.	.	.	.	.	.	.	.	.	.	.	.	.	.	.	.	.	.	.	.	.	.	.	.
Anthoxanthum redolens	.	.	.	.	.	.	.	.	.	.	.	.	.	.	.	.	.	.	.	.	.	.	.	.	.	.	.	.	.
Apium panul	.	.	.	.	.	.	.	.	.	.	.	.	.	.	.	.	.	.	.	.	.	.	.	.	.	.	.	.	.
Arenaria rivularis	.	.	.	.	.	.	.	.	.	.	.	.	.	.	.	.	.	.	.	.	.	.	.	.	.	.	.	.	.
Arenaria serpens	.	.	.	.	.	.	.	.	.	.	.	.	.	.	.	.	.	.	.	.	.	.	.	.	.	.	.	.	.
Arjona pusilla	.	.	.	.	.	.	.	.	.	.	.	.	.	.	.	.	.	.	.	1	.	.	.	.	.	.	.	.	.
Astragalus bustillosii	.	.	.	.	.	.	.	.	.	.	.	.	.	.	.	.	.	.	.	.	.	.	.	.	.	.	.	.	.
Astragalus micranthellus	.	.	.	.	.	.	.	.	.	.	.	.	.	.	.	.	.	.	.	.	.	.	.	.	.	.	.	.	.
Azolla filiculoides	.	.	.	1	.	1	1	.	.	.	.	.	1	.	1	.	.	.	.	.	.	.	.	.	.	.	.	1	1
Azorella boelckei	.	.	.	.	.	.	.	.	.	.	.	.	.	.	.	.	.	.	.	.	.	.	.	.	.	.	.	.	.

Bog	233	234	235	236	237	238	239	240	241	242	243	244	245	246	247	248	249	250	251	252	253	254	255	256	257	258	259	260	261
Azorella burkartii	.	.	.	.	.	.	.	.	.	.	.	.	.	.	.	.	.	.	.	.	.	.	.	.	.	.	.	.	.
Azorella cryptantha	.	.	.	.	.	.	.	.	.	.	.	.	.	.	.	.	.	.	.	.	.	.	.	.	.	.	.	.	.
Azorella lycopodioides	.	.	.	.	.	.	.	.	.	.	.	.	.	.	.	.	.	.	.	.	.	.	.	1	.	.	.	.	.
Azorella trifoliolata	.	.	.	.	.	.	.	.	.	.	.	.	.	.	.	.	.	.	.	.	.	.	.	.	.	.	.	.	.
Baccharis acaulis	.	.	.	.	.	.	.	.	.	.	.	.	.	.	.	.	.	.	.	.	.	.	.	.	.	.	.	1	.
Baccharis caespitosa	.	.	.	.	.	.	.	.	.	.	.	.	.	.	.	.	.	.	.	1	.	.	.	.	.	.	.	.	.
Baccharis magellanica	.	.	.	.	.	.	.	.	.	.	.	.	.	.	.	.	.	.	.	.	.	.	.	1	.	.	.	.	.
Belloa chilensis	.	.	.	.	.	.	.	.	.	.	.	.	.	.	.	.	.	.	.	.	.	.	.	1	.	.	.	.	.
Bromus catharticus	.	.	.	.	.	.	.	.	.	.	.	.	.	.	.	.	.	.	.	.	.	.	.	.	.	.	.	.	.
Calandrinia acaulis	.	.	.	.	.	.	.	.	.	.	.	.	.	.	.	.	.	.	.	.	.	.	.	.	.	.	.	.	.
Calandrinia compacta	1	.	1	.	.	1	.	1	.	.	.	1	1	1	.	.	.	.	1	.	1	.	.	.	.	.	1	.	.
Calceolaria biflora	.	.	.	.	.	.	.	.	.	.	.	.	.	.	.	.	.	.	.	.	.	.	.	.	.	.	.	.	.
Calceolaria cana	.	.	.	.	.	.	.	.	.	.	.	.	.	.	.	.	.	.	.	.	.	.	.	.	.	.	.	.	.
Calceolaria corymbosa	.	.	.	.	.	.	.	.	.	.	.	.	.	.	.	.	.	.	.	.	.	.	.	.	.	.	.	.	.
Calceolaria filicaulis	.	.	.	.	.	.	.	.	.	.	.	.	.	.	.	.	.	.	.	.	.	.	.	.	.	.	.	.	.
Callitriche lechleri	.	.	.	.	.	.	.	.	.	.	.	.	.	.	.	.	.	.	.	.	.	.	.	.	.	.	.	.	.
Caltha appendiculata	.	.	.	.	.	.	.	.	.	.	.	.	.	.	.	.	.	.	.	.	.	.	.	.	.	.	.	.	.
Caltha sagittata	.	.	.	.	.	.	.	.	.	.	.	.	.	.	.	.	.	.	1	.	1	.	.	.	.	.	.	.	.
Cardamine cordata	.	.	.	.	.	.	.	.	.	.	.	.	.	.	.	.	.	.	.	.	.	.	.	.	.	.	.	.	.
Cardamine glacialis	.	.	.	.	.	.	.	.	.	.	.	.	.	.	.	.	.	.	.	.	.	.	.	.	.	.	.	.	.
Cardamine tenuirostris	.	.	.	.	.	.	.	.	.	.	.	.	.	.	.	.	.	.	.	.	.	.	.	.	.	.	.	.	.
Cardamine volckmannii	.	.	.	.	.	.	.	.	.	.	.	.	.	.	.	.	.	.	.	.	.	.	.	.	.	.	.	.	.
Carex acaulis	.	.	.	.	.	.	.	.	.	.	.	.	.	.	.	.	.	.	.	.	.	.	.	.	.	.	.	.	.
Carex atropicta	.	.	.	.	.	.	.	.	.	.	.	.	.	.	.	.	.	.	.	.	.	.	.	.	.	.	.	.	.
Carex banksii	.	.	.	.	.	.	.	.	.	.	.	.	.	.	.	.	.	.	.	.	.	.	.	.	.	.	.	.	.
Carex caduca	.	.	.	.	.	.	.	.	.	.	.	.	.	.	.	.	.	.	.	.	.	.	.	1	.	.	.	.	.
Carex decidua	.	.	.	.	.	.	.	.	.	.	.	.	.	.	.	.	.	.	.	.	.	.	.	.	.	.	.	.	.
Carex fuscula	.	.	.	.	.	.	.	.	.	.	.	.	.	.	.	.	.	.	.	.	.	.	.	.	.	.	.	.	.
Carex gayana	.	.	.	.	.	.	.	.	1	.	.	.	.	.	.	.	.	.	.	.	.	.	1	.	.	.	.	.	.
Carex hypoleucos	.	.	.	.	.	.	.	.	.	.	.	.	.	.	.	.	.	.	.	.	.	.	.	.	.	.	.	.	.
Carex macloviana	.	.	.	.	.	.	.	.	.	.	.	.	.	.	.	.	.	.	.	.	.	.	.	.	.	.	.	.	.
Carex magellanica	.	.	.	.	.	.	.	.	.	.	.	.	.	.	.	.	.	.	.	.	.	.	.	.	.	.	.	.	.
Carex malmei	.	.	.	.	.	.	.	.	.	.	.	.	.	.	.	.	.	.	.	.	.	.	.	.	.	.	.	.	.
Carex maritima	.	.	1	.	.	.	.	1	.	1	.	.	1	1	.	.	1	.	1	1	1	1	.	.	.	.	.	.	.
Carex microglochin	.	.	.	.	.	.	.	.	.	.	.	.	.	.	.	.	.	.	.	.	.	.	.	.	.	.	.	.	.
Carex pleioneura	.	.	.	.	.	.	.	.	.	.	.	.	.	.	.	.	.	.	.	.	.	.	.	.	.	.	.	.	.
Carex ruthsatzae	.	.	1	.	.	.	.	.	.	.	.	.	.	.	.	.	.	.	.	.	.	.	.	.	.	.	.	.	.
Carex vallis-pulchrae	.	.	1	.	.	1	1	.	.	.	.	.	.	.	.	.	.	.	.	.	.	.	.	.	.	.	.	1	.

Bog	233	234	235	236	237	238	239	240	241	242	243	244	245	246	247	248	249	250	251	252	253	254	255	256	257	258	259	260	261
Carpha schoenoides	.	.	.	.	.	.	.	.	.	.	.	.	.	.	.	.	.	.	.	.	.	.	.	.	.	.	.	.	.
Castilleja pumila	.	.	1	.	.	1	.	.	1	.	.	.	.	.	.	.	.	1	1	1	.	1	1	.	1	.	1	1	1
Catabrosa werdermannii	.	.	.	.	.	.	.	.	.	.	.	.	.	.	.	.	.	.	.	.	.	.	.	.	.	.	.	.	.
Cerastium humifusum	.	.	.	.	.	.	.	.	.	.	.	.	.	.	.	.	.	.	.	.	.	.	.	.	.	.	.	.	.
Cerastium montioides	.	.	.	.	.	.	.	.	.	.	.	.	.	.	.	.	.	.	.	.	.	.	.	.	.	.	.	.	.
Chiliotrichum diffusum	.	.	.	.	.	.	.	.	.	.	.	.	.	.	.	.	.	.	.	.	.	.	.	.	.	.	.	.	.
Chusquea culeou	.	.	.	.	.	.	.	.	.	.	.	.	.	.	.	.	.	.	.	.	.	.	.	.	.	.	.	.	.
Colobanthus quitensis	.	.	1	.	1	1	1	1	.	.	.	1	1	.	.	.	.	.	1	.	1	1	.	.	.	.	.	.	.
Cortaderia egmontiana	.	.	.	.	.	.	.	.	.	.	.	.	.	.	.	.	.	.	.	.	.	.	.	1	.	.	.	.	.
Cotula mexicana	.	.	.	1	1	1	1	1	1	.	.	1	1	1	1	.	.	1	1	1	1	1	1	.	1	1	.	1	1
Crassula peduncularis	.	.	.	.	.	.	.	.	.	.	.	.	.	.	.	.	.	.	.	.	.	.	.	.	.	.	.	.	.
Cuatrecasasiella argentina	1	.	1	1	1	1	1	1	.	.	.	1	1	1	1	.	.	.	.	1	.	.	.	.	.	1	.	1	1
Deschampsia antarctica	.	.	.	.	.	.	.	.	.	.	.	.	.	.	.	.	.	.	.	.	.	.	.	.	.	.	.	.	.
Deschampsia caespitosa	.	.	.	.	.	.	.	.	.	.	.	.	.	.	.	.	.	.	.	.	.	.	.	.	.	.	.	.	.
Deschampsia patula	.	.	.	.	.	.	.	.	.	.	.	.	.	.	.	.	.	.	.	.	.	.	.	.	.	.	.	.	.
Cinnagrostis brevifolia	.	.	.	.	.	.	.	.	.	.	.	.	.	.	.	.	.	.	.	.	.	.	.	.	.	.	.	.	.
Deschampsia chrysantha	.	1	.	.	.	.	.	.	.	.	.	.	.	.	.	.	.	.	.	.	.	.	.	.	.	.	1	.	.
Cinnagrostis chrysophylla	.	.	.	.	.	.	.	.	.	.	.	.	.	.	.	.	.	.	.	.	.	.	.	.	.	.	.	.	.
Deschampsia chrysostachya	.	.	.	.	.	.	.	.	.	.	.	.	.	.	.	.	.	.	.	.	.	.	.	.	.	.	.	.	.
Deschampsia eminens	1	.	1	.	.	.	.	.	.	.	.	.	.	.	.	.	.	.	.	.	.	.	.	.	.	.	.	.	.
Deschampsia hackelii	.	.	.	.	.	.	.	.	.	.	.	.	.	.	.	.	.	.	.	.	.	.	.	.	.	.	.	.	.
Cinnagrostis minima	.	.	.	.	.	.	.	.	.	.	.	.	.	.	.	.	.	.	.	.	.	.	.	.	.	.	.	.	.
Deschampsia ovata	.	.	.	.	.	.	.	.	.	.	.	.	.	.	.	.	.	.	.	.	.	.	.	.	.	.	.	.	.
Cinnagrostis rigescens	1	.	1	1	1	1	1	1	1	.	.	1	1	1	.	1	.	1	1	1	1	1	1	.	1	1	.	1	1
Cinnagrostis spicigera	1	1	.	.	.	.	.	.	.	.	.	.	.	.	.	.	.	.	.	.	.	.	.	.	.	.	1	.	1
Cinnagrostis velutina	.	.	.	.	.	.	.	.	.	.	.	.	.	.	.	.	.	.	.	.	.	.	.	.	.	.	.	.	.
Cinnagrostis vicunarum	.	.	.	.	.	.	.	.	.	.	.	.	.	.	.	.	.	1	.	.	.	.	.	.	.	.	.	.	.
Distichia filamentosa	.	.	.	.	.	.	.	.	.	.	.	.	.	.	.	.	.	.	.	.	.	.	.	.	.	.	.	.	.
Distichia muscoides	1	1	1	1	1	1	1	1	.	1	1	1	1	1	1	.	.	.	1	1	1	1	1	.	1	1	1	1	1
Distichlis humilis	.	.	.	.	.	.	.	.	.	.	.	.	.	1	.	.	.	.	.	.	.	.	.	.	.	.	.	.	.
Distichlis scoparia	.	.	.	.	.	.	.	.	.	.	.	.	.	.	.	.	.	.	.	.	.	.	.	.	.	.	.	.	.
Distichlis spicata	.	.	.	.	.	.	.	.	.	.	.	.	.	.	.	.	.	.	.	.	.	.	.	.	.	.	.	.	.

Bog	233	234	235	236	237	238	239	240	241	242	243	244	245	246	247	248	249	250	251	252	253	254	255	256	257	258	259	260	261
Draba pusilla	.	.	.	.	.	.	.	.	.	.	.	.	.	.	.	.	.	.	.	.	.	.	.	.	.	.	.	.	.
Eleocharis melanomphala	.	.	.	.	.	.	.	.	.	.	.	.	.	.	.	.	.	.	.	.	.	.	.	.	.	.	.	.	.
Eleocharis pseudoalbibracteata	.	.	.	.	.	.	.	.	.	.	.	.	.	.	.	.	.	.	.	.	.	.	.	.	.	.	.	.	.
Elodea potamogeton	.	.	.	.	.	.	.	.	.	.	.	.	1	.	.	.	.	.	.	.	.	.	.	.	1	.	.	.	.
Empetrum rubrum	.	.	.	.	.	.	.	.	.	.	.	.	.	.	.	.	.	.	.	.	.	.	.	1	.	.	.	.	.
Epilobium australe	.	.	.	.	.	.	.	.	.	.	.	.	.	.	.	.	.	.	.	.	.	.	.	.	.	.	.	.	.
Epilobium barbeyanum	.	.	.	.	.	.	.	.	.	.	.	.	.	.	.	.	.	.	.	.	.	.	.	.	.	.	.	.	.
Epilobium ciliatum	.	.	.	.	.	.	.	.	.	.	.	.	.	.	.	.	.	.	.	.	.	.	.	.	.	.	.	.	.
Epilobium denticulatum	.	.	.	.	.	.	.	.	1	.	.	.	.	.	.	.	.	.	.	.	.	.	.	.	.	.	.	.	.
Epilobium fragile	.	.	.	.	.	.	.	.	.	.	.	.	.	.	.	.	.	.	.	.	.	.	.	.	.	.	.	.	.
Epilobium glaucum	.	.	.	.	.	.	.	.	.	.	.	.	.	.	.	.	.	.	.	.	.	.	.	.	.	.	.	.	.
Epilobium nivale	.	.	.	.	.	.	.	.	.	.	.	.	.	.	.	.	.	.	.	.	.	.	.	.	.	.	.	.	.
Erigeron andicola	.	.	.	.	.	.	.	.	.	.	.	.	.	.	.	.	.	.	.	.	.	.	.	.	.	.	.	.	.
Erigeron leptopetalus	.	.	.	.	.	.	.	.	.	.	.	.	.	.	.	.	.	.	.	.	.	.	.	.	.	.	.	.	.
Erigeron myosotis	.	.	.	.	.	.	.	.	.	.	.	.	.	.	.	.	.	.	.	.	.	.	.	1	.	.	.	.	.
Erigeron patagonicus	.	.	.	.	.	.	.	.	.	.	.	.	.	.	.	.	.	.	.	.	.	.	.	.	.	.	.	.	.
Erythranthe cuprea	.	.	.	.	.	.	.	.	.	.	.	.	.	.	.	.	.	.	.	.	.	.	.	.	.	.	.	.	.
Erythranthe depressa	1	.	.	.	.	.	.	.	.	.	.	.	.	.	.	.	.	.	.	.	.	.	.	.	.	.	.	.	.
Erythranthe glabrata	.	.	.	.	.	1	.	.	1	.	.	1	1	1	.	.	.	1	.	.	.	.	.	.	.	.	.	1	.
Erythranthe lutea	.	.	.	.	.	.	.	.	.	.	.	.	.	.	.	.	.	.	.	.	.	.	.	.	.	.	.	.	.
Escallonia virgata	.	.	.	.	.	.	.	.	.	.	.	.	.	.	.	.	.	.	.	.	.	.	.	.	.	.	.	.	.
Euphrasia antarctica	.	.	.	.	.	.	.	.	.	.	.	.	.	.	.	.	.	.	.	.	.	.	.	.	.	.	.	.	.
Euphrasia chrysantha	.	.	.	.	.	.	.	.	.	.	.	.	.	.	.	.	.	.	.	.	.	.	.	.	.	.	.	.	.
Euphrasia subexserta	.	.	.	.	.	.	.	.	.	.	.	.	.	.	.	.	.	.	.	.	.	.	.	1	.	.	.	.	.
Festuca hypsophila	.	.	.	.	.	.	.	.	.	.	.	.	.	.	.	.	.	.	.	.	.	.	.	.	.	.	.	.	.
Festuca kurtziana	.	.	.	.	.	.	.	.	.	.	.	.	.	.	.	.	.	.	.	.	.	.	.	.	.	.	.	.	.
Festuca lilloi	.	.	.	.	.	.	.	.	.	.	.	.	.	.	.	.	.	.	.	.	.	.	.	.	.	.	.	.	.
Festuca magellanica	.	.	.	.	.	.	.	.	.	.	.	.	.	.	.	.	.	.	.	.	.	.	.	.	.	.	.	.	.
Festuca nardifolia	.	.	.	.	.	.	.	.	.	.	.	.	.	.	.	.	.	.	.	.	.	.	.	.	.	.	.	.	.
Festuca rigescens	.	.	.	.	.	.	.	.	.	.	.	.	.	.	.	.	.	.	.	.	.	.	.	.	.	.	.	.	.
Festuca werdermannii	.	.	.	.	.	.	.	.	.	.	.	.	.	.	.	.	.	.	.	.	.	.	.	.	.	.	.	.	.
Frankenia triandra	.	.	.	.	.	.	.	.	.	.	.	.	.	.	.	.	.	.	.	.	.	.	.	.	.	.	.	.	.
Gamocarpha graminea	.	.	.	.	.	.	.	.	.	.	.	.	.	.	.	.	.	.	.	.	.	.	.	.	.	.	.	.	.
Gamocarpha ventosa	.	.	.	.	.	.	.	.	.	.	.	.	.	.	.	.	.	.	.	.	.	.	.	.	.	.	.	.	.
Gamochaeta chamissonis	.	.	.	.	.	.	.	.	.	.	.	.	.	.	.	.	.	.	.	.	.	.	.	1	.	.	.	.	.

Bog	233	234	235	236	237	238	239	240	241	242	243	244	245	246	247	248	249	250	251	252	253	254	255	256	257	258	259	260	261
Gamochaeta longipedicellata	.	.	.	.	.	.	.	.	.	.	.	.	.	.	.	.	.	.	.	.	.	.	.	.	.	.	.	.	.
Gamochaeta neuquensis	.	.	.	.	.	.	.	.	.	.	.	.	.	.	.	.	.	.	.	.	.	.	.	.	.	.	.	.	.
Gaultheria antarctica	.	.	.	.	.	.	.	.	.	.	.	.	.	.	.	.	.	.	.	.	.	.	.	1	.	.	.	.	.
Gaultheria caespitosa	.	.	.	.	.	.	.	.	.	.	.	.	.	.	.	.	.	.	.	.	.	.	.	1	.	.	.	.	.
Gaultheria pumila	.	.	.	.	.	.	.	.	.	.	.	.	.	.	.	.	.	.	.	.	.	.	.	1	.	.	.	.	.
Gavilea chica	.	.	.	.	.	.	.	.	.	.	.	.	.	.	.	.	.	.	.	.	.	.	.	.	.	.	.	.	.
Gentiana prostrata	1	1	1	1	1	1	1	1	.	.	1	.	1	1	.	.	.	.	1	1	1	1	1	.	.	.	1	.	1
Gentianella fiebrigii	.	.	.	.	.	.	.	.	.	.	.	.	.	.	.	.	.	.	.	.	.	.	.	.	.	.	.	.	.
Gentianella magellanica	.	.	.	.	.	.	.	.	.	.	.	.	.	.	.	.	.	.	.	.	.	.	.	.	.	.	.	.	.
Gentianella multicaulis	.	.	.	.	.	.	.	.	.	.	.	.	.	.	.	.	.	.	.	.	.	.	.	.	.	.	.	.	.
Gentianella ottonis	.	.	.	.	.	.	.	.	.	.	.	.	.	.	.	.	.	.	.	.	.	.	.	.	.	.	.	.	.
Gentianella primuloides	.	.	.	.	.	.	.	.	.	.	.	.	.	.	.	.	.	.	1	1	.	.	.	.	.	.	.	.	.
Gentianella pseudocrassula	.	.	.	.	.	.	.	.	.	.	.	.	.	.	.	.	.	.	.	.	.	.	.	.	.	.	.	.	.
Geranium sessiliflorum	.	.	.	.	.	.	.	.	.	.	.	.	.	.	.	.	.	.	.	.	.	.	.	.	.	.	.	.	.
Gunnera magellanica	.	.	.	.	.	.	.	.	.	.	.	.	.	.	.	.	.	.	.	.	.	.	.	1	.	.	.	.	.
Halenia caespitosa	.	.	.	.	.	.	.	.	.	.	.	.	.	.	.	.	.	.	.	.	.	.	.	.	.	.	.	.	.
Halerpestes cymbalaria	.	.	.	.	.	.	.	.	.	.	.	.	.	.	.	.	.	.	.	.	.	.	.	.	.	.	.	.	.
Halerpestes exilis	.	.	.	.	.	.	.	.	.	.	.	.	.	.	.	.	.	.	.	.	.	.	.	.	.	.	.	.	.
Hieracium antarcticum	.	.	.	.	.	.	.	.	.	.	.	.	.	.	.	.	.	.	.	.	.	.	.	1	.	.	.	.	.
Hordeum comosum	.	.	.	.	.	.	.	.	.	.	.	.	.	.	.	.	.	.	.	.	.	.	.	.	.	.	.	.	.
Hordeum muticum	.	.	.	.	.	.	.	.	.	.	.	.	.	.	.	.	.	.	.	.	.	.	.	.	.	.	.	.	.
Hypochaeris acaulis	.	.	.	.	.	.	.	.	.	.	.	.	.	.	.	.	.	.	.	.	.	.	.	.	.	.	.	.	.
Hypochaeris chondrilloides	.	.	.	.	.	.	.	.	.	.	.	.	.	.	.	.	.	.	.	.	.	.	.	.	.	.	.	.	.
Hypochaeris meyeniana	.	.	.	.	.	.	.	.	.	.	.	.	.	.	.	.	.	.	.	.	.	.	.	.	.	.	.	.	.
Hypochaeris palustris	.	.	.	.	.	.	.	.	.	.	.	.	.	.	.	.	.	.	.	.	.	.	.	.	.	.	.	.	.
Hypochaeris taraxacoides	.	.	1	.	.	1	1	1	1	.	.	1	1	1	.	.	.	1	1	1	1	1	1	.	1	1	1	.	1
Hypochaeris tenerifolia	.	.	.	.	.	.	.	.	.	.	.	.	.	.	.	.	.	.	.	.	.	.	.	1	.	.	.	.	.
Isolepis nigricans	.	.	.	.	.	.	.	.	.	.	.	.	.	.	.	.	.	.	.	.	.	.	.	.	.	.	.	.	.
Isolepsis inundata	.	.	.	.	.	.	.	.	.	.	.	.	.	.	.	.	.	.	.	.	.	.	.	.	.	.	.	.	.
Juncus balticus	.	.	.	.	.	.	.	.	.	.	.	.	.	.	.	.	.	.	.	.	.	.	.	.	.	.	.	.	.
Juncus stipulatus	1	.	1	1	.	1	1	1	1	.	.	1	1	1	.	.	.	1	1	1	.	1	1	.	1	1	.	1	1
Koeleria kurtzii	.	.	.	.	.	.	.	.	.	.	.	.	.	.	.	.	.	.	.	.	.	.	.	.	.	.	.	.	.

Bog	233	234	235	236	237	238	239	240	241	242	243	244	245	246	247	248	249	250	251	252	253	254	255	256	257	258	259	260	261
Lachemilla diplophylla	1	.	1	1	1	1	1	1	1	.	.	1	1	1	1	.	.	.	1	1	1	1	1	.	1	1	1	1	1
Lachemilla pinnata	.	.	.	1	.	1	1	1	.	.	.	1	.	1	.	.	.	1	1	1	1	1	.	.	.	1	1	.	.
Lagenophora nudicaulis	.	.	.	.	.	.	.	.	.	.	.	.	.	.	.	.	.	.	.	.	.	.	.	.	.	.	.	.	.
Lemna minuta	1	.	.	.	.	.	.	.	.	.	.	1	.	1	.	.	.	.	.	.	.	.	.	.	.	.	.	.	.
Leptinella scariosa	.	.	.	.	.	.	.	.	.	.	.	.	.	.	.	.	.	.	.	.	.	.	.	.	.	.	.	.	.
Leucheria candidissima	.	.	.	.	.	.	.	.	.	.	.	.	.	.	.	.	.	.	.	.	.	.	.	.	.	.	.	.	.
Leucheria nutans	.	.	.	.	.	.	.	.	.	.	.	.	.	.	.	.	.	.	.	.	.	.	.	.	.	.	.	.	.
Lilaea scilloides	.	.	.	.	.	.	.	.	.	.	.	.	.	.	.	.	.	.	.	.	.	.	.	.	.	.	.	.	.
Lilaeopsis macloviana	.	.	1	.	1	1	1	1	.	1	.	1	1	1	1	.	1	.	1	.	.	1	.	.	.	1	1	1	1
Limosella australis	.	.	.	.	.	.	.	.	.	.	.	.	.	.	.	.	.	.	.	.	.	.	.	.	.	.	.	.	.
Lobelia oligophylla	1	1	1	1	1	1	1	1	1	1	1	1	1	1	1	1	1	1	1	1	1	1	1	.	1	1	.	1	1
Luzula brachyphylla	.	.	.	.	.	.	.	.	.	.	.	.	.	.	.	.	.	.	.	.	.	.	.	.	.	.	.	.	.
Luzula chilensis	.	.	.	.	.	.	.	.	.	.	.	.	.	.	.	.	.	.	.	.	.	.	.	.	.	.	.	.	.
Luzula racemosa	.	.	.	.	.	.	.	.	.	.	.	.	.	.	.	.	.	.	.	.	.	.	.	.	.	.	.	.	.
Luzula vulcanica	.	.	.	.	.	.	.	.	.	.	.	.	.	.	.	.	.	.	.	.	.	.	.	.	.	.	.	.	.
Lysipomia pumila	.	.	.	.	.	.	.	.	.	.	.	.	.	.	.	.	.	.	.	.	.	.	.	.	.	.	.	.	.
Marsippospermum philippii	.	.	.	.	.	.	.	.	.	.	.	.	.	.	.	.	.	.	.	.	.	.	.	.	.	.	.	.	.
Marsippospermum reichei	.	.	.	.	.	.	.	.	.	.	.	.	.	.	.	.	.	.	.	.	.	.	.	.	.	.	.	.	.
Montia fontana	.	.	1	.	.	1	.	.	.	.	.	.	.	.	.	.	.	.	.	.	.	.	.	.	.	.	1	.	.
Muhlenbergia asperifolia	.	.	.	.	.	.	.	.	.	.	.	.	.	.	.	.	.	.	.	.	.	.	.	.	.	.	.	.	.
Myriophyllum quitense	.	.	.	.	.	1	.	.	.	.	.	.	1	1	1	.	.	.	.	.	.	.	.	.	.	.	.	.	.
Myrosmodes nervosa	.	.	.	.	.	.	.	.	.	.	.	.	.	.	.	.	.	.	.	.	.	.	.	.	.	.	.	.	.
Myrosmodes paludosa	.	.	.	.	.	.	.	.	.	.	.	.	.	.	.	.	.	.	.	.	.	.	.	.	.	.	.	.	.
Myrteola nummularia	.	.	.	.	.	.	.	.	.	.	.	.	.	.	.	.	.	.	.	.	.	.	.	.	.	.	.	.	.
Nanodea muscosa	.	.	.	.	.	.	.	.	.	.	.	.	.	.	.	.	.	.	.	.	.	.	.	.	.	.	.	.	.
Neobartsia crenoloba	.	.	.	.	.	.	.	.	.	.	.	.	.	.	.	.	.	.	.	.	.	.	.	.	.	.	.	.	.
Neobartsia pedicularoides	.	.	.	.	.	.	.	.	.	.	.	.	.	.	.	.	.	.	.	.	.	.	.	.	.	.	.	.	.
Neobartsia peruviana	.	.	.	.	.	.	.	.	.	.	.	.	.	.	.	.	.	.	.	.	.	.	.	.	.	.	.	.	.
Nertera granadensis	.	.	.	.	.	.	.	.	.	.	.	.	.	.	.	.	.	.	.	.	.	.	.	.	.	.	.	.	.
Nicoraepoa andina	.	.	.	.	.	.	.	.	.	.	.	.	.	.	.	.	.	.	.	.	.	.	.	.	.	.	.	.	.
Nicoraepoa pugionifolia	.	.	.	.	.	.	.	.	.	.	.	.	.	.	.	.	.	.	.	.	.	.	.	.	.	.	.	.	.
Nicoraepoa subenervis	.	.	.	.	.	.	.	.	.	.	.	.	.	.	.	.	.	.	.	.	.	.	.	.	.	.	.	.	.

**Bog**	**233**	**234**	**235**	**236**	**237**	**238**	**239**	**240**	**241**	**242**	**243**	**244**	**245**	**246**	**247**	**248**	**249**	**250**	**251**	**252**	**253**	**254**	**255**	**256**	**257**	**258**	**259**	**260**	**261**
Nitrophila australis	.	.	.	.	.	.	.	.	.	.	.	.	.	.	.	.	.	.	.	.	.	.	.	.	.	.	.	.	.
Nothofagus antarctica	.	.	.	.	.	.	.	.	.	.	.	.	.	.	.	.	.	.	.	.	.	.	.	.	.	.	.	.	.
Nototriche rugosa	.	.	.	.	.	.	.	.	.	.	.	.	.	.	.	.	.	.	.	.	.	.	.	.	.	.	.	.	.
Ochetophila nana	.	.	.	.	.	.	.	.	.	.	.	.	.	.	.	.	.	.	.	.	.	.	.	.	.	.	.	.	.
Olsynium junceum	.	.	.	.	.	.	.	.	.	.	.	.	.	.	.	.	.	.	.	.	.	.	.	.	.	.	.	.	.
Oreobolus obtusangulus	.	.	.	.	.	.	.	.	.	.	.	.	.	.	.	.	.	.	.	.	.	.	.	1	.	.	.	.	.
Oritrophium limnophilum	.	.	.	.	.	.	.	.	.	.	.	.	.	.	.	.	.	.	.	.	.	.	.	.	.	.	.	.	.
Osmorhiza glabrata	.	.	.	.	.	.	.	.	.	.	.	.	.	.	.	.	.	.	.	.	.	.	.	.	.	.	.	.	.
Ourisia alpina	.	.	.	.	.	.	.	.	.	.	.	.	.	.	.	.	.	.	.	.	.	.	.	.	.	.	.	.	.
Ourisia muscosa	1	.	.	.	.	.	.	.	.	.	1	.	.	.	.	.	.	.	.	.	1	.	.	.	1	1	1	1	1
Ourisia ruelloides	.	.	.	.	.	.	.	.	.	.	.	.	.	.	.	.	.	.	.	.	.	.	.	.	.	.	.	.	.
Oxychloe andina	1	1	1	1	1	1	1	1	.	1	1	1	1	1	1	1	1	.	.	.	.	.	.	.	.	1	1	1	1
Oxychloe bisexualis	.	.	.	.	.	.	.	.	.	.	.	.	.	.	.	.	.	.	.	.	.	.	.	.	.	.	.	.	.
Oxychloe castellanosii	.	.	.	.	.	.	.	.	.	.	.	.	.	.	.	.	.	.	.	.	.	.	.	.	.	.	.	.	.
Oxychloe haumaniana	.	.	.	.	.	.	.	.	.	.	.	.	.	.	.	.	.	.	.	.	.	.	.	.	.	.	.	.	.
Oxychloe mendocina	.	.	.	.	.	.	.	.	.	.	.	.	.	.	.	.	.	.	.	.	.	.	.	.	.	.	.	.	.
Patosia clandestina	.	.	.	.	.	.	.	.	1	.	.	.	.	.	.	.	.	1	1	.	.	.	.	.	1	1	.	.	.
Perezia capito	.	.	.	.	.	.	.	.	.	.	.	.	.	.	.	.	.	.	.	.	.	.	.	.	.	.	.	.	.
Perezia delicata	.	.	.	.	.	.	.	.	.	.	.	.	.	.	.	.	.	.	.	.	.	.	.	.	.	.	.	.	.
Perezia fonkii	.	.	.	.	.	.	.	.	.	.	.	.	.	.	.	.	.	.	.	.	.	.	.	.	.	.	.	.	.
Perezia pedicularidifolia	.	.	.	.	.	.	.	.	.	.	.	.	.	.	.	.	.	.	.	.	.	.	.	1	.	.	.	.	.
Perezia pinnatifida	.	.	.	.	.	.	.	.	.	.	.	.	.	.	.	.	.	.	.	.	.	.	.	.	.	.	.	.	.
Petroravenia friesii	.	.	.	1	.	.	.	1	.	.	.	.	.	.	.	.	.	.	.	.	.	.	.	.	1	.	1	.	.
Petroravenia werdermannii	.	.	.	.	.	.	.	.	.	.	.	.	.	.	.	.	.	.	.	.	.	.	.	.	.	.	.	.	.
Phleum alpinum	.	.	.	.	.	.	.	.	.	.	.	.	.	.	.	.	.	.	.	.	.	.	.	.	.	.	.	.	.
Phylloscirpus acaulis	.	.	.	.	.	.	.	.	.	.	.	.	.	.	.	.	.	.	.	.	.	.	.	.	.	.	.	.	.
Phylloscirpus boliviensis	.	1	1	1	1	1	1	1	.	1	1	1	.	.	.	.	.	.	.	.	.	.	.	.	.	.	1	.	1
Phylloscirpus deserticola	1	1	1	1	1	1	1	1	1	.	1	1	1	1	1	1	1	1	1	1	.	1	.	.	1	1	.	1	1
Pinguicula antarctica	.	.	.	.	.	.	.	.	.	.	.	.	.	.	.	.	.	.	.	.	.	.	.	.	.	.	.	.	.
Plantago barbata	.	.	.	.	.	.	.	.	.	.	.	.	.	.	.	.	.	.	.	.	.	.	.	.	.	.	.	.	.
Plantago rigida	.	.	.	.	.	.	.	.	.	.	.	.	.	.	.	.	.	.	.	.	.	.	.	.	.	.	.	.	.
Plantago tubulosa	1	.	.	1	1	1	1	1	1	.	1	1	1	1	1	1	1	1	1	1	1	1	1	.	1	1	.	1	1

**Bog**	**233**	**234**	**235**	**236**	**237**	**238**	**239**	**240**	**241**	**242**	**243**	**244**	**245**	**246**	**247**	**248**	**249**	**250**	**251**	**252**	**253**	**254**	**255**	**256**	**257**	**258**	**259**	**260**	**261**
Plantago uniglumis	.	.	.	.	.	.	.	.	.	.	.	.	.	.	.	.	.	.	.	.	.	.	.	.	.	.	.	.	.
Poa alopecurus	.	.	.	.	.	.	.	.	.	.	.	.	.	.	.	.	.	.	.	.	.	.	.	1	.	.	.	.	.
Poa hachadoensis	.	.	.	.	.	.	.	.	.	.	.	.	.	.	.	.	.	.	.	.	.	.	.	.	.	.	.	.	.
Poa perligulata	1	.	1	.	1	.	.	.	.	.	.	.	.	.	.	.	.	.	.	.	1	.	.	.	1	1	1	.	.
Polypogon interruptus	.	.	.	.	.	.	.	.	.	.	.	.	.	.	.	.	.	.	.	.	.	.	.	.	.	.	.	.	.
Primula magellanica	.	.	.	.	.	.	.	.	.	.	.	.	.	.	.	.	.	.	.	.	.	.	.	.	.	.	.	.	.
Puccinellia frigida	.	.	.	.	.	.	.	.	.	.	.	.	.	.	.	.	.	.	.	.	.	.	.	.	.	.	.	.	.
Quinchamalium chilense	.	.	.	.	.	.	.	.	.	.	.	.	.	.	.	.	.	.	.	.	.	.	.	1	.	.	.	.	.
Ranunculus breviscapus	.	.	.	.	.	.	.	.	.	.	.	.	.	.	.	.	.	.	.	.	.	.	.	.	.	.	.	.	.
Ranunculus fuegianus	.	.	.	.	.	.	.	.	.	.	.	.	.	.	.	.	.	.	.	.	.	.	.	.	.	.	.	.	.
Ranunculus mandonianus	.	.	.	.	.	.	.	.	.	.	.	.	.	.	1	.	.	.	.	.	.	.	.	.	.	.	.	.	.
Ranunculus peduncularis	.	.	.	.	.	.	.	.	.	.	.	.	.	.	.	.	.	.	.	.	.	.	.	.	.	.	.	.	.
Ranunculus trichophyllus	.	.	.	.	.	1	.	.	.	.	.	.	1	.	.	.	.	.	.	.	.	.	.	.	1	.	.	.	1
Halerpestes uniflora	.	.	.	.	.	.	.	.	.	.	.	.	.	.	.	.	.	.	.	.	.	.	.	.	1	.	.	1	1
Rubus geoides	.	.	.	.	.	.	.	.	.	.	.	.	.	.	.	.	.	.	.	.	.	.	.	1	.	.	.	.	.
Rumex magellanicus	.	.	.	.	.	.	.	.	.	.	.	.	.	.	.	.	.	.	.	.	.	.	.	.	.	.	.	.	.
Rytidosperma lechleri	.	.	.	.	.	.	.	.	.	.	.	.	.	.	.	.	.	.	.	.	.	.	.	1	.	.	.	.	.
Sarcocornia pulvinata	.	.	.	.	.	.	.	.	.	.	.	.	.	.	.	.	.	.	.	.	.	.	.	.	.	.	.	.	.
Schoenoplectus pungens	.	.	.	.	.	.	.	.	.	.	.	.	.	.	.	.	.	.	.	.	.	.	.	.	.	.	.	.	.
Schoenus andinus	.	.	.	.	.	.	.	.	.	.	.	.	.	.	.	.	.	.	.	.	.	.	.	.	.	.	.	.	.
Senecio breviscapus	.	.	.	1	1	.	.	.	1	.	.	.	.	.	1	.	.	1	.	.	.	.	.	.	.	.	.	.	.
Senecio diemii	.	.	.	.	.	.	.	.	.	.	.	.	.	.	.	.	.	.	.	.	.	.	.	.	.	.	.	.	.
Senecio fistulosus	.	.	.	.	.	.	.	.	.	.	.	.	.	.	.	.	.	.	.	.	.	.	.	.	.	.	.	.	.
Senecio parodii	.	.	.	.	.	.	.	.	.	.	.	.	.	.	.	.	.	.	.	.	.	.	.	.	.	.	.	.	.
Senecio peteroanus	.	.	.	.	.	.	.	.	.	.	.	.	.	.	.	.	.	.	.	.	.	.	.	.	.	.	.	.	.
Senecio serratifolius	1	.	.	.	.	.	.	.	.	.	.	.	.	.	.	.	.	.	.	.	.	.	.	.	.	.	1	.	1
Senecio trifurcatus	.	.	.	.	.	.	.	.	.	.	.	.	.	.	.	.	.	.	.	.	.	.	.	1	.	.	.	.	.
Sisyrinchium chilense	.	.	.	.	.	.	.	.	.	.	.	.	.	.	.	.	.	.	.	.	.	.	.	.	.	.	.	.	.
Sisyrinchium patagonicum	.	.	.	.	.	.	.	.	.	.	.	.	.	.	.	.	.	.	.	.	.	.	.	.	.	.	.	.	.
Sisyrinchium pearcei	.	.	.	.	.	.	.	.	.	.	.	.	.	.	.	.	.	.	.	.	.	.	.	1	.	.	.	.	.
Stellaria debilis	.	.	.	.	.	.	.	.	.	.	.	.	.	.	.	.	.	.	.	.	.	.	.	.	.	.	.	.	.
Stuckenia filiformis	.	.	.	.	.	.	.	.	.	.	.	.	.	.	.	1	.	.	.	.	.	.	.	.	.	.	.	.	.

Bog	233	234	235	236	237	238	239	240	241	242	243	244	245	246	247	248	249	250	251	252	253	254	255	256	257	258	259	260	261
Stuckenia striata	.	.	.	.	.	.	.	.	.	.	.	.	.	.	.	.	.	.	.	.	.	.	.	.	.	.	.	.	.
Symphyotrichum peteroanum	.	.	.	.	.	.	.	.	.	.	.	.	.	.	.	.	.	.	.	.	.	.	.	.	.	.	.	.	.
Symphyotrichum vahlii	.	.	.	.	.	.	.	.	.	.	.	.	.	.	.	.	.	.	.	.	.	.	.	.	.	.	.	.	.
Tetroncium magellanicum	.	.	.	.	.	.	.	.	.	.	.	.	.	.	.	.	.	.	.	.	.	.	.	.	.	.	.	.	.
Tribeles australis	.	.	.	.	.	.	.	.	.	.	.	.	.	.	.	.	.	.	.	.	.	.	.	1	.	.	.	.	.
Trifolium amabile	.	.	.	.	.	.	.	.	.	.	.	.	.	.	.	.	.	.	.	.	.	.	.	.	.	.	.	.	.
Trifolium polymorphum	.	.	.	.	.	.	.	.	.	.	.	.	.	.	.	.	.	.	.	.	.	.	.	.	.	.	.	.	.
Triglochin concinna	.	.	.	.	.	.	.	.	.	.	.	.	.	.	.	.	.	.	.	.	.	.	.	.	.	.	.	.	.
Triglochin palustris	.	.	.	.	.	.	.	.	.	.	.	.	.	.	.	.	.	.	.	.	.	.	.	.	.	.	.	.	.
Triglochin striata	.	.	.	.	.	.	.	.	.	.	.	.	.	.	.	.	.	.	.	.	.	.	.	.	.	.	.	.	.
Trisetum caudulatum	.	.	.	.	.	.	.	.	.	.	.	.	.	.	.	.	.	.	.	.	.	.	.	.	.	.	.	.	.
Trisetum preslei	.	.	.	.	.	.	.	.	.	.	.	.	.	.	.	.	.	.	.	.	.	.	.	.	.	.	.	.	.
Koeleria spicata	.	.	.	.	.	.	.	.	.	.	.	.	.	.	.	.	.	.	.	.	.	.	.	1	.	.	.	.	.
Utricularia gibba	.	.	.	.	.	.	.	.	.	.	.	.	.	.	.	.	.	.	.	.	.	.	.	.	.	.	.	.	.
Vahlodea atropurpurea	.	.	.	.	.	.	.	.	.	.	.	.	.	.	.	.	.	.	.	.	.	.	.	.	.	.	.	.	.
Valeriana fonckii	.	.	.	.	.	.	.	.	.	.	.	.	.	.	.	.	.	.	.	.	.	.	.	1	.	.	.	.	.
Valeriana macrorhiza	.	.	.	.	.	.	.	.	.	.	.	.	.	.	.	.	.	.	.	.	.	.	.	.	.	.	.	.	.
Viola pygmaea	.	.	.	.	.	.	.	.	.	.	.	.	.	.	.	.	.	.	.	.	.	.	.	.	.	.	.	.	.
Werneria apiculata	.	.	.	.	.	.	.	.	.	.	.	.	.	.	.	.	.	.	.	.	.	.	.	.	.	.	.	.	.
Werneria pinnatifida	.	.	1	1	1	.	1	.	.	.	.	.	1	.	.	.	.	.	1	1	1	1	1	.	1	.	.	1	1
Werneria pygmaea	1	.	1	1	1	1	1	1	1	.	1	1	1	1	1	1	1	1	1	1	1	1	1	.	1	1	1	.	1
Werneria solivifolia	.	.	1	.	.	1	1	1	1	.	.	1	1	.	.	.	.	.	1	.	.	.	.	.	.	.	1	1	1
Werneria spathulata	1	1	1	1	.	.	.	.	1	.	.	.	.	.	.	.	.	1	.	.	.	.	.	.	.	.	1	1	.
Xenophyllum incisum	.	.	.	.	.	.	.	.	.	.	1	.	.	.	.	.	.	.	.	.	.	.	.	.	.	.	.	.	.
Zameioscirpus atacamensis	.	.	.	.	.	.	.	.	.	.	.	.	.	.	.	.	.	.	.	.	.	.	.	.	.	.	.	.	.
Zameioscirpus gaimardiodes	.	.	.	.	.	.	.	.	.	.	.	.	.	.	.	.	.	.	.	.	.	.	.	.	.	.	.	.	.
Zameioscirpus muticus	1	1	1	.	1	1	.	1	.	1	1	.	.	.	.	1	.	.	1	1	1	1	1	.	1	1	1	.	1

Bog	262	263	264	265	266	267	268	269	270	271	272	273	274	275	276	277	278	279	280	281	282	283	284	285	286	287	288	289	290
**Operational zone**	N	N	N	N	N	N	N	N	N	N	N	N	N	N	N	N	N	N	N	N	N	N	N	N	N	N	N	N	N
**Cluster**	1	1	1	1	1	1	1	1	1	1	1	1	1	1	1	1	1	1	1	1	1	1	1	1	1	1	1	1	1
**Bioregion**	N	N	N	N	N	N	N	N	N	N	N	N	N	N	N	N	N	N	N	N	N	N	N	N	N	N	N	N	N
**Longitude**	-71.58	-71.63	-65.56	-66.28	-65.66	-66.05	-65.75	-66.43	-65.92	-65.66	-65.67	-65.67	-65.41	-66.07	-65.69	-66.56	-66.57	-66.52	-65.97	-66.65	-66.64	-66.58	-66.52	-66.91	-66.08	-66.91	-66.66	-66.97	-67.03
**Latitude**	-15.55	-15.71	-19.77	-19.11	-19.82	-19.78	-19.67	-19.27	-19.73	-19.82	-19.77	-19.77	-19.77	-19.27	-19.65	-21.77	-21.63	-21.58	-19.77	-20.01	-20.02	-20.13	-21.8	-21.86	-19.8	-21.86	-19.98	-21.95	-21.97
Acaena antarctica	.	.	.	.	.	.	.	.	.	.	.	.	.	.	.	.	.	.	.	.	.	.	.	.	.	.	.	.	.
Acaena macrocephala	.	.	.	.	.	.	.	.	.	.	.	.	.	.	.	.	.	.	.	.	.	.	.	.	.	.	.	.	.
Acaena magellanica	.	.	.	.	.	.	.	.	.	.	.	.	.	.	.	.	.	.	.	.	.	.	.	.	.	.	.	.	.
Acaena ovalifolia	.	.	.	.	.	.	.	.	.	.	.	.	.	.	.	.	.	.	.	.	.	.	.	.	.	.	.	.	.
Acaena pinnatifida	.	.	.	.	.	.	.	.	.	.	.	.	.	.	.	.	.	.	.	.	.	.	.	.	.	.	.	.	.
Adesmia retusa	.	.	.	.	.	.	.	.	.	.	.	.	.	.	.	.	.	.	.	.	.	.	.	.	.	.	.	.	.
Agrostis breviculmis	.	.	.	.	.	.	.	.	.	.	.	.	.	.	.	.	.	.	.	.	.	.	.	.	.	.	.	.	.
Agrostis imberbis	.	.	.	.	.	.	.	.	.	.	.	.	.	.	.	.	.	.	.	.	.	.	.	.	.	.	.	.	.
Agrostis meyenii	.	.	.	.	.	.	.	.	.	.	.	.	.	.	.	.	.	.	.	.	.	.	.	.	.	.	.	.	.
Agrostis perennans	.	.	.	.	.	.	.	.	.	.	.	.	.	.	.	.	.	.	.	.	.	.	.	.	.	.	.	.	.
Alchemilla pinnata	.	.	.	.	.	.	.	.	.	.	.	.	.	.	.	.	.	.	.	.	.	.	.	.	.	.	.	.	.
Alopecurus magellanicus	.	.	.	.	.	.	.	.	.	.	.	.	.	.	.	.	.	.	.	.	.	.	.	.	.	.	.	.	.
Amphiscirpus nevadensis	.	.	.	.	.	.	.	.	.	.	.	.	.	.	.	.	.	.	.	.	.	.	.	.	.	.	.	.	.
Anagallis alternifolia	.	.	.	.	.	.	.	.	.	.	.	.	.	.	.	.	.	.	.	.	.	.	.	.	.	.	.	.	.
Antennaria chilensis	.	.	.	.	.	.	.	.	.	.	.	.	.	.	.	.	.	.	.	.	.	.	.	.	.	.	.	.	.
Anthoxanthum redolens	.	.	.	.	.	.	.	.	.	.	.	.	.	.	.	.	.	.	.	.	.	.	.	.	.	.	.	.	.
Apium panul	.	.	.	.	.	.	.	.	.	.	.	.	.	.	.	.	.	.	.	.	.	.	.	.	.	.	.	.	.
Arenaria rivularis	.	.	.	.	.	.	.	.	.	.	.	.	.	.	.	.	.	.	.	.	.	.	.	.	.	.	.	.	.
Arenaria serpens	.	.	.	.	.	.	.	.	.	.	.	.	.	.	.	.	.	.	.	.	.	.	.	.	.	.	.	.	.
Arjona pusilla	.	.	.	1	.	.	.	.	.	.	.	.	.	.	.	.	.	.	.	.	.	.	.	.	.	.	.	.	.
Astragalus bustillosii	.	.	.	.	.	.	.	.	.	.	.	.	.	.	.	.	.	.	.	.	.	.	.	.	.	.	.	.	.
Astragalus micranthellus	.	.	.	.	.	.	.	.	.	.	.	.	.	.	.	.	.	.	.	.	.	.	.	.	.	.	.	.	.
Azolla filiculoides	1	1	.	.	.	.	.	.	.	.	.	.	.	.	.	.	.	.	.	.	.	.	.	.	.	.	.	.	.
Azorella boelckei	.	.	.	.	.	.	.	.	.	.	.	.	.	.	.	.	.	.	.	.	.	.	.	.	.	.	.	.	.

Bog	262	263	264	265	266	267	268	269	270	271	272	273	274	275	276	277	278	279	280	281	282	283	284	285	286	287	288	289	290
Azorella burkartii	.	.	.	.	.	.	.	.	.	.	.	.	.	.	.	.	.	.	.	.	.	.	.	.	.	.	.	.	.
Azorella cryptantha	.	.	.	.	.	.	.	.	.	.	.	.	.	.	.	.	.	.	.	.	.	.	.	.	.	.	.	.	.
Azorella lycopodioides	.	.	.	.	.	.	.	.	.	.	.	.	.	.	.	.	.	.	.	.	.	.	.	.	.	.	.	.	.
Azorella trifoliolata	.	.	.	.	.	.	.	.	.	.	.	.	.	.	.	.	.	.	.	.	.	.	.	.	.	.	.	.	.
Baccharis acaulis	.	.	.	.	.	.	.	.	.	.	.	.	.	.	.	.	.	.	.	.	.	.	.	.	.	.	.	.	.
Baccharis caespitosa	.	.	.	.	.	.	.	.	.	.	.	.	.	.	.	.	.	.	.	.	.	.	.	.	.	.	.	.	.
Baccharis magellanica	.	.	.	.	.	.	.	.	.	.	.	.	.	.	.	.	.	.	.	.	.	.	.	.	.	.	.	.	.
Belloa chilensis	.	.	.	.	.	.	.	.	.	.	.	.	.	.	.	.	.	.	.	.	.	.	.	.	.	.	.	.	.
Bromus catharticus	.	.	.	.	.	.	.	.	.	.	.	.	.	.	.	.	.	.	.	.	.	.	.	.	.	.	.	.	.
Calandrinia acaulis	.	.	.	.	.	.	.	.	.	.	.	.	.	.	.	.	.	.	.	.	.	.	.	.	.	.	.	.	.
Calandrinia compacta	.	.	1	.	.	1	1	.	1	.	.	.	.	.	.	.	1	1	1	1	.	.	1	1	1	1	.	1	.
Calceolaria biflora	.	.	.	.	.	.	.	.	.	.	.	.	.	.	.	.	.	.	.	.	.	.	.	.	.	.	.	.	.
Calceolaria cana	.	.	.	.	.	.	.	.	.	.	.	.	.	.	.	.	.	.	.	.	.	.	.	.	.	.	.	.	.
Calceolaria corymbosa	.	.	.	.	.	.	.	.	.	.	.	.	.	.	.	.	.	.	.	.	.	.	.	.	.	.	.	.	.
Calceolaria filicaulis	.	.	.	.	.	.	.	.	.	.	.	.	.	.	.	.	.	.	.	.	.	.	.	.	.	.	.	.	.
Callitriche lechleri	.	.	.	.	.	.	.	.	.	.	.	.	.	.	.	.	.	.	.	.	.	.	.	.	.	.	.	.	.
Caltha appendiculata	.	.	.	.	.	.	.	.	.	.	.	.	.	.	.	.	.	.	.	.	.	.	.	.	.	.	.	.	.
Caltha sagittata	.	.	.	.	.	.	.	.	.	.	.	.	.	.	.	.	.	.	.	.	.	.	.	.	.	.	.	.	.
Cardamine cordata	.	.	.	.	.	.	.	.	.	.	.	.	.	.	.	.	.	.	.	.	.	.	.	.	.	.	.	.	.
Cardamine glacialis	.	.	.	.	.	.	.	.	.	.	.	.	.	.	.	.	.	.	.	.	.	.	.	.	.	.	.	.	.
Cardamine tenuirostris	.	.	.	.	.	.	.	.	.	.	.	.	.	.	.	.	.	.	.	.	.	.	.	.	.	.	.	.	.
Cardamine volckmannii	.	.	.	.	.	.	.	.	.	.	.	.	.	.	.	.	.	.	.	.	.	.	.	.	.	.	.	.	.
Carex acaulis	.	.	.	.	.	.	.	.	.	.	.	.	.	.	.	.	.	.	.	.	.	.	.	.	.	.	.	.	.
Carex atropicta	.	.	.	.	.	.	.	.	.	.	.	.	.	.	.	.	.	.	.	.	.	.	.	.	.	.	.	.	.
Carex banksii	.	.	.	.	.	.	.	.	.	.	.	.	.	.	.	.	.	.	.	.	.	.	.	.	.	.	.	.	.
Carex caduca	.	.	.	.	.	.	.	.	.	.	.	.	.	.	.	.	.	.	.	.	.	.	.	.	.	.	.	.	.
Carex decidua	.	.	.	.	.	.	.	.	.	.	.	.	.	.	.	.	.	.	.	.	.	.	.	.	.	.	.	.	.
Carex fuscula	.	.	.	.	.	.	.	.	.	.	.	.	.	.	.	.	.	.	.	.	.	.	.	.	.	.	.	.	.
Carex gayana	.	.	.	.	.	.	.	.	.	.	.	.	.	.	.	.	.	.	.	.	.	.	.	.	.	.	.	.	.
Carex hypoleucos	.	.	.	.	.	.	.	.	.	.	.	.	.	.	.	.	.	.	.	.	.	.	.	.	.	.	.	.	.
Carex macloviana	.	.	.	.	.	.	.	.	.	.	.	.	.	.	.	.	.	.	.	.	.	.	.	.	.	.	.	.	.
Carex magellanica	.	.	.	.	.	.	.	.	.	.	.	.	.	.	.	.	.	.	.	.	.	.	.	.	.	.	.	.	.
Carex malmei	.	.	.	.	.	.	.	.	.	.	.	.	.	.	.	.	.	.	.	.	.	.	.	.	.	.	.	.	.
Carex maritima	.	1	.	1	.	1	1	.	1	.	1	.	1	.	1	1	1	1	.	1	1	1	1	1	.	1	1	1	1
Carex microglochin	.	.	.	.	.	.	.	.	.	.	.	.	.	.	.	.	.	.	.	.	.	.	.	.	.	.	.	.	.
Carex pleioneura	.	.	.	.	.	.	.	.	.	.	.	.	.	.	.	.	.	.	.	.	.	.	.	.	.	.	.	.	.
Carex ruthsatzae	.	.	.	.	.	.	.	.	.	.	.	.	.	.	.	.	.	.	.	.	.	.	.	.	.	.	.	.	.

**Bog**	**262**	**263**	**264**	**265**	**266**	**267**	**268**	**269**	**270**	**271**	**272**	**273**	**274**	**275**	**276**	**277**	**278**	**279**	**280**	**281**	**282**	**283**	**284**	**285**	**286**	**287**	**288**	**289**	**290**
Carex vallis-pulchrae	.	.	.	.	.	.	.	.	.	.	.	.	.	.	.	.	.	.	.	.	.	.	.	.	.	.	.	.	.
Carpha schoenoides	.	.	.	.	.	.	.	.	.	.	.	.	.	.	.	.	.	.	.	.	.	.	.	.	.	.	.	.	.
Castilleja pumila	1	1	1	1	1	1	1	1	1	.	.	.	1	1	.	.	.	.	1	.	1	1	1	.	.	.	.	.	.
Catabrosa werdermannii	.	.	.	.	.	.	.	.	.	.	.	.	.	.	.	.	.	.	.	.	.	.	.	.	.	.	.	.	.
Cerastium humifusum	.	.	.	.	.	.	.	.	.	.	.	.	.	.	.	.	.	.	.	.	.	.	.	.	.	.	.	.	.
Cerastium montioides	.	.	.	.	.	.	.	.	.	.	.	.	.	.	.	.	.	.	.	.	.	.	.	.	.	.	.	.	.
Chiliotrichum diffusum	.	.	.	.	.	.	.	.	.	.	.	.	.	.	.	.	.	.	.	.	.	.	.	.	.	.	.	.	.
Chusquea culeou	.	.	.	.	.	.	.	.	.	.	.	.	.	.	.	.	.	.	.	.	.	.	.	.	.	.	.	.	.
Colobanthus quitensis	.	1	1	1	1	1	1	.	1	.	.	1	.	.	1	.	.	.	1	1	1	1	1	1	1	1	1	1	.
Cortaderia egmontiana	.	.	.	.	.	.	.	.	.	.	.	.	.	.	.	.	.	.	.	.	.	.	.	.	.	.	.	.	.
Cotula mexicana	.	1	1	1	1	1	1	1	1	1	.	1	.	1	.	.	.	.	1	1	1	1	.	.	1	.	.	.	.
Crassula peduncularis	.	.	.	.	.	.	.	.	.	.	.	.	.	.	.	.	.	.	.	.	.	.	.	.	.	.	.	.	.
Cuatrecasasiella argentina	.	.	1	.	1	1	1	1	1	1	.	1	.	.	.	.	.	.	.	1	.	.	.	.	1	.	.	.	.
Deschampsia antarctica	.	.	.	.	.	.	.	.	.	.	.	.	.	.	.	.	.	.	.	.	.	.	.	.	.	.	.	.	.
Deschampsia caespitosa	.	.	.	.	.	.	.	.	.	.	.	.	.	.	.	.	.	.	.	.	.	.	.	.	.	.	.	.	.
Deschampsia patula	.	.	.	.	.	.	.	.	.	.	.	.	.	.	.	.	.	.	.	.	.	.	.	.	.	.	.	.	.
Cinnagrostis brevifolia	.	1	.	.	.	.	.	.	.	.	.	.	.	.	.	.	.	.	.	.	.	.	.	.	.	.	.	.	.
Deschampsia chrysantha	.	1	.	.	.	.	.	.	.	.	.	1	.	.	.	1	.	.	.	.	.	.	.	.	.	.	.	1	1
Cinnagrostis chrysophylla	.	.	.	.	.	.	.	.	.	.	.	.	.	.	.	.	.	.	.	.	.	.	.	.	.	.	.	.	.
Deschampsia chrysostachya	.	.	.	.	.	.	.	.	.	.	.	.	.	.	.	.	.	.	.	.	.	.	.	.	.	.	.	.	.
Deschampsia eminens	.	.	.	.	.	.	.	.	.	.	.	.	.	.	1	.	.	.	.	.	.	.	.	.	.	.	.	.	.
Deschampsia hackelii	.	.	.	.	.	.	.	.	.	.	.	.	.	.	.	.	.	.	.	.	.	.	.	.	.	.	.	.	.
Cinnagrostis minima	.	.	.	.	.	.	.	.	.	.	.	.	.	.	.	.	.	.	.	.	.	.	.	.	.	.	.	.	.
Deschampsia ovata	.	.	.	.	.	.	.	.	.	.	.	.	.	.	.	.	.	.	.	.	.	.	.	.	.	.	.	.	.
Cinnagrostis rigescens	1	.	1	1	1	1	1	1	1	1	.	1	.	1	.	.	.	.	1	.	1	1	1	.	1	.	.	.	.
Cinnagrostis spicigera	.	.	.	.	.	.	.	.	.	.	1	.	.	.	.	.	.	.	.	.	.	.	.	.	.	.	.	.	.
Cinnagrostis velutina	.	.	.	.	.	.	.	.	.	.	.	.	.	.	.	.	.	.	.	.	.	.	.	.	.	.	.	.	.
Cinnagrostis vicunarum	.	.	.	.	.	.	.	1	.	.	.	.	.	.	.	.	.	.	.	.	.	.	.	.	.	.	.	.	.
Distichia filamentosa	.	.	.	.	.	.	.	.	.	.	.	.	.	.	.	.	.	.	.	.	.	.	.	.	.	.	.	.	.
Distichia muscoides	1	1	.	1	1	1	1	1	1	1	1	1	1	.	1	1	1	1	1	1	1	1	1	1	.	1	1	1	1
Distichlis humilis	.	.	.	.	.	.	.	.	.	.	.	.	.	.	.	.	.	.	.	.	.	.	.	.	.	.	.	.	.

Bog	262	263	264	265	266	267	268	269	270	271	272	273	274	275	276	277	278	279	280	281	282	283	284	285	286	287	288	289	290
Distichlis scoparia	.	.	.	.	.	.	.	.	.	.	.	.	.	.	.	.	.	.	.	.	.	.	.	.	.	.	.	.	.
Distichlis spicata	.	.	.	.	.	.	.	.	.	.	.	.	.	.	.	.	.	.	.	.	.	.	.	.	.	.	.	.	.
Draba pusilla	.	.	.	.	.	.	.	.	.	.	.	.	.	.	.	.	.	.	.	.	.	.	.	.	.	.	.	.	.
Eleocharis melanomphala	.	.	.	.	.	.	.	.	.	.	.	.	.	.	.	.	.	.	.	.	.	.	.	.	.	.	.	.	.
Eleocharis pseudoalbibracteata	.	.	.	.	.	.	.	.	.	.	.	.	.	.	.	.	.	.	.	.	.	.	.	.	.	.	.	.	.
Elodea potamogeton	1	1	.	.	.	.	.	.	.	.	.	.	.	.	.	.	.	.	.	.	.	.	.	.	1	.	.	.	.
Empetrum rubrum	.	.	.	.	.	.	.	.	.	.	.	.	.	.	.	.	.	.	.	.	.	.	.	.	.	.	.	.	.
Epilobium australe	.	.	.	.	.	.	.	.	.	.	.	.	.	.	.	.	.	.	.	.	.	.	.	.	.	.	.	.	.
Epilobium barbeyanum	.	.	.	.	.	.	.	.	.	.	.	.	.	.	.	.	.	.	.	.	.	.	.	.	.	.	.	.	.
Epilobium ciliatum	.	.	.	.	.	.	.	.	.	.	.	.	.	.	.	.	.	.	.	.	.	.	.	.	.	.	.	.	.
Epilobium denticulatum	.	.	1	.	.	.	.	.	.	.	.	.	.	.	.	.	.	.	.	.	.	.	.	.	.	.	.	.	.
Epilobium fragile	.	.	.	.	.	.	.	.	1	.	.	.	.	.	.	.	.	.	.	.	.	.	.	.	.	.	.	.	.
Epilobium glaucum	.	.	.	.	.	.	.	.	.	.	.	.	.	.	.	.	.	.	.	.	.	.	.	.	.	.	.	.	.
Epilobium nivale	.	.	.	.	.	.	.	.	.	.	.	.	.	.	.	.	.	.	.	.	.	.	.	.	.	.	.	.	.
Erigeron andicola	.	.	.	.	.	.	.	.	.	.	.	.	.	.	.	.	.	.	.	.	.	.	.	.	.	.	.	.	.
Erigeron leptopetalus	.	.	.	.	.	.	.	.	.	.	.	.	.	.	.	.	.	.	.	.	.	.	.	.	.	.	.	.	.
Erigeron myosotis	.	.	.	.	.	.	.	.	.	.	.	.	.	.	.	.	.	.	.	.	.	.	.	.	.	.	.	.	.
Erigeron patagonicus	.	.	.	.	.	.	.	.	.	.	.	.	.	.	.	.	.	.	.	.	.	.	.	.	.	.	.	.	.
Erythranthe cuprea	.	.	.	.	.	.	.	.	.	.	.	.	.	.	.	.	.	.	.	.	.	.	.	.	.	.	.	.	.
Erythranthe depressa	.	.	.	.	.	.	.	.	.	.	.	.	.	.	.	.	.	.	.	.	.	.	.	.	.	.	.	.	.
Erythranthe glabrata	.	.	1	1	.	1	.	.	1	.	.	.	.	1	.	.	.	.	.	.	.	.	.	.	1	.	.	.	.
Erythranthe lutea	.	.	.	.	.	.	.	.	.	.	.	.	.	.	.	.	.	.	.	.	.	.	.	.	.	.	.	.	.
Escallonia virgata	.	.	.	.	.	.	.	.	.	.	.	.	.	.	.	.	.	.	.	.	.	.	.	.	.	.	.	.	.
Euphrasia antarctica	.	.	.	.	.	.	.	.	.	.	.	.	.	.	.	.	.	.	.	.	.	.	.	.	.	.	.	.	.
Euphrasia chrysantha	.	.	.	.	.	.	.	.	.	.	.	.	.	.	.	.	.	.	.	.	.	.	.	.	.	.	.	.	.
Euphrasia subexserta	.	.	.	.	.	.	.	.	.	.	.	.	.	.	.	.	.	.	.	.	.	.	.	.	.	.	.	.	.
Festuca hypsophila	.	.	.	.	.	.	.	.	.	.	.	.	.	.	.	.	.	.	.	.	.	.	.	.	.	.	.	.	.
Festuca kurtziana	.	.	.	.	.	.	.	.	.	.	.	.	.	.	.	.	.	.	.	.	.	.	.	.	.	.	.	.	.
Festuca lilloi	.	.	.	.	.	.	.	.	.	.	.	.	.	.	.	.	.	.	.	.	.	.	.	.	.	.	.	.	.
Festuca magellanica	.	.	.	.	.	.	.	.	.	.	.	.	.	.	.	.	.	.	.	.	.	.	.	.	.	.	.	.	.
Festuca nardifolia	.	.	.	.	.	.	.	.	.	.	.	.	.	.	.	.	.	.	.	.	.	.	.	.	.	.	.	.	.
Festuca rigescens	.	.	.	.	.	.	.	.	.	.	.	.	.	.	.	.	.	.	.	.	.	.	.	.	.	.	.	.	.
Festuca werdermannii	.	.	.	.	.	.	.	.	.	.	.	.	.	.	.	.	.	.	.	.	.	.	.	.	.	.	.	.	.
Frankenia triandra	.	.	.	.	.	.	.	.	.	.	.	.	.	.	.	.	.	.	.	.	.	.	.	.	.	.	.	.	.
Gamocarpha graminea	.	.	.	.	.	.	.	.	.	.	.	.	.	.	.	.	.	.	.	.	.	.	.	.	.	.	.	.	.

**Bog**	**262**	**263**	**264**	**265**	**266**	**267**	**268**	**269**	**270**	**271**	**272**	**273**	**274**	**275**	**276**	**277**	**278**	**279**	**280**	**281**	**282**	**283**	**284**	**285**	**286**	**287**	**288**	**289**	**290**
Gamocarpha ventosa	.	.	.	.	.	.	.	.	.	.	.	.	.	.	.	.	.	.	.	.	.	.	.	.	.	.	.	.	.
Gamochaeta chamissonis	.	.	.	.	.	.	.	.	.	.	.	.	.	.	.	.	.	.	.	.	.	.	.	.	.	.	.	.	.
Gamochaeta longipedicellata	.	.	.	.	.	.	.	.	.	.	.	.	.	.	.	.	.	.	.	.	.	.	.	.	.	.	.	.	.
Gamochaeta neuquensis	.	.	.	.	.	.	.	.	.	.	.	.	.	.	.	.	.	.	.	.	.	.	.	.	.	.	.	.	.
Gaultheria antarctica	.	.	.	.	.	.	.	.	.	.	.	.	.	.	.	.	.	.	.	.	.	.	.	.	.	.	.	.	.
Gaultheria caespitosa	.	.	.	.	.	.	.	.	.	.	.	.	.	.	.	.	.	.	.	.	.	.	.	.	.	.	.	.	.
Gaultheria pumila	.	.	.	.	.	.	.	.	.	.	.	.	.	.	.	.	.	.	.	.	.	.	.	.	.	.	.	.	.
Gavilea chica	.	.	.	.	.	.	.	.	.	.	.	.	.	.	.	.	.	.	.	.	.	.	.	.	.	.	.	.	.
Gentiana prostrata	.	1	1	1	1	1	1	.	1	1	1	1	1	1	1	1	1	1	1	1	.	1	1	1	1	1	1	.	1
Gentianella fiebrigii	.	.	.	.	.	.	.	.	.	.	.	.	.	.	.	.	.	.	.	.	.	.	.	.	.	.	.	.	.
Gentianella magellanica	.	.	.	.	.	.	.	.	.	.	.	.	.	.	.	.	.	.	.	.	.	.	.	.	.	.	.	.	.
Gentianella multicaulis	.	.	.	.	.	.	.	.	.	.	.	.	.	.	.	.	.	.	.	.	.	.	.	.	.	.	.	.	.
Gentianella ottonis	.	.	.	.	.	.	.	.	.	.	.	.	.	.	.	.	.	.	.	.	.	.	.	.	.	.	.	.	.
Gentianella primuloides	.	.	.	.	.	.	.	.	.	.	.	.	.	.	.	.	.	.	.	.	.	.	.	.	.	.	.	.	.
Gentianella pseudocrassula	.	.	.	.	.	.	.	.	.	.	.	.	.	.	.	.	.	.	.	.	.	.	.	.	.	.	.	.	.
Geranium sessiliflorum	.	.	.	.	.	.	.	.	.	.	.	.	.	.	.	.	.	.	.	.	.	.	.	.	.	.	.	.	.
Gunnera magellanica	.	.	.	.	.	.	.	.	.	.	.	.	.	.	.	.	.	.	.	.	.	.	.	.	.	.	.	.	.
Halenia caespitosa	.	.	.	.	.	.	.	.	.	.	.	.	.	.	.	.	.	.	.	.	.	.	.	.	.	.	.	.	.
Halerpestes cymbalaria	.	.	.	.	.	.	.	.	.	.	.	.	.	.	.	.	.	.	.	.	.	.	.	.	.	.	.	.	.
Halerpestes exilis	.	.	.	.	.	.	.	.	.	.	.	.	.	.	.	1	1	1	.	1	.	.	.	1	.	1	.	1	.
Hieracium antarcticum	.	.	.	.	.	.	.	.	.	.	.	.	.	.	.	.	.	.	.	.	.	.	.	.	.	.	.	.	.
Hordeum comosum	.	.	.	.	.	.	.	.	.	.	.	.	.	.	.	.	.	.	.	.	.	.	.	.	.	.	.	.	.
Hordeum muticum	.	.	.	.	.	.	.	.	.	.	.	.	.	.	.	.	.	.	.	.	.	.	.	.	.	.	.	.	.
Hypochaeris acaulis	.	.	.	.	.	.	.	.	.	.	.	.	.	.	.	.	.	.	.	.	.	.	.	.	.	.	.	.	.
Hypochaeris chondrilloides	.	.	.	.	.	.	.	.	.	.	.	.	.	.	.	.	.	.	.	.	.	.	.	.	.	.	.	.	.
Hypochaeris meyeniana	.	.	.	.	.	.	.	.	.	.	.	.	.	.	.	.	.	.	.	.	.	.	.	.	.	.	.	.	.
Hypochaeris palustris	.	.	.	.	.	.	.	.	.	.	.	.	.	.	.	.	.	.	.	.	.	.	.	.	.	.	.	.	.
Hypochaeris taraxacoides	.	1	1	1	1	1	1	1	1	1	.	1	1	1	1	.	.	1	1	1	1	1	1	.	1	.	.	.	.
Hypochaeris tenerifolia	.	.	.	.	.	.	.	.	.	.	.	.	.	.	.	.	.	.	.	.	.	.	.	.	.	.	.	.	.
Isolepis nigricans	.	.	.	.	.	.	.	.	.	.	.	.	.	.	.	.	.	.	.	.	.	.	.	.	.	.	.	.	.

Bog	262	263	264	265	266	267	268	269	270	271	272	273	274	275	276	277	278	279	280	281	282	283	284	285	286	287	288	289	290
Isolepsis inundata	.	.	.	.	.	.	.	.	.	.	.	.	.	.	.	.	.	.	.	.	.	.	.	.	.	.	.	.	.
Juncus balticus	.	.	.	.	.	.	.	.	.	.	.	.	.	.	.	.	.	.	.	.	.	.	.	.	.	.	.	.	.
Juncus stipulatus	.	1	1	1	1	1	1	1	1	1	1	1	1	1	1	.	.	.	1	1	1	1	1	1	1	.	1	.	.
Koeleria kurtzii	.	.	.	.	.	.	.	.	.	.	.	.	.	.	.	.	.	.	.	.	.	.	.	.	.	.	.	.	.
Lachemilla diplophylla	1	1	1	1	1	1	1	1	1	.	.	1	1	1	.	.	.	.	1	1	1	.	.	1	1	.	.	.	.
Lachemilla pinnata	.	.	1	1	.	1	1	1	1	.	1	1	1	1	1	1	.	.	1	1	.	.	1	1	.	1	.	.	1
Lagenophora nudicaulis	.	.	.	.	.	.	.	.	.	.	.	.	.	.	.	.	.	.	.	.	.	.	.	.	.	.	.	.	.
Lemna minuta	.	.	.	1	.	.	.	.	.	.	.	.	.	1	.	.	.	.	1	.	.	.	.	1	1	.	.	.	.
Leptinella scariosa	.	.	.	.	.	.	.	.	.	.	.	.	.	.	.	.	.	.	.	.	.	.	.	.	.	.	.	.	.
Leucheria candidissima	.	.	.	.	.	.	.	.	.	.	.	.	.	.	.	.	.	.	.	.	.	.	.	.	.	.	.	.	.
Leucheria nutans	.	.	.	.	.	.	.	.	.	.	.	.	.	.	.	.	.	.	.	.	.	.	.	.	.	.	.	.	.
Lilaea scilloides	.	.	1	.	.	.	.	.	.	.	1	.	.	.	.	.	.	.	.	.	.	.	.	.	.	.	.	.	.
Lilaeopsis macloviana	1	1	1	1	.	1	1	1	1	.	.	.	.	1	.	1	1	.	1	1	1	1	1	1	1	1	1	1	.
Limosella australis	.	.	.	.	.	.	.	.	.	.	.	.	.	.	.	.	.	.	.	.	.	.	.	.	.	.	.	.	.
Lobelia oligophylla	1	1	1	1	1	1	1	1	1	1	1	1	1	1	1	1	1	.	.	1	1	1	1	1	1	1	1	1	1
Luzula brachyphylla	.	.	.	.	.	.	.	.	.	.	.	.	.	.	.	.	.	.	.	.	.	.	.	.	.	.	.	.	.
Luzula chilensis	.	.	.	.	.	.	.	.	.	.	.	.	.	.	.	.	.	.	.	.	.	.	.	.	.	.	.	.	.
Luzula racemosa	.	.	.	.	.	.	.	.	.	.	.	.	.	.	.	.	.	.	.	.	.	.	.	.	.	.	.	.	.
Luzula vulcanica	.	.	.	.	.	.	.	.	.	.	.	.	.	.	.	.	.	.	.	.	.	.	.	.	.	.	.	.	.
Lysipomia pumila	.	.	.	.	.	.	.	.	.	.	.	.	.	.	.	.	.	.	.	.	.	.	.	.	.	.	.	.	.
Marsippospermum philippii	.	.	.	.	.	.	.	.	.	.	.	.	.	.	.	.	.	.	.	.	.	.	.	.	.	.	.	.	.
Marsippospermum reichei	.	.	.	.	.	.	.	.	.	.	.	.	.	.	.	.	.	.	.	.	.	.	.	.	.	.	.	.	.
Montia fontana	.	.	.	.	.	.	.	.	.	.	.	.	.	.	1	.	.	.	1	.	.	.	1	1	.	.	.	.	.
Muhlenbergia asperifolia	.	.	.	.	.	.	.	.	.	.	.	.	.	.	.	.	.	.	.	.	.	.	.	.	.	.	.	.	.
Myriophyllum quitense	.	1	1	.	.	.	1	.	.	.	.	.	.	.	.	.	.	.	.	1	1	.	.	.	.	.	.	.	.
Myrosmodes nervosa	.	.	.	.	.	.	.	.	.	.	.	.	.	.	.	.	.	.	.	.	.	.	.	.	.	.	.	.	.
Myrosmodes paludosa	.	.	.	.	.	.	.	.	.	.	.	.	.	.	.	.	.	.	.	.	.	.	.	.	.	.	.	.	.
Myrteola nummularia	.	.	.	.	.	.	.	.	.	.	.	.	.	.	.	.	.	.	.	.	.	.	.	.	.	.	.	.	.
Nanodea muscosa	.	.	.	.	.	.	.	.	.	.	.	.	.	.	.	.	.	.	.	.	.	.	.	.	.	.	.	.	.
Neobartsia crenoloba	.	.	.	.	.	.	.	.	.	.	.	.	.	.	.	.	.	.	.	.	.	.	.	.	.	.	.	.	.
Neobartsia pedicularoides	.	.	.	.	.	.	.	.	.	.	.	.	.	.	.	.	.	.	.	.	.	.	.	.	.	.	.	.	.
Neobartsia peruviana	.	.	.	.	.	.	.	.	.	.	.	.	.	.	.	.	.	.	.	.	.	.	.	.	.	.	.	.	.

Bog	262	263	264	265	266	267	268	269	270	271	272	273	274	275	276	277	278	279	280	281	282	283	284	285	286	287	288	289	290
Nertera granadensis	.	.	.	.	.	.	.	.	.	.	.	.	.	.	.	.	.	.	.	.	.	.	.	.	.	.	.	.	.
Nicoraepoa andina	.	.	.	.	.	.	.	.	.	.	.	.	.	.	.	.	.	.	.	.	.	.	.	.	.	.	.	.	.
Nicoraepoa pugionifolia	.	.	.	.	.	.	.	.	.	.	.	.	.	.	.	.	.	.	.	.	.	.	.	.	.	.	.	.	.
Nicoraepoa subenervis	.	.	.	.	.	.	.	.	.	.	.	.	.	.	.	.	.	.	.	.	.	.	.	.	.	.	.	.	.
Nitrophila australis	.	.	.	.	.	.	.	.	.	.	.	.	.	.	.	.	.	.	.	.	.	.	.	.	.	.	.	.	.
Nothofagus antarctica	.	.	.	.	.	.	.	.	.	.	.	.	.	.	.	.	.	.	.	.	.	.	.	.	.	.	.	.	.
Nototriche rugosa	.	.	.	.	.	.	.	.	.	.	.	.	.	.	.	.	.	.	.	.	.	.	.	.	.	.	.	.	.
Ochetophila nana	.	.	.	.	.	.	.	.	.	.	.	.	.	.	.	.	.	.	.	.	.	.	.	.	.	.	.	.	.
Olsynium junceum	.	.	.	.	.	.	.	.	.	.	.	.	.	.	.	.	.	.	.	.	.	.	.	.	.	.	.	.	.
Oreobolus obtusangulus	.	.	.	.	.	.	.	.	.	.	.	.	.	.	.	.	.	.	.	.	.	.	.	.	.	.	.	.	.
Oritrophium limnophilum	.	.	.	.	.	.	.	.	.	.	.	.	.	.	.	.	.	.	.	.	.	.	.	.	.	.	.	.	.
Osmorhiza glabrata	.	.	.	.	.	.	.	.	.	.	.	.	.	.	.	.	.	.	.	.	.	.	.	.	.	.	.	.	.
Ourisia alpina	.	.	.	.	.	.	.	.	.	.	.	.	.	.	.	.	.	.	.	.	.	.	.	.	.	.	.	.	.
Ourisia muscosa	1	.	.	.	.	.	.	.	.	.	1	1	.	.	1	.	.	.	.	.	.	.	.	.	.	1	.	.	1
Ourisia ruelloides	.	.	.	.	.	.	.	.	.	.	.	.	.	.	.	.	.	.	.	.	.	.	.	.	.	.	.	.	.
Oxychloe andina	1	.	.	.	.	.	.	.	.	.	.	.	.	.	.	.	.	.	1	1	1	1	1	1	.	1	1	1	1
Oxychloe bisexualis	.	.	.	.	.	.	.	.	.	.	.	.	.	.	.	.	.	.	.	.	.	.	.	.	.	.	.	.	.
Oxychloe castellanosii	.	.	.	.	.	.	.	.	.	.	.	.	.	.	.	.	.	.	.	.	.	.	.	.	.	.	.	.	.
Oxychloe haumaniana	.	.	.	.	.	.	.	.	.	.	.	.	.	.	.	.	.	.	.	.	.	.	.	.	.	.	.	.	.
Oxychloe mendocina	.	.	.	.	.	.	.	.	.	.	.	.	.	.	.	.	.	.	.	.	.	.	.	.	.	.	.	.	.
Patosia clandestina	.	.	1	1	1	1	1	1	1	1	.	.	.	1	.	.	.	.	.	.	.	.	.	.	1	.	.	.	.
Perezia capito	.	.	.	.	.	.	.	.	.	.	.	.	.	.	.	.	.	.	.	.	.	.	.	.	.	.	.	.	.
Perezia delicata	.	.	.	.	.	.	.	.	.	.	.	.	.	.	.	.	.	.	.	.	.	.	.	.	.	.	.	.	.
Perezia fonkii	.	.	.	.	.	.	.	.	.	.	.	.	.	.	.	.	.	.	.	.	.	.	.	.	.	.	.	.	.
Perezia pedicularidifolia	.	.	.	.	.	.	.	.	.	.	.	.	.	.	.	.	.	.	.	.	.	.	.	.	.	.	.	.	.
Perezia pinnatifida	.	1	.	.	.	.	.	.	.	.	1	.	1	.	.	.	.	.	.	.	.	.	.	1	.	.	.	.	.
Petroravenia friesii	.	1	.	.	.	.	.	.	.	.	.	.	.	.	.	1	1	.	.	1	.	.	1	1	.	1	1	1	1
Petroravenia werdermannii	.	.	.	.	.	.	.	.	.	.	.	.	.	.	.	.	.	.	.	.	.	.	.	.	.	.	.	.	.
Phleum alpinum	.	.	.	.	.	.	.	.	.	.	.	.	.	.	.	.	.	.	.	.	.	.	.	.	.	.	.	.	.
Phylloscirpus acaulis	.	.	.	.	.	.	.	.	.	.	.	.	.	.	.	.	.	.	.	.	.	.	.	.	.	.	.	.	.
Phylloscirpus boliviensis	1	.	.	.	.	.	.	.	.	.	1	1	1	.	.	.	.	.	1	1	1	1	1	1	.	1	1	1	1

Bog	262	263	264	265	266	267	268	269	270	271	272	273	274	275	276	277	278	279	280	281	282	283	284	285	286	287	288	289	290
Phylloscirpus deserticola	1	1	1	1	1	1	1	1	1	1	1	.	.	1	.	1	.	.	1	1	1	1	1	1	1	1	.	.	.
Pinguicula antarctica	.	.	.	.	.	.	.	.	.	.	.	.	.	.	.	.	.	.	.	.	.	.	.	.	.	.	.	.	.
Plantago barbata	.	.	.	.	.	.	.	.	.	.	.	.	.	.	.	.	.	.	.	.	.	.	.	.	.	.	.	.	.
Plantago rigida	.	.	.	.	.	.	.	.	.	.	.	.	.	.	.	.	.	.	.	.	.	.	.	.	.	.	.	.	.
Plantago tubulosa	1	.	1	1	1	1	1	1	.	1	1	1	1	1	1	1	1	1	1	1	1	1	1	1	1	1	1	1	.
Plantago uniglumis	.	.	.	.	.	.	.	.	.	.	.	.	.	.	.	.	.	.	.	.	.	.	.	.	.	.	.	.	.
Poa alopecurus	.	.	.	.	.	.	.	.	.	.	.	.	.	.	.	.	.	.	.	.	.	.	.	.	.	.	.	.	.
Poa hachadoensis	.	.	.	.	.	.	.	.	.	.	.	.	.	.	.	.	.	.	.	.	.	.	.	.	.	.	.	.	.
Poa perligulata	.	.	.	.	.	.	.	.	.	.	1	1	1	.	.	.	1	.	.	.	.	.	1	1	.	1	1	1	1
Polypogon interruptus	.	.	.	.	.	.	.	.	.	.	.	.	.	.	.	.	.	.	.	.	.	.	.	.	.	.	.	.	.
Primula magellanica	.	.	.	.	.	.	.	.	.	.	.	.	.	.	.	.	.	.	.	.	.	.	.	.	.	.	.	.	.
Puccinellia frigida	.	.	.	.	.	.	.	.	.	.	.	.	.	.	.	.	.	.	.	.	.	.	.	.	.	.	.	.	.
Quinchamalium chilense	.	.	.	.	.	.	.	.	.	.	.	.	.	.	.	.	.	.	.	.	.	.	.	.	.	.	.	.	.
Ranunculus breviscapus	.	.	1	.	.	.	.	.	.	.	.	.	.	.	.	.	.	.	.	.	.	.	.	.	.	.	.	.	.
Ranunculus fuegianus	.	.	.	.	.	.	.	.	.	.	.	.	.	.	.	.	.	.	.	.	.	.	.	.	.	.	.	.	.
Ranunculus mandonianus	.	.	.	.	.	.	.	.	.	.	.	.	.	.	.	.	.	.	.	.	.	.	.	.	.	.	.	.	.
Ranunculus peduncularis	.	.	.	.	.	.	.	.	.	.	.	.	.	.	.	.	.	.	.	.	.	.	.	.	.	.	.	.	.
Ranunculus trichophyllus	.	.	.	.	.	.	.	.	.	.	.	.	.	.	.	.	.	.	.	1	.	.	.	.	.	.	.	.	.
Halerpestes uniflora	1	.	.	.	.	.	.	.	.	.	.	.	.	.	.	.	.	.	.	.	.	.	.	.	.	.	.	.	.
Rubus geoides	.	.	.	.	.	.	.	.	.	.	.	.	.	.	.	.	.	.	.	.	.	.	.	.	.	.	.	.	.
Rumex magellanicus	.	.	.	.	.	.	.	.	.	.	.	.	.	.	.	.	.	.	.	.	.	.	.	.	.	.	.	.	.
Rytidosperma lechleri	.	.	.	.	.	.	.	.	.	.	.	.	.	.	.	.	.	.	.	.	.	.	.	.	.	.	.	.	.
Sarcocornia pulvinata	.	.	.	.	.	.	.	.	.	.	.	.	.	.	.	.	.	.	.	.	.	.	.	.	.	.	.	.	.
Schoenoplectus pungens	.	.	.	.	.	.	.	.	.	.	.	.	.	.	.	.	.	.	.	.	.	.	.	.	.	.	.	.	.
Schoenus andinus	.	.	.	.	.	.	.	.	.	.	.	.	.	.	.	.	.	.	.	.	.	.	.	.	.	.	.	.	.
Senecio breviscapus	.	.	.	1	.	.	.	.	.	.	.	.	.	.	.	.	.	.	.	.	.	.	.	.	.	.	.	.	.
Senecio diemii	.	.	.	.	.	.	.	.	.	.	.	.	.	.	.	.	.	.	.	.	.	.	.	.	.	.	.	.	.
Senecio fistulosus	.	.	.	.	.	.	.	.	.	.	.	.	.	.	.	.	.	.	.	.	.	.	.	.	.	.	.	.	.
Senecio parodii	.	.	.	.	.	.	.	.	.	.	.	.	.	.	.	.	.	.	.	.	.	.	.	.	.	.	.	.	.
Senecio peteroanus	.	.	.	.	.	.	.	.	.	.	.	.	.	.	.	.	.	.	.	.	.	.	.	.	.	.	.	.	.
Senecio serratifolius	.	.	.	.	.	.	.	.	.	.	.	1	.	.	1	.	.	.	.	.	.	.	.	.	.	.	.	.	.

Bog	262	263	264	265	266	267	268	269	270	271	272	273	274	275	276	277	278	279	280	281	282	283	284	285	286	287	288	289	290
Senecio trifurcatus	.	.	.	.	.	.	.	.	.	.	.	.	.	.	.	.	.	.	.	.	.	.	.	.	.	.	.	.	.
Sisyrinchium chilense	.	.	.	.	.	.	.	.	.	.	.	.	.	.	.	.	.	.	.	.	.	.	.	.	.	.	.	.	.
Sisyrinchium patagonicum	.	.	.	.	.	.	.	.	.	.	.	.	.	.	.	.	.	.	.	.	.	.	.	.	.	.	.	.	.
Sisyrinchium pearcei	.	.	.	.	.	.	.	.	.	.	.	.	.	.	.	.	.	.	.	.	.	.	.	.	.	.	.	.	.
Stellaria debilis	.	.	.	.	.	.	.	.	.	.	.	.	.	.	.	.	.	.	.	.	.	.	.	.	.	.	.	.	.
Stuckenia filiformis	.	.	.	.	.	.	.	.	.	.	.	.	.	.	.	.	.	1	.	.	1	.	.	1	.	.	.	.	.
Stuckenia striata	.	.	.	.	.	.	.	.	.	.	.	.	.	.	.	.	.	.	.	.	.	.	.	.	.	.	.	.	.
Symphyotrichum peteroanum	.	.	.	.	.	.	.	.	.	.	.	.	.	.	.	.	.	.	.	.	.	.	.	.	.	.	.	.	.
Symphyotrichum vahlii	.	.	.	.	.	.	.	.	.	.	.	.	.	.	.	.	.	.	.	.	.	.	.	.	.	.	.	.	.
Tetroncium magellanicum	.	.	.	.	.	.	.	.	.	.	.	.	.	.	.	.	.	.	.	.	.	.	.	.	.	.	.	.	.
Tribeles australis	.	.	.	.	.	.	.	.	.	.	.	.	.	.	.	.	.	.	.	.	.	.	.	.	.	.	.	.	.
Trifolium amabile	.	.	.	.	.	.	.	.	.	.	.	.	.	.	.	.	.	.	.	.	.	.	.	.	.	.	.	.	.
Trifolium polymorphum	.	.	.	.	.	.	.	.	.	.	.	.	.	.	.	.	.	.	.	.	.	.	.	.	.	.	.	.	.
Triglochin concinna	.	.	.	.	.	.	.	.	.	.	.	.	.	.	.	.	.	.	.	.	.	.	.	.	.	.	.	.	.
Triglochin palustris	.	.	.	.	.	.	.	.	.	.	.	.	.	.	.	.	.	.	.	.	.	.	.	.	.	.	.	.	.
Triglochin striata	.	.	.	.	.	.	.	.	.	.	.	.	.	.	.	.	.	.	.	.	.	.	.	.	.	.	.	.	.
Trisetum caudulatum	.	.	.	.	.	.	.	.	.	.	.	.	.	.	.	.	.	.	.	.	.	.	.	.	.	.	.	.	.
Trisetum preslei	.	.	.	.	.	.	.	.	.	.	.	.	.	.	.	.	.	.	.	.	.	.	.	.	.	.	.	.	.
Koeleria spicata	.	.	.	.	.	.	.	.	.	.	.	.	.	.	.	.	.	.	.	.	.	.	.	.	.	.	.	.	.
Utricularia gibba	.	.	.	.	.	.	.	.	.	.	.	.	.	.	.	.	.	.	.	.	.	.	.	.	.	.	.	.	.
Vahlodea atropurpurea	.	.	.	.	.	.	.	.	.	.	.	.	.	.	.	.	.	.	.	.	.	.	.	.	.	.	.	.	.
Valeriana fonckii	.	.	.	.	.	.	.	.	.	.	.	.	.	.	.	.	.	.	.	.	.	.	.	.	.	.	.	.	.
Valeriana macrorhiza	.	.	.	.	.	.	.	.	.	.	.	.	.	.	.	.	.	.	.	.	.	.	.	.	.	.	.	.	.
Viola pygmaea	.	.	.	.	.	.	.	.	.	.	.	.	.	.	.	.	.	.	.	.	.	.	.	.	.	.	.	.	.
Werneria apiculata	.	.	.	.	.	.	.	.	.	.	.	.	.	.	.	.	.	.	.	.	.	.	.	.	.	.	.	.	.
Werneria pinnatifida	.	.	.	1	.	.	.	1	1	.	1	1	1	1	.	1	1	1	1	1	.	.	1	.	1	1	1	.	.
Werneria pygmaea	1	1	1	1	1	1	1	1	1	1	1	1	1	1	1	1	1	1	1	1	1	1	1	1	1	1	1	1	1
Werneria solivifolia	.	.	.	1	.	1	.	.	.	.	.	.	.	.	.	1	.	.	1	.	.	.	.	1	.	1	.	1	.
Werneria spathulata	1	.	.	.	.	.	.	.	.	.	.	.	.	.	.	.	.	.	.	.	.	.	.	.	.	.	.	1	1
Xenophyllum incisum	.	.	.	.	.	.	.	.	.	.	.	.	.	.	.	.	.	.	.	.	.	.	.	.	.	.	.	.	.
Zameioscirpus atacamensis	.	.	.	.	.	.	.	.	.	.	.	.	.	.	.	.	.	.	.	.	.	.	.	.	.	.	.	.	.
Zameioscirpus gaimardiodes	.	.	.	.	.	.	.	.	.	.	.	.	.	.	.	.	.	.	.	.	.	.	.	.	.	.	.	.	.
Zameioscirpus muticus	1	1	.	1	1	1	1	.	.	1	1	1	1	.	1	1	1	1	1	1	1	1	.	1	.	1	1	1	1

Bog	291	292	293	294	295	296	297	298	299	300	301	302	303	304	305	306	307	308	309	310	311	312	313	314	315	316	317	318	319
**Operational zone**	N	N	N	N	N	N	N	N	N	N	N	N	N	N	N	N	N	N	N	N	N	N	N	N	N	N	N	N	N
**Cluster**	1	1	1	1	1	1	1	1	1	1	1	1	1	1	1	1	1	1	1	1	1	1	1	1	1	1	1	1	1
**Bioregion**	N	N	N	N	N	N	N	N	N	N	N	N	N	N	N	N	N	N	N	N	N	N	N	N	N	N	N	N	N
**Longitude**	-66.61	-67.1	-66.59	-66.45	-67.13	-67.11	-66.28	-67.18	-67.24	-67.4	-67.31	-67.22	-67.75	-67.75	-67.73	-67.35	-67.4	-65.69	-67.38	-67.12	-67.16	-67.72	-67.19	-67.16	-67.65	-67.86	-67.65	-67.38	-66.43
**Latitude**	-21.77	-22.04	-21.78	-21.61	-22.07	-22.65	-19.21	-22.08	-22.32	-22.77	-22.24	-22.57	-22.65	-22.65	-22.64	-22.44	-22.8	-19.65	-22.34	-22.64	-22.63	-22.18	-22.71	-22.08	-21.29	-21.52	-21.29	-22.32	-19.27
Acaena antarctica	.	.	.	.	.	.	.	.	.	.	.	.	.	.	.	.	.	.	.	.	.	.	.	.	.	.	.	.	.
Acaena macrocephala	.	.	.	.	.	.	.	.	.	.	.	.	.	.	.	.	.	.	.	.	.	.	.	.	.	.	.	.	.
Acaena magellanica	.	.	.	.	.	.	.	.	.	.	.	.	.	.	.	.	.	.	.	.	.	.	.	.	.	.	.	.	.
Acaena ovalifolia	.	.	.	.	.	.	.	.	.	.	.	.	.	.	.	.	.	.	.	.	.	.	.	.	.	.	.	.	.
Acaena pinnatifida	.	.	.	.	.	.	.	.	.	.	.	.	.	.	.	.	.	.	.	.	.	.	.	.	.	.	.	.	.
Adesmia retusa	.	.	.	.	.	.	.	.	.	.	.	.	.	.	.	.	.	.	.	.	.	.	.	.	.	.	.	.	.
Agrostis breviculmis	.	.	.	.	.	.	.	.	.	.	.	.	.	.	.	.	.	.	.	.	.	.	.	.	.	.	.	.	.
Agrostis imberbis	.	.	.	.	.	.	.	.	.	.	.	.	.	.	.	.	.	.	.	.	.	.	.	.	.	.	.	.	.
Agrostis meyenii	.	.	.	.	.	.	.	.	.	.	.	.	.	.	.	.	.	.	.	.	.	.	.	.	.	.	.	.	.
Agrostis perennans	.	.	.	.	.	.	.	.	.	.	.	.	.	.	.	.	.	.	.	.	.	.	.	.	.	.	.	.	.
Alchemilla pinnata	.	.	.	.	.	.	.	.	.	.	.	.	.	.	.	.	.	.	.	.	.	.	.	.	.	.	.	.	.
Alopecurus magellanicus	.	.	.	.	.	.	.	.	.	.	.	.	.	.	.	.	.	.	.	.	.	.	.	.	.	.	.	.	.
Amphiscirpus nevadensis	.	.	.	.	.	.	.	.	.	.	.	.	.	.	.	.	.	.	.	.	.	.	.	.	.	.	.	.	.
Anagallis alternifolia	.	.	.	.	.	.	.	.	.	.	.	.	.	.	.	.	.	.	.	.	.	.	.	.	.	.	.	.	.
Antennaria chilensis	.	.	.	.	.	.	.	.	.	.	.	.	.	.	.	.	.	.	.	.	.	.	.	.	.	.	.	.	.
Anthoxanthum redolens	.	.	.	.	.	.	.	.	.	.	.	.	.	.	.	.	.	.	.	.	.	.	.	.	.	.	.	.	.
Apium panul	.	.	.	.	.	.	.	.	.	.	.	.	.	.	.	.	.	.	.	.	.	.	.	.	.	.	.	.	.
Arenaria rivularis	.	.	.	.	.	.	.	.	.	.	.	.	.	.	.	.	.	.	.	.	.	.	.	.	.	.	.	.	.
Arenaria serpens	.	.	.	.	.	.	.	.	.	.	.	.	.	.	.	.	.	.	.	.	.	.	.	.	.	.	.	.	.
Arjona pusilla	.	.	.	.	.	.	.	.	.	.	.	.	.	.	.	.	.	1	.	.	.	.	.	.	.	.	.	.	.
Astragalus bustillosii	.	.	.	.	.	.	.	.	.	.	.	.	.	.	.	.	.	.	.	.	.	.	.	.	.	.	.	.	.
Astragalus micranthellus	.	.	.	.	.	.	.	.	.	.	.	.	.	.	.	.	.	.	.	.	.	.	.	.	.	.	.	.	.
Azolla filiculoides	.	.	.	.	.	.	.	.	.	.	.	.	.	.	.	.	.	.	.	.	.	.	.	.	1	1	.	.	.
Azorella boelckei	.	.	.	.	.	.	.	.	.	.	.	.	.	.	.	.	.	.	.	.	.	.	.	.	.	.	.	.	.

**Bog**	**291**	**292**	**293**	**294**	**295**	**296**	**297**	**298**	**299**	**300**	**301**	**302**	**303**	**304**	**305**	**306**	**307**	**308**	**309**	**310**	**311**	**312**	**313**	**314**	**315**	**316**	**317**	**318**	**319**
Azorella burkartii	.	.	.	.	.	.	.	.	.	.	.	.	.	.	.	.	.	.	.	.	.	.	.	.	.	.	.	.	.
Azorella cryptantha	.	.	.	.	.	.	.	.	.	.	.	.	.	.	.	.	.	.	.	.	.	.	.	.	.	.	.	.	.
Azorella lycopodioides	.	.	.	.	.	.	.	.	.	.	.	.	.	.	.	.	.	.	.	.	.	.	.	.	.	.	.	.	.
Azorella trifoliolata	.	.	.	.	.	.	.	.	.	.	.	.	.	.	.	.	.	.	.	.	.	.	.	.	.	.	.	.	.
Baccharis acaulis	.	.	.	.	.	.	.	.	.	.	.	.	.	.	.	.	.	.	.	.	.	.	.	.	.	.	.	.	.
Baccharis caespitosa	.	.	.	.	.	.	.	.	.	.	.	.	.	.	.	.	.	.	.	.	.	.	.	.	.	.	.	.	.
Baccharis magellanica	.	.	.	.	.	.	.	.	.	.	.	.	.	.	.	.	.	.	.	.	.	.	.	.	.	.	.	.	.
Belloa chilensis	.	.	.	.	.	.	.	.	.	.	.	.	.	.	.	.	.	.	.	.	.	.	.	.	.	.	.	.	.
Bromus catharticus	.	.	.	.	.	.	.	.	.	.	.	.	.	.	.	.	.	.	.	.	.	.	.	.	.	.	.	.	.
Calandrinia acaulis	.	.	.	.	.	.	.	.	.	.	.	.	.	.	.	.	.	.	.	.	.	.	.	.	.	.	.	.	.
Calandrinia compacta	1	1	.	1	1	1	.	1	1	1	.	1	1	.	1	1	.	.	.	.	1	1	.	.	1	.	.	.	.
Calceolaria biflora	.	.	.	.	.	.	.	.	.	.	.	.	.	.	.	.	.	.	.	.	.	.	.	.	.	.	.	.	.
Calceolaria cana	.	.	.	.	.	.	.	.	.	.	.	.	.	.	.	.	.	.	.	.	.	.	.	.	.	.	.	.	.
Calceolaria corymbosa	.	.	.	.	.	.	.	.	.	.	.	.	.	.	.	.	.	.	.	.	.	.	.	.	.	.	.	.	.
Calceolaria filicaulis	.	.	.	.	.	.	.	.	.	.	.	.	.	.	.	.	.	.	.	.	.	.	.	.	.	.	.	.	.
Callitriche lechleri	.	.	.	.	.	.	.	.	.	.	.	.	.	.	.	.	.	.	.	.	.	.	.	.	.	.	.	.	.
Caltha appendiculata	.	.	.	.	.	.	.	.	.	.	.	.	.	.	.	.	.	.	.	.	.	.	.	.	.	.	.	.	.
Caltha sagittata	.	.	.	.	.	.	.	.	.	.	.	.	.	.	.	.	.	.	.	.	.	.	.	.	.	.	.	.	.
Cardamine cordata	.	.	.	.	.	.	.	.	.	.	.	.	.	.	.	.	.	.	.	.	.	.	.	.	.	.	.	.	.
Cardamine glacialis	.	.	.	.	.	.	.	.	.	.	.	.	.	.	.	.	.	.	.	.	.	.	.	.	.	.	.	.	.
Cardamine tenuirostris	.	.	.	.	.	.	.	.	.	.	.	.	.	.	.	.	.	.	.	.	.	.	.	.	.	.	.	.	.
Cardamine volckmannii	.	.	.	.	.	.	.	.	.	.	.	.	.	.	.	.	.	.	.	.	.	.	.	.	.	.	.	.	.
Carex acaulis	.	.	.	.	.	.	.	.	.	.	.	.	.	.	.	.	.	.	.	.	.	.	.	.	.	.	.	.	.
Carex atropicta	.	.	.	.	.	.	.	.	.	.	.	.	.	.	.	.	.	.	.	.	.	.	.	.	.	.	.	.	.
Carex banksii	.	.	.	.	.	.	.	.	.	.	.	.	.	.	.	.	.	.	.	.	.	.	.	.	.	.	.	.	.
Carex caduca	.	.	.	.	.	.	.	.	.	.	.	.	.	.	.	.	.	.	.	.	.	.	.	.	.	.	.	.	.
Carex decidua	.	.	.	.	.	.	.	.	.	.	.	.	.	.	.	.	.	.	.	.	.	.	.	.	.	.	.	.	.
Carex fuscula	.	.	.	.	.	.	.	.	.	.	.	.	.	.	.	.	.	.	.	.	.	.	.	.	.	.	.	.	.
Carex gayana	.	.	.	.	.	.	.	.	.	.	.	.	.	.	.	.	.	.	.	.	.	.	.	.	.	.	.	.	.
Carex hypoleucos	.	.	.	.	.	.	.	.	.	.	.	.	.	.	.	.	.	.	.	.	.	.	.	.	.	.	.	.	.
Carex macloviana	.	.	.	.	.	.	.	.	.	.	.	.	.	.	.	.	.	.	.	.	.	.	.	.	.	.	.	.	.
Carex magellanica	.	.	.	.	.	.	.	.	.	.	.	.	.	.	.	.	.	.	.	.	.	.	.	.	.	.	.	.	.
Carex malmei	.	.	.	.	.	.	.	.	.	.	.	.	.	.	.	.	.	.	.	.	.	.	.	.	.	.	.	.	.
Carex maritima	1	1	1	1	1	1	.	1	1	1	1	1	1	1	1	1	1	1	.	1	1	1	1	1	1	1	.	1	.
Carex microglochin	.	.	.	.	.	.	.	.	.	.	.	.	.	.	.	.	.	.	.	.	.	.	.	.	.	.	.	.	.
Carex pleioneura	.	.	.	.	.	.	.	.	.	.	.	.	.	.	.	.	.	.	.	.	.	.	.	.	.	.	.	.	.
Carex ruthsatzae	.	.	.	.	.	.	.	.	.	.	.	.	.	.	.	.	.	.	.	.	.	.	.	.	.	.	.	.	.
Carex vallis-pulchrae	.	.	.	.	.	.	.	.	.	.	.	.	.	.	.	.	.	.	.	.	.	.	.	.	.	.	.	.	.

**Bog**	**291**	**292**	**293**	**294**	**295**	**296**	**297**	**298**	**299**	**300**	**301**	**302**	**303**	**304**	**305**	**306**	**307**	**308**	**309**	**310**	**311**	**312**	**313**	**314**	**315**	**316**	**317**	**318**	**319**
Carpha schoenoides	.	.	.	.	.	.	.	.	.	.	.	.	.	.	.	.	.	.	.	.	.	.	.	.	.	.	.	.	.
Castilleja pumila	.	.	.	.	.	.	.	.	.	.	.	.	.	.	.	.	.	1	.	.	.	.	.	.	.	.	.	.	1
Catabrosa werdermannii	.	.	.	.	.	.	.	.	.	.	.	.	.	.	.	.	.	.	.	.	.	.	.	.	.	.	.	.	.
Cerastium humifusum	.	.	.	.	.	.	.	.	.	.	.	.	.	.	.	.	.	.	.	.	.	.	.	.	.	.	.	.	.
Cerastium montioides	.	.	.	.	.	.	.	.	.	.	.	.	.	.	.	.	.	.	.	.	.	.	.	.	.	.	.	.	.
Chiliotrichum diffusum	.	.	.	.	.	.	.	.	.	.	.	.	.	.	.	.	.	.	.	.	.	.	.	.	.	.	.	.	.
Chusquea culeou	.	.	.	.	.	.	.	.	.	.	.	.	.	.	.	.	.	.	.	.	.	.	.	.	.	.	.	.	.
Colobanthus quitensis	.	.	1	1	.	.	.	.	.	.	1	.	.	.	.	.	.	1	.	.	.	.	.	.	1	.	1	1	.
Cortaderia egmontiana	.	.	.	.	.	.	.	.	.	.	.	.	.	.	.	.	.	.	.	.	.	.	.	.	.	.	.	.	.
Cotula mexicana	.	.	.	.	.	.	1	.	.	.	1	.	.	.	.	.	.	1	.	.	.	.	.	.	1	1	.	1	1
Crassula peduncularis	.	.	.	.	.	.	.	.	.	.	.	.	.	.	.	.	.	.	.	.	.	.	.	.	.	.	.	.	.
Cuatrecasasiella argentina	.	.	.	.	.	.	.	.	.	.	.	.	.	.	.	.	.	1	.	.	.	.	.	.	.	.	.	.	1
Deschampsia antarctica	.	.	.	.	.	.	.	.	.	.	.	.	.	.	.	.	.	.	.	.	.	.	.	.	.	.	.	.	.
Deschampsia caespitosa	.	.	.	.	.	.	.	.	.	.	.	.	.	.	.	.	.	.	.	.	.	.	.	.	.	.	.	.	.
Deschampsia patula	.	.	.	.	.	.	.	.	.	.	.	.	.	.	.	.	.	.	.	.	.	.	.	.	.	.	.	.	.
Cinnagrostis brevifolia	.	.	.	.	.	.	.	.	.	.	.	.	.	.	.	.	.	.	.	.	.	.	.	.	.	.	.	.	.
Deschampsia chrysantha	1	1	.	1	1	1	.	1	1	1	.	1	1	1	1	1	.	.	.	1	1	1	1	1	.	1	.	.	.
Cinnagrostis chrysophylla	.	.	.	.	.	.	.	.	.	.	.	.	.	.	.	.	.	.	.	.	.	.	.	.	.	.	.	.	.
Deschampsia chrysostachya	.	.	.	.	.	.	.	.	.	.	.	.	.	.	.	.	.	.	.	.	.	.	.	.	.	.	.	.	.
Deschampsia eminens	.	.	.	.	.	.	.	.	.	.	1	.	.	.	.	.	.	.	1	.	.	.	.	.	.	.	1	.	.
Deschampsia hackelii	.	.	.	.	.	.	.	.	.	.	.	.	.	.	.	.	.	.	.	.	.	.	.	.	.	.	.	.	.
Cinnagrostis minima	.	.	.	.	.	.	.	.	.	.	.	.	.	.	.	.	.	.	.	.	.	.	.	.	.	.	.	.	.
Deschampsia ovata	.	.	.	.	.	.	.	.	.	.	.	.	.	.	.	.	.	.	.	.	.	.	.	.	.	.	.	.	.
Cinnagrostis rigescens	.	.	.	.	.	.	1	.	.	.	.	.	.	.	.	.	.	1	.	.	.	.	.	.	1	.	1	.	1
Cinnagrostis spicigera	.	.	.	.	.	.	.	.	.	.	.	.	.	.	.	.	.	.	.	.	.	.	.	.	.	.	.	.	.
Cinnagrostis velutina	.	.	.	.	.	.	.	.	.	.	.	.	.	.	.	.	.	.	.	.	.	.	.	.	.	.	.	.	.
Cinnagrostis vicunarum	.	.	.	.	.	.	.	.	.	.	.	.	.	.	.	.	.	.	.	.	.	.	.	.	.	.	.	.	.
Distichia filamentosa	.	.	.	.	.	.	.	.	.	.	.	.	.	.	.	.	.	.	.	.	.	.	.	.	.	.	.	.	.
Distichia muscoides	1	1	1	1	1	1	.	1	1	1	1	1	1	1	1	1	1	1	1	1	1	1	1	1	.	.	.	.	1
Distichlis humilis	.	.	.	.	.	.	.	.	.	.	.	.	.	.	.	.	.	.	.	.	.	.	.	.	1	.	.	.	.
Distichlis scoparia	.	.	.	.	.	.	.	.	.	.	.	.	.	.	.	.	.	.	.	.	.	.	.	.	.	.	.	.	.
Distichlis spicata	.	.	.	.	.	.	.	.	.	.	.	.	.	.	.	.	.	.	.	.	.	.	.	.	.	.	.	.	.

Bog	291	292	293	294	295	296	297	298	299	300	301	302	303	304	305	306	307	308	309	310	311	312	313	314	315	316	317	318	319
Draba pusilla	.	.	.	.	.	.	.	.	.	.	.	.	.	.	.	.	.	.	.	.	.	.	.	.	.	.	.	.	.
Eleocharis melanomphala	.	.	.	.	.	.	.	.	.	.	.	.	.	.	.	.	.	.	.	.	.	.	.	.	.	.	.	.	.
Eleocharis pseudoalbibracteata	.	.	.	.	.	.	.	.	.	.	.	.	.	.	.	.	.	.	.	.	.	.	.	.	.	.	.	.	.
Elodea potamogeton	.	.	.	.	.	.	.	.	.	.	.	.	.	.	.	.	.	.	.	.	.	.	.	.	.	.	.	.	.
Empetrum rubrum	.	.	.	.	.	.	.	.	.	.	.	.	.	.	.	.	.	.	.	.	.	.	.	.	.	.	.	.	.
Epilobium australe	.	.	.	.	.	.	.	.	.	.	.	.	.	.	.	.	.	.	.	.	.	.	.	.	.	.	.	.	.
Epilobium barbeyanum	.	.	.	.	.	.	.	.	.	.	.	.	.	.	.	.	.	.	.	.	.	.	.	.	.	.	.	.	.
Epilobium ciliatum	.	.	.	.	.	.	.	.	.	.	.	.	.	.	.	.	.	.	.	.	.	.	.	.	.	.	.	.	.
Epilobium denticulatum	.	.	.	.	.	.	.	.	.	.	.	.	.	.	.	.	.	.	.	.	.	.	.	.	.	.	.	.	.
Epilobium fragile	.	.	.	.	.	.	.	.	.	.	.	.	.	.	.	.	.	.	.	.	.	.	.	.	.	.	.	.	.
Epilobium glaucum	.	.	.	.	.	.	.	.	.	.	.	.	.	.	.	.	.	.	.	.	.	.	.	.	.	.	.	.	.
Epilobium nivale	.	.	.	.	.	.	.	.	.	.	.	.	.	.	.	.	.	.	.	.	.	.	.	.	.	.	.	.	.
Erigeron andicola	.	.	.	.	.	.	.	.	.	.	.	.	.	.	.	.	.	.	.	.	.	.	.	.	.	.	.	.	.
Erigeron leptopetalus	.	.	.	.	.	.	.	.	.	.	.	.	.	.	.	.	.	.	.	.	.	.	.	.	.	.	.	.	.
Erigeron myosotis	.	.	.	.	.	.	.	.	.	.	.	.	.	.	.	.	.	.	.	.	.	.	.	.	.	.	.	.	.
Erigeron patagonicus	.	.	.	.	.	.	.	.	.	.	.	.	.	.	.	.	.	.	.	.	.	.	.	.	.	.	.	.	.
Erythranthe cuprea	.	.	.	.	.	.	.	.	.	.	.	.	.	.	.	.	.	.	.	.	.	.	.	.	.	.	.	.	.
Erythranthe depressa	.	.	.	.	.	.	.	.	.	.	.	.	.	.	.	.	.	.	.	.	.	.	.	.	.	.	.	.	.
Erythranthe glabrata	.	.	.	.	.	.	.	.	.	.	.	.	.	.	.	.	.	.	.	.	.	.	.	.	1	.	.	.	1
Erythranthe lutea	.	.	.	.	.	.	.	.	.	.	.	.	.	.	.	.	.	.	.	.	.	.	.	.	.	.	.	.	.
Escallonia virgata	.	.	.	.	.	.	.	.	.	.	.	.	.	.	.	.	.	.	.	.	.	.	.	.	.	.	.	.	.
Euphrasia antarctica	.	.	.	.	.	.	.	.	.	.	.	.	.	.	.	.	.	.	.	.	.	.	.	.	.	.	.	.	.
Euphrasia chrysantha	.	.	.	.	.	.	.	.	.	.	.	.	.	.	.	.	.	.	.	.	.	.	.	.	.	.	.	.	.
Euphrasia subexserta	.	.	.	.	.	.	.	.	.	.	.	.	.	.	.	.	.	.	.	.	.	.	.	.	.	.	.	.	.
Festuca hypsophila	.	.	.	.	.	.	.	.	.	.	.	.	.	.	.	.	.	.	.	.	.	.	.	.	.	.	.	.	.
Festuca kurtziana	.	.	.	.	.	.	.	.	.	.	.	.	.	.	.	.	.	.	.	.	.	.	.	.	.	.	.	.	.
Festuca lilloi	.	.	.	.	.	.	.	.	.	.	.	.	.	.	.	.	.	.	.	.	.	.	.	.	.	.	.	.	.
Festuca magellanica	.	.	.	.	.	.	.	.	.	.	.	.	.	.	.	.	.	.	.	.	.	.	.	.	.	.	.	.	.
Festuca nardifolia	.	.	.	.	.	.	.	.	.	.	.	.	.	.	.	.	.	.	.	.	.	.	.	.	.	.	.	.	.
Festuca rigescens	.	.	.	.	.	.	.	.	.	.	.	.	.	.	.	.	.	.	.	.	.	.	.	.	.	.	.	.	.
Festuca werdermannii	.	.	.	.	.	.	.	.	.	.	.	.	.	.	.	.	.	.	.	.	.	.	.	.	.	.	.	.	.
Frankenia triandra	.	.	.	.	.	.	.	.	.	.	.	.	.	.	.	.	.	.	.	.	.	.	.	.	.	.	.	.	.
Gamocarpha graminea	.	.	.	.	.	.	.	.	.	.	.	.	.	.	.	.	.	.	.	.	.	.	.	.	.	.	.	.	.
Gamocarpha ventosa	.	.	.	.	.	.	.	.	.	.	.	.	.	.	.	.	.	.	.	.	.	.	.	.	.	.	.	.	.
Gamochaeta chamissonis	.	.	.	.	.	.	.	.	.	.	.	.	.	.	.	.	.	.	.	.	.	.	.	.	.	.	.	.	.

Bog	291	292	293	294	295	296	297	298	299	300	301	302	303	304	305	306	307	308	309	310	311	312	313	314	315	316	317	318	319
Gamochaeta longipedicellata	.	.	.	.	.	.	.	.	.	.	.	.	.	.	.	.	.	.	.	.	.	.	.	.	.	.	.	.	.
Gamochaeta neuquensis	.	.	.	.	.	.	.	.	.	.	.	.	.	.	.	.	.	.	.	.	.	.	.	.	.	.	.	.	.
Gaultheria antarctica	.	.	.	.	.	.	.	.	.	.	.	.	.	.	.	.	.	.	.	.	.	.	.	.	.	.	.	.	.
Gaultheria caespitosa	.	.	.	.	.	.	.	.	.	.	.	.	.	.	.	.	.	.	.	.	.	.	.	.	.	.	.	.	.
Gaultheria pumila	.	.	.	.	.	.	.	.	.	.	.	.	.	.	.	.	.	.	.	.	.	.	.	.	.	.	.	.	.
Gavilea chica	.	.	.	.	.	.	.	.	.	.	.	.	.	.	.	.	.	.	.	.	.	.	.	.	.	.	.	.	.
Gentiana prostrata	1	1	1	.	1	1	1	1	.	1	1	.	1	1	.	.	.	1	1	.	1	1	.	.	1	1	.	1	1
Gentianella fiebrigii	.	.	.	.	.	.	.	.	.	.	.	.	.	.	.	.	.	.	.	.	.	.	.	.	.	.	.	.	.
Gentianella magellanica	.	.	.	.	.	.	.	.	.	.	.	.	.	.	.	.	.	.	.	.	.	.	.	.	.	.	.	.	.
Gentianella multicaulis	.	.	.	.	.	.	.	.	.	.	.	.	.	.	.	.	.	.	.	.	.	.	.	.	.	.	.	.	.
Gentianella ottonis	.	.	.	.	.	.	.	.	.	.	.	.	.	.	.	.	.	.	.	.	.	.	.	.	.	.	.	.	.
Gentianella primuloides	.	.	.	.	.	.	.	.	.	.	.	.	.	.	.	.	.	.	.	.	.	.	.	.	.	.	.	.	.
Gentianella pseudocrassula	.	.	.	.	.	.	.	.	.	.	.	.	.	.	.	.	.	.	.	.	.	.	.	.	.	.	.	.	.
Geranium sessiliflorum	.	.	.	.	.	.	.	.	.	.	.	.	.	.	.	.	.	.	.	.	.	.	.	.	.	.	.	.	.
Gunnera magellanica	.	.	.	.	.	.	.	.	.	.	.	.	.	.	.	.	.	.	.	.	.	.	.	.	.	.	.	.	.
Halenia caespitosa	.	.	.	.	.	.	.	.	.	.	.	.	.	.	.	.	.	.	.	.	.	.	.	.	.	.	.	.	.
Halerpestes cymbalaria	.	.	.	.	.	.	.	.	.	.	.	.	.	.	.	.	.	.	.	.	.	.	.	.	.	.	1	.	.
Halerpestes exilis	1	1	1	1	.	1	.	1	.	.	1	1	1	.	1	.	.	.	.	.	1	1	1	.	.	1	.	.	.
Hieracium antarcticum	.	.	.	.	.	.	.	.	.	.	.	.	.	.	.	.	.	.	.	.	.	.	.	.	.	.	.	.	.
Hordeum comosum	.	.	.	.	.	.	.	.	.	.	.	.	.	.	.	.	.	.	.	.	.	.	.	.	.	.	.	.	.
Hordeum muticum	.	.	.	.	.	.	.	.	.	.	.	.	.	.	.	.	.	.	.	.	.	.	.	.	.	.	.	.	.
Hypochaeris acaulis	.	.	.	.	.	.	.	.	.	.	.	.	.	.	.	.	.	.	.	.	.	.	.	.	.	.	.	.	.
Hypochaeris chondrilloides	.	.	.	.	.	.	.	.	.	.	.	.	.	.	.	.	.	.	.	.	.	.	.	.	.	.	.	.	.
Hypochaeris meyeniana	.	.	.	.	.	.	.	.	.	.	.	.	.	.	.	.	.	.	.	.	.	.	.	.	.	.	.	.	.
Hypochaeris palustris	.	.	.	.	.	.	.	.	.	.	.	.	.	.	.	.	.	.	.	.	.	.	.	.	.	.	.	.	.
Hypochaeris taraxacoides	1	.	.	.	.	.	1	.	1	.	.	.	.	1	.	.	.	1	.	.	1	.	.	.	1	.	1	1	1
Hypochaeris tenerifolia	.	.	.	.	.	.	.	.	.	.	.	.	.	.	.	.	.	.	.	.	.	.	.	.	.	.	.	.	.
Isolepis nigricans	.	.	.	.	.	.	.	.	.	.	.	.	.	.	.	.	.	.	.	.	.	.	.	.	.	.	.	.	.
Isolepsis inundata	.	.	.	.	.	.	.	.	.	.	.	.	.	.	.	.	.	.	.	.	.	.	.	.	.	.	.	.	.
Juncus balticus	.	.	.	.	.	.	.	.	.	.	.	.	.	.	.	.	.	.	.	.	.	.	.	.	.	.	.	.	.
Juncus stipulatus	.	.	.	.	.	.	1	.	.	.	1	.	.	.	.	.	.	1	1	.	.	.	.	.	1	.	.	1	1
Koeleria kurtzii	.	.	.	.	.	.	.	.	.	.	.	.	.	.	.	.	.	.	.	.	.	.	.	.	.	.	.	.	.

**Bog**	**291**	**292**	**293**	**294**	**295**	**296**	**297**	**298**	**299**	**300**	**301**	**302**	**303**	**304**	**305**	**306**	**307**	**308**	**309**	**310**	**311**	**312**	**313**	**314**	**315**	**316**	**317**	**318**	**319**
Lachemilla diplophylla	.	.	.	.	.	.	1	.	.	.	.	.	.	.	.	.	.	1	.	.	.	.	.	.	1	.	.	.	1
Lachemilla pinnata	1	1	.	.	.	.	1	.	.	.	.	.	.	.	.	.	.	1	.	.	.	.	.	.	.	.	.	.	1
Lagenophora nudicaulis	.	.	.	.	.	.	.	.	.	.	.	.	.	.	.	.	.	.	.	.	.	.	.	.	.	.	.	.	.
Lemna minuta	.	.	.	.	.	1	.	.	.	.	.	.	.	1	1	.	.	.	.	.	.	.	.	.	.	1	.	1	.
Leptinella scariosa	.	.	.	.	.	.	.	.	.	.	.	.	.	.	.	.	.	.	.	.	.	.	.	.	.	.	.	.	.
Leucheria candidissima	.	.	.	.	.	.	.	.	.	.	.	.	.	.	.	.	.	.	.	.	.	.	.	.	.	.	.	.	.
Leucheria nutans	.	.	.	.	.	.	.	.	.	.	.	.	.	.	.	.	.	.	.	.	.	.	.	.	.	.	.	.	.
Lilaea scilloides	.	.	.	.	.	.	.	.	.	.	.	.	.	.	.	.	.	.	.	.	.	.	.	.	1	.	.	.	.
Lilaeopsis macloviana	.	1	.	1	.	1	1	.	.	.	1	1	1	1	.	.	.	.	1	.	1	1	.	1	1	1	.	1	1
Limosella australis	.	.	.	.	.	.	.	.	.	.	.	.	.	.	.	.	.	.	.	.	.	.	.	.	.	.	.	.	.
Lobelia oligophylla	1	1	1	1	1	1	1	1	1	.	1	1	1	1	1	.	.	1	1	1	1	1	1	1	1	1	1	1	1
Luzula brachyphylla	.	.	.	.	.	.	.	.	.	.	.	.	.	.	.	.	.	.	.	.	.	.	.	.	.	.	.	.	.
Luzula chilensis	.	.	.	.	.	.	.	.	.	.	.	.	.	.	.	.	.	.	.	.	.	.	.	.	.	.	.	.	.
Luzula racemosa	.	.	.	.	.	.	.	.	.	.	.	.	.	.	.	.	.	.	.	.	.	.	.	.	.	.	.	.	.
Luzula vulcanica	.	.	.	.	.	.	.	.	.	.	.	.	.	.	.	.	.	.	.	.	.	.	.	.	.	.	.	.	.
Lysipomia pumila	.	.	.	.	.	.	.	.	.	.	.	.	.	.	.	.	.	.	.	.	.	.	.	.	.	.	.	.	.
Marsippospermum philippii	.	.	.	.	.	.	.	.	.	.	.	.	.	.	.	.	.	.	.	.	.	.	.	.	.	.	.	.	.
Marsippospermum reichei	.	.	.	.	.	.	.	.	.	.	.	.	.	.	.	.	.	.	.	.	.	.	.	.	.	.	.	.	.
Montia fontana	1	.	.	.	1	.	.	.	.	.	.	.	.	.	.	.	.	.	.	.	.	.	.	.	.	.	.	.	.
Muhlenbergia asperifolia	.	.	.	.	.	.	.	.	.	.	.	.	.	.	.	.	.	.	.	.	.	.	.	.	.	.	.	.	.
Myriophyllum quitense	.	.	.	.	.	.	.	.	.	.	.	1	.	1	.	.	.	.	1	1	1	.	.	1	1	1	.	1	.
Myrosmodes nervosa	.	.	.	.	.	.	.	.	.	.	.	.	.	.	.	.	.	.	.	.	.	.	.	.	.	.	.	.	.
Myrosmodes paludosa	.	.	.	.	.	.	.	.	.	.	.	.	.	.	.	.	.	.	.	.	.	.	.	.	.	.	.	.	.
Myrteola nummularia	.	.	.	.	.	.	.	.	.	.	.	.	.	.	.	.	.	.	.	.	.	.	.	.	.	.	.	.	.
Nanodea muscosa	.	.	.	.	.	.	.	.	.	.	.	.	.	.	.	.	.	.	.	.	.	.	.	.	.	.	.	.	.
Neobartsia crenoloba	.	.	.	.	.	.	.	.	.	.	.	.	.	.	.	.	.	.	.	.	.	.	.	.	.	.	.	.	.
Neobartsia pedicularoides	.	.	.	.	.	.	.	.	.	.	.	.	.	.	.	.	.	.	.	.	.	.	.	.	.	.	.	.	.
Neobartsia peruviana	.	.	.	.	.	.	.	.	.	.	.	.	.	.	.	.	.	.	.	.	.	.	.	.	.	.	.	.	.
Nertera granadensis	.	.	.	.	.	.	.	.	.	.	.	.	.	.	.	.	.	.	.	.	.	.	.	.	.	.	.	.	.
Nicoraepoa andina	.	.	.	.	.	.	.	.	.	.	.	.	.	.	.	.	.	.	.	.	.	.	.	.	.	.	.	.	.
Nicoraepoa pugionifolia	.	.	.	.	.	.	.	.	.	.	.	.	.	.	.	.	.	.	.	.	.	.	.	.	.	.	.	.	.
Nicoraepoa subenervis	.	.	.	.	.	.	.	.	.	.	.	.	.	.	.	.	.	.	.	.	.	.	.	.	.	.	.	.	.

Bog	291	292	293	294	295	296	297	298	299	300	301	302	303	304	305	306	307	308	309	310	311	312	313	314	315	316	317	318	319
Nitrophila australis	.	.	.	.	.	.	.	.	.	.	.	.	.	.	.	.	.	.	.	.	.	.	.	.	.	.	.	.	.
Nothofagus antarctica	.	.	.	.	.	.	.	.	.	.	.	.	.	.	.	.	.	.	.	.	.	.	.	.	.	.	.	.	.
Nototriche rugosa	.	.	.	.	.	.	.	.	.	.	.	.	.	.	.	.	.	.	.	.	.	.	.	.	.	.	.	.	.
Ochetophila nana	.	.	.	.	.	.	.	.	.	.	.	.	.	.	.	.	.	.	.	.	.	.	.	.	.	.	.	.	.
Olsynium junceum	.	.	.	.	.	.	.	.	.	.	.	.	.	.	.	.	.	.	.	.	.	.	.	.	.	.	.	.	.
Oreobolus obtusangulus	.	.	.	.	.	.	.	.	.	.	.	.	.	.	.	.	.	.	.	.	.	.	.	.	.	.	.	.	.
Oritrophium limnophilum	.	.	.	.	.	.	.	.	.	.	.	.	.	.	.	.	.	.	.	.	.	.	.	.	.	.	.	.	.
Osmorhiza glabrata	.	.	.	.	.	.	.	.	.	.	.	.	.	.	.	.	.	.	.	.	.	.	.	.	.	.	.	.	.
Ourisia alpina	.	.	.	.	.	.	.	.	.	.	.	.	.	.	.	.	.	.	.	.	.	.	.	.	.	.	.	.	.
Ourisia muscosa	.	.	.	.	.	.	.	.	.	.	.	.	.	.	.	.	.	.	.	.	.	.	.	.	.	.	.	.	1
Ourisia ruelloides	.	.	.	.	.	.	.	.	.	.	.	.	.	.	.	.	.	.	.	.	.	.	.	.	.	.	.	.	.
Oxychloe andina	1	1	1	1	1	1	.	1	1	1	1	1	1	1	1	1	1	.	1	1	1	1	1	1	1	1	1	1	.
Oxychloe bisexualis	.	.	.	.	.	.	.	.	.	.	.	.	.	.	.	.	.	.	.	.	.	.	.	.	.	.	.	.	.
Oxychloe castellanosii	.	.	.	.	.	.	.	.	.	.	.	.	.	.	.	.	.	.	.	.	.	.	.	.	.	.	.	.	.
Oxychloe haumaniana	.	.	.	.	.	.	.	.	.	.	.	.	.	.	.	.	.	.	.	.	.	.	.	.	.	.	.	.	.
Oxychloe mendocina	.	.	.	.	.	.	.	.	.	.	.	.	.	.	.	.	.	.	.	.	.	.	.	.	.	.	.	.	.
Patosia clandestina	.	.	.	.	.	.	1	.	.	.	.	.	.	.	.	.	.	1	.	.	.	.	.	.	.	.	.	.	1
Perezia capito	.	.	.	.	.	.	.	.	.	.	.	.	.	.	.	.	.	.	.	.	.	.	.	.	.	.	.	.	.
Perezia delicata	.	.	.	.	.	.	.	.	.	.	.	.	.	.	.	.	.	.	.	.	.	.	.	.	.	.	.	.	.
Perezia fonkii	.	.	.	.	.	.	.	.	.	.	.	.	.	.	.	.	.	.	.	.	.	.	.	.	.	.	.	.	.
Perezia pedicularidifolia	.	.	.	.	.	.	.	.	.	.	.	.	.	.	.	.	.	.	.	.	.	.	.	.	.	.	.	.	.
Perezia pinnatifida	.	.	.	.	.	.	.	.	.	.	.	.	.	.	.	.	.	.	.	.	.	.	.	.	1	.	.	.	.
Petroravenia friesii	1	1	1	.	1	1	.	1	1	1	.	.	.	1	1	1	.	.	.	.	.	.	.	.	.	.	.	.	.
Petroravenia werdermannii	.	.	.	.	.	.	.	.	.	.	.	.	.	.	.	.	.	.	.	.	.	.	.	.	.	.	.	.	.
Phleum alpinum	.	.	.	.	.	.	.	.	.	.	.	.	.	.	.	.	.	.	.	.	.	.	.	.	.	.	.	.	.
Phylloscirpus acaulis	.	.	.	.	.	.	.	.	.	.	.	.	.	.	.	.	.	.	.	.	.	.	.	.	.	.	.	.	.
Phylloscirpus boliviensis	1	1	1	1	1	1	.	1	1	1	1	1	1	1	1	1	1	.	.	.	.	.	.	.	.	.	.	.	1
Phylloscirpus deserticola	.	1	.	.	.	.	1	.	.	.	1	.	.	.	.	.	.	1	.	.	.	1	.	.	1	1	1	1	1
Pinguicula antarctica	.	.	.	.	.	.	.	.	.	.	.	.	.	.	.	.	.	.	.	.	.	.	.	.	.	.	.	.	.
Plantago barbata	.	.	.	.	.	.	.	.	.	.	.	.	.	.	.	.	.	.	.	.	.	.	.	.	.	.	.	.	.
Plantago rigida	.	.	.	.	.	.	.	.	.	.	.	.	.	.	.	.	.	.	.	.	.	.	.	.	.	.	.	.	.
Plantago tubulosa	1	1	1	1	.	1	1	1	.	.	1	.	.	.	.	.	.	1	1	.	1	1	.	.	1	1	1	1	1

Bog	291	292	293	294	295	296	297	298	299	300	301	302	303	304	305	306	307	308	309	310	311	312	313	314	315	316	317	318	319
Plantago uniglumis	.	.	.	.	.	.	.	.	.	.	.	.	.	.	.	.	.	.	.	.	.	.	.	.	.	.	.	.	.
Poa alopecurus	.	.	.	.	.	.	.	.	.	.	.	.	.	.	.	.	.	.	.	.	.	.	.	.	.	.	.	.	.
Poa hachadoensis	.	.	.	.	.	.	.	.	.	.	.	.	.	.	.	.	.	.	.	.	.	.	.	.	.	.	.	.	.
Poa perligulata	1	1	.	.	1	1	.	1	1	1	.	.	.	1	.	1	.	1	.	.	.	1	.	.	.	1	.	.	.
Polypogon interruptus	.	.	.	.	.	.	.	.	.	.	.	.	.	.	.	.	.	.	.	.	.	.	.	.	.	.	.	.	.
Primula magellanica	.	.	.	.	.	.	.	.	.	.	.	.	.	.	.	.	.	.	.	.	.	.	.	.	.	.	.	.	.
Puccinellia frigida	.	.	.	.	.	.	.	.	.	.	.	.	.	.	.	.	.	.	.	.	.	.	.	.	.	.	.	.	.
Quinchamalium chilense	.	.	.	.	.	.	.	.	.	.	.	.	.	.	.	.	.	.	.	.	.	.	.	.	.	.	.	.	.
Ranunculus breviscapus	.	.	.	.	.	.	.	.	.	.	.	.	.	.	.	.	.	.	.	.	.	.	.	.	.	.	.	.	.
Ranunculus fuegianus	.	.	.	.	.	.	.	.	.	.	.	.	.	.	.	.	.	.	.	.	.	.	.	.	.	.	.	.	.
Ranunculus mandonianus	.	.	.	.	.	.	.	.	.	.	.	.	.	1	1	.	.	.	1	.	.	.	.	1	1	1	.	.	.
Ranunculus peduncularis	.	.	.	.	.	.	.	.	.	.	.	.	.	.	.	.	.	.	.	.	.	.	.	.	.	.	.	.	.
Ranunculus trichophyllus	.	.	.	.	.	.	.	.	.	.	.	.	.	.	.	.	.	.	.	.	.	.	.	.	1	.	.	.	.
Halerpestes uniflora	.	.	.	.	.	.	.	.	.	.	.	.	.	.	.	.	.	.	.	.	.	.	.	.	.	.	.	.	.
Rubus geoides	.	.	.	.	.	.	.	.	.	.	.	.	.	.	.	.	.	.	.	.	.	.	.	.	.	.	.	.	.
Rumex magellanicus	.	.	.	.	.	.	.	.	.	.	.	.	.	.	.	.	.	.	.	.	.	.	.	.	.	.	.	.	.
Rytidosperma lechleri	.	.	.	.	.	.	.	.	.	.	.	.	.	.	.	.	.	.	.	.	.	.	.	.	.	.	.	.	.
Sarcocornia pulvinata	.	.	.	.	.	.	.	.	.	.	.	.	.	.	.	.	.	.	.	.	.	.	.	.	.	.	.	.	.
Schoenoplectus pungens	.	.	.	.	.	.	.	.	.	.	.	.	.	.	.	.	.	.	.	.	.	.	.	.	.	.	.	.	.
Schoenus andinus	.	.	.	.	.	.	.	.	.	.	.	.	.	.	.	.	.	.	.	.	.	.	.	.	.	.	.	.	.
Senecio breviscapus	.	.	.	.	.	.	.	.	.	.	.	.	.	.	.	.	.	.	.	.	.	.	.	.	.	.	.	.	.
Senecio diemii	.	.	.	.	.	.	.	.	.	.	.	.	.	.	.	.	.	.	.	.	.	.	.	.	.	.	.	.	.
Senecio fistulosus	.	.	.	.	.	.	.	.	.	.	.	.	.	.	.	.	.	.	.	.	.	.	.	.	.	.	.	.	.
Senecio parodii	.	.	.	.	.	.	.	.	.	.	.	.	.	.	.	.	.	.	.	.	.	.	.	.	.	.	.	.	.
Senecio peteroanus	.	.	.	.	.	.	.	.	.	.	.	.	.	.	.	.	.	.	.	.	.	.	.	.	.	.	.	.	.
Senecio serratifolius	1	.	.	.	1	.	.	.	.	1	.	.	.	1	1	.	.	.	.	.	.	.	.	.	.	.	.	.	.
Senecio trifurcatus	.	.	.	.	.	.	.	.	.	.	.	.	.	.	.	.	.	.	.	.	.	.	.	.	.	.	.	.	.
Sisyrinchium chilense	.	.	.	.	.	.	.	.	.	.	.	.	.	.	.	.	.	.	.	.	.	.	.	.	.	.	.	.	.
Sisyrinchium patagonicum	.	.	.	.	.	.	.	.	.	.	.	.	.	.	.	.	.	.	.	.	.	.	.	.	.	.	.	.	.
Sisyrinchium pearcei	.	.	.	.	.	.	.	.	.	.	.	.	.	.	.	.	.	.	.	.	.	.	.	.	.	.	.	.	.
Stellaria debilis	.	.	.	.	.	.	.	.	.	.	.	.	.	.	.	.	.	.	.	.	.	.	.	.	.	.	.	.	.
Stuckenia filiformis	.	.	.	.	.	.	.	.	1	1	1	1	.	.	.	1	.	1	1	.	1	1	1	1	1	1	.	1	.

Bog	291	292	293	294	295	296	297	298	299	300	301	302	303	304	305	306	307	308	309	310	311	312	313	314	315	316	317	318	319
Stuckenia striata	.	.	.	.	.	.	.	.	.	.	.	.	.	.	.	.	.	.	.	.	.	.	.	.	.	.	.	.	.
Symphyotrichum peteroanum	.	.	.	.	.	.	.	.	.	.	.	.	.	.	.	.	.	.	.	.	.	.	.	.	.	.	.	.	.
Symphyotrichum vahlii	.	.	.	.	.	.	.	.	.	.	.	.	.	.	.	.	.	.	.	.	.	.	.	.	.	.	.	.	.
Tetroncium magellanicum	.	.	.	.	.	.	.	.	.	.	.	.	.	.	.	.	.	.	.	.	.	.	.	.	.	.	.	.	.
Tribeles australis	.	.	.	.	.	.	.	.	.	.	.	.	.	.	.	.	.	.	.	.	.	.	.	.	.	.	.	.	.
Trifolium amabile	.	.	.	.	.	.	.	.	.	.	.	.	.	.	.	.	.	.	.	.	.	.	.	.	.	.	.	.	.
Trifolium polymorphum	.	.	.	.	.	.	.	.	.	.	.	.	.	.	.	.	.	.	.	.	.	.	.	.	.	.	.	.	.
Triglochin concinna	.	.	.	.	.	.	.	.	.	.	.	.	.	.	.	.	.	.	.	.	1	.	.	.	.	.	.	.	.
Triglochin palustris	.	.	.	.	.	.	.	.	.	.	.	.	.	.	.	.	.	.	.	.	.	.	.	.	.	.	.	.	.
Triglochin striata	.	.	.	.	.	.	.	.	.	.	.	.	.	.	.	.	.	.	.	.	.	.	.	.	.	.	.	.	.
Trisetum caudulatum	.	.	.	.	.	.	.	.	.	.	.	.	.	.	.	.	.	.	.	.	.	.	.	.	.	.	.	.	.
Trisetum preslei	.	.	.	.	.	.	.	.	.	.	.	.	.	.	.	.	.	.	.	.	.	.	.	.	.	.	.	.	.
Koeleria spicata	.	.	.	.	.	.	.	.	.	.	.	.	.	.	.	.	.	.	.	.	.	.	.	.	.	.	.	.	.
Utricularia gibba	.	.	.	.	.	.	.	.	.	.	.	.	.	.	.	.	.	.	.	.	.	.	.	.	.	.	.	.	.
Vahlodea atropurpurea	.	.	.	.	.	.	.	.	.	.	.	.	.	.	.	.	.	.	.	.	.	.	.	.	.	.	.	.	.
Valeriana fonckii	.	.	.	.	.	.	.	.	.	.	.	.	.	.	.	.	.	.	.	.	.	.	.	.	.	.	.	.	.
Valeriana macrorhiza	.	.	.	.	.	.	.	.	.	.	.	.	.	.	.	.	.	.	.	.	.	.	.	.	.	.	.	.	.
Viola pygmaea	.	.	.	.	.	.	.	.	.	.	.	.	.	.	.	.	.	.	.	.	.	.	.	.	.	.	.	.	.
Werneria apiculata	.	.	.	.	.	.	.	.	.	.	.	.	.	.	.	.	.	.	.	.	.	.	.	.	.	.	.	.	.
Werneria pinnatifida	1	1	.	1	.	.	1	.	.	.	1	.	.	.	.	.	.	1	.	.	.	.	.	.	.	.	.	1	1
Werneria pygmaea	1	1	1	1	1	.	1	.	.	.	1	.	.	1	.	.	.	1	1	.	1	.	.	.	1	.	1	1	1
Werneria solivifolia	1	1	.	.	.	1	.	1	1	1	1	.	1	1	.	1	.	1	.	.	.	.	.	.	.	.	.	.	1
Werneria spathulata	.	1	.	.	1	1	.	.	.	.	1	.	.	.	.	.	.	.	1	1	1	.	.	.	.	1	.	1	.
Xenophyllum incisum	.	.	.	.	.	.	.	.	.	.	.	.	1	.	1	.	.	.	.	.	1	.	.	.	.	1	.	.	.
Zameioscirpus atacamensis	.	.	.	.	.	.	.	.	.	.	1	1	.	.	.	.	.	.	.	.	1	1	.	.	.	.	.	.	.
Zameioscirpus gaimardiodes	.	.	.	.	.	.	.	.	.	.	.	.	.	.	.	.	.	.	.	.	.	.	.	.	.	.	.	.	.
Zameioscirpus muticus	1	1	1	1	1	1	1	1	1	1	1	1	1	1	1	1	1	.	1	1	1	1	1	1	1	1	1	1	.

Bog	320	321	322	323	324	325	326	327	328	329	330	331	332	333	334	335	336	337	338	339	340	341	342	343	344	345	346	347	348
**Operational zone**	N	N	N	N	N	N	N	N	N	N	N	N	N	N	N	N	N	N	N	N	N	N	N	N	N	N	N	N	N
**Cluster**	1	1	1	1	1	1	1	1	1	1	1	1	1	1	1	1	1	1	1	1	3	1	3	1	3	3	3	4	1
**Bioregion**	N	N	N	N	N	N	N	N	N	N	N	N	N	N	N	N	N	N	T	N	T	N	N	N	N	T	T	N	N
**Longitude**	-67.37	-67.95	-67.91	-67.66	-67.82	-67.52	-66.3	-66.29	-66.3	-66.3	-65.7	-67.35	-67.51	-67.48	-67.3	-67.35	-67.3	-68.05	-67.61	-67.96	-67.2	-66.43	-66.55	-66.58	-67.64	-67.26	-67.86	-67.74	-66.13
**Latitude**	-22.29	-21.54	-21.54	-21.31	-21.58	-21.6	-21.25	-21.32	-21.32	-21.31	-19.7	-22.2	-22.67	-22.5	-22.79	-22.79	-22.79	-22.07	-22.61	-21.72	-22.6	-19.27	-20.22	-20.11	-21.4	-23	-21.63	-22.21	-19.14
Acaena antarctica	.	.	.	.	.	.	.	.	.	.	.	.	.	.	.	.	.	.	.	.	.	.	.	.	.	.	.	.	.
Acaena macrocephala	.	.	.	.	.	.	.	.	.	.	.	.	.	.	.	.	.	.	.	.	.	.	.	.	.	.	.	.	.
Acaena magellanica	.	.	.	.	.	.	.	.	.	.	.	.	.	.	.	.	.	.	.	.	.	.	.	.	.	.	.	.	.
Acaena ovalifolia	.	.	.	.	.	.	.	.	.	.	.	.	.	.	.	.	.	.	.	.	.	.	.	.	.	.	.	.	.
Acaena pinnatifida	.	.	.	.	.	.	.	.	.	.	.	.	.	.	.	.	.	.	.	.	.	.	.	.	.	.	.	.	.
Adesmia retusa	.	.	.	.	.	.	.	.	.	.	.	.	.	.	.	.	.	.	.	.	.	.	.	.	.	.	.	.	.
Agrostis breviculmis	.	.	.	.	.	.	.	.	.	.	.	.	.	.	.	.	.	.	.	.	.	.	.	.	.	.	.	.	.
Agrostis imberbis	.	.	.	.	.	.	.	.	.	.	.	.	.	.	.	.	.	.	.	.	.	.	.	.	.	.	.	.	.
Agrostis meyenii	.	.	.	.	.	.	.	.	.	.	.	.	.	.	.	.	.	.	.	.	.	.	.	.	.	.	.	.	.
Agrostis perennans	.	.	.	.	.	.	.	.	.	.	.	.	.	.	.	.	.	.	.	.	.	.	.	.	.	.	.	.	.
Alchemilla pinnata	.	.	.	.	.	.	.	.	.	.	.	.	.	.	.	.	.	.	.	.	.	.	.	.	.	.	.	.	.
Alopecurus magellanicus	.	.	.	.	.	.	.	.	.	.	.	.	.	.	.	.	.	.	.	.	.	.	.	.	.	.	.	.	.
Amphiscirpus nevadensis	.	.	.	.	.	.	.	.	.	.	.	.	.	.	.	.	.	.	.	.	.	.	.	.	.	.	.	.	.
Anagallis alternifolia	.	.	.	.	.	.	.	.	.	.	.	.	.	.	.	.	.	.	.	.	.	.	.	.	.	.	.	.	.
Antennaria chilensis	.	.	.	.	.	.	.	.	.	.	.	.	.	.	.	.	.	.	.	.	.	.	.	.	.	.	.	.	.
Anthoxanthum redolens	.	.	.	.	.	.	.	.	.	.	.	.	.	.	.	.	.	.	.	.	.	.	.	.	.	.	.	.	.
Apium panul	.	.	.	.	.	.	.	.	.	.	.	.	.	.	.	.	.	.	.	.	.	.	.	.	.	.	.	.	.
Arenaria rivularis	.	.	.	.	.	.	.	.	.	.	.	.	.	.	.	.	.	.	.	.	.	.	.	.	.	.	.	.	.
Arenaria serpens	.	.	.	.	.	.	.	.	.	.	.	.	.	.	.	.	.	.	.	.	.	.	.	.	.	.	.	.	.
Arjona pusilla	.	.	.	.	.	.	.	.	.	.	.	.	.	.	.	.	.	.	.	.	.	.	.	.	.	.	.	.	.
Astragalus bustillosii	.	.	.	.	.	.	.	.	.	.	.	.	.	.	.	.	.	.	.	.	.	.	.	.	.	.	.	.	.
Astragalus micranthellus	.	.	.	.	.	.	.	.	.	.	.	.	.	.	.	.	.	.	.	.	.	.	.	.	.	.	.	.	.
Azolla filiculoides	.	.	.	.	.	.	.	.	.	.	.	.	.	.	.	.	.	.	.	1	.	.	.	.	.	.	.	.	.
Azorella boelckei	.	.	.	.	.	.	.	.	.	.	.	.	.	.	.	.	.	.	.	.	.	.	.	.	.	.	.	.	.

Bog	320	321	322	323	324	325	326	327	328	329	330	331	332	333	334	335	336	337	338	339	340	341	342	343	344	345	346	347	348
Azorella burkartii	.	.	.	.	.	.	.	.	.	.	.	.	.	.	.	.	.	.	.	.	.	.	.	.	.	.	.	.	.
Azorella cryptantha	.	.	.	.	.	.	.	.	.	.	.	.	.	.	.	.	.	.	.	.	.	.	.	.	.	.	.	.	.
Azorella lycopodioides	.	.	.	.	.	.	.	.	.	.	.	.	.	.	.	.	.	.	.	.	.	.	.	.	.	.	.	.	.
Azorella trifoliolata	.	.	.	.	.	.	.	.	.	.	.	.	.	.	.	.	.	.	.	.	.	.	.	.	.	.	.	.	.
Baccharis acaulis	.	.	.	.	.	.	.	.	.	.	.	.	.	.	.	.	.	.	.	.	.	.	1	.	.	.	.	.	.
Baccharis caespitosa	.	.	.	.	.	.	.	.	.	.	.	.	.	.	.	.	.	.	.	.	.	.	.	.	.	.	.	.	.
Baccharis magellanica	.	.	.	.	.	.	.	.	.	.	.	.	.	.	.	.	.	.	.	.	.	.	.	.	.	.	.	.	.
Belloa chilensis	.	.	.	.	.	.	.	.	.	.	.	.	.	.	.	.	.	.	.	.	.	.	.	.	.	.	.	.	.
Bromus catharticus	.	.	.	.	.	.	.	.	.	.	.	.	.	.	.	.	.	.	.	.	.	.	.	.	.	.	.	.	.
Calandrinia acaulis	.	.	.	.	.	.	.	.	.	.	.	.	.	.	.	.	.	.	.	.	.	.	.	.	.	.	.	.	.
Calandrinia compacta	.	.	1	.	1	1	1	1	1	1	1	.	1	1	.	1	.	1	1	1	.	.	.	1	.	.	.	1	.
Calceolaria biflora	.	.	.	.	.	.	.	.	.	.	.	.	.	.	.	.	.	.	.	.	.	.	.	.	.	.	.	.	.
Calceolaria cana	.	.	.	.	.	.	.	.	.	.	.	.	.	.	.	.	.	.	.	.	.	.	.	.	.	.	.	.	.
Calceolaria corymbosa	.	.	.	.	.	.	.	.	.	.	.	.	.	.	.	.	.	.	.	.	.	.	.	.	.	.	.	.	.
Calceolaria filicaulis	.	.	.	.	.	.	.	.	.	.	.	.	.	.	.	.	.	.	.	.	.	.	.	.	.	.	.	.	.
Callitriche lechleri	.	.	.	.	.	.	.	.	.	.	.	.	.	.	.	.	.	.	.	.	.	.	.	.	.	.	.	.	.
Caltha appendiculata	.	.	.	.	.	.	.	.	.	.	.	.	.	.	.	.	.	.	.	.	.	.	.	.	.	.	.	.	.
Caltha sagittata	.	.	.	.	.	.	.	.	.	.	.	.	.	.	.	.	.	.	.	.	.	.	.	.	.	.	.	.	.
Cardamine cordata	.	.	.	.	.	.	.	.	.	.	.	.	.	.	.	.	.	.	.	.	.	.	.	.	.	.	.	.	.
Cardamine glacialis	.	.	.	.	.	.	.	.	.	.	.	.	.	.	.	.	.	.	.	.	.	.	.	.	.	.	.	.	.
Cardamine tenuirostris	.	.	.	.	.	.	.	.	.	.	.	.	.	.	.	.	.	.	.	.	.	.	.	.	.	.	.	.	.
Cardamine volckmannii	.	.	.	.	.	.	.	.	.	.	.	.	.	.	.	.	.	.	.	.	.	.	.	.	.	.	.	.	.
Carex acaulis	.	.	.	.	.	.	.	.	.	.	.	.	.	.	.	.	.	.	.	.	.	.	.	.	.	.	.	.	.
Carex atropicta	.	.	.	.	.	.	.	.	.	.	.	.	.	.	.	.	.	.	.	.	.	.	.	.	.	.	.	.	.
Carex banksii	.	.	.	.	.	.	.	.	.	.	.	.	.	.	.	.	.	.	.	.	.	.	.	.	.	.	.	.	.
Carex caduca	.	.	.	.	.	.	.	.	.	.	.	.	.	.	.	.	.	.	.	.	.	.	.	.	.	.	.	.	.
Carex decidua	.	.	.	.	.	.	.	.	.	.	.	.	.	.	.	.	.	.	.	.	.	.	.	.	.	.	.	.	.
Carex fuscula	.	.	.	.	.	.	.	.	.	.	.	.	.	.	.	.	.	.	.	.	.	.	.	.	.	.	.	.	.
Carex gayana	.	.	.	.	.	.	.	.	.	.	.	.	.	.	.	.	.	.	.	.	.	.	.	.	.	.	.	.	.
Carex hypoleucos	.	.	.	.	.	.	.	.	.	.	.	.	.	.	.	.	.	.	.	.	.	.	.	.	.	.	.	.	.
Carex macloviana	.	.	.	.	.	.	.	.	.	.	.	.	.	.	.	.	.	.	.	.	.	.	.	.	.	.	.	.	.
Carex magellanica	.	.	.	.	.	.	.	.	.	.	.	.	.	.	.	.	.	.	.	.	.	.	.	.	.	.	.	.	.
Carex malmei	.	.	.	.	.	.	.	.	.	.	.	.	.	.	.	.	.	.	.	.	.	.	.	.	.	.	.	.	.
Carex maritima	1	1	1	1	1	1	1	1	1	1	1	1	1	1	1	1	1	1	1	1	.	.	1	1	.	1	.	1	1
Carex microglochin	.	.	.	.	.	.	.	.	.	.	.	.	.	.	.	.	.	.	.	.	.	.	.	.	.	.	.	.	.
Carex pleioneura	.	.	.	.	.	.	.	.	.	.	.	.	.	.	.	.	.	.	.	.	.	.	.	.	.	.	.	.	.
Carex ruthsatzae	.	.	.	.	.	.	.	.	.	.	.	.	.	.	.	.	.	.	.	.	.	.	.	.	.	.	.	.	.

Bog	320	321	322	323	324	325	326	327	328	329	330	331	332	333	334	335	336	337	338	339	340	341	342	343	344	345	346	347	348
Carex vallis-pulchrae	.	.	.	.	.	.	.	.	.	.	.	.	.	.	.	.	.	.	.	.	.	.	.	.	.	.	.	.	.
Carpha schoenoides	.	.	.	.	.	.	.	.	.	.	.	.	.	.	.	.	.	.	.	.	.	.	.	.	.	.	.	.	.
Castilleja pumila	.	.	.	.	.	.	.	.	.	.	.	.	.	.	.	.	.	.	.	.	.	1	.	.	.	.	.	.	1
Catabrosa werdermannii	.	.	.	.	.	.	.	.	.	.	.	.	.	.	.	.	.	.	.	.	.	.	.	.	.	.	.	.	.
Cerastium humifusum	.	.	.	.	.	.	.	.	.	.	.	.	.	.	.	.	.	.	.	.	.	.	.	.	.	.	.	.	.
Cerastium montioides	.	.	.	.	.	.	.	.	.	.	.	.	.	.	.	.	.	.	.	.	.	.	.	.	.	.	.	.	.
Chiliotrichum diffusum	.	.	.	.	.	.	.	.	.	.	.	.	.	.	.	.	.	.	.	.	.	.	.	.	.	.	.	.	.
Chusquea culeou	.	.	.	.	.	.	.	.	.	.	.	.	.	.	.	.	.	.	.	.	.	.	.	.	.	.	.	.	.
Colobanthus quitensis	.	.	.	.	.	.	.	1	1	.	.	.	.	.	.	.	.	.	.	.	.	.	.	1	.	.	.	.	.
Cortaderia egmontiana	.	.	.	.	.	.	.	.	.	.	.	.	.	.	.	.	.	.	.	.	.	.	.	.	.	.	.	.	.
Cotula mexicana	.	.	1	.	.	.	.	.	.	.	.	.	.	.	.	.	.	.	.	.	.	1	.	.	.	.	.	.	1
Crassula peduncularis	.	.	.	.	.	.	.	.	.	.	.	.	.	.	.	.	.	.	.	.	.	.	.	.	.	.	.	.	.
Cuatrecasasiella argentina	.	.	.	.	.	.	.	.	.	.	.	.	.	.	.	.	.	.	.	.	.	.	.	.	.	.	.	.	1
Deschampsia antarctica	.	.	.	.	.	.	.	.	.	.	.	.	.	.	.	.	.	.	.	.	.	.	.	.	.	.	.	.	.
Deschampsia caespitosa	.	.	.	.	.	.	.	.	.	.	.	.	.	.	.	.	.	.	.	.	.	.	.	.	.	.	.	.	.
Deschampsia patula	.	.	.	.	.	.	.	.	.	.	.	.	.	.	.	.	.	.	.	.	.	.	.	.	.	.	.	.	.
Cinnagrostis brevifolia	.	.	.	.	.	.	.	.	.	.	.	.	.	.	.	.	.	.	.	.	.	.	.	.	.	.	.	.	.
Deschampsia chrysantha	1	1	.	.	1	1	1	.	.	.	.	.	1	1	1	1	1	1	.	1	1	.	.	.	1	.	.	.	.
Cinnagrostis chrysophylla	.	.	.	.	.	.	.	.	.	.	.	.	.	.	.	.	.	.	.	.	.	.	.	.	.	.	.	.	.
Deschampsia chrysostachya	.	.	.	.	.	.	.	.	.	.	.	.	.	.	.	.	.	.	.	.	.	.	.	.	.	.	.	.	.
Deschampsia eminens	1	.	.	.	.	.	1	.	.	.	.	.	.	.	.	.	.	.	.	.	.	.	.	.	.	.	.	.	.
Deschampsia hackelii	.	.	.	.	.	.	.	.	.	.	.	.	.	.	.	.	.	.	.	.	.	.	.	.	.	.	.	.	.
Cinnagrostis minima	.	.	.	.	.	.	.	.	.	.	.	.	.	.	.	.	.	.	.	.	.	.	.	.	.	.	.	.	.
Deschampsia ovata	.	.	.	.	.	.	.	.	.	.	.	.	.	.	.	.	.	.	.	.	.	.	.	.	.	.	.	.	.
Cinnagrostis rigescens	.	.	.	.	.	.	.	.	.	.	1	.	.	.	.	.	.	.	.	.	.	1	.	.	.	.	.	.	1
Cinnagrostis spicigera	.	.	.	.	.	.	.	.	.	.	.	.	.	.	.	.	.	.	.	.	.	.	.	.	.	.	.	.	.
Cinnagrostis velutina	.	.	.	.	.	.	.	.	.	.	.	.	.	.	.	.	.	.	.	.	.	.	.	.	.	.	.	.	.
Cinnagrostis vicunarum	.	.	.	.	.	.	.	.	.	.	.	.	.	.	.	.	.	.	.	.	.	.	.	.	.	.	.	.	.
Distichia filamentosa	.	.	.	.	.	.	.	.	.	.	.	.	.	.	.	.	.	.	.	.	.	.	.	.	.	.	.	.	.
Distichia muscoides	.	.	.	.	.	.	.	.	.	.	1	.	.	.	.	.	.	.	.	.	.	1	.	.	.	.	.	.	1
Distichlis humilis	.	.	.	.	.	.	.	.	.	.	.	.	.	.	.	.	.	.	.	.	.	.	1	.	1	.	.	.	.

Bog	320	321	322	323	324	325	326	327	328	329	330	331	332	333	334	335	336	337	338	339	340	341	342	343	344	345	346	347	348
Distichlis scoparia	.	.	.	.	.	.	.	.	.	.	.	.	.	.	.	.	.	.	.	.	.	.	.	.	.	.	.	.	.
Distichlis spicata	.	.	.	.	.	.	.	.	.	.	.	.	.	.	.	.	.	.	.	.	.	.	.	.	.	.	.	.	.
Draba pusilla	.	.	.	.	.	.	.	.	.	.	.	.	.	.	.	.	.	.	.	.	.	.	.	.	.	.	.	.	.
Eleocharis melanomphala	.	.	.	.	.	.	.	.	.	.	.	.	.	.	.	.	.	.	.	.	.	.	.	.	.	.	.	.	.
Eleocharis pseudoalbibracteata	.	.	.	.	.	.	.	.	.	.	.	.	.	.	.	.	.	.	.	.	.	.	.	.	.	.	.	.	.
Elodea potamogeton	.	.	.	.	.	.	.	.	.	.	.	.	.	.	.	.	.	.	.	.	.	.	.	.	.	.	.	.	.
Empetrum rubrum	.	.	.	.	.	.	.	.	.	.	.	.	.	.	.	.	.	.	.	.	.	.	.	.	.	.	.	.	.
Epilobium australe	.	.	.	.	.	.	.	.	.	.	.	.	.	.	.	.	.	.	.	.	.	.	.	.	.	.	.	.	.
Epilobium barbeyanum	.	.	.	.	.	.	.	.	.	.	.	.	.	.	.	.	.	.	.	.	.	.	.	.	.	.	.	.	.
Epilobium ciliatum	.	.	.	.	.	.	.	.	.	.	.	.	.	.	.	.	.	.	.	.	.	.	.	.	.	.	.	.	.
Epilobium denticulatum	.	.	.	.	.	.	.	.	.	.	.	.	.	.	.	.	.	.	.	.	.	.	.	.	.	.	.	.	.
Epilobium fragile	.	.	.	.	.	.	.	.	.	.	.	.	.	.	.	.	.	.	.	.	.	.	.	.	.	.	.	.	.
Epilobium glaucum	.	.	.	.	.	.	.	.	.	.	.	.	.	.	.	.	.	.	.	.	.	.	.	.	.	.	.	.	.
Epilobium nivale	.	.	.	.	.	.	.	.	.	.	.	.	.	.	.	.	.	.	.	.	.	.	.	.	.	.	.	.	.
Erigeron andicola	.	.	.	.	.	.	.	.	.	.	.	.	.	.	.	.	.	.	.	.	.	.	.	.	.	.	.	.	.
Erigeron leptopetalus	.	.	.	.	.	.	.	.	.	.	.	.	.	.	.	.	.	.	.	.	.	.	.	.	.	.	.	.	.
Erigeron myosotis	.	.	.	.	.	.	.	.	.	.	.	.	.	.	.	.	.	.	.	.	.	.	.	.	.	.	.	.	.
Erigeron patagonicus	.	.	.	.	.	.	.	.	.	.	.	.	.	.	.	.	.	.	.	.	.	.	.	.	.	.	.	.	.
Erythranthe cuprea	.	.	.	.	.	.	.	.	.	.	.	.	.	.	.	.	.	.	.	.	.	.	.	.	.	.	.	.	.
Erythranthe depressa	.	.	.	.	.	.	.	.	.	.	.	.	.	.	.	.	.	.	.	.	.	.	.	.	.	.	.	.	.
Erythranthe glabrata	1	.	1	.	.	.	.	.	.	.	.	.	.	.	.	.	.	1	.	.	.	.	.	.	.	.	.	.	1
Erythranthe lutea	.	.	.	.	.	.	.	.	.	.	.	.	.	.	.	.	.	.	.	.	.	.	.	.	.	.	.	.	.
Escallonia virgata	.	.	.	.	.	.	.	.	.	.	.	.	.	.	.	.	.	.	.	.	.	.	.	.	.	.	.	.	.
Euphrasia antarctica	.	.	.	.	.	.	.	.	.	.	.	.	.	.	.	.	.	.	.	.	.	.	.	.	.	.	.	.	.
Euphrasia chrysantha	.	.	.	.	.	.	.	.	.	.	.	.	.	.	.	.	.	.	.	.	.	.	.	.	.	.	.	.	.
Euphrasia subexserta	.	.	.	.	.	.	.	.	.	.	.	.	.	.	.	.	.	.	.	.	.	.	.	.	.	.	.	.	.
Festuca hypsophila	.	.	.	.	.	.	.	.	.	.	.	.	.	.	.	.	.	.	.	.	.	.	.	.	.	.	.	.	.
Festuca kurtziana	.	.	.	.	.	.	.	.	.	.	.	.	.	.	.	.	.	.	.	.	.	.	.	.	.	.	.	.	.
Festuca lilloi	.	.	.	.	.	.	.	.	.	.	.	.	.	.	.	.	.	.	.	.	.	.	.	.	.	.	.	.	.
Festuca magellanica	.	.	.	.	.	.	.	.	.	.	.	.	.	.	.	.	.	.	.	.	.	.	.	.	.	.	.	.	.
Festuca nardifolia	.	.	.	.	.	.	.	.	.	.	.	.	.	.	.	.	.	.	.	.	.	.	.	.	.	.	.	.	.
Festuca rigescens	.	.	.	.	.	.	.	.	.	.	.	.	.	.	.	.	.	.	.	.	.	.	.	.	.	.	.	.	.
Festuca werdermannii	.	.	.	.	.	.	.	.	.	.	.	.	.	.	.	.	.	.	.	.	.	.	.	.	.	.	.	.	.
Frankenia triandra	.	.	.	.	.	.	.	.	.	.	.	.	.	.	.	.	.	.	.	.	.	.	.	.	.	.	.	.	.
Gamocarpha graminea	.	.	.	.	.	.	.	.	.	.	.	.	.	.	.	.	.	.	.	.	.	.	.	.	.	.	.	.	.

Bog	320	321	322	323	324	325	326	327	328	329	330	331	332	333	334	335	336	337	338	339	340	341	342	343	344	345	346	347	348
Gamocarpha ventosa	.	.	.	.	.	.	.	.	.	.	.	.	.	.	.	.	.	.	.	.	.	.	.	.	.	.	.	.	.
Gamochaeta chamissonis	.	.	.	.	.	.	.	.	.	.	.	.	.	.	.	.	.	.	.	.	.	.	.	.	.	.	.	.	.
Gamochaeta longipedicellata	.	.	.	.	.	.	.	.	.	.	.	.	.	.	.	.	.	.	.	.	.	.	.	.	.	.	.	.	.
Gamochaeta neuquensis	.	.	.	.	.	.	.	.	.	.	.	.	.	.	.	.	.	.	.	.	.	.	.	.	.	.	.	.	.
Gaultheria antarctica	.	.	.	.	.	.	.	.	.	.	.	.	.	.	.	.	.	.	.	.	.	.	.	.	.	.	.	.	.
Gaultheria caespitosa	.	.	.	.	.	.	.	.	.	.	.	.	.	.	.	.	.	.	.	.	.	.	.	.	.	.	.	.	.
Gaultheria pumila	.	.	.	.	.	.	.	.	.	.	.	.	.	.	.	.	.	.	.	.	.	.	.	.	.	.	.	.	.
Gavilea chica	.	.	.	.	.	.	.	.	.	.	.	.	.	.	.	.	.	.	.	.	.	.	.	.	.	.	.	.	.
Gentiana prostrata	1	1	.	1	.	1	1	1	1	1	1	1	.	.	1	1	.	1	.	.	.	1	.	1	1	.	.	.	1
Gentianella fiebrigii	.	.	.	.	.	.	.	.	.	.	.	.	.	.	.	.	.	.	.	.	.	.	.	.	.	.	.	.	.
Gentianella magellanica	.	.	.	.	.	.	.	.	.	.	.	.	.	.	.	.	.	.	.	.	.	.	.	.	.	.	.	.	.
Gentianella multicaulis	.	.	.	.	.	.	.	.	.	.	.	.	.	.	.	.	.	.	.	.	.	.	.	.	.	.	.	.	.
Gentianella ottonis	.	.	.	.	.	.	.	.	.	.	.	.	.	.	.	.	.	.	.	.	.	.	.	.	.	.	.	.	.
Gentianella primuloides	.	.	.	.	.	.	.	.	.	.	.	.	.	.	.	.	.	.	.	.	.	.	.	.	.	.	.	.	.
Gentianella pseudocrassula	.	.	.	.	.	.	.	.	.	.	.	.	.	.	.	.	.	.	.	.	.	.	.	.	.	.	.	.	.
Geranium sessiliflorum	.	.	.	.	.	.	.	.	.	.	.	.	.	.	.	.	.	.	.	.	.	.	.	.	.	.	.	.	.
Gunnera magellanica	.	.	.	.	.	.	.	.	.	.	.	.	.	.	.	.	.	.	.	.	.	.	.	.	.	.	.	.	.
Halenia caespitosa	.	.	.	.	.	.	.	.	.	.	.	.	.	.	.	.	.	.	.	.	.	.	.	.	.	.	.	.	.
Halerpestes cymbalaria	.	.	.	.	.	.	.	.	.	.	.	.	.	.	.	.	.	.	.	.	.	.	.	.	.	.	.	.	.
Halerpestes exilis	1	.	1	.	1	.	.	1	1	1	.	.	.	1	.	.	.	1	1	1	1	.	1	1	.	.	.	.	.
Hieracium antarcticum	.	.	.	.	.	.	.	.	.	.	.	.	.	.	.	.	.	.	.	.	.	.	.	.	.	.	.	.	.
Hordeum comosum	.	.	.	.	.	.	.	.	.	.	.	.	.	.	.	.	.	.	.	.	.	.	.	.	.	.	.	.	.
Hordeum muticum	.	.	.	.	.	.	.	.	.	.	.	.	.	.	.	.	.	.	.	.	.	.	.	.	.	.	.	.	.
Hypochaeris acaulis	.	.	.	.	.	.	.	.	.	.	.	.	.	.	.	.	.	.	.	.	.	.	.	.	.	.	.	.	.
Hypochaeris chondrilloides	.	.	.	.	.	.	.	.	.	.	.	.	.	.	.	.	.	.	.	.	.	.	.	.	.	.	.	.	.
Hypochaeris meyeniana	.	.	.	.	.	.	.	.	.	.	.	.	.	.	.	.	.	.	.	.	.	.	.	.	.	.	.	.	.
Hypochaeris palustris	.	.	.	.	.	.	.	.	.	.	.	.	.	.	.	.	.	.	.	.	.	.	.	.	.	.	.	.	.
Hypochaeris taraxacoides	1	.	.	1	.	.	1	.	1	.	1	.	.	.	.	.	.	.	.	.	.	1	1	.	1	1	.	.	1
Hypochaeris tenerifolia	.	.	.	.	.	.	.	.	.	.	.	.	.	.	.	.	.	.	.	.	.	.	.	.	.	.	.	.	.
Isolepis nigricans	.	.	.	.	.	.	.	.	.	.	.	.	.	.	.	.	.	.	.	.	.	.	.	.	.	.	.	.	.

Bog	320	321	322	323	324	325	326	327	328	329	330	331	332	333	334	335	336	337	338	339	340	341	342	343	344	345	346	347	348
Isolepsis inundata	.	.	.	.	.	.	.	.	.	.	.	.	.	.	.	.	.	.	.	.	.	.	.	.	.	.	.	.	.
Juncus balticus	.	.	.	.	.	.	.	.	.	.	.	.	.	.	.	.	.	.	.	.	.	.	.	.	.	.	.	.	.
Juncus stipulatus	1	.	.	1	.	.	1	.	.	.	1	1	.	.	.	.	.	.	.	.	.	1	.	.	.	.	.	.	1
Koeleria kurtzii	.	.	.	.	.	.	.	.	.	.	.	.	.	.	.	.	.	.	.	.	.	.	.	.	.	.	.	.	.
Lachemilla diplophylla	.	.	.	.	.	.	.	.	.	.	1	.	.	.	.	.	.	.	.	.	.	1	.	.	.	.	.	.	1
Lachemilla pinnata	.	.	.	.	.	.	1	1	1	1	1	.	.	.	.	.	.	.	.	.	.	.	.	.	.	.	.	.	1
Lagenophora nudicaulis	.	.	.	.	.	.	.	.	.	.	.	.	.	.	.	.	.	.	.	.	.	.	.	.	.	.	.	.	.
Lemna minuta	1	.	1	.	.	.	.	.	.	.	.	.	.	.	.	.	.	1	.	.	.	.	1	.	.	.	.	.	.
Leptinella scariosa	.	.	.	.	.	.	.	.	.	.	.	.	.	.	.	.	.	.	.	.	.	.	.	.	.	.	.	.	.
Leucheria candidissima	.	.	.	.	.	.	.	.	.	.	.	.	.	.	.	.	.	.	.	.	.	.	.	.	.	.	.	.	.
Leucheria nutans	.	.	.	.	.	.	.	.	.	.	.	.	.	.	.	.	.	.	.	.	.	.	.	.	.	.	.	.	.
Lilaea scilloides	.	.	.	1	.	.	.	.	.	.	.	.	.	.	.	.	.	.	.	.	.	.	.	.	.	.	.	.	.
Lilaeopsis macloviana	1	1	1	1	1	1	.	.	1	1	.	1	.	1	.	.	.	1	1	1	.	1	1	1	1	.	.	.	.
Limosella australis	.	.	.	.	.	.	.	.	.	.	.	.	.	.	.	.	.	.	.	.	.	.	.	.	.	.	.	.	.
Lobelia oligophylla	1	1	1	1	1	1	1	1	1	1	1	1	.	1	.	.	1	1	1	1	.	1	1	1	1	.	.	.	1
Luzula brachyphylla	.	.	.	.	.	.	.	.	.	.	.	.	.	.	.	.	.	.	.	.	.	.	.	.	.	.	.	.	.
Luzula chilensis	.	.	.	.	.	.	.	.	.	.	.	.	.	.	.	.	.	.	.	.	.	.	.	.	.	.	.	.	.
Luzula racemosa	.	.	.	.	.	.	.	.	.	.	.	.	.	.	.	.	.	.	.	.	.	.	.	.	.	.	.	.	.
Luzula vulcanica	.	.	.	.	.	.	.	.	.	.	.	.	.	.	.	.	.	.	.	.	.	.	.	.	.	.	.	.	.
Lysipomia pumila	.	.	.	.	.	.	.	.	.	.	.	.	.	.	.	.	.	.	.	.	.	.	.	.	.	.	.	.	.
Marsippospermum philippii	.	.	.	.	.	.	.	.	.	.	.	.	.	.	.	.	.	.	.	.	.	.	.	.	.	.	.	.	.
Marsippospermum reichei	.	.	.	.	.	.	.	.	.	.	.	.	.	.	.	.	.	.	.	.	.	.	.	.	.	.	.	.	.
Montia fontana	.	.	.	.	.	.	.	.	.	.	1	.	.	.	.	.	.	.	.	.	.	.	.	.	.	.	.	.	1
Muhlenbergia asperifolia	.	.	.	.	.	.	.	.	.	.	.	.	.	.	.	.	.	.	.	.	.	.	.	.	.	.	.	.	.
Myriophyllum quitense	1	1	1	.	.	.	.	.	.	.	.	1	.	1	.	.	.	.	1	.	.	.	.	.	1	.	.	.	.
Myrosmodes nervosa	.	.	.	.	.	.	.	.	.	.	.	.	.	.	.	.	.	.	.	.	.	.	.	.	.	.	.	.	.
Myrosmodes paludosa	.	.	.	.	.	.	.	.	.	.	.	.	.	.	.	.	.	.	.	.	.	.	.	.	.	.	.	.	.
Myrteola nummularia	.	.	.	.	.	.	.	.	.	.	.	.	.	.	.	.	.	.	.	.	.	.	.	.	.	.	.	.	.
Nanodea muscosa	.	.	.	.	.	.	.	.	.	.	.	.	.	.	.	.	.	.	.	.	.	.	.	.	.	.	.	.	.
Neobartsia crenoloba	.	.	.	.	.	.	.	.	.	.	.	.	.	.	.	.	.	.	.	.	.	.	.	.	.	.	.	.	.
Neobartsia pedicularoides	.	.	.	.	.	.	.	.	.	.	.	.	.	.	.	.	.	.	.	.	.	.	.	.	.	.	.	.	.
Neobartsia peruviana	.	.	.	.	.	.	.	.	.	.	.	.	.	.	.	.	.	.	.	.	.	.	.	.	.	.	.	.	.

**Bog**	**320**	**321**	**322**	**323**	**324**	**325**	**326**	**327**	**328**	**329**	**330**	**331**	**332**	**333**	**334**	**335**	**336**	**337**	**338**	**339**	**340**	**341**	**342**	**343**	**344**	**345**	**346**	**347**	**348**
Nertera granadensis	.	.	.	.	.	.	.	.	.	.	.	.	.	.	.	.	.	.	.	.	.	.	.	.	.	.	.	.	.
Nicoraepoa andina	.	.	.	.	.	.	.	.	.	.	.	.	.	.	.	.	.	.	.	.	.	.	.	.	.	.	.	.	.
Nicoraepoa pugionifolia	.	.	.	.	.	.	.	.	.	.	.	.	.	.	.	.	.	.	.	.	.	.	.	.	.	.	.	.	.
Nicoraepoa subenervis	.	.	.	.	.	.	.	.	.	.	.	.	.	.	.	.	.	.	.	.	.	.	.	.	.	.	.	.	.
Nitrophila australis	.	.	.	.	.	.	.	.	.	.	.	.	.	.	.	.	.	.	.	.	.	.	.	.	.	.	.	.	.
Nothofagus antarctica	.	.	.	.	.	.	.	.	.	.	.	.	.	.	.	.	.	.	.	.	.	.	.	.	.	.	.	.	.
Nototriche rugosa	.	.	.	.	.	.	.	.	.	.	.	.	.	.	.	.	.	.	.	.	.	.	.	.	.	.	.	.	.
Ochetophila nana	.	.	.	.	.	.	.	.	.	.	.	.	.	.	.	.	.	.	.	.	.	.	.	.	.	.	.	.	.
Olsynium junceum	.	.	.	.	.	.	.	.	.	.	.	.	.	.	.	.	.	.	.	.	.	.	.	.	.	.	.	.	.
Oreobolus obtusangulus	.	.	.	.	.	.	.	.	.	.	.	.	.	.	.	.	.	.	.	.	.	.	.	.	.	.	.	.	.
Oritrophium limnophilum	.	.	.	.	.	.	.	.	.	.	.	.	.	.	.	.	.	.	.	.	.	.	.	.	.	.	.	.	.
Osmorhiza glabrata	.	.	.	.	.	.	.	.	.	.	.	.	.	.	.	.	.	.	.	.	.	.	.	.	.	.	.	.	.
Ourisia alpina	.	.	.	.	.	.	.	.	.	.	.	.	.	.	.	.	.	.	.	.	.	.	.	.	.	.	.	.	.
Ourisia muscosa	.	.	.	.	.	.	.	.	.	.	.	.	.	.	.	.	.	.	.	.	.	.	.	.	.	.	.	.	.
Ourisia ruelloides	.	.	.	.	.	.	.	.	.	.	.	.	.	.	.	.	.	.	.	.	.	.	.	.	.	.	.	.	.
Oxychloe andina	1	1	1	1	1	1	1	1	1	1	1	1	1	1	1	1	1	1	1	1	1	.	.	.	.	.	.	.	.
Oxychloe bisexualis	.	.	.	.	.	.	.	.	.	.	.	.	.	.	.	.	.	.	.	.	.	.	.	.	.	.	.	.	.
Oxychloe castellanosii	.	.	.	.	.	.	.	.	.	.	.	.	.	.	.	.	.	.	.	.	.	.	.	.	.	.	.	.	.
Oxychloe haumaniana	.	.	.	.	.	.	.	.	.	.	.	.	.	.	.	.	.	.	.	.	.	.	.	.	.	.	.	.	.
Oxychloe mendocina	.	.	.	.	.	.	.	.	.	.	.	.	.	.	.	.	.	.	.	.	.	.	.	.	.	.	.	.	.
Patosia clandestina	.	.	.	.	.	.	.	.	.	.	1	.	.	.	.	.	.	.	.	.	.	1	.	.	.	.	.	.	1
Perezia capito	.	.	.	.	.	.	.	.	.	.	.	.	.	.	.	.	.	.	.	.	.	.	.	.	.	.	.	.	.
Perezia delicata	.	.	.	.	.	.	.	.	.	.	.	.	.	.	.	.	.	.	.	.	.	.	.	.	.	.	.	.	.
Perezia fonkii	.	.	.	.	.	.	.	.	.	.	.	.	.	.	.	.	.	.	.	.	.	.	.	.	.	.	.	.	.
Perezia pedicularidifolia	.	.	.	.	.	.	.	.	.	.	.	.	.	.	.	.	.	.	.	.	.	.	.	.	.	.	.	.	.
Perezia pinnatifida	.	.	.	.	.	.	.	.	.	.	.	.	.	.	.	.	.	.	.	.	.	.	.	1	.	.	.	.	.
Petroravenia friesii	.	.	1	.	.	.	.	1	1	1	.	.	.	1	1	1	.	.	.	.	.	.	.	.	.	.	.	.	.
Petroravenia werdermannii	.	.	.	.	.	.	.	.	.	.	.	.	.	.	.	.	.	.	.	.	.	.	.	.	.	.	.	.	.
Phleum alpinum	.	.	.	.	.	.	.	.	.	.	.	.	.	.	.	.	.	.	.	.	.	.	.	.	.	.	.	.	.
Phylloscirpus acaulis	.	.	.	.	.	.	.	.	.	.	.	.	.	.	.	.	.	.	.	.	.	.	.	.	.	.	.	.	.
Phylloscirpus boliviensis	.	.	.	.	.	.	.	.	.	.	1	.	.	.	.	.	.	.	.	.	.	1	.	.	.	.	.	.	.

Bog	320	321	322	323	324	325	326	327	328	329	330	331	332	333	334	335	336	337	338	339	340	341	342	343	344	345	346	347	348
Phylloscirpus deserticola	1	.	1	1	.	1	.	1	.	.	1	1	.	.	.	.	.	1	.	.	.	1	1	1	1	.	.	.	1
Pinguicula antarctica	.	.	.	.	.	.	.	.	.	.	.	.	.	.	.	.	.	.	.	.	.	.	.	.	.	.	.	.	.
Plantago barbata	.	.	.	.	.	.	.	.	.	.	.	.	.	.	.	.	.	.	.	.	.	.	.	.	.	.	.	.	.
Plantago rigida	.	.	.	.	.	.	.	.	.	.	.	.	.	.	.	.	.	.	.	.	.	.	.	.	.	.	.	.	.
Plantago tubulosa	1	.	1	1	1	1	1	1	1	1	1	1	.	1	.	.	.	1	.	.	.	1	1	1	1	.	1	.	1
Plantago uniglumis	.	.	.	.	.	.	.	.	.	.	.	.	.	.	.	.	.	.	.	.	.	.	.	.	.	.	.	.	.
Poa alopecurus	.	.	.	.	.	.	.	.	.	.	.	.	.	.	.	.	.	.	.	.	.	.	.	.	.	.	.	.	.
Poa hachadoensis	.	.	.	.	.	.	.	.	.	.	.	.	.	.	.	.	.	.	.	.	.	.	.	.	.	.	.	.	.
Poa perligulata	.	.	.	.	.	.	1	.	.	.	1	.	.	.	.	.	.	.	.	.	.	.	.	.	.	.	.	.	.
Polypogon interruptus	.	.	.	.	.	.	.	.	.	.	.	.	.	.	.	.	.	.	.	.	.	.	.	.	.	.	.	.	.
Primula magellanica	.	.	.	.	.	.	.	.	.	.	.	.	.	.	.	.	.	.	.	.	.	.	.	.	.	.	.	.	.
Puccinellia frigida	.	.	.	.	.	.	.	.	.	.	.	.	.	.	.	.	.	.	.	.	.	.	.	.	.	.	.	.	.
Quinchamalium chilense	.	.	.	.	.	.	.	.	.	.	.	.	.	.	.	.	.	.	.	.	.	.	.	.	.	.	.	.	.
Ranunculus breviscapus	.	.	.	.	.	.	.	.	.	.	.	.	.	.	.	.	.	.	.	.	.	.	.	.	.	.	.	.	.
Ranunculus fuegianus	.	.	.	.	.	.	.	.	.	.	.	.	.	.	.	.	.	.	.	.	.	.	.	.	.	.	.	.	.
Ranunculus mandonianus	1	1	1	.	.	.	.	.	.	.	.	.	.	1	.	.	.	1	.	.	.	.	.	.	1	.	.	.	.
Ranunculus peduncularis	.	.	.	.	.	.	.	.	.	.	.	.	.	.	.	.	.	.	.	.	.	.	.	.	.	.	.	.	.
Ranunculus trichophyllus	.	.	.	.	.	.	.	.	.	.	.	.	.	.	.	.	.	.	.	.	.	.	.	.	.	.	.	.	.
Halerpestes uniflora	.	.	.	.	.	.	.	.	.	.	.	.	.	.	.	.	.	.	.	.	.	.	.	.	.	.	.	.	.
Rubus geoides	.	.	.	.	.	.	.	.	.	.	.	.	.	.	.	.	.	.	.	.	.	.	.	.	.	.	.	.	.
Rumex magellanicus	.	.	.	.	.	.	.	.	.	.	.	.	.	.	.	.	.	.	.	.	.	.	.	.	.	.	.	.	.
Rytidosperma lechleri	.	.	.	.	.	.	.	.	.	.	.	.	.	.	.	.	.	.	.	.	.	.	.	.	.	.	.	.	.
Sarcocornia pulvinata	.	.	.	.	.	.	.	.	.	.	.	.	.	.	.	.	.	.	.	.	.	.	.	.	.	.	.	.	.
Schoenoplectus pungens	.	.	.	.	.	.	.	.	.	.	.	.	.	.	.	.	.	.	.	.	.	.	.	.	.	.	.	.	.
Schoenus andinus	.	.	.	.	.	.	.	.	.	.	.	.	.	.	.	.	.	.	.	.	.	.	.	.	.	.	.	.	.
Senecio breviscapus	.	.	.	.	.	.	.	.	.	.	.	.	.	.	.	.	.	.	.	.	.	.	.	.	.	.	.	.	.
Senecio diemii	.	.	.	.	.	.	.	.	.	.	.	.	.	.	.	.	.	.	.	.	.	.	.	.	.	.	.	.	.
Senecio fistulosus	.	.	.	.	.	.	.	.	.	.	.	.	.	.	.	.	.	.	.	.	.	.	.	.	.	.	.	.	.
Senecio parodii	.	.	.	.	.	.	.	.	.	.	.	.	.	.	.	.	.	.	.	.	.	.	.	.	.	.	.	.	.
Senecio peteroanus	.	.	.	.	.	.	.	.	.	.	.	.	.	.	.	.	.	.	.	.	.	.	.	.	.	.	.	.	.
Senecio serratifolius	.	.	.	.	.	.	.	.	.	.	.	.	.	.	.	.	1	.	.	.	.	.	.	.	.	.	.	.	.

**Bog**	**320**	**321**	**322**	**323**	**324**	**325**	**326**	**327**	**328**	**329**	**330**	**331**	**332**	**333**	**334**	**335**	**336**	**337**	**338**	**339**	**340**	**341**	**342**	**343**	**344**	**345**	**346**	**347**	**348**
Senecio trifurcatus	.	.	.	.	.	.	.	.	.	.	.	.	.	.	.	.	.	.	.	.	.	.	.	.	.	.	.	.	.
Sisyrinchium chilense	.	.	.	.	.	.	.	.	.	.	.	.	.	.	.	.	.	.	.	.	.	.	.	.	.	.	.	.	.
Sisyrinchium patagonicum	.	.	.	.	.	.	.	.	.	.	.	.	.	.	.	.	.	.	.	.	.	.	.	.	.	.	.	.	.
Sisyrinchium pearcei	.	.	.	.	.	.	.	.	.	.	.	.	.	.	.	.	.	.	.	.	.	.	.	.	.	.	.	.	.
Stellaria debilis	.	.	.	.	.	.	.	.	.	.	.	.	.	.	.	.	.	.	.	.	.	.	.	.	.	.	.	.	.
Stuckenia filiformis	1	.	.	.	1	.	.	.	.	.	.	1	.	1	.	.	.	1	1	1	1	.	.	.	1	1	.	.	.
Stuckenia striata	.	.	.	.	.	.	.	.	.	.	.	.	.	.	.	.	.	.	.	.	.	.	.	.	.	.	.	.	.
Symphyotrichum peteroanum	.	.	.	.	.	.	.	.	.	.	.	.	.	.	.	.	.	.	.	.	.	.	.	.	.	.	.	.	.
Symphyotrichum vahlii	.	.	.	.	.	.	.	.	.	.	.	.	.	.	.	.	.	.	.	.	.	.	.	.	.	.	.	.	.
Tetroncium magellanicum	.	.	.	.	.	.	.	.	.	.	.	.	.	.	.	.	.	.	.	.	.	.	.	.	.	.	.	.	.
Tribeles australis	.	.	.	.	.	.	.	.	.	.	.	.	.	.	.	.	.	.	.	.	.	.	.	.	.	.	.	.	.
Trifolium amabile	.	.	.	.	.	.	.	.	.	.	.	.	.	.	.	.	.	.	.	.	.	.	.	.	.	.	.	.	.
Trifolium polymorphum	.	.	.	.	.	.	.	.	.	.	.	.	.	.	.	.	.	.	.	.	.	.	.	.	.	.	.	.	.
Triglochin concinna	.	.	.	.	.	.	.	.	.	.	.	.	.	.	.	.	.	.	.	.	1	.	1	1	1	1	1	.	.
Triglochin palustris	.	.	.	.	.	.	.	.	.	.	.	.	.	.	.	.	.	.	.	.	.	.	.	.	.	.	.	.	.
Triglochin striata	.	.	.	.	.	.	.	.	.	.	.	.	.	.	.	.	.	.	.	.	.	.	.	.	.	.	.	.	.
Trisetum caudulatum	.	.	.	.	.	.	.	.	.	.	.	.	.	.	.	.	.	.	.	.	.	.	.	.	.	.	.	.	.
Trisetum preslei	.	.	.	.	.	.	.	.	.	.	.	.	.	.	.	.	.	.	.	.	.	.	.	.	.	.	.	.	.
Koeleria spicata	.	.	.	.	.	.	.	.	.	.	.	.	.	.	.	.	.	.	.	.	.	.	.	.	.	.	.	.	.
Utricularia gibba	.	.	.	.	.	.	.	.	.	.	.	.	.	.	.	.	.	.	.	.	.	.	.	.	.	.	.	.	.
Vahlodea atropurpurea	.	.	.	.	.	.	.	.	.	.	.	.	.	.	.	.	.	.	.	.	.	.	.	.	.	.	.	.	.
Valeriana fonckii	.	.	.	.	.	.	.	.	.	.	.	.	.	.	.	.	.	.	.	.	.	.	.	.	.	.	.	.	.
Valeriana macrorhiza	.	.	.	.	.	.	.	.	.	.	.	.	.	.	.	.	.	.	.	.	.	.	.	.	.	.	.	.	.
Viola pygmaea	.	.	.	.	.	.	.	.	.	.	.	.	.	.	.	.	.	.	.	.	.	.	.	.	.	.	.	.	.
Werneria apiculata	.	.	.	.	.	.	.	.	.	.	.	.	.	.	.	.	.	.	.	.	.	.	.	.	.	.	.	.	.
Werneria pinnatifida	1	.	.	.	.	.	.	1	1	1	1	.	.	.	1	.	.	.	.	.	.	.	.	.	.	.	.	.	.
Werneria pygmaea	1	.	.	1	.	.	1	1	1	1	1	1	.	.	.	.	.	.	1	.	.	1	.	1	1	.	.	.	1
Werneria solivifolia	.	.	.	.	.	.	.	.	.	1	.	.	1	1	1	1	.	.	.	.	.	.	.	.	.	.	.	.	1
Werneria spathulata	1	1	.	.	.	.	.	.	.	.	.	.	.	.	.	.	.	.	.	.	.	.	.	.	.	.	.	.	1
Xenophyllum incisum	.	.	1	.	1	1	.	.	.	.	.	.	.	.	.	1	.	1	1	1	.	.	.	.	.	1	.	1	.
Zameioscirpus atacamensis	1	.	.	.	.	.	.	.	.	.	.	1	.	.	.	.	.	1	1	1	1	.	1	1	1	1	1	1	.
Zameioscirpus gaimardiodes	.	.	.	.	.	.	.	.	.	.	.	.	.	.	.	.	.	.	.	.	.	.	.	.	.	.	.	.	.
Zameioscirpus muticus	1	1	1	1	1	1	1	1	1	1	1	1	1	1	1	1	1	1	1	.	.	1	.	1	.	1	.	.	1

Bog	349	350	351	352	353	354	355	356	357	358	359	360	361	362	363	364	365	366	367	368	369	370	371	372	373	374	375	376	377
**Operational zone**	**T**	**T**	**T**	**T**	**T**	**T**	**T**	**T**	**T**	**T**	**T**	**T**	**T**	**T**	**T**	**T**	**T**	**T**	**T**	**T**	**T**	**T**	**T**	**T**	**T**	**T**	**T**	**T**	**T**
**Cluster**	**2**	**2**	**2**	**2**	**2**	**2**	**8**	**2**	**2**	**2**	**2**	**2**	**2**	**2**	**2**	**2**	**2**	**2**	**2**	**2**	**2**	**2**	**4**	**2**	**2**	**2**	**2**	**2**	**2**
**Bioregion**	**S**	**S**	**S**	**S**	**S**	**S**	**S**	**T**	**T**	**T**	**T**	**T**	**T**	**T**	**T**	**T**	**T**	**T**	**T**	**T**	**T**	**T**	**T**	**T**	**T**	**T**	**T**	**T**	**T**
**Longitude**	**-70.28**	**-70**	**-70.28**	**-70.33**	**-70.45**	**-70.36**	**-70.32**	**-69.53**	**-70.43**	**-70.5**	**-70.8**	**-70.11**	**-70.24**	**-70.13**	**-70.22**	**-70.02**	**-70.22**	**-70.24**	**-70.01**	**-70.08**	**-70.3**	**-70.15**	**-70.26**	**-70.16**	**-70**	**-70.27**	**-70.27**	**-70.28**	**-70.25**
**Latitude**	**-33.82**	**-33.62**	**-33.3**	**-33.3**	**-33.3**	**-33.3**	**-33.3**	**-32.05**	**-31.43**	**-31.45**	**-31.27**	**-31.27**	**-31.21**	**-31.21**	**-31.19**	**-31.18**	**-31.17**	**-31.14**	**-31.54**	**-31.13**	**-31.05**	**-31.03**	**-31.09**	**-31.02**	**-31**	**-30.95**	**-30.95**	**-30.9**	**-30.88**
Acaena antarctica	.	.	.	.	.	.	.	.	.	.	.	.	.	.	.	.	.	.	.	.	.	.	.	.	.	.	.	.	.
Acaena macrocephala	.	.	.	.	.	.	.	.	.	.	.	.	.	.	.	.	.	.	.	.	.	.	.	.	.	.	.	.	.
Acaena magellanica	1	1	1	.	.	.	.	1	.	.	.	.	.	.	.	.	.	.	1	.	.	.	.	.	.	.	.	.	.
Acaena ovalifolia	.	.	.	.	.	.	.	.	.	.	.	.	.	.	.	.	.	.	.	.	.	.	.	.	.	.	.	.	.
Acaena pinnatifida	.	.	.	.	.	.	.	.	.	.	.	.	.	.	.	.	.	.	1	.	.	.	.	.	.	.	.	.	.
Adesmia retusa	.	.	.	.	.	.	.	.	.	.	.	.	.	.	.	.	.	.	.	.	.	.	.	.	.	.	.	.	.
Agrostis breviculmis	.	.	1	1	.	.	1	.	.	.	.	.	.	.	.	.	.	.	.	.	.	.	.	.	.	.	.	.	.
Agrostis imberbis	.	.	.	.	.	.	.	.	.	.	.	.	.	.	.	.	.	.	.	.	.	.	.	.	.	.	.	.	.
Agrostis meyenii	.	.	.	.	.	.	.	.	.	.	.	.	.	.	.	.	.	.	.	.	.	.	.	.	.	.	.	.	.
Agrostis perennans	.	.	.	.	.	.	.	.	1	.	.	.	.	.	.	.	.	.	.	.	.	.	.	.	.	.	.	.	.
Alchemilla pinnata	.	.	.	.	.	.	.	.	.	.	.	.	.	.	.	.	.	.	.	.	.	.	.	.	.	.	.	.	.
Alopecurus magellanicus	.	.	.	.	.	.	.	.	.	.	.	.	.	.	.	.	.	.	.	.	.	.	.	.	.	.	.	.	.
Amphiscirpus nevadensis	.	.	.	.	.	.	.	.	.	.	.	.	.	.	.	.	.	.	.	.	.	.	.	.	.	.	.	.	.
Anagallis alternifolia	1	.	.	.	1	1	.	.	.	.	.	.	.	.	.	.	.	.	.	.	.	.	.	.	.	.	.	.	.
Antennaria chilensis	.	.	1	.	.	.	1	.	.	.	.	.	.	.	.	.	.	.	.	.	.	.	.	.	.	.	.	.	.
Anthoxanthum redolens	.	.	.	.	.	.	.	.	.	.	.	.	.	.	.	.	.	.	.	.	.	.	.	.	.	.	.	.	.
Apium panul	1	.	.	.	.	.	.	.	.	.	.	.	.	.	.	.	.	.	.	.	.	.	.	.	.	.	.	.	.
Arenaria rivularis	.	.	.	.	.	.	.	1	.	.	.	.	.	.	.	.	.	.	.	.	.	.	.	.	.	.	.	.	.
Arenaria serpens	1	1	1	.	.	1	.	.	1	1	.	.	.	.	.	.	.	.	.	.	.	1	1	.	1	.	1	1	1
Arjona pusilla	.	.	.	.	.	.	.	.	.	.	1	1	.	.	.	1	.	.	.	.	.	.	.	.	.	.	.	.	.
Astragalus bustillosii	.	.	.	.	.	.	.	.	.	.	.	.	.	.	.	.	.	.	.	.	.	.	1	.	.	.	.	.	.
Astragalus micranthellus	.	.	.	.	.	.	.	.	.	.	.	.	.	.	.	.	.	.	.	.	.	.	.	.	.	.	.	.	.
Azolla filiculoides	.	.	.	.	.	.	.	.	.	.	.	.	.	.	.	.	.	.	.	.	.	.	.	.	.	.	.	.	.
Azorella boelckei	.	.	.	.	.	.	.	.	.	.	.	.	.	.	.	.	.	.	.	.	.	.	.	.	.	.	.	.	.

**Bog**	**349**	**350**	**351**	**352**	**353**	**354**	**355**	**356**	**357**	**358**	**359**	**360**	**361**	**362**	**363**	**364**	**365**	**366**	**367**	**368**	**369**	**370**	**371**	**372**	**373**	**374**	**375**	**376**	**377**
Azorella burkartii	.	.	.	.	.	.	.	.	.	.	.	.	.	.	.	.	.	.	.	.	.	.	.	.	.	.	.	.	.
Azorella cryptantha	.	.	.	.	.	.	.	.	.	.	.	.	.	.	.	.	.	.	.	.	.	.	.	.	.	.	.	.	.
Azorella lycopodioides	.	.	.	.	.	.	.	.	.	.	.	.	.	.	.	.	.	.	.	.	.	.	.	.	.	.	.	.	.
Azorella trifoliolata	.	.	1	.	1	.	.	.	.	.	.	.	.	.	.	.	.	.	.	.	.	.	.	.	.	1	.	.	.
Baccharis acaulis	.	.	.	.	.	.	.	.	.	.	.	.	.	.	.	.	.	.	.	.	.	.	.	.	.	.	.	.	.
Baccharis caespitosa	.	.	.	.	.	.	.	.	.	.	.	.	.	.	.	.	.	.	.	.	.	.	.	.	.	.	.	.	.
Baccharis magellanica	.	.	.	.	.	.	.	.	.	.	.	.	.	.	.	.	.	.	.	.	.	.	.	.	.	.	.	.	.
Belloa chilensis	.	.	.	.	.	.	.	.	.	.	.	.	.	.	.	.	.	.	.	.	.	.	.	.	.	.	.	.	.
Bromus catharticus	.	.	.	.	.	.	.	.	.	.	.	.	.	.	.	.	.	.	.	.	.	.	.	.	.	.	.	.	.
Calandrinia acaulis	.	.	.	.	.	.	.	.	.	.	.	.	.	.	.	.	.	.	.	.	.	.	.	.	.	.	.	.	.
Calandrinia compacta	.	.	.	.	.	.	.	.	.	.	.	.	.	.	.	.	.	.	.	.	.	1	1	1	.	.	.	.	1
Calceolaria biflora	1	1	1	.	1	1	.	.	.	.	.	.	.	.	.	.	.	.	.	.	.	.	.	.	.	.	.	.	.
Calceolaria cana	.	.	.	.	.	.	.	.	.	.	.	.	.	.	.	.	.	.	.	.	.	.	.	.	.	.	.	.	.
Calceolaria corymbosa	.	.	.	.	.	.	.	.	.	.	.	.	.	.	.	.	.	.	.	.	.	.	.	.	.	.	.	.	.
Calceolaria filicaulis	.	.	.	.	.	.	.	1	.	1	.	1	.	1	.	.	.	1	1	.	.	.	.	.	.	1	1	.	1
Callitriche lechleri	.	.	.	.	.	.	.	.	1	.	.	.	.	.	.	.	.	.	.	.	.	.	.	.	1	.	.	.	.
Caltha appendiculata	.	.	.	.	.	.	.	.	.	.	.	.	.	.	.	.	.	.	.	.	.	.	.	.	.	.	.	.	.
Caltha sagittata	.	.	1	.	.	1	.	.	.	.	.	.	.	.	.	.	.	.	.	.	.	.	.	.	.	1	.	.	.
Cardamine cordata	.	.	.	.	.	.	.	.	.	.	.	.	.	.	.	.	.	.	.	.	.	.	.	.	.	.	.	.	.
Cardamine glacialis	.	.	.	.	.	.	.	.	.	.	.	.	.	.	.	.	.	.	.	.	.	.	.	.	.	.	.	.	.
Cardamine tenuirostris	1	1	1	.	1	1	.	.	.	.	.	.	.	.	.	.	.	.	.	.	.	.	.	.	.	.	.	.	.
Cardamine volckmannii	.	.	.	.	.	.	.	.	.	.	.	.	.	.	.	.	.	.	.	.	.	.	.	.	.	.	.	.	.
Carex acaulis	.	.	.	.	.	.	.	.	.	.	.	.	.	.	.	.	.	.	.	.	.	.	.	.	.	.	.	.	.
Carex atropicta	.	.	.	.	.	.	.	.	.	.	.	.	.	.	.	.	.	.	.	.	.	.	.	.	.	.	.	.	.
Carex banksii	.	.	.	.	.	.	.	.	.	.	.	.	.	.	.	.	.	.	.	.	.	.	.	.	.	.	.	.	.
Carex caduca	.	.	.	.	.	.	.	.	.	.	.	.	.	.	.	.	.	.	.	.	.	.	.	.	.	.	.	.	.
Carex decidua	.	.	.	.	.	.	.	.	.	.	.	.	.	.	.	.	.	.	.	.	.	.	.	.	.	.	.	.	.
Carex fuscula	.	.	1	.	.	.	.	.	.	.	.	.	.	.	.	.	.	.	.	.	.	.	.	.	.	.	.	.	.
Carex gayana	1	1	1	1	1	1	.	1	1	1	1	1	1	1	.	1	1	1	1	1	1	.	1	1	1	1	1	1	1
Carex hypoleucos	.	.	.	.	.	.	.	.	.	.	.	.	.	.	.	.	.	.	.	.	.	.	.	.	.	.	.	.	.
Carex macloviana	.	.	1	1	.	.	1	.	.	.	.	.	.	1	1	.	1	1	.	1	.	1	.	1	.	.	.	.	.
Carex magellanica	.	.	.	.	.	.	.	.	.	.	.	.	.	.	.	.	.	.	.	.	.	.	.	.	.	.	.	.	.
Carex malmei	.	.	.	.	.	.	.	.	.	.	.	1	.	1	.	.	1	1	.	.	.	.	.	.	.	.	.	.	.
Carex maritima	.	.	1	.	.	.	.	.	.	1	.	1	.	1	1	.	.	.	.	1	.	.	1	1	1	.	.	.	.
Carex microglochin	.	.	.	.	.	.	.	.	.	.	.	.	.	.	.	.	.	.	.	.	.	.	.	.	.	.	.	.	.
Carex pleioneura	.	.	.	.	.	.	.	.	.	.	.	.	.	.	.	.	.	.	.	.	.	.	.	.	.	.	.	.	.
Carex ruthsatzae	.	.	.	.	.	.	.	.	.	.	.	.	.	.	.	.	.	.	.	.	.	.	.	.	.	.	.	.	.

Bog	349	350	351	352	353	354	355	356	357	358	359	360	361	362	363	364	365	366	367	368	369	370	371	372	373	374	375	376	377
Carex vallis-pulchrae	.	1	.	.	.	.	.	1	.	.	1	1	.	.	.	.	1	.	1	.	.	.	.	.	.	1	.	.	.
Carpha schoenoides	.	.	.	.	.	.	.	.	.	.	.	.	.	.	.	.	.	.	.	.	.	.	.	.	.	.	.	.	.
Castilleja pumila	.	.	.	.	.	.	.	.	.	.	.	.	.	.	.	.	.	.	.	.	.	.	.	.	.	.	.	.	.
Catabrosa werdermannii	.	.	.	.	.	.	.	.	1	.	.	.	.	.	.	.	.	.	.	.	.	.	.	.	.	.	.	.	.
Cerastium humifusum	.	.	.	.	.	.	.	.	.	.	.	.	.	.	.	.	.	.	1	.	.	.	.	.	.	1	1	1	1
Cerastium montioides	1	1	1	.	1	1	.	.	.	.	.	.	.	.	.	.	.	.	.	.	.	.	.	.	.	.	.	.	.
Chiliotrichum diffusum	.	.	.	.	.	.	.	.	.	.	.	.	.	.	.	.	.	.	.	.	.	.	.	.	.	.	.	.	.
Chusquea culeou	.	.	.	.	.	.	.	.	.	.	.	.	.	.	.	.	.	.	.	.	.	.	.	.	.	.	.	.	.
Colobanthus quitensis	1	1	1	1	.	1	1	.	1	.	.	.	.	1	1	1	.	1	.	1	.	.	.	.	.	.	1	1	1
Cortaderia egmontiana	.	.	.	.	.	.	.	.	.	.	.	.	.	.	.	.	.	.	.	.	.	.	.	.	.	.	.	.	.
Cotula mexicana	.	.	.	.	.	.	.	.	.	.	.	.	.	.	.	.	.	.	.	.	.	.	.	.	.	.	.	.	.
Crassula peduncularis	.	.	.	.	.	.	1	.	.	.	.	.	.	.	.	.	.	.	.	.	.	.	.	.	.	.	.	.	.
Cuatrecasasiella argentina	.	.	.	.	.	.	.	.	.	.	.	.	.	.	.	.	.	.	.	.	.	.	.	.	.	.	.	.	.
Deschampsia antarctica	.	.	.	.	.	.	.	.	.	.	.	.	.	.	.	.	.	.	.	.	.	.	.	.	.	.	.	.	.
Deschampsia caespitosa	1	.	.	.	.	.	.	.	.	.	.	.	.	.	.	.	.	.	1	.	.	.	.	.	.	.	.	.	.
Deschampsia patula	.	.	.	.	.	.	.	.	.	.	.	.	.	.	.	.	.	.	.	.	.	.	.	.	.	.	.	.	.
Cinnagrostis brevifolia	.	.	.	.	.	.	.	.	.	.	.	.	.	.	.	.	.	.	.	.	.	.	.	.	.	.	.	.	.
Deschampsia chrysantha	.	.	.	.	.	.	.	.	.	.	.	.	.	.	.	.	.	.	.	.	.	.	.	.	.	.	.	.	.
Cinnagrostis chrysophylla	.	.	.	.	.	.	.	.	.	.	.	.	.	.	.	.	.	.	.	.	.	.	.	.	.	.	.	.	.
Deschampsia chrysostachya	.	.	.	.	.	.	.	.	.	1	.	.	.	.	1	.	.	.	.	.	1	1	.	.	1	.	1	1	1
Deschampsia eminens	.	.	.	.	.	.	.	1	.	.	1	.	1	1	1	.	1	1	1	1	.	1	1	1	.	1	1	1	1
Deschampsia hackelii	.	.	.	.	.	.	.	.	.	.	.	.	.	.	.	.	.	.	.	.	.	.	.	.	.	.	.	.	.
Cinnagrostis minima	.	.	.	.	.	.	.	.	.	.	.	.	.	.	.	.	.	.	.	.	.	.	.	.	.	.	.	.	.
Deschampsia ovata	.	.	.	.	.	.	.	.	.	.	.	.	.	.	.	.	.	.	.	.	.	.	.	.	.	.	.	.	.
Cinnagrostis rigescens	.	.	.	.	.	.	.	.	.	.	.	.	.	.	.	.	.	.	.	.	.	.	.	.	.	.	.	.	.
Cinnagrostis spicigera	.	.	.	.	.	.	.	.	.	.	.	.	.	.	.	.	.	.	.	.	.	.	.	.	.	.	.	.	.
Cinnagrostis velutina	1	.	1	1	.	.	1	.	1	1	1	1	1	1	.	.	1	1	1	1	1	1	1	1	1	1	1	1	1
Cinnagrostis vicunarum	.	.	.	.	.	.	.	.	.	.	.	.	.	.	.	.	.	.	.	.	.	.	.	.	.	.	.	.	.
Distichia filamentosa	.	.	.	.	.	.	.	.	.	.	.	.	.	.	.	.	.	.	.	.	.	.	.	.	.	.	.	.	.
Distichia muscoides	.	.	.	.	.	.	.	.	.	.	.	.	.	.	.	.	.	.	.	.	.	.	.	.	.	.	.	.	.
Distichlis humilis	.	.	.	.	.	.	.	.	.	.	.	.	.	.	.	.	.	.	.	.	.	.	.	.	.	.	.	.	.

Bog	349	350	351	352	353	354	355	356	357	358	359	360	361	362	363	364	365	366	367	368	369	370	371	372	373	374	375	376	377
Distichlis scoparia	.	.	.	.	.	.	.	.	.	.	.	.	.	.	.	.	.	.	.	.	.	.	.	.	.	.	.	.	.
Distichlis spicata	.	.	.	.	.	.	.	.	.	.	.	.	.	.	.	.	.	.	.	.	.	.	.	.	.	.	.	.	.
Draba pusilla	.	.	1	.	.	.	1	.	.	.	.	.	.	.	.	.	.	.	.	.	.	.	.	.	.	.	1	.	.
Eleocharis melanomphala	.	.	.	.	.	.	.	.	.	.	.	.	.	.	.	.	.	.	.	.	.	.	.	.	.	.	.	.	.
Eleocharis pseudoalbibracteata	.	.	.	.	.	.	.	.	1	1	1	1	1	1	.	1	1	.	1	1	.	.	.	.	.	1	1	.	1
Elodea potamogeton	.	.	.	.	.	.	.	.	.	.	.	.	.	.	.	.	.	.	.	.	.	.	.	.	.	.	.	.	.
Empetrum rubrum	.	.	.	.	.	.	.	.	.	.	.	.	.	.	.	.	.	.	.	.	.	.	.	.	.	.	.	.	.
Epilobium australe	.	.	.	.	.	.	.	.	.	.	.	.	.	.	.	.	.	.	.	.	.	.	.	.	.	.	.	.	.
Epilobium barbeyanum	1	.	.	.	.	.	.	.	.	.	.	.	.	.	.	.	.	.	.	.	.	.	.	.	.	.	.	.	.
Epilobium ciliatum	1	.	.	.	.	.	.	.	.	.	.	.	.	.	.	.	.	.	.	.	.	.	.	.	.	.	.	.	.
Epilobium denticulatum	.	.	.	.	.	.	.	.	.	.	.	.	.	.	.	.	.	.	.	.	.	.	.	.	.	.	.	.	.
Epilobium fragile	.	.	.	.	.	.	.	.	.	.	.	.	.	.	.	.	.	.	.	.	.	.	.	.	.	.	.	.	.
Epilobium glaucum	1	1	.	.	.	.	.	.	.	.	.	.	.	.	.	.	.	.	.	.	.	.	.	.	.	.	.	.	.
Epilobium nivale	1	.	1	.	.	1	.	.	.	.	.	.	.	.	.	.	.	.	.	.	.	.	.	.	.	.	.	.	.
Erigeron andicola	.	.	1	.	.	.	1	.	.	.	.	.	.	.	.	.	.	.	.	.	.	.	.	.	.	.	.	.	.
Erigeron leptopetalus	.	.	.	.	.	.	.	1	1	.	.	.	.	.	.	.	.	.	1	.	1	.	.	.	.	.	1	.	1
Erigeron myosotis	.	.	.	.	.	.	.	.	.	.	.	.	.	.	.	.	.	.	.	.	.	.	.	.	.	.	.	.	.
Erigeron patagonicus	.	.	.	.	.	.	.	.	.	.	.	.	.	.	.	.	.	.	.	.	.	.	.	.	.	.	.	.	.
Erythranthe cuprea	.	.	.	.	.	.	.	.	.	.	.	.	.	.	.	.	.	.	.	.	.	.	.	.	.	.	.	.	.
Erythranthe depressa	.	.	.	.	.	.	.	.	1	1	.	1	.	.	.	.	1	.	1	1	.	.	.	1	.	1	1	1	1
Erythranthe glabrata	.	.	.	.	.	.	.	.	.	.	.	.	.	1	.	.	.	.	.	.	.	.	.	.	.	.	.	.	.
Erythranthe lutea	1	.	.	.	.	1	.	1	.	.	.	.	.	1	1	.	.	1	1	.	.	.	.	.	.	.	.	.	.
Escallonia virgata	.	.	.	.	.	.	.	.	.	.	.	.	.	.	.	.	.	.	.	.	.	.	.	.	.	.	.	.	.
Euphrasia antarctica	.	.	.	.	.	.	.	.	1	.	.	.	.	.	.	.	.	.	.	.	.	.	.	.	.	.	.	1	.
Euphrasia chrysantha	.	.	.	.	.	.	.	.	.	.	.	.	.	.	.	.	.	.	.	.	.	.	.	.	.	.	.	.	.
Euphrasia subexserta	.	.	.	.	.	.	.	.	.	.	.	.	.	.	.	.	.	.	.	.	.	.	.	.	.	.	.	.	.
Festuca hypsophila	.	.	.	.	.	.	.	.	.	.	.	.	.	.	.	.	.	.	.	.	.	.	.	.	.	.	.	.	.
Festuca kurtziana	1	1	.	.	.	.	.	.	.	.	.	.	.	.	.	.	.	.	.	.	.	.	.	.	.	.	.	.	.
Festuca lilloi	.	.	.	.	.	.	.	.	.	.	.	.	.	.	.	.	.	.	.	.	.	.	.	.	.	.	.	.	.
Festuca magellanica	1	.	1	1	.	1	1	.	.	.	.	.	.	.	.	.	.	.	.	.	.	.	.	.	.	.	.	.	.
Festuca nardifolia	.	.	.	.	.	.	.	.	.	.	1	1	1	.	.	.	1	1	.	.	.	.	.	.	.	1	1	1	.
Festuca rigescens	.	.	.	.	.	.	.	.	.	.	.	.	.	.	.	.	.	.	.	.	.	.	.	.	.	.	.	.	.
Festuca werdermannii	.	.	.	.	.	.	.	.	.	.	.	.	.	.	.	.	.	.	.	.	.	.	.	.	.	.	.	.	.
Frankenia triandra	.	.	.	.	.	.	.	.	.	.	.	.	.	.	.	.	.	.	.	.	.	.	.	.	.	.	.	.	.
Gamocarpha graminea	.	.	.	.	.	.	.	.	.	.	.	.	.	.	.	.	.	.	.	.	.	.	.	.	.	.	.	.	.

Bog	349	350	351	352	353	354	355	356	357	358	359	360	361	362	363	364	365	366	367	368	369	370	371	372	373	374	375	376	377
Gamocarpha ventosa	.	.	.	.	.	.	.	.	.	.	.	.	.	.	.	.	.	.	.	.	.	.	.	.	.	.	.	.	.
Gamochaeta chamissonis	.	.	.	.	.	.	.	.	.	.	.	.	.	.	.	.	.	.	.	.	.	.	.	.	.	.	.	.	.
Gamochaeta longipedicellata	.	.	.	.	.	.	.	.	.	.	.	.	.	.	.	.	.	.	.	.	.	.	.	.	.	.	.	.	.
Gamochaeta neuquensis	.	.	.	.	.	.	.	.	.	.	.	.	.	.	.	.	.	.	.	.	.	.	.	.	.	.	.	.	.
Gaultheria antarctica	.	.	.	.	.	.	.	.	.	.	.	.	.	.	.	.	.	.	.	.	.	.	.	.	.	.	.	.	.
Gaultheria caespitosa	.	.	.	.	.	.	.	.	.	.	.	.	.	.	.	.	.	.	.	.	.	.	.	.	.	.	.	.	.
Gaultheria pumila	.	.	.	.	.	.	.	.	.	.	.	.	.	.	.	.	.	.	.	.	.	.	.	.	.	.	.	.	.
Gavilea chica	.	.	.	.	.	.	.	.	.	.	.	.	.	.	.	.	.	.	.	.	.	.	.	.	.	.	.	.	.
Gentiana prostrata	1	.	1	1	.	1	.	1	1	1	.	1	1	1	1	1	1	1	1	1	1	1	.	1	1	1	1	1	1
Gentianella fiebrigii	.	.	.	.	.	.	.	.	.	.	.	.	.	.	.	.	.	.	.	.	.	.	.	.	.	.	.	.	.
Gentianella magellanica	1	.	.	.	.	.	.	.	.	.	.	.	.	.	.	.	.	.	.	.	.	.	.	.	.	.	.	.	.
Gentianella multicaulis	.	.	.	.	.	.	.	1	.	.	.	.	.	.	.	1	.	.	1	.	.	.	.	.	.	.	.	.	.
Gentianella ottonis	1	.	.	.	.	.	.	.	.	.	.	.	.	.	.	.	.	.	.	.	.	.	.	.	.	.	.	.	.
Gentianella primuloides	.	.	.	.	.	.	.	.	.	.	.	.	.	.	.	.	.	.	.	.	.	.	.	.	.	.	.	.	.
Gentianella pseudocrassula	.	.	.	.	.	.	.	.	.	.	.	.	.	.	.	.	.	.	.	.	.	.	.	.	.	.	.	1	1
Geranium sessiliflorum	.	.	.	.	.	.	.	.	.	.	.	.	.	.	.	.	.	.	.	.	.	.	.	.	.	.	.	.	.
Gunnera magellanica	.	.	.	.	.	.	.	.	.	.	.	.	.	.	.	.	.	.	.	.	.	.	.	.	.	.	.	.	.
Halenia caespitosa	.	.	.	.	.	.	.	.	.	.	.	.	.	.	.	.	.	.	.	.	.	.	.	.	.	.	.	.	.
Halerpestes cymbalaria	1	1	.	.	.	.	.	.	1	.	.	.	.	.	.	.	.	.	.	.	.	.	.	.	.	.	.	.	.
Halerpestes exilis	.	.	.	.	.	.	.	.	.	.	.	.	.	.	.	.	.	.	.	.	.	.	.	.	.	.	.	.	.
Hieracium antarcticum	.	.	.	.	.	.	.	.	.	.	.	.	.	.	.	.	.	.	.	.	.	.	.	.	.	.	.	.	.
Hordeum comosum	.	.	.	.	.	.	.	.	.	.	.	.	.	.	.	.	.	.	.	.	.	.	.	.	.	.	.	.	.
Hordeum muticum	.	.	.	.	.	.	.	.	.	.	.	.	.	.	.	.	.	.	.	.	.	.	.	.	.	.	.	.	.
Hypochaeris acaulis	1	.	.	.	.	.	.	.	.	.	.	.	.	.	.	.	.	.	.	.	.	.	.	.	.	.	.	.	.
Hypochaeris chondrilloides	.	.	.	.	.	.	.	.	.	.	.	.	.	.	.	.	.	.	.	.	.	.	.	.	.	.	.	.	.
Hypochaeris meyeniana	.	.	.	.	.	.	.	.	.	.	.	.	.	.	.	.	.	.	.	.	.	.	.	.	.	.	.	.	.
Hypochaeris palustris	.	.	.	.	.	.	.	.	.	.	.	.	.	.	.	.	.	.	.	.	.	.	.	.	.	.	.	.	.
Hypochaeris taraxacoides	.	.	.	.	.	.	.	.	.	.	.	.	.	.	.	.	.	.	.	.	.	.	.	.	.	.	.	.	.
Hypochaeris tenerifolia	.	.	.	.	.	.	.	.	.	.	.	.	.	.	.	.	.	.	.	.	.	.	.	.	.	.	.	.	.
Isolepis nigricans	.	.	.	.	.	.	.	.	.	.	.	.	.	.	.	.	.	.	.	.	.	.	.	.	.	.	.	.	.

Bog	349	350	351	352	353	354	355	356	357	358	359	360	361	362	363
Isolepsis inundata	.	.	.	.	.	.	.	.	.	.	.	.	.	.	.
Juncus balticus	1	1	.	.	.	.	.	1	.	.	1	1	.	.	.
Juncus stipulatus	1	1	1	1	1	1	1	.	1	.	.	.	1	1	1
Koeleria kurtzii	.	.	.	.	.	.	.	.	1	.	.	.	.	.	.
Lachemilla diplophylla	.	.	.	.	.	.	.	.	.	.	.	.	.	.	.
Lachemilla pinnata	.	.	.	.	.	.	.	.	.	.	.	.	.	.	.
Lagenophora nudicaulis	.	.	.	.	.	.	.	.	.	.	.	.	.	.	.
Lemna minuta	.	.	.	.	.	.	.	.	.	.	.	.	.	.	.
Leptinella scariosa	.	.	.	.	.	.	.	.	.	.	.	.	.	.	.
Leucheria candidissima	.	.	.	.	.	.	.	.	.	.	.	.	.	.	.
Leucheria nutans	.	.	.	.	.	.	.	.	.	.	.	.	.	.	.
Lilaea scilloides	.	.	.	.	.	.	.	.	.	.	.	.	.	.	.
Lilaeopsis macloviana	.	.	.	.	1	1	.	.	.	.	.	.	.	.	.
Limosella australis	.	.	.	.	.	.	1	.	.	.	.	.	.	.	.
Lobelia oligophylla	1	1	1	.	1	1	.	1	1	1	1	1	.	.	.
Luzula brachyphylla	.	.	.	1	.	.	.	.	.	.	.	.	.	.	.
Luzula chilensis	.	.	1	.	.	.	1	.	.	.	.	.	.	.	.
Luzula racemosa	.	.	.	.	.	.	.	.	.	.	.	.	.	.	.
Luzula vulcanica	.	.	.	.	.	.	.	.	.	.	.	.	.	.	.
Lysipomia pumila	.	.	.	.	.	.	.	.	.	.	.	.	.	.	.
Marsippospermum philippii	.	.	.	.	.	.	.	.	.	.	.	.	.	.	.
Marsippospermum reichei	.	.	.	.	.	.	.	.	.	.	.	.	.	.	.
Montia fontana	.	.	.	.	.	.	.	.	.	.	.	.	.	.	.
Muhlenbergia asperifolia	.	.	.	.	.	.	.	.	.	.	.	.	.	.	.
Myriophyllum quitense	1	.	.	.	.	.	1	.	1	.	.	.	1	.	.
Myrosmodes nervosa	.	.	.	.	.	.	.	.	.	.	.	.	.	.	.
Myrosmodes paludosa	.	.	.	.	.	.	.	.	.	.	.	.	.	.	.
Myrteola nummularia	.	.	.	.	.	.	.	.	.	.	.	.	.	.	.
Nanodea muscosa	.	.	.	.	.	.	.	.	.	.	.	.	.	.	.
Neobartsia crenoloba	.	.	.	.	.	.	.	.	.	.	.	.	.	.	.
Neobartsia pedicularoides	.	.	.	.	.	.	.	.	.	.	.	.	.	.	.
Neobartsia peruviana	.	.	.	.	.	.	.	.	.	.	.	.	.	.	.

Bog	364	365	366	367	368	369	370	371	372	373	374	375	376	377
Isolepsis inundata	.	.	.	.	.	.	.	.	.	.	.	.	.	.
Juncus balticus	1	.	.	1	.	.	.	1	.	.	1	.	.	.
Juncus stipulatus	.	1	1	1	1	.	.	.	.	.	1	1	.	1
Koeleria kurtzii	.	.	.	.	.	.	.	.	.	.	.	.	.	.
Lachemilla diplophylla	.	.	.	.	.	.	.	.	.	.	.	.	.	.
Lachemilla pinnata	.	.	.	.	.	.	.	.	.	.	.	.	.	.
Lagenophora nudicaulis	.	.	.	.	.	.	.	.	.	.	.	.	.	.
Lemna minuta	.	.	.	.	.	.	.	.	.	.	.	.	.	.
Leptinella scariosa	.	.	.	.	.	.	.	.	.	.	.	.	.	.
Leucheria candidissima	.	.	.	.	.	.	.	.	.	.	.	.	.	.
Leucheria nutans	.	.	.	.	.	.	.	.	.	.	.	.	.	.
Lilaea scilloides	.	.	.	.	.	.	.	.	.	.	.	.	.	.
Lilaeopsis macloviana	.	.	.	.	.	.	.	.	.	.	.	.	.	.
Limosella australis	.	.	.	.	.	.	.	.	.	.	.	1	.	1
Lobelia oligophylla	1	1	.	1	.	.	.	1	.	.	1	1	.	1
Luzula brachyphylla	.	.	.	.	.	.	.	.	.	.	.	.	.	.
Luzula chilensis	.	.	.	.	.	.	.	.	.	.	.	.	.	.
Luzula racemosa	.	.	.	.	.	.	.	.	.	.	.	.	.	.
Luzula vulcanica	.	.	.	.	.	.	.	.	.	.	.	.	.	.
Lysipomia pumila	.	.	.	.	.	.	.	.	.	.	.	.	.	.
Marsippospermum philippii	.	.	.	.	.	.	.	.	.	.	.	.	.	.
Marsippospermum reichei	.	.	.	.	.	.	.	.	.	.	.	.	.	.
Montia fontana	.	.	.	.	.	.	.	.	.	.	.	.	.	.
Muhlenbergia asperifolia	.	.	.	.	.	.	.	.	.	.	.	.	.	.
Myriophyllum quitense	1	.	.	.	.	1	1	.	.	.	.	1	.	1
Myrosmodes nervosa	.	.	.	.	.	.	.	.	.	.	.	.	.	.
Myrosmodes paludosa	.	.	.	.	.	.	.	.	.	.	.	.	.	.
Myrteola nummularia	.	.	.	.	.	.	.	.	.	.	.	.	.	.
Nanodea muscosa	.	.	.	.	.	.	.	.	.	.	.	.	.	.
Neobartsia crenoloba	.	.	.	.	.	.	.	.	.	.	.	.	.	.
Neobartsia pedicularoides	.	.	.	.	.	.	.	.	.	.	.	.	.	.
Neobartsia peruviana	.	.	.	.	.	.	.	.	.	.	.	.	.	.

**Bog**	349	350	351	352	353	354	355	356	357	358	359	360	361	362	363	364	365	366	367	368	369	370	371	372	373	374	375	376	377
Nertera granadensis	.	.	.	.	.	.	.	.	.	.	.	.	.	.	.	.	.	.	.	.	.	.	.	.	.	.	.	.	.
Nicoraepoa andina	.	.	.	.	.	.	.	.	.	.	.	.	.	.	.	.	.	.	.	.	.	.	.	.	.	.	.	.	.
Nicoraepoa pugionifolia	1	.	.	.	.	.	.	.	.	.	.	.	.	.	.	.	.	.	.	.	.	.	.	.	.	.	.	.	.
Nicoraepoa subenervis	.	.	.	.	.	.	.	.	.	.	.	.	.	.	.	.	.	.	1	.	.	.	.	.	.	.	.	.	.
Nitrophila australis	.	.	.	.	.	.	.	.	.	.	.	.	.	.	.	.	.	.	.	.	.	.	.	.	.	.	.	.	.
Nothofagus antarctica	.	.	.	.	.	.	.	.	.	.	.	.	.	.	.	.	.	.	.	.	.	.	.	.	.	.	.	.	.
Nototriche rugosa	.	.	.	.	.	.	.	.	.	.	.	.	.	.	.	.	.	.	.	.	.	.	.	.	.	.	.	.	.
Ochetophila nana	1	1	1	.	.	.	.	1	.	.	1	1	.	1	.	.	1	.	.	.	.	.	.	.	.	.	.	.	.
Olsynium junceum	.	.	.	.	.	.	.	.	.	.	.	.	.	.	.	.	.	.	.	.	.	.	.	.	.	.	.	.	.
Oreobolus obtusangulus	.	.	.	.	.	.	.	.	.	.	.	.	.	.	.	.	.	.	.	.	.	.	.	.	.	.	.	.	.
Oritrophium limnophilum	.	.	.	.	.	.	.	.	.	.	.	.	.	.	.	.	.	.	.	.	.	.	.	.	.	.	.	.	.
Osmorhiza glabrata	.	.	.	.	.	.	.	.	.	.	.	.	.	.	.	.	.	.	.	.	.	.	.	.	.	.	.	.	.
Ourisia alpina	.	.	.	.	.	.	.	.	.	.	.	.	.	.	.	.	.	.	.	.	.	.	.	.	.	.	.	.	.
Ourisia muscosa	.	.	.	.	.	.	.	.	.	.	.	.	.	.	.	.	.	.	.	.	.	.	.	.	.	.	.	.	.
Ourisia ruelloides	.	.	.	.	.	.	.	.	.	.	.	.	.	.	.	.	.	.	.	.	.	.	.	.	.	.	.	.	.
Oxychloe andina	.	1	1	.	.	.	1	.	.	.	1	1	.	.	.	1	.	.	.	.	.	.	.	.	.	.	.	.	.
Oxychloe bisexualis	.	.	.	.	.	.	.	.	.	.	.	1	.	.	.	.	.	.	.	.	.	.	.	.	.	.	.	.	.
Oxychloe castellanosii	.	.	.	.	.	.	.	.	.	.	.	.	.	.	.	.	.	.	.	.	.	.	.	.	.	.	.	.	.
Oxychloe haumaniana	.	.	.	.	.	.	.	.	.	.	.	.	.	.	.	1	.	.	.	.	.	.	.	.	.	.	.	.	.
Oxychloe mendocina	.	.	.	.	.	.	.	.	.	.	.	.	.	.	.	.	.	.	.	.	.	.	.	.	.	.	.	.	.
Patosia clandestina	.	.	1	1	.	1	.	1	1	1	.	1	1	1	1	.	1	1	1	1	1	1	1	1	1	1	1	1	1
Perezia capito	.	.	.	.	.	.	.	.	.	.	.	.	.	.	.	.	.	.	.	.	.	.	.	.	.	.	.	.	.
Perezia delicata	.	.	.	.	.	.	.	.	.	.	.	.	.	.	.	.	.	.	.	.	.	.	.	.	.	.	.	.	.
Perezia fonkii	.	.	.	.	.	.	.	.	.	.	.	.	.	.	.	.	.	.	.	.	.	.	.	.	.	.	.	.	.
Perezia pedicularidifolia	.	.	.	.	.	.	.	.	.	.	.	.	.	.	.	.	.	.	.	.	.	.	.	.	.	.	.	.	.
Perezia pinnatifida	.	.	.	.	.	.	.	.	.	.	.	.	.	.	.	.	.	.	.	.	.	.	.	.	.	.	.	.	.
Petroravenia friesii	.	.	.	.	.	.	.	.	.	.	.	.	.	.	.	.	.	.	.	.	.	.	.	.	.	.	.	.	.
Petroravenia werdermannii	.	.	.	.	.	.	.	.	.	.	.	.	.	.	.	.	.	.	.	.	.	.	.	.	.	.	.	.	.
Phleum alpinum	.	1	1	.	.	1	.	.	.	.	.	.	.	1	.	.	1	.	.	1	.	.	.	.	1	.	1	1	.
Phylloscirpus acaulis	1	1	1	.	1	1	.	1	.	.	1	1	.	.	.	.	1	.	1	.	.	.	.	.	.	1	.	.	.
Phylloscirpus boliviensis	.	.	.	.	.	.	.	.	.	.	.	.	.	.	.	.	.	.	.	.	.	.	.	.	.	.	.	.	.

Bog	349	350	351	352	353	354	355	356	357	358	359	360	361	362	363	364	365	366	367	368	369	370	371	372	373	374	375	376	377
Phylloscirpus deserticola	.	.	.	.	.	.	.	.	1	.	.	.	.	.	.	.	.	.	.	.	.	.	.	.	.	.	.	.	.
Pinguicula antarctica	.	.	.	.	.	.	.	.	.	.	.	.	.	.	.	.	.	.	.	.	.	.	.	.	.	.	.	.	.
Plantago barbata	1	1	1	1	.	1	1	1	1	1	1	1	1	1	1	1	.	1	1	1	1	.	1	1	1	1	1	1	1
Plantago rigida	.	.	.	.	.	.	.	.	.	.	.	.	.	.	.	.	.	.	.	.	.	.	.	.	.	.	.	.	.
Plantago tubulosa	.	.	.	.	.	.	.	.	.	.	.	.	.	.	.	.	.	.	.	.	.	.	.	.	.	.	.	.	.
Plantago uniglumis	.	.	.	.	.	.	.	.	.	.	.	.	.	.	.	.	.	.	.	.	.	.	.	.	.	.	.	.	1
Poa alopecurus	.	.	.	.	.	.	.	.	.	.	.	.	.	.	.	.	.	.	.	.	.	.	.	.	.	.	.	.	.
Poa hachadoensis	.	.	.	.	.	.	.	.	.	.	.	.	.	.	.	.	.	.	.	.	.	.	.	.	.	.	.	.	.
Poa perligulata	.	.	.	.	.	.	.	.	.	.	.	.	.	.	.	.	.	.	.	.	.	.	.	.	.	.	.	.	.
Polypogon interruptus	1	.	.	.	1	.	.	.	.	.	.	.	.	.	.	.	.	.	.	.	.	.	.	.	.	.	.	.	.
Primula magellanica	.	.	.	.	.	.	.	.	.	.	.	.	.	.	.	.	.	.	.	.	.	.	.	.	.	.	.	.	.
Puccinellia frigida	.	.	.	.	.	.	.	.	.	.	.	.	.	.	.	.	.	.	.	.	.	.	1	.	.	.	.	.	.
Quinchamalium chilense	.	.	.	.	.	.	.	.	.	.	.	.	.	.	.	.	.	.	.	.	.	.	.	.	.	.	.	.	.
Ranunculus breviscapus	.	.	.	.	.	.	.	.	.	.	.	.	.	.	.	.	.	.	.	.	.	.	.	.	.	.	.	.	.
Ranunculus fuegianus	.	.	.	.	.	.	.	.	1	.	.	.	.	.	.	1	.	.	.	.	.	.	.	.	.	.	.	1	.
Ranunculus mandonianus	.	.	.	.	.	.	.	.	.	.	.	.	.	.	.	.	.	.	.	.	.	.	.	.	.	.	.	.	.
Ranunculus peduncularis	.	.	1	.	1	.	.	.	.	.	.	.	.	.	.	.	.	.	.	.	.	.	.	.	.	.	.	.	.
Ranunculus trichophyllus	.	.	.	.	.	.	.	.	.	.	.	.	.	.	.	.	.	.	.	.	.	.	.	.	1	.	.	.	.
Halerpestes uniflora	.	.	.	.	.	.	.	.	.	.	.	.	.	.	.	.	.	.	.	.	.	.	.	.	.	.	.	.	.
Rubus geoides	.	.	.	.	.	.	.	.	.	.	.	.	.	.	.	.	.	.	.	.	.	.	.	.	.	.	.	.	.
Rumex magellanicus	.	.	.	.	.	.	.	.	.	.	.	.	.	.	.	.	.	.	.	.	.	.	.	.	.	.	.	.	.
Rytidosperma lechleri	.	.	.	.	.	.	.	.	.	.	.	.	.	.	.	.	.	.	.	.	.	.	.	.	.	.	.	.	.
Sarcocornia pulvinata	.	.	.	.	.	.	.	.	.	.	.	.	.	.	.	.	.	.	.	.	.	.	.	.	.	.	.	.	.
Schoenoplectus pungens	.	.	.	.	.	.	.	.	.	.	.	.	.	.	.	.	.	.	.	.	.	.	.	.	.	.	.	.	.
Schoenus andinus	.	.	.	.	.	.	.	.	.	.	.	.	.	.	.	.	.	.	.	.	.	.	.	.	.	.	.	.	.
Senecio breviscapus	.	.	.	.	.	.	.	.	.	.	.	.	.	.	.	.	.	.	1	.	.	.	.	.	.	1	.	.	.
Senecio diemii	.	.	.	.	.	.	.	.	.	.	.	.	.	.	.	.	.	.	.	.	.	.	.	.	.	.	.	.	.
Senecio fistulosus	.	.	.	.	.	.	.	.	.	.	.	.	.	.	.	.	.	.	.	.	.	.	.	.	.	.	.	.	.
Senecio parodii	.	.	.	.	.	.	.	.	.	.	.	.	.	.	.	.	.	.	.	.	.	.	.	.	.	.	.	.	.
Senecio peteroanus	.	.	.	.	.	.	.	.	.	.	.	.	.	.	.	.	.	.	.	.	.	.	.	.	.	.	.	.	.
Senecio serratifolius	.	.	.	.	.	.	.	.	.	.	.	.	.	.	.	.	.	.	.	.	.	.	.	.	.	.	.	.	.

**Bog**	**349**	**350**	**351**	**352**	**353**	**354**	**355**	**356**	**357**	**358**	**359**	**360**	**361**	**362**	**363**	**364**	**365**	**366**	**367**	**368**	**369**	**370**	**371**	**372**	**373**	**374**	**375**	**376**	**377**
Senecio trifurcatus	.	.	.	.	.	.	.	.	.	.	.	.	.	.	.	.	.	.	.	.	.	.	.	.	.	.	.	.	.
Sisyrinchium chilense	.	.	.	.	.	.	.	1	.	.	.	.	.	.	.	.	.	.	1	.	.	.	.	.	.	.	.	.	.
Sisyrinchium patagonicum	.	.	.	.	.	.	.	.	.	.	.	.	.	.	.	.	.	.	.	.	.	.	.	.	.	.	.	.	.
Sisyrinchium pearcei	.	.	.	.	.	.	.	.	.	.	.	.	.	.	.	.	.	.	.	.	.	.	.	.	.	.	.	.	.
Stellaria debilis	.	.	.	.	.	.	.	.	.	.	.	.	.	.	.	.	.	.	.	.	.	.	.	.	.	.	.	.	.
Stuckenia filiformis	1	.	.	.	.	.	.	.	1	.	1	.	.	.	.	1	.	.	.	.	.	.	1	.	1	.	.	.	.
Stuckenia striata	.	.	.	.	.	.	.	.	.	.	.	.	.	.	.	.	.	.	.	.	.	.	.	.	.	.	.	.	.
Symphyotrichum peteroanum	.	.	.	.	.	.	.	.	.	.	.	.	.	.	.	.	.	.	.	.	.	.	.	.	.	.	.	.	.
Symphyotrichum vahlii	.	.	.	.	.	.	.	.	.	.	.	.	.	.	.	.	.	.	.	.	.	.	.	.	.	.	.	.	.
Tetroncium magellanicum	.	.	.	.	.	.	.	.	.	.	.	.	.	.	.	.	.	.	.	.	.	.	.	.	.	.	.	.	.
Tribeles australis	.	.	.	.	.	.	.	.	.	.	.	.	.	.	.	.	.	.	.	.	.	.	.	.	.	.	.	.	.
Trifolium amabile	.	.	.	.	.	.	.	.	.	.	.	.	.	.	.	.	.	.	.	.	.	.	.	.	.	.	.	.	.
Trifolium polymorphum	.	.	.	.	1	1	.	.	.	.	.	.	.	.	.	.	.	.	.	.	.	.	.	.	.	.	.	.	.
Triglochin concinna	.	.	.	.	.	.	.	.	.	.	.	.	.	.	.	.	.	.	.	.	.	.	.	.	.	.	.	.	.
Triglochin palustris	1	1	.	.	.	.	.	.	.	.	1	1	1	.	.	.	.	.	.	.	.	.	1	.	.	.	.	.	.
Triglochin striata	.	.	.	.	.	.	.	.	.	.	.	.	.	.	.	.	.	.	.	.	.	.	.	.	.	.	.	.	.
Trisetum caudulatum	.	.	.	.	.	.	.	.	.	.	.	.	.	.	.	.	.	.	.	.	.	.	.	.	.	.	.	.	.
Trisetum preslei	.	.	1	.	.	.	.	.	.	.	.	.	.	.	.	.	.	.	.	.	.	.	.	.	.	.	.	.	.
Koeleria spicata	.	.	.	.	.	.	.	.	.	.	.	.	.	.	.	.	.	.	.	.	.	.	.	.	.	.	.	.	.
Utricularia gibba	.	.	.	.	.	.	.	.	.	.	.	.	.	.	.	.	.	.	.	.	.	.	.	.	.	.	.	.	.
Vahlodea atropurpurea	.	.	.	.	.	.	.	.	.	.	.	.	.	.	.	.	.	.	.	.	.	.	.	.	.	.	.	.	.
Valeriana fonckii	.	.	.	.	.	.	.	.	.	.	.	.	.	.	.	.	.	.	.	.	.	.	.	.	.	.	.	.	.
Valeriana macrorhiza	.	.	.	.	.	.	.	.	.	.	.	.	.	.	.	.	.	.	.	.	.	.	.	.	.	.	.	.	.
Viola pygmaea	.	.	.	.	.	.	.	.	.	.	.	.	.	.	.	.	.	.	.	.	.	.	.	.	.	.	.	.	.
Werneria apiculata	.	.	.	.	.	.	.	.	.	.	.	.	.	.	.	.	.	.	.	.	.	.	.	.	.	.	.	.	.
Werneria pinnatifida	.	.	.	.	.	.	.	.	.	.	.	.	.	.	1	.	1	.	.	.	.	.	.	.	.	.	.	.	.
Werneria pygmaea	1	1	1	1	.	1	.	1	1	1	1	1	.	1	.	1	.	1	1	1	.	.	.	.	1	1	1	1	1
Werneria solivifolia	.	.	.	.	.	.	.	.	.	.	.	.	.	.	.	.	.	.	.	.	.	.	.	.	.	.	.	.	.
Werneria spathulata	.	.	.	.	.	.	.	.	.	.	.	.	.	.	.	.	.	.	.	.	.	.	.	.	.	.	.	.	.
Xenophyllum incisum	.	.	.	.	.	.	.	.	.	.	.	.	.	.	.	.	.	.	.	.	.	.	.	.	.	.	.	.	.
Zameioscirpus atacamensis	.	.	.	.	.	.	.	.	.	.	.	.	.	.	.	.	.	.	.	.	.	.	1	.	.	.	.	.	.
Zameioscirpus gaimardiodes	.	.	.	.	.	.	.	.	.	.	1	.	.	.	.	.	.	.	.	.	.	.	.	.	.	.	.	.	.
Zameioscirpus muticus	.	.	.	.	.	.	.	.	1	1	.	1	1	1	1	1	1	1	.	1	1	1	.	1	1	1	1	1	1

**Bog**	**378**	**379**	**380**	**381**	**382**	**383**	**384**	**385**	**386**	**387**	**388**	**389**	**390**	**391**	**392**	**393**	**394**	**395**	**396**	**397**	**398**	**399**	**400**	**401**	**402**	**403**	**404**	**405**	**406**
**Operational zone**	**T**	**T**	**T**	**T**	**T**	**T**	**T**	**T**	**T**	**T**	**T**	**T**	**T**	**T**	**T**	**T**	**T**	**T**	**T**	**T**	**T**	**T**	**T**	**T**	**T**	**T**	**T**	**T**	**T**
**Cluster**	**2**	**2**	**2**	**2**	**2**	**2**	**2**	**2**	**2**	**2**	**2**	**2**	**2**	**2**	**4**	**4**	**4**	**4**	**4**	**4**	**2**	**2**	**2**	**2**	**4**	**4**	**4**	**4**	**4**
**Bioregion**	**T**	**T**	**T**	**T**	**T**	**T**	**T**	**T**	**T**	**T**	**T**	**T**	**T**	**T**	**T**	**T**	**T**	**T**	**T**	**T**	**T**	**T**	**T**	**T**	**T**	**T**	**T**	**T**	**T**
**Longitude**	**-70.01**	**-70.25**	**-70.22**	**-69.75**	**-69.73**	**-69.73**	**-69.75**	**-69.74**	**-69.77**	**-69.75**	**-69.78**	**-70.01**	**-69.88**	**-69.57**	**-69.72**	**-69.75**	**-69.72**	**-65.7**	**-69.8**	**-69.88**	**-69.82**	**-69.88**	**-70.02**	**-69.93**	**-69.85**	**-69.78**	**-69.87**	**-69.88**	**-69.86**
**Latitude**	**-31.54**	**-30.88**	**-30.87**	**-30.35**	**-30.34**	**-30.33**	**-30.33**	**-30.32**	**-30.32**	**-30.31**	**-30.3**	**-31.54**	**-30.16**	**-30.05**	**-29.97**	**-29.95**	**-29.83**	**-29.8**	**-29.78**	**-29.57**	**-29.45**	**-29.35**	**-31.5**	**-29.33**	**-29.28**	**-29.78**	**-29.5**	**-29.45**	**-29.42**
Acaena antarctica	.	.	.	.	.	.	.	.	.	.	.	.	.	.	.	.	.	.	.	.	.	.	.	.	.	.	.	.	.
Acaena macrocephala	.	.	.	.	.	.	.	.	.	.	.	.	.	.	.	.	.	.	.	.	.	.	.	.	.	.	.	.	.
Acaena magellanica	1	.	.	1	.	.	.	.	.	.	.	1	.	1	.	.	.	.	.	.	.	.	.	.	.	.	.	.	.
Acaena ovalifolia	.	.	.	.	.	.	.	.	.	.	.	.	.	.	.	.	.	.	.	.	.	.	.	.	.	.	.	.	.
Acaena pinnatifida	.	.	.	1	.	.	.	.	.	.	.	.	.	1	.	.	.	.	.	.	.	.	1	.	.	.	.	.	.
Adesmia retusa	.	.	.	.	.	.	.	.	.	.	.	.	.	.	.	.	.	.	.	.	.	.	.	.	.	.	.	.	.
Agrostis breviculmis	.	.	.	.	.	.	.	.	.	.	.	.	.	.	.	.	.	.	.	.	.	.	.	.	.	.	.	.	.
Agrostis imberbis	.	.	.	.	.	.	.	.	.	.	.	.	.	.	.	.	.	.	.	.	.	.	.	.	.	.	.	.	.
Agrostis meyenii	.	.	.	.	.	.	.	.	.	.	.	.	.	.	.	.	.	.	.	.	.	.	.	.	.	.	.	.	.
Agrostis perennans	.	.	.	.	.	.	.	.	.	.	.	.	.	.	.	.	.	.	.	.	.	.	.	.	.	.	.	.	.
Alchemilla pinnata	.	.	.	.	.	.	.	.	.	.	.	.	.	.	.	.	.	.	.	.	.	.	.	.	.	.	.	.	.
Alopecurus magellanicus	.	.	.	.	.	.	.	.	.	.	.	.	.	.	.	.	.	.	.	.	.	.	.	.	.	.	.	.	.
Amphiscirpus nevadensis	.	.	.	.	.	.	.	.	.	.	.	.	.	.	.	.	.	.	.	.	.	.	.	.	.	.	.	.	.
Anagallis alternifolia	.	.	.	.	.	.	.	.	.	.	.	.	.	.	.	.	.	.	.	.	.	.	.	.	.	.	.	.	.
Antennaria chilensis	.	.	.	.	.	.	.	.	.	.	.	.	.	.	.	.	.	.	.	.	.	.	.	.	.	.	.	.	.
Anthoxanthum redolens	.	.	.	.	.	.	.	.	.	.	.	.	.	.	.	.	.	.	.	.	.	.	.	.	.	.	.	.	.
Apium panul	.	.	.	.	.	.	.	.	.	.	.	.	.	.	.	.	.	.	.	.	.	.	.	.	.	.	.	.	.
Arenaria rivularis	.	.	.	.	.	.	.	.	.	.	.	.	.	.	.	1	.	.	.	.	.	.	.	.	.	.	.	.	.
Arenaria serpens	1	1	1	.	.	.	.	.	.	1	.	.	.	.	1	.	1	1	1	1	1	1	.	.	1	1	1	1	1
Arjona pusilla	.	.	.	.	.	.	.	.	.	.	.	.	.	.	.	.	.	.	.	.	.	.	.	.	.	.	.	.	.
Astragalus bustillosii	.	.	.	1	1	1	.	.	.	1	.	.	.	.	.	.	.	.	.	.	.	.	.	.	.	.	.	.	.
Astragalus micranthellus	.	.	.	.	.	.	.	.	.	.	.	.	.	.	.	.	.	.	.	.	.	.	.	.	.	.	.	.	.
Azolla filiculoides	.	.	.	.	.	.	.	.	.	.	.	.	.	.	.	.	.	.	.	.	.	.	.	.	.	.	.	.	.
Azorella boelckei	.	.	.	.	.	.	.	.	.	.	.	.	.	.	.	.	.	.	.	.	.	.	.	.	.	.	.	.	.

Bog	378	379	380	381	382	383	384	385	386	387	388	389	390	391	392	393	394	395	396	397	398	399	400	401	402	403	404	405	406
Azorella burkartii	.	.	.	.	.	.	.	.	.	.	.	.	.	.	.	.	.	.	.	.	.	.	.	.	.	.	.	.	.
Azorella cryptantha	.	.	.	1	.	.	1	.	1	.	.	.	1	.	1	.	.	.	.	.	.	.	.	.	.	1	.	.	.
Azorella lycopodioides	.	.	.	.	.	.	.	.	.	.	.	.	.	.	.	.	.	.	.	.	.	.	.	.	.	.	.	.	.
Azorella trifoliolata	.	.	.	.	.	.	.	.	.	.	.	.	.	.	.	.	.	.	.	.	.	.	.	.	.	.	.	.	.
Baccharis acaulis	.	.	.	.	.	.	.	.	.	.	.	.	.	.	.	.	.	.	.	.	.	.	.	.	.	.	.	.	.
Baccharis caespitosa	.	.	.	.	.	.	.	.	.	.	.	.	.	.	.	.	.	.	.	.	.	.	.	.	.	.	.	.	.
Baccharis magellanica	.	.	.	.	.	.	.	.	.	.	.	.	.	.	.	.	.	.	.	.	.	.	.	.	.	.	.	.	.
Belloa chilensis	.	.	.	.	.	.	.	.	.	.	.	.	.	.	.	.	.	.	.	.	.	.	.	.	.	.	.	.	.
Bromus catharticus	.	.	.	.	.	.	.	.	.	.	.	.	.	.	.	.	.	.	.	.	.	.	.	.	.	.	.	.	.
Calandrinia acaulis	.	.	.	.	.	.	.	.	.	.	.	.	.	.	.	.	.	.	.	.	.	.	.	.	.	.	.	.	.
Calandrinia compacta	.	.	.	1	1	.	.	.	1	1	1	.	1	.	1	1	1	1	.	1	.	.	.	1	.	1	.	.	.
Calceolaria biflora	.	.	.	.	.	.	.	.	.	.	.	.	.	.	.	.	.	.	.	.	.	.	.	.	.	.	.	.	.
Calceolaria cana	.	.	.	.	.	.	.	.	.	.	.	.	.	.	.	.	.	.	.	.	.	.	.	.	.	.	.	.	.
Calceolaria corymbosa	.	.	.	.	.	.	.	.	.	.	.	.	.	.	.	.	.	.	.	.	.	.	.	.	.	.	.	.	.
Calceolaria filicaulis	1	.	.	1	.	.	.	1	.	.	.	1	.	.	.	.	.	.	.	.	.	.	1	.	.	.	.	.	.
Callitriche lechleri	.	.	.	.	.	.	.	.	.	.	.	.	.	.	.	.	.	.	.	.	.	.	.	.	.	.	.	.	.
Caltha appendiculata	.	.	.	.	.	.	.	.	.	.	.	.	.	.	.	.	.	.	.	.	.	.	.	.	.	.	.	.	.
Caltha sagittata	.	1	.	.	.	.	.	.	.	.	.	.	.	.	.	.	.	.	.	.	.	.	.	.	.	.	.	.	.
Cardamine cordata	.	.	.	.	.	.	.	.	.	.	.	.	.	.	.	.	.	.	.	.	.	.	.	.	.	.	.	.	.
Cardamine glacialis	.	.	.	.	.	.	.	.	.	.	.	.	.	.	.	.	.	.	.	.	.	.	.	.	.	.	.	.	.
Cardamine tenuirostris	.	.	.	.	.	.	.	.	.	.	.	.	.	.	.	.	.	.	.	.	.	.	.	.	.	.	.	.	.
Cardamine volckmannii	.	.	.	.	.	.	.	.	.	.	.	.	.	.	.	.	.	.	.	.	.	.	.	.	.	.	.	.	.
Carex acaulis	.	.	.	.	.	.	.	.	.	.	.	.	.	.	.	.	.	.	.	.	.	.	.	.	.	.	.	.	.
Carex atropicta	.	.	.	.	.	.	.	.	.	.	.	.	.	.	.	.	.	.	.	.	.	.	.	.	.	.	.	.	.
Carex banksii	.	.	.	.	.	.	.	.	.	.	.	.	.	.	.	.	.	.	.	.	.	.	.	.	.	.	.	.	.
Carex caduca	.	.	.	.	.	.	.	.	.	.	.	.	.	.	.	.	.	.	.	.	.	.	.	.	.	.	.	.	.
Carex decidua	.	.	.	.	.	.	.	.	.	.	.	.	.	.	.	.	.	.	.	.	.	.	.	.	.	.	.	.	.
Carex fuscula	.	.	.	.	.	.	.	.	.	.	.	.	.	.	.	.	.	.	.	.	.	.	.	.	.	.	.	.	.
Carex gayana	1	1	1	.	1	1	.	.	.	.	1	1	1	.	.	.	1	.	.	.	1	1	1	1	1	.	.	.	.
Carex hypoleucos	.	.	.	.	.	.	.	.	.	.	.	.	.	.	.	.	.	.	.	.	.	.	.	.	.	.	.	.	.
Carex macloviana	.	.	.	.	.	.	.	.	.	.	.	.	.	.	.	.	.	.	.	.	.	.	.	.	.	.	.	.	.
Carex magellanica	.	.	.	.	.	.	.	.	.	.	.	.	.	.	.	.	.	.	.	.	.	.	.	.	.	.	.	.	.
Carex malmei	.	.	.	1	.	.	1	1	1	.	.	.	.	1	.	.	.	.	.	.	.	.	1	.	.	.	.	.	.
Carex maritima	.	1	1	1	.	1	1	1	1	1	.	.	1	.	1	1	1	1	1	1	1	1	.	1	1	1	1	.	1
Carex microglochin	.	.	.	.	.	.	1	1	.	.	.	.	.	1	.	.	.	.	.	.	.	.	.	.	.	.	.	.	.
Carex pleioneura	.	.	.	.	.	.	.	.	.	.	.	.	.	.	.	.	.	.	.	.	.	.	.	.	.	.	.	.	.
Carex ruthsatzae	.	.	.	.	.	.	.	.	.	.	.	.	.	.	.	.	.	.	.	.	.	.	.	.	.	.	.	.	.

Bog	378	379	380	381	382	383	384	385	386	387	388	389	390	391	392	393	394	395	396	397	398	399	400	401	402	403	404	405	406
Carex vallis-pulchrae	1	.	.	.	.	.	.	.	.	.	.	1	.	.	.	.	.	1	.	1	.	.	1	.	.	.	.	.	.
Carpha schoenoides	.	.	.	.	.	.	.	.	.	.	.	.	.	.	.	.	.	.	.	.	.	.	.	.	.	.	.	.	.
Castilleja pumila	.	.	.	.	.	.	.	.	.	.	.	.	.	.	.	.	.	.	.	.	.	.	.	.	.	.	.	.	.
Catabrosa werdermannii	.	.	.	.	.	.	.	.	.	.	.	.	.	.	1	.	1	1	1	1	1	.	.	.	.	.	.	1	.
Cerastium humifusum	1	1	.	.	.	.	.	.	.	.	.	1	.	.	1	.	1	1	.	1	.	1	1	1	.	.	.	.	.
Cerastium montioides	.	.	.	.	.	.	.	.	.	.	.	.	.	.	.	.	.	.	.	.	.	.	.	.	.	.	.	.	.
Chiliotrichum diffusum	.	.	.	.	.	.	.	.	.	.	.	.	.	.	.	.	.	.	.	.	.	.	.	.	.	.	.	.	.
Chusquea culeou	.	.	.	.	.	.	.	.	.	.	.	.	.	.	.	.	.	.	.	.	.	.	.	.	.	.	.	.	.
Colobanthus quitensis	.	.	1	1	.	1	.	.	.	1	.	.	1	.	.	.	.	.	.	.	.	.	.	.	.	.	.	.	.
Cortaderia egmontiana	.	.	.	.	.	.	.	.	.	.	.	.	.	.	.	.	.	.	.	.	.	.	.	.	.	.	.	.	.
Cotula mexicana	.	.	.	.	.	.	.	.	.	.	.	.	.	.	.	.	.	.	.	.	.	.	.	.	.	.	.	.	.
Crassula peduncularis	.	.	.	.	.	.	.	.	.	.	.	.	.	.	.	.	.	.	.	.	.	.	.	.	.	.	.	.	.
Cuatrecasasiella argentina	.	.	.	.	.	.	.	.	.	.	.	.	.	.	.	.	.	.	.	.	.	.	.	.	.	.	.	.	.
Deschampsia antarctica	.	.	.	.	.	.	.	.	.	.	.	.	.	.	.	.	.	.	.	.	.	.	.	.	.	.	.	.	.
Deschampsia caespitosa	1	.	.	.	.	.	.	.	.	.	.	.	.	.	.	.	.	.	.	.	.	.	.	.	.	.	.	.	.
Deschampsia patula	.	.	.	.	.	.	.	.	.	.	.	.	.	.	.	.	.	.	.	.	.	.	.	.	.	.	.	.	.
Cinnagrostis brevifolia	.	.	.	.	.	.	.	.	.	.	.	.	.	.	.	.	.	.	.	.	.	.	.	.	.	.	.	.	.
Deschampsia chrysantha	.	.	.	.	.	.	.	.	.	.	.	.	.	.	.	.	.	.	.	.	.	.	.	.	.	.	.	.	.
Cinnagrostis chrysophylla	.	.	.	.	.	.	.	.	.	.	.	.	.	.	.	.	.	.	.	.	.	.	.	.	.	.	.	.	.
Deschampsia chrysostachya	.	1	1	.	.	.	.	.	.	.	.	.	.	.	.	.	.	.	.	.	.	.	.	.	.	.	.	.	.
Deschampsia eminens	.	1	.	1	.	1	1	.	1	1	.	1	1	.	1	1	1	1	1	1	1	1	1	1	1	1	1	1	1
Deschampsia hackelii	.	.	.	.	.	.	.	.	.	.	.	.	.	.	.	.	.	.	.	.	.	.	.	.	.	.	.	.	.
Cinnagrostis minima	.	.	.	.	.	.	.	.	.	.	.	.	.	.	.	.	.	.	.	.	.	.	.	.	.	.	.	.	.
Deschampsia ovata	.	.	.	.	.	.	.	.	.	.	.	.	.	.	.	.	.	.	.	.	.	.	.	.	.	.	.	.	.
Cinnagrostis rigescens	.	.	.	.	.	.	.	.	.	.	.	.	.	.	.	.	.	.	.	.	.	.	.	.	.	.	.	.	.
Cinnagrostis spicigera	.	.	.	.	.	.	.	.	.	.	.	.	.	.	.	.	.	.	.	.	.	.	.	.	.	.	.	.	.
Cinnagrostis velutina	1	1	1	1	.	1	1	1	1	1	.	.	1	1	1	1	1	1	1	1	1	1	1	1	1	1	.	1	.
Cinnagrostis vicunarum	.	.	.	.	.	.	.	.	.	.	.	.	.	.	.	.	.	.	.	.	.	.	.	.	.	.	.	.	.
Distichia filamentosa	.	.	.	.	.	.	.	.	.	.	.	.	.	.	.	.	.	.	.	.	.	.	.	.	.	.	.	.	.
Distichia muscoides	.	.	.	.	.	.	.	.	.	.	.	.	.	.	.	.	.	.	.	.	.	.	.	.	.	.	.	.	.
Distichlis humilis	.	.	.	.	.	.	.	.	.	.	.	.	.	.	.	.	.	.	.	.	.	.	.	.	.	.	.	.	.

Bog	378	379	380	381	382	383	384	385	386	387	388	389	390	391	392	393	394	395	396	397	398	399	400	401	402	403	404	405	406
Distichlis scoparia	.	.	.	.	.	.	.	.	.	.	.	.	.	.	.	.	.	.	.	.	.	.	.	.	.	.	.	.	.
Distichlis spicata	.	.	.	.	.	.	.	.	.	.	.	.	.	.	.	.	.	.	.	.	.	.	.	.	.	.	.	.	.
Draba pusilla	.	.	.	.	.	.	.	.	.	.	.	.	.	.	.	.	.	1	.	.	.	.	.	.	.	.	.	.	.
Eleocharis melanomphala	.	.	.	.	.	.	.	.	.	.	.	.	.	.	.	.	.	.	.	.	.	.	.	.	.	.	.	.	.
Eleocharis pseudoalbibracteata	1	1	1	1	1	1	.	1	.	.	.	1	.	.	.	.	.	.	.	1	1	.	1	1	.	.	.	.	.
Elodea potamogeton	.	.	.	.	.	.	.	.	.	.	.	.	.	.	.	.	.	.	.	.	.	.	.	.	.	.	.	.	.
Empetrum rubrum	.	.	.	.	.	.	.	.	.	.	.	.	.	.	.	.	.	.	.	.	.	.	.	.	.	.	.	.	.
Epilobium australe	.	.	.	.	.	.	.	.	.	.	.	.	.	.	.	.	.	.	.	.	.	.	.	.	.	.	.	.	.
Epilobium barbeyanum	.	.	.	.	.	.	.	.	.	.	.	.	.	.	.	.	.	.	.	.	.	.	.	.	.	.	.	.	.
Epilobium ciliatum	1	.	.	.	.	.	.	.	.	.	.	.	.	.	.	.	.	.	.	.	.	.	.	.	.	.	.	.	.
Epilobium denticulatum	.	.	.	.	.	.	.	.	.	.	.	.	.	.	.	.	.	.	.	.	.	.	.	.	.	.	.	.	.
Epilobium fragile	.	.	.	.	.	.	.	.	.	.	.	.	.	.	.	.	.	.	.	.	.	.	.	.	.	.	.	.	.
Epilobium glaucum	.	.	.	.	.	.	.	.	.	.	.	.	.	.	.	.	.	.	.	.	.	.	.	.	.	.	.	.	.
Epilobium nivale	.	.	.	.	.	.	.	.	.	.	.	.	.	.	.	.	.	.	.	.	.	.	.	.	.	.	.	.	.
Erigeron andicola	.	.	.	.	.	.	.	.	.	.	.	.	.	.	.	.	.	.	.	.	.	.	.	.	.	.	.	.	.
Erigeron leptopetalus	1	.	.	.	.	.	.	.	.	.	.	1	1	.	.	.	.	.	.	1	.	.	1	.	.	.	.	.	.
Erigeron myosotis	.	.	.	.	.	.	.	.	.	.	.	.	.	.	.	.	.	.	.	.	.	.	.	.	.	.	.	.	.
Erigeron patagonicus	.	.	.	.	.	.	.	.	.	.	.	.	.	.	.	.	.	.	.	.	.	.	.	.	.	.	.	.	.
Erythranthe cuprea	.	.	.	.	.	.	.	.	.	.	.	.	.	.	.	.	.	.	.	.	.	.	.	.	.	.	.	.	.
Erythranthe depressa	1	1	1	1	1	1	1	1	1	.	1	1	.	1	.	.	1	.	.	.	1	.	1	.	.	.	.	.	.
Erythranthe glabrata	.	.	.	.	.	.	.	.	.	.	.	.	.	.	.	.	.	.	.	.	.	.	.	.	.	.	.	.	.
Erythranthe lutea	.	.	.	.	.	.	.	.	.	.	.	.	.	.	.	.	.	.	.	.	.	.	.	.	.	.	.	.	.
Escallonia virgata	.	.	.	.	.	.	.	.	.	.	.	.	.	.	.	.	.	.	.	.	.	.	.	.	.	.	.	.	.
Euphrasia antarctica	.	.	1	.	.	.	.	.	.	.	.	.	.	.	.	.	.	.	.	.	.	.	.	.	.	.	.	.	.
Euphrasia chrysantha	.	.	.	.	.	.	.	.	.	.	.	.	.	.	.	.	.	.	.	.	.	.	.	.	.	.	.	.	.
Euphrasia subexserta	.	.	.	.	.	.	.	.	.	.	.	.	.	.	.	.	.	.	.	.	.	.	.	.	.	.	.	.	.
Festuca hypsophila	.	.	.	.	.	.	.	.	.	.	.	.	.	.	.	.	.	.	.	.	.	.	.	.	.	.	.	.	.
Festuca kurtziana	.	.	.	.	.	.	.	.	.	.	.	.	.	.	.	.	.	.	.	.	.	.	.	.	.	.	.	.	.
Festuca lilloi	.	.	.	.	.	.	.	.	.	.	.	.	.	.	.	.	.	.	.	.	.	.	.	.	.	.	.	.	.
Festuca magellanica	.	.	.	.	.	.	.	.	.	.	.	.	.	.	.	.	.	.	.	.	.	.	.	.	.	.	.	.	.
Festuca nardifolia	1	1	.	.	1	1	.	1	.	.	1	.	.	1	.	.	.	.	.	.	.	.	1	.	.	.	.	.	.
Festuca rigescens	.	.	.	.	.	.	.	.	.	.	.	.	.	.	.	.	.	.	.	.	.	.	.	.	.	.	.	.	.
Festuca werdermannii	.	.	.	.	.	.	.	.	.	.	.	.	.	.	.	.	.	.	.	.	.	.	.	.	.	.	.	.	.
Frankenia triandra	.	.	.	.	.	.	.	.	.	.	.	.	.	.	.	.	.	.	.	.	.	.	.	.	.	.	.	.	.
Gamocarpha graminea	.	.	.	.	.	.	.	.	.	.	.	.	.	.	.	.	.	.	.	.	.	.	.	.	.	.	.	.	.

Bog	378	379	380	381	382	383	384	385	386	387	388	389	390	391	392	393	394	395	396	397	398	399	400	401	402	403	404	405	406
Gamocarpha ventosa	.	.	.	1	.	.	1	1	1	.	.	.	1	1	1	1	.	1	.	.	.	.	.	.	.	.	.	.	.
Gamochaeta chamissonis	.	.	.	.	.	.	.	.	.	.	.	.	.	.	.	.	.	.	.	.	.	.	.	.	.	.	.	.	.
Gamochaeta longipedicellata	.	.	.	.	.	.	.	.	.	.	.	.	.	.	.	.	.	.	.	.	.	.	.	.	.	.	.	.	.
Gamochaeta neuquensis	.	.	.	.	.	.	.	.	.	.	.	.	.	.	.	.	.	.	.	.	.	.	.	.	.	.	.	.	.
Gaultheria antarctica	.	.	.	.	.	.	.	.	.	.	.	.	.	.	.	.	.	.	.	.	.	.	.	.	.	.	.	.	.
Gaultheria caespitosa	.	.	.	.	.	.	.	.	.	.	.	.	.	.	.	.	.	.	.	.	.	.	.	.	.	.	.	.	.
Gaultheria pumila	.	.	.	.	.	.	.	.	.	.	.	.	.	.	.	.	.	.	.	.	.	.	.	.	.	.	.	.	.
Gavilea chica	.	.	.	.	.	.	.	.	.	.	.	.	.	.	.	.	.	.	.	.	.	.	.	.	.	.	.	.	.
Gentiana prostrata	.	1	1	1	.	1	1	1	1	1	.	.	1	1	1	1	1	1	1	1	.	1	1	1	.	1	.	.	.
Gentianella fiebrigii	.	.	.	.	.	.	.	.	.	.	.	.	.	.	.	.	.	.	.	.	.	.	.	.	.	.	.	.	.
Gentianella magellanica	.	.	.	.	.	.	.	.	.	.	.	.	.	.	.	.	.	.	.	.	.	.	.	.	.	.	.	.	.
Gentianella multicaulis	1	.	.	.	.	.	.	.	.	1	.	.	.	1	.	.	.	.	.	.	.	.	1	.	.	.	.	.	.
Gentianella ottonis	.	.	.	.	.	.	.	.	.	.	.	.	.	.	.	.	.	.	.	.	.	.	.	.	.	.	.	.	.
Gentianella primuloides	.	.	.	.	.	.	.	.	.	.	.	.	.	.	.	.	.	.	.	.	.	.	.	.	.	.	.	.	.
Gentianella pseudocrassula	.	1	.	.	.	.	.	.	.	.	.	.	.	.	.	.	.	.	.	.	.	.	.	.	.	.	.	.	.
Geranium sessiliflorum	.	.	.	.	.	.	.	.	.	.	.	.	.	.	.	.	.	.	.	.	.	.	.	.	.	.	.	.	.
Gunnera magellanica	.	.	.	.	.	.	.	.	.	.	.	.	.	.	.	.	.	.	.	.	.	.	.	.	.	.	.	.	.
Halenia caespitosa	.	.	.	.	.	.	.	.	.	.	.	.	.	.	.	.	.	.	.	.	.	.	.	.	.	.	.	.	.
Halerpestes cymbalaria	.	.	.	.	.	.	.	.	.	.	.	.	.	.	.	.	1	1	.	1	1	.	.	.	.	1	1	.	.
Halerpestes exilis	.	.	.	.	.	.	.	.	.	.	.	.	.	.	.	.	.	.	1	1	.	.	.	.	.	.	.	1	.
Hieracium antarcticum	.	.	.	.	.	.	.	.	.	.	.	.	.	.	.	.	.	.	.	.	.	.	.	.	.	.	.	.	.
Hordeum comosum	.	.	.	.	.	.	.	.	.	.	.	.	.	.	.	.	.	.	.	.	.	.	.	.	.	.	.	.	.
Hordeum muticum	.	.	.	.	.	.	.	.	.	.	.	.	.	.	.	.	.	.	.	.	.	.	.	.	.	.	.	.	.
Hypochaeris acaulis	.	.	.	.	.	.	.	.	.	.	.	.	.	.	.	.	.	.	.	.	.	.	.	.	.	.	.	.	.
Hypochaeris chondrilloides	.	.	.	.	.	.	.	.	.	.	.	.	.	.	.	.	.	.	.	.	.	.	.	.	.	.	.	.	.
Hypochaeris meyeniana	.	.	.	.	.	.	.	.	.	.	.	.	.	.	.	.	.	.	.	.	.	.	.	.	.	.	.	.	.
Hypochaeris palustris	.	.	.	.	.	.	.	.	.	.	.	.	.	.	.	.	.	.	.	.	.	.	.	.	.	.	.	.	.
Hypochaeris taraxacoides	.	.	.	.	.	.	.	.	.	.	.	.	.	.	.	.	.	.	.	.	.	.	.	.	.	.	.	.	.
Hypochaeris tenerifolia	.	.	.	.	.	.	.	.	.	.	.	.	.	.	.	.	.	.	.	.	.	.	.	.	.	.	.	.	.
Isolepis nigricans	.	.	.	.	.	.	.	.	.	.	.	.	.	.	.	.	.	.	.	.	.	.	.	.	.	.	.	.	.

Bog	378	379	380	381	382	383	384	385	386	387	388	389	390	391	392	393	394	395	396	397	398	399	400	401	402	403	404	405	406
Isolepsis inundata	.	.	.	.	.	.	.	.	.	.	.	.	.	.	.	.	.	.	.	.	.	.	.	.	.	.	.	.	.
Juncus balticus	1	.	.	1	1	1	.	.	.	.	.	1	.	.	.	.	.	.	.	.	1	1	1	.	.	.	.	1	.
Juncus stipulatus	1	1	1	1	1	1	1	1	1	.	1	.	.	1	.	.	.	.	.	.	1	.	.	.	.	.	.	.	.
Koeleria kurtzii	.	.	.	.	.	.	.	.	.	.	.	.	.	.	.	.	.	.	.	.	.	.	.	.	.	.	.	.	.
Lachemilla diplophylla	.	.	.	.	.	.	.	.	.	.	.	.	.	.	.	.	.	.	.	.	.	.	.	.	.	.	.	.	.
Lachemilla pinnata	.	.	.	.	.	.	.	.	.	.	.	.	.	.	.	.	.	.	.	.	.	.	.	.	.	.	.	.	.
Lagenophora nudicaulis	.	.	.	.	.	.	.	.	.	.	.	.	.	.	.	.	.	.	.	.	.	.	.	.	.	.	.	.	.
Lemna minuta	.	.	.	.	.	.	.	.	.	.	.	.	.	.	.	.	.	.	.	.	.	.	.	.	.	.	.	.	.
Leptinella scariosa	.	.	.	.	.	.	.	.	.	.	.	.	.	.	.	.	.	.	.	.	.	.	.	.	.	.	.	.	.
Leucheria candidissima	.	.	.	.	.	.	.	.	.	.	.	.	.	.	.	.	.	.	.	.	.	.	.	.	.	.	.	.	.
Leucheria nutans	.	.	.	.	.	.	.	.	.	.	.	.	.	.	.	.	.	.	.	.	.	.	.	.	.	.	.	.	.
Lilaea scilloides	.	.	.	.	.	.	.	.	.	.	.	.	.	.	.	.	.	.	.	.	.	.	.	.	.	.	.	.	.
Lilaeopsis macloviana	.	.	.	.	.	.	.	.	.	.	.	.	.	.	.	.	.	.	.	.	.	.	.	.	.	.	.	.	.
Limosella australis	.	.	.	.	.	.	.	.	.	.	.	.	.	.	.	.	.	.	.	.	.	.	.	.	.	.	.	.	.
Lobelia oligophylla	1	1	1	.	.	.	.	.	1	.	.	1	1	1	.	.	.	.	.	.	1	.	1	.	.	.	.	.	.
Luzula brachyphylla	.	.	.	.	.	.	.	.	.	.	.	.	.	.	.	.	.	.	.	.	.	.	.	.	.	.	.	.	.
Luzula chilensis	.	.	.	.	.	.	.	.	.	.	.	.	.	.	.	.	.	.	.	.	.	.	.	.	.	.	.	.	.
Luzula racemosa	.	.	.	.	.	.	.	.	.	.	.	.	.	.	.	.	.	.	.	.	.	.	.	.	.	.	.	.	.
Luzula vulcanica	.	.	.	.	.	.	.	.	.	.	.	.	.	.	.	.	.	.	.	.	.	.	.	.	.	.	.	.	.
Lysipomia pumila	.	.	.	.	.	.	.	.	.	.	.	.	.	.	.	.	.	.	.	.	.	.	.	.	.	.	.	.	.
Marsippospermum philippii	.	.	.	.	.	.	.	.	.	.	.	.	.	.	.	.	.	.	.	.	.	.	.	.	.	.	.	.	.
Marsippospermum reichei	.	.	.	.	.	.	.	.	.	.	.	.	.	.	.	.	.	.	.	.	.	.	.	.	.	.	.	.	.
Montia fontana	.	.	.	.	.	.	.	.	.	.	.	.	.	.	.	.	.	.	.	.	.	.	.	.	.	.	.	.	.
Muhlenbergia asperifolia	.	.	.	.	.	.	.	.	.	.	.	.	.	.	.	.	.	.	.	.	.	.	.	.	.	.	.	.	.
Myriophyllum quitense	.	1	1	.	.	.	.	.	.	.	.	.	.	.	.	.	.	.	.	.	.	.	.	.	1	.	.	.	.
Myrosmodes nervosa	.	.	.	.	.	.	.	.	.	.	.	.	.	.	.	.	.	.	.	.	.	.	.	.	.	.	.	.	.
Myrosmodes paludosa	.	.	.	.	.	.	.	.	.	.	.	.	.	.	.	.	.	.	.	.	.	.	.	.	.	.	.	.	.
Myrteola nummularia	.	.	.	.	.	.	.	.	.	.	.	.	.	.	.	.	.	.	.	.	.	.	.	.	.	.	.	.	.
Nanodea muscosa	.	.	.	.	.	.	.	.	.	.	.	.	.	.	.	.	.	.	.	.	.	.	.	.	.	.	.	.	.
Neobartsia crenoloba	.	.	.	.	.	.	.	.	.	.	.	.	.	.	.	.	.	.	.	.	.	.	.	.	.	.	.	.	.
Neobartsia pedicularoides	.	.	.	.	.	.	.	.	.	.	.	.	.	.	.	.	.	.	.	.	.	.	.	.	.	.	.	.	.
Neobartsia peruviana	.	.	.	.	.	.	.	.	.	.	.	.	.	.	.	.	.	.	.	.	.	.	.	.	.	.	.	.	.

Bog	378	379	380	381	382	383	384	385	386	387	388	389	390	391	392	393	394	395	396	397	398	399	400	401	402	403	404	405	406
Nertera granadensis	.	.	.	.	.	.	.	.	.	.	.	.	.	.	.	.	.	.	.	.	.	.	.	.	.	.	.	.	.
Nicoraepoa andina	.	.	.	.	.	.	.	.	.	.	.	.	.	.	.	.	.	.	.	.	.	.	.	.	.	.	.	.	.
Nicoraepoa pugionifolia	.	.	.	.	.	.	.	.	.	.	.	.	.	.	.	.	.	.	.	.	.	.	.	.	.	.	.	.	.
Nicoraepoa subenervis	.	1	.	.	.	.	.	.	.	.	.	.	.	.	.	.	.	.	.	.	.	.	.	.	.	.	.	.	.
Nitrophila australis	.	.	.	.	.	.	.	.	.	.	.	.	.	.	.	.	.	.	.	.	.	.	.	.	.	.	.	.	.
Nothofagus antarctica	.	.	.	.	.	.	.	.	.	.	.	.	.	.	.	.	.	.	.	.	.	.	.	.	.	.	.	.	.
Nototriche rugosa	.	.	.	.	.	.	.	.	.	.	.	.	.	.	.	.	.	.	.	.	.	.	.	.	.	.	.	.	.
Ochetophila nana	.	.	.	.	.	.	.	.	.	.	.	.	.	.	.	.	.	.	.	.	.	.	1	.	.	.	.	.	.
Olsynium junceum	.	.	.	.	.	.	.	.	.	.	.	.	.	.	.	.	.	.	.	.	.	.	.	.	.	.	.	.	.
Oreobolus obtusangulus	.	.	.	.	.	.	.	.	.	.	.	.	.	.	.	.	.	.	.	.	.	.	.	.	.	.	.	.	.
Oritrophium limnophilum	.	.	.	.	.	.	.	.	.	.	.	.	.	.	.	.	.	.	.	.	.	.	.	.	.	.	.	.	.
Osmorhiza glabrata	.	.	.	.	.	.	.	.	.	.	.	.	.	.	.	.	.	.	.	.	.	.	.	.	.	.	.	.	.
Ourisia alpina	.	.	.	.	.	.	.	.	.	.	.	.	.	.	.	.	.	.	.	.	.	.	.	.	.	.	.	.	.
Ourisia muscosa	.	.	.	.	.	.	.	.	.	.	.	.	.	.	.	.	.	.	.	.	.	.	.	.	.	.	.	.	.
Ourisia ruelloides	.	.	.	.	.	.	.	.	.	.	.	.	.	.	.	.	.	.	.	.	.	.	.	.	.	.	.	.	.
Oxychloe andina	.	.	.	.	.	.	.	.	.	.	.	.	.	.	.	.	1	.	1	1	1	1	.	1	1	1	.	1	.
Oxychloe bisexualis	.	.	.	.	.	1	.	.	.	.	.	.	.	1	.	.	.	.	.	.	.	.	.	.	.	.	.	.	.
Oxychloe castellanosii	.	.	.	1	1	.	1	1	1	1	1	.	1	.	1	1	.	1	.	.	.	.	.	.	.	.	.	.	.
Oxychloe haumaniana	.	.	.	.	.	.	.	.	.	.	.	.	.	.	.	.	.	.	.	.	.	.	.	.	.	.	.	.	.
Oxychloe mendocina	.	.	.	.	.	.	.	.	.	.	.	.	.	.	.	.	.	.	.	.	.	.	.	.	.	.	.	.	.
Patosia clandestina	1	1	1	.	.	.	.	.	.	.	.	1	.	.	.	.	.	.	.	.	.	.	1	.	.	.	.	.	.
Perezia capito	.	.	.	.	.	.	.	.	.	.	.	.	.	.	.	.	.	.	.	.	.	.	.	.	.	.	.	.	.
Perezia delicata	.	.	.	.	.	.	.	.	.	.	.	.	.	.	.	.	.	.	.	.	.	.	.	.	.	.	.	.	.
Perezia fonkii	.	.	.	.	.	.	.	.	.	.	.	.	.	.	.	.	.	.	.	.	.	.	.	.	.	.	.	.	.
Perezia pedicularidifolia	.	.	.	.	.	.	.	.	.	.	.	.	.	.	.	.	.	.	.	.	.	.	.	.	.	.	.	.	.
Perezia pinnatifida	.	.	.	.	.	.	.	.	.	.	.	.	.	.	.	.	.	.	.	.	.	.	.	.	.	.	.	.	.
Petroravenia friesii	.	.	.	.	.	.	.	.	.	.	.	.	.	.	.	.	.	.	.	.	.	.	.	.	.	.	.	.	.
Petroravenia werdermannii	.	.	.	.	.	.	.	.	.	.	.	.	.	1	1	1	.	.	.	1	.	.	.	.	.	.	.	.	.
Phleum alpinum	.	1	.	.	.	.	.	.	1	.	.	1	.	.	.	.	.	.	.	.	.	.	.	.	.	.	.	.	.
Phylloscirpus acaulis	1	.	.	.	.	.	.	.	.	.	.	1	.	1	.	.	.	.	.	.	1	.	1	.	.	.	.	.	.
Phylloscirpus boliviensis	.	.	.	.	.	.	.	.	.	.	.	.	.	.	.	.	.	.	.	.	.	.	.	.	.	.	.	.	.

**Bog**	**378**	**379**	**380**	**381**	**382**	**383**	**384**	**385**	**386**	**387**	**388**	**389**	**390**	**391**	**392**	**393**	**394**	**395**	**396**	**397**	**398**	**399**	**400**	**401**	**402**	**403**	**404**	**405**	**406**
Phylloscirpus deserticola	.	1	1	.	.	.	.	.	.	.	.	.	.	.	.	.	.	.	.	.	.	.	.	.	.	.	.	.	.
Pinguicula antarctica	.	.	.	.	.	.	.	.	.	.	.	.	.	.	.	.	.	.	.	.	.	.	.	.	.	.	.	.	.
Plantago barbata	1	1	1	1	1	1	1	1	1	1	1	.	1	1	.	.	.	.	.	.	.	1	1	1	.	.	.	.	.
Plantago rigida	.	.	.	.	.	.	.	.	.	.	.	.	.	.	.	.	.	.	.	.	.	.	.	.	.	.	.	.	.
Plantago tubulosa	.	.	.	.	.	.	.	.	.	.	.	.	.	.	.	.	.	.	.	.	.	.	.	.	.	.	.	.	.
Plantago uniglumis	.	.	.	.	.	.	.	.	.	.	.	.	.	1	.	.	.	.	.	.	.	.	.	.	.	.	.	.	.
Poa alopecurus	.	.	.	.	.	.	.	.	.	.	.	.	.	.	.	.	.	.	.	.	.	.	.	.	.	.	.	.	.
Poa hachadoensis	.	.	.	.	.	.	.	.	.	.	.	.	.	.	.	.	.	.	.	.	.	.	.	.	.	.	.	.	.
Poa perligulata	.	.	.	.	.	.	.	.	.	.	.	.	.	.	.	.	.	.	.	.	.	.	.	.	.	.	.	.	.
Polypogon interruptus	.	.	.	.	.	.	.	.	.	.	.	.	.	.	.	.	.	.	.	.	.	.	.	.	.	.	.	.	.
Primula magellanica	.	.	.	.	.	.	.	.	.	.	.	.	.	.	.	.	.	.	.	.	.	.	.	.	.	.	.	.	.
Puccinellia frigida	.	.	.	.	.	.	.	.	.	.	.	.	.	.	1	1	1	1	1	1	1	.	.	.	.	1	1	1	1
Quinchamalium chilense	.	.	.	.	.	.	.	.	.	.	.	.	.	.	.	.	.	.	.	.	.	.	.	.	.	.	.	.	.
Ranunculus breviscapus	.	.	.	.	.	.	.	.	.	.	.	.	.	.	.	.	.	.	.	.	.	.	.	.	.	.	.	.	.
Ranunculus fuegianus	.	.	.	.	.	.	.	.	.	.	.	.	.	.	.	.	1	.	.	.	1	.	.	.	1	.	.	.	.
Ranunculus mandonianus	.	.	.	.	.	.	.	.	.	.	.	.	.	.	.	.	.	.	.	.	.	.	.	.	.	.	.	.	.
Ranunculus peduncularis	.	.	.	.	.	.	.	.	.	.	.	.	.	.	.	.	.	.	.	.	.	.	.	.	.	.	.	.	.
Ranunculus trichophyllus	.	.	1	.	.	.	.	.	.	.	.	.	.	.	.	.	.	.	.	.	.	.	.	1	.	.	.	.	.
Halerpestes uniflora	.	.	.	.	.	.	.	.	.	.	.	.	.	.	.	.	.	.	.	.	.	.	.	.	.	.	.	.	.
Rubus geoides	.	.	.	.	.	.	.	.	.	.	.	.	.	.	.	.	.	.	.	.	.	.	.	.	.	.	.	.	.
Rumex magellanicus	.	.	.	.	.	.	.	.	.	.	.	.	.	.	.	.	.	.	.	.	.	.	.	.	.	.	.	.	.
Rytidosperma lechleri	.	.	.	.	.	.	.	.	.	.	.	.	.	.	.	.	.	.	.	.	.	.	.	.	.	.	.	.	.
Sarcocornia pulvinata	.	.	.	.	.	.	.	.	.	.	.	.	.	.	.	.	.	.	.	.	.	.	.	.	.	.	.	.	.
Schoenoplectus pungens	.	.	.	.	.	.	.	.	.	.	.	.	.	.	.	.	.	.	.	.	.	.	.	.	.	.	.	.	.
Schoenus andinus	.	.	.	.	.	.	.	.	.	.	.	.	.	.	.	.	.	.	.	.	.	.	.	.	.	.	.	.	.
Senecio breviscapus	1	.	.	.	1	.	.	.	.	.	1	.	.	.	.	.	.	.	.	.	.	.	.	.	.	.	.	.	.
Senecio diemii	.	.	.	.	.	.	.	.	.	.	.	.	.	.	.	.	.	.	.	.	.	.	.	.	.	.	.	.	.
Senecio fistulosus	.	.	.	.	.	.	.	.	.	.	.	.	.	.	.	.	.	.	.	.	.	.	.	.	.	.	.	.	.
Senecio parodii	.	.	.	.	.	.	.	.	.	.	.	.	.	.	.	.	.	.	.	.	.	.	.	.	.	.	.	.	.
Senecio peteroanus	.	.	.	.	.	.	.	.	.	.	.	.	.	.	.	.	.	.	.	.	.	.	.	.	.	.	.	.	.
Senecio serratifolius	.	.	.	.	.	.	.	.	.	.	.	.	.	.	.	.	.	.	.	.	.	.	.	.	.	.	.	.	.

Bog	378	379	380	381	382	383	384	385	386	387	388	389	390	391	392	393	394	395	396	397	398	399	400	401	402	403	404	405	406
Senecio trifurcatus	.	.	.	.	.	.	.	.	.	.	.	.	.	.	.	.	.	.	.	.	.	.	.	.	.	.	.	.	.
Sisyrinchium chilense	1	.	.	.	.	.	.	.	.	.	.	.	.	.	.	.	.	.	.	.	.	.	.	.	.	.	.	.	.
Sisyrinchium patagonicum	.	.	.	.	.	.	.	.	.	.	.	.	.	.	.	.	.	.	.	.	.	.	.	.	.	.	.	.	.
Sisyrinchium pearcei	.	.	.	.	.	.	.	.	.	.	.	.	.	.	.	.	.	.	.	.	.	.	.	.	.	.	.	.	.
Stellaria debilis	.	.	.	.	.	.	.	.	.	.	.	.	.	.	.	.	.	.	.	.	.	.	.	.	.	.	.	.	.
Stuckenia filiformis	.	.	.	.	.	.	.	.	.	.	.	.	.	.	1	1	1	.	.	.	1	.	.	.	1	1	1	1	1
Stuckenia striata	.	.	.	.	.	.	.	.	.	.	.	.	.	.	.	.	.	.	.	.	.	.	.	.	.	.	.	.	.
Symphyotrichum peteroanum	.	.	.	.	.	.	.	.	.	.	.	.	.	.	.	.	.	.	.	.	.	.	.	.	.	.	.	.	.
Symphyotrichum vahlii	.	.	.	.	.	.	.	.	.	.	.	.	.	.	.	.	.	.	.	.	.	.	.	.	.	.	.	.	.
Tetroncium magellanicum	.	.	.	.	.	.	.	.	.	.	.	.	.	.	.	.	.	.	.	.	.	.	.	.	.	.	.	.	.
Tribeles australis	.	.	.	.	.	.	.	.	.	.	.	.	.	.	.	.	.	.	.	.	.	.	.	.	.	.	.	.	.
Trifolium amabile	.	.	.	.	.	.	.	.	.	.	.	.	.	.	.	.	.	.	.	.	.	.	.	.	.	.	.	.	.
Trifolium polymorphum	.	.	.	.	.	.	.	.	.	.	.	.	.	.	.	.	.	.	.	.	.	.	.	.	.	.	.	.	.
Triglochin concinna	.	.	.	.	.	.	.	.	.	.	.	.	.	.	.	.	.	.	.	.	.	.	.	.	.	.	.	.	.
Triglochin palustris	.	.	.	.	.	.	.	.	.	.	.	.	.	1	.	.	.	.	.	.	.	.	.	.	1	.	.	.	.
Triglochin striata	.	.	.	.	.	.	.	.	.	.	.	.	.	.	.	.	.	.	.	.	.	.	.	.	.	.	.	.	.
Trisetum caudulatum	.	.	.	.	.	.	.	.	.	.	.	.	.	.	.	.	.	.	.	.	.	.	.	.	.	.	.	.	.
Trisetum preslei	.	.	.	.	.	.	.	.	.	.	.	.	.	.	.	.	.	.	.	.	.	.	.	.	.	.	.	.	.
Koeleria spicata	.	.	.	.	.	.	.	.	.	.	.	.	.	.	.	.	.	.	.	.	.	.	.	.	.	.	.	.	.
Utricularia gibba	.	.	.	.	.	.	.	.	.	.	.	.	.	.	.	.	.	.	.	.	.	.	.	.	.	.	.	.	.
Vahlodea atropurpurea	.	.	.	.	.	.	.	.	.	.	.	.	.	.	.	.	.	.	.	.	.	.	.	.	.	.	.	.	.
Valeriana fonckii	.	.	.	.	.	.	.	.	.	.	.	.	.	.	.	.	.	.	.	.	.	.	.	.	.	.	.	.	.
Valeriana macrorhiza	.	.	.	.	.	.	.	.	.	.	.	.	.	.	.	.	.	.	.	.	.	.	.	.	.	.	.	.	.
Viola pygmaea	.	.	.	.	.	.	.	.	.	.	.	.	.	.	.	.	.	.	.	.	.	.	.	.	.	.	.	.	.
Werneria apiculata	.	.	.	.	.	.	.	.	.	.	.	.	.	.	.	.	.	.	.	.	.	.	.	.	.	.	.	.	.
Werneria pinnatifida	.	.	1	1	.	1	.	.	1	1	.	.	1	.	1	1	.	1	.	1	1	1	.	1	.	.	.	.	.
Werneria pygmaea	1	1	1	1	.	1	1	1	1	1	1	1	1	1	.	1	.	.	.	.	1	1	1	1	.	.	.	.	.
Werneria solivifolia	.	.	.	.	.	.	.	.	.	.	.	.	.	.	.	.	.	.	.	.	.	.	.	.	.	.	.	.	.
Werneria spathulata	.	.	.	.	.	.	.	.	.	.	.	.	.	.	.	.	.	.	.	.	.	.	.	.	.	.	.	.	.
Xenophyllum incisum	.	.	.	.	.	.	.	.	.	.	.	.	.	.	.	.	.	.	.	.	.	.	.	.	.	.	.	.	.
Zameioscirpus atacamensis	.	.	.	.	.	.	.	.	.	.	.	.	.	.	.	.	.	.	.	.	.	.	.	.	.	.	1	1	1
Zameioscirpus gaimardiodes	.	.	.	.	.	.	.	.	.	.	.	.	.	.	.	.	.	.	.	.	.	.	1	.	.	.	.	.	.
Zameioscirpus muticus	.	1	.	1	1	1	1	1	1	1	1	.	1	1	1	1	1	1	1	1	1	1	.	1	1	1	.	.	.

**Bog**	**407**	**408**	**409**	**410**	**411**	**412**	**413**	**414**	**415**	**416**	**417**	**418**	**419**	**420**	**421**
**Operational zone**	**T**	**T**	**T**	**T**	**T**	**T**	**T**	**T**	**T**	**T**	**S**	**S**	**S**	**S**	**N**
**Cluster**	**2**	**4**	**5**	**2**	**2**	**2**	**4**	**2**	**2**	**2**	**7**	**7**	**7**	**7**	**9**
**Bioregion**	**T**	**T**	**T**	**T**	**T**	**T**	**T**	**T**	**T**	**T**	**S**	**S**	**S**	**S**	**N**
**Longitude**	**-69.21**	**-69.17**	**-69.2**	**-69.2**	**-70.03**	**-69.19**	**-69.22**	**-70.33**	**-70.4**	**-70.45**	**-70.51**	**-70.51**	**-70.5**	**-70.5**	**-71.32**
**Latitude**	**-29.04**	**-28.43**	**-28.41**	**-28.41**	**-31.52**	**-28.41**	**-28.35**	**-31.55**	**-31.47**	**-31.43**	**-36.02**	**-36.02**	**-36.02**	**-36.02**	**-15.66**
Acaena antarctica	.	.	.	.	.	.	.	.	.	.	.	.	.	.	.
Acaena macrocephala	.	.	.	.	.	.	.	.	.	.	.	.	1	.	.
Acaena magellanica	.	.	.	.	1	.	.	1	.	.	.	.	.	.	.
Acaena ovalifolia	.	.	.	.	.	.	.	.	.	.	.	.	.	.	.
Acaena pinnatifida	.	.	.	.	.	.	.	.	.	.	.	.	.	.	.
Adesmia retusa	.	.	.	.	.	.	.	.	.	.	.	.	.	.	.
Agrostis breviculmis	.	.	.	.	.	.	.	.	.	.	.	.	.	.	.
Agrostis imberbis	.	.	.	.	.	.	.	.	.	.	.	1	1	.	.
Agrostis meyenii	.	.	.	.	.	.	.	.	.	.	1	1	1	.	.
Agrostis perennans	.	.	.	.	.	.	.	.	.	1	.	.	.	.	.
Alchemilla pinnata	.	.	.	.	.	.	.	1	.	.	.	.	.	.	.
Alopecurus magellanicus	.	.	.	.	.	.	.	.	.	.	1	1	1	.	.
Amphiscirpus nevadensis	.	.	.	.	.	1	.	.	.	.	.	.	.	.	.
Anagallis alternifolia	.	.	.	.	.	.	.	.	.	.	.	1	1	.	.
Antennaria chilensis	.	.	.	.	.	.	.	.	.	.	.	.	.	.	.
Anthoxanthum redolens	.	.	.	.	.	.	.	.	.	.	.	.	.	.	.
Apium panul	.	.	.	.	.	.	.	.	.	.	.	.	.	.	.
Arenaria rivularis	.	.	.	.	.	.	.	.	.	.	.	.	.	.	.
Arenaria serpens	.	.	.	.	.	1	.	1	1	1	.	1	1	.	.
Arjona pusilla	.	.	.	.	.	.	.	.	.	.	.	.	.	.	.
Astragalus bustillosii	.	.	.	.	.	.	.	.	.	.	.	.	.	.	.
Astragalus micranthellus	.	.	.	.	.	.	.	.	.	.	.	.	.	.	.
Azolla filiculoides	.	.	.	.	.	.	.	.	.	.	.	.	.	.	.
Azorella boelckei	.	.	.	.	.	.	.	.	.	.	.	.	.	.	.

Bog

	407	408	409	410	411	412	413	414	415	416	417	418	419	420	421
Azorella burkartii	.	.	.	.	.	.	.	.	.	.	.	.	.	.	.
Azorella cryptantha	.	.	.	.	.	.	.	.	.	.	.	.	.	.	.
Azorella lycopodioides	.	.	.	.	.	.	.	.	.	.	.	.	1	1	.
Azorella trifoliolata	.	.	.	.	.	.	.	1	.	.	.	.	.	.	.
Baccharis acaulis	.	.	.	.	.	.	.	.	.	.	.	.	.	.	.
Baccharis caespitosa	.	.	.	.	.	.	.	.	.	.	.	.	.	.	.
Baccharis magellanica	.	.	.	.	.	.	.	.	.	.	.	.	.	.	.
Belloa chilensis	.	.	.	.	.	.	.	.	.	.	.	.	.	.	.
Bromus catharticus	.	.	.	.	.	.	.	.	.	.	.	.	.	.	.
Calandrinia acaulis	.	.	.	.	.	.	.	.	.	.	.	.	.	.	.
Calandrinia compacta	.	1	.	.	.	.	.	.	.	.	.	.	.	.	.
Calceolaria biflora	.	.	.	.	.	.	.	.	.	.	.	1	1	.	.
Calceolaria cana	.	.	.	.	.	.	.	.	.	.	.	.	.	.	.
Calceolaria corymbosa	.	.	.	.	.	.	.	.	.	.	.	1	.	.	.
Calceolaria filicaulis	.	.	.	.	1	.	.	1	1	1	.	.	.	.	.
Callitriche lechleri	.	.	.	.	.	.	.	.	.	.	.	.	.	.	.
Caltha appendiculata	.	.	.	.	.	.	.	.	.	.	.	.	.	.	.
Caltha sagittata	.	.	.	.	.	.	.	.	.	.	1	1	1	1	1
Cardamine cordata	.	.	.	.	.	.	.	.	.	.	.	.	.	.	.
Cardamine glacialis	.	.	.	.	.	.	.	.	.	.	.	.	.	.	.
Cardamine tenuirostris	.	.	.	.	.	.	.	.	.	.	.	.	.	1	.
Cardamine volckmannii	.	.	.	.	.	.	.	.	.	.	.	.	.	.	.
Carex acaulis	.	.	.	.	.	.	.	.	.	.	.	.	.	.	.
Carex atropicta	.	.	.	.	.	.	.	.	.	.	.	1	1	.	.
Carex banksii	.	.	.	.	.	.	.	.	.	.	1	.	.	.	.
Carex caduca	.	.	.	.	.	.	.	.	.	.	.	.	.	.	.
Carex decidua	.	.	.	.	.	.	.	.	.	.	.	.	1	.	.
Carex fuscula	.	.	.	.	.	.	.	.	.	.	.	.	.	.	.
Carex gayana	1	.	.	.	1	1	.	1	1	1	1	1	1	.	.
Carex hypoleucos	.	.	.	.	.	.	.	.	.	.	.	.	.	.	.
Carex macloviana	.	.	.	.	.	.	.	.	.	.	1	1	1	1	.
Carex magellanica	.	.	.	.	.	.	.	.	.	.	1	1	.	.	.
Carex malmei	.	.	.	.	1	.	.	.	.	.	.	.	.	.	.
Carex maritima	1	1	1	.	.	.	1	.	.	.	.	.	.	.	.
Carex microglochin	.	.	.	.	.	.	.	.	.	.	.	.	.	.	.
Carex pleioneura	.	.	.	.	.	.	.	.	.	.	.	.	.	.	.
Carex ruthsatzae	.	.	.	.	.	.	.	.	.	.	.	.	.	.	.

**Bog**	**407**	**408**	**409**	**410**	**411**	**412**	**413**	**414**	**415**	**416**	**417**	**418**	**419**	**420**	**421**
Carex vallis-pulchrae	.	.	.	.	1	.	.	1	.	.	.	.	.	.	.
Carpha schoenoides	.	.	.	.	.	.	.	.	.	.	.	.	.	.	.
Castilleja pumila	.	.	.	.	.	.	.	.	.	.	.	.	.	.	1
Catabrosa werdermannii	.	.	.	.	.	.	.	.	.	.	.	.	.	.	.
Cerastium humifusum	.	.	.	.	1	.	.	.	1	.	.	.	.	.	.
Cerastium montioides	.	.	.	.	.	.	.	.	.	.	.	.	.	.	.
Chiliotrichum diffusum	.	.	.	.	.	.	.	.	.	.	1	1	1	.	.
Chusquea culeou	.	.	.	.	.	.	.	.	.	.	.	.	.	.	.
Colobanthus quitensis	.	.	.	.	.	1	.	1	.	1	.	1	1	1	.
Cortaderia egmontiana	.	.	.	.	.	.	.	.	.	.	1	.	.	.	.
Cotula mexicana	.	.	.	.	.	.	.	.	.	.	.	.	.	.	1
Crassula peduncularis	.	.	.	.	.	.	.	.	.	.	.	.	.	.	.
Cuatrecasasiella argentina	.	.	.	.	.	.	.	.	.	.	.	.	.	.	.
Deschampsia antarctica	.	.	.	.	.	.	.	.	.	.	1	.	.	.	.
Deschampsia caespitosa	.	.	.	.	.	.	.	.	.	.	.	.	1	.	.
Deschampsia patula	.	.	.	.	.	.	.	.	.	.	.	.	.	.	.
Cinnagrostis brevifolia	.	.	.	.	.	.	.	.	.	.	.	.	.	.	.
Deschampsia chrysantha	.	.	.	.	.	.	.	.	.	.	.	.	.	.	.
Cinnagrostis chrysophylla	.	.	.	.	.	.	.	.	.	.	.	.	.	.	.
Deschampsia chrysostachya	.	.	.	.	.	.	.	.	.	1	.	.	.	.	.
Deschampsia eminens	1	.	.	1	.	1	1	1	1	1	.	.	.	.	.
Deschampsia hackelii	.	.	.	.	.	.	.	.	.	.	.	.	.	.	.
Cinnagrostis minima	.	.	.	.	.	.	.	.	.	.	.	.	.	.	.
Deschampsia ovata	.	.	.	.	.	.	.	.	.	.	.	.	.	.	.
Cinnagrostis rigescens	.	.	.	.	.	.	.	.	.	.	.	.	.	.	1
Cinnagrostis spicigera	.	.	.	.	.	.	.	.	.	.	.	.	.	.	.
Cinnagrostis velutina	1	.	1	1	.	1	1	.	1	1	.	.	1	.	.
Cinnagrostis vicunarum	.	.	.	.	.	.	.	.	.	.	.	.	.	.	.
Distichia filamentosa	.	.	.	.	.	.	.	.	.	.	.	.	.	.	.
Distichia muscoides	.	.	.	.	.	.	.	.	.	.	.	.	.	.	1
Distichlis humilis	.	.	.	.	.	.	.	.	.	.	.	.	.	.	.
Distichlis scoparia	.	.	.	.	.	.	.	.	.	.	.	.	.	.	.

**Bog**	**407**	**408**	**409**	**410**	**411**	**412**	**413**	**414**	**415**	**416**	**417**	**418**	**419**	**420**	**421**
Distichlis spicata	.	.	.	.	.	.	.	.	.	.	.	.	.	.	.
Draba pusilla	.	.	.	.	.	.	.	.	.	.	.	.	.	.	.
Eleocharis melanomphala	.	.	.	.	.	.	.	.	.	.	.	.	.	.	.
Eleocharis pseudoalbibracteata	1	.	.	.	1	1	1	1	1	1	.	.	.	.	.
Elodea potamogeton	.	.	.	.	.	.	.	.	.	.	.	.	.	.	.
Empetrum rubrum	.	.	.	.	.	.	.	.	.	.	1	.	1	.	.
Epilobium australe	.	.	.	.	.	.	.	.	.	.	1	1	1	.	.
Epilobium barbeyanum	.	.	.	.	.	.	.	.	.	.	.	.	1	.	.
Epilobium ciliatum	.	.	.	.	.	.	.	.	.	.	.	1	1	.	.
Epilobium denticulatum	.	.	.	.	.	.	.	.	.	.	.	.	.	.	.
Epilobium fragile	.	.	.	.	.	.	.	.	.	.	.	.	.	.	.
Epilobium glaucum	.	.	.	.	.	.	.	.	.	.	.	.	.	.	.
Epilobium nivale	.	.	.	.	.	.	.	.	.	.	.	.	.	.	.
Erigeron andicola	.	.	.	.	.	.	.	.	.	.	.	.	1	.	.
Erigeron leptopetalus	.	.	.	.	.	.	.	.	.	.	.	.	.	.	.
Erigeron myosotis	.	.	.	.	.	.	.	.	.	.	.	1	.	1	.
Erigeron patagonicus	.	.	.	.	.	.	.	.	.	.	.	.	.	.	.
Erythranthe cuprea	.	.	.	.	.	.	.	.	.	.	.	.	.	.	.
Erythranthe depressa	1	.	.	.	1	.	.	1	1	1	.	.	.	.	.
Erythranthe glabrata	.	.	.	.	.	.	.	.	.	.	.	.	.	.	.
Erythranthe lutea	.	.	.	.	.	.	.	.	.	.	.	1	1	.	.
Escallonia virgata	.	.	.	.	.	.	.	.	.	.	1	.	.	.	.
Euphrasia antarctica	.	.	.	.	.	.	.	.	.	.	.	.	.	.	.
Euphrasia chrysantha	.	.	.	.	.	.	.	.	.	.	.	1	.	.	.
Euphrasia subexserta	.	.	.	.	.	.	.	.	.	.	.	1	1	1	.
Festuca hypsophila	.	.	.	.	.	.	.	.	.	.	.	.	.	.	.
Festuca kurtziana	.	.	.	.	.	.	.	.	.	.	.	.	.	.	.
Festuca lilloi	.	.	.	.	.	.	.	.	.	.	.	.	.	.	.
Festuca magellanica	.	.	.	.	.	.	.	.	.	.	1	1	1	.	.
Festuca nardifolia	1	1	1	1	1	.	.	.	.	.	.	.	.	.	.
Festuca rigescens	.	.	.	.	.	.	.	.	.	.	.	.	.	.	.
Festuca werdermannii	.	.	.	.	.	.	.	.	.	.	.	.	.	.	.
Frankenia triandra	.	.	.	.	.	.	.	.	.	.	.	.	.	.	.
Gamocarpha graminea	.	.	.	.	.	.	.	.	.	.	.	.	.	.	.
Gamocarpha ventosa	.	.	.	.	.	.	.	.	.	.	.	.	.	.	.

**Bog**	**407**	**408**	**409**	**410**	**411**	**412**	**413**	**414**	**415**	**416**	**417**	**418**	**419**	**420**	**421**
Gamochaeta chamissonis	.	.	.	.	.	.	.	.	.	.	.	.	.	.	.
Gamochaeta longipedicellata	.	.	.	.	.	.	.	.	.	.	.	.	.	.	.
Gamochaeta neuquensis	.	.	.	.	.	.	.	.	.	.	.	.	1	.	.
Gaultheria antarctica	.	.	.	.	.	.	.	.	.	.	.	.	.	.	.
Gaultheria caespitosa	.	.	.	.	.	.	.	.	.	.	.	1	1	1	.
Gaultheria pumila	.	.	.	.	.	.	.	.	.	.	1	1	1	.	.
Gavilea chica	.	.	.	.	.	.	.	.	.	.	.	.	.	.	.
Gentiana prostrata	1	1	.	1	1	1	.	.	1	1	.	1	1	.	1
Gentianella fiebrigii	.	.	.	.	.	.	.	.	.	.	.	.	.	.	.
Gentianella magellanica	.	.	.	.	.	.	.	.	.	.	1	1	1	1	.
Gentianella multicaulis	.	.	.	.	1	.	.	.	.	.	.	.	.	.	.
Gentianella ottonis	.	.	.	.	.	.	.	.	.	.	.	.	.	.	.
Gentianella primuloides	.	.	.	.	.	.	.	.	.	.	.	.	.	.	.
Gentianella pseudocrassula	.	.	.	.	.	.	.	.	.	1	.	.	.	.	.
Geranium sessiliflorum	.	.	.	.	.	.	.	.	.	.	.	1	1	.	.
Gunnera magellanica	.	.	.	.	.	.	.	.	.	.	1	1	1	.	.
Halenia caespitosa	.	.	.	.	.	.	.	.	.	.	.	.	.	.	.
Halerpestes cymbalaria	.	.	.	.	.	.	.	1	.	1	.	.	.	.	.
Halerpestes exilis	.	.	.	.	.	.	.	.	.	.	.	.	.	.	.
Hieracium antarcticum	.	.	.	.	.	.	.	.	.	.	.	.	.	.	.
Hordeum comosum	.	.	.	.	.	.	.	.	.	.	.	.	.	.	.
Hordeum muticum	.	.	.	.	.	.	.	.	.	.	.	.	.	.	.
Hypochaeris acaulis	.	.	.	.	.	.	.	.	.	.	.	1	1	.	.
Hypochaeris chondrilloides	.	.	.	.	.	.	.	.	.	.	.	.	.	.	.
Hypochaeris meyeniana	.	.	.	.	.	.	.	.	.	.	.	.	.	.	.
Hypochaeris palustris	.	.	.	.	.	.	.	.	.	.	.	.	.	.	.
Hypochaeris taraxacoides	.	.	.	.	.	.	.	.	.	.	.	.	.	.	1
Hypochaeris tenerifolia	.	.	.	.	.	.	.	.	.	.	.	.	.	.	.
Isolepis nigricans	.	.	.	.	.	.	.	.	.	.	.	.	.	.	.
Isolepsis inundata	.	.	.	.	.	.	.	.	.	.	.	.	.	.	.
Juncus balticus	1	.	.	.	1	1	.	1	.	1	1	1	1	.	.

**Bog**	**407**	**408**	**409**	**410**	**411**	**412**	**413**	**414**	**415**	**416**	**417**	**418**	**419**	**420**	**421**
Juncus stipulatus	.	.	.	.	.	.	.	.	.	.	1	1	1	1	.
Koeleria kurtzii	.	.	.	.	.	.	.	1	.	.	.	.	.	.	.
Lachemilla diplophylla	.	.	.	.	.	.	.	.	.	.	.	.	.	.	1
Lachemilla pinnata	.	.	.	.	.	.	.	.	.	.	.	.	.	.	1
Lagenophora nudicaulis	.	.	.	.	.	.	.	.	.	.	.	1	1	.	.
Lemna minuta	.	.	.	.	.	.	.	.	.	.	.	.	.	.	.
Leptinella scariosa	.	.	.	.	.	.	.	.	.	.	.	.	.	.	.
Leucheria candidissima	.	.	.	.	.	.	.	.	.	.	.	.	.	1	.
Leucheria nutans	.	.	.	.	.	.	.	.	.	.	.	.	.	.	.
Lilaea scilloides	.	.	.	.	.	.	.	.	.	.	.	.	.	.	.
Lilaeopsis macloviana	.	.	.	.	.	.	.	.	.	.	.	.	.	.	1
Limosella australis	.	.	.	.	.	.	.	.	.	.	.	.	.	.	.
Lobelia oligophylla	1	.	.	.	1	1	.	1	1	1	1	1	.	.	1
Luzula brachyphylla	.	.	.	.	.	.	.	.	.	.	.	.	.	.	.
Luzula chilensis	.	.	.	.	.	.	.	.	.	.	.	1	1	1	.
Luzula racemosa	.	.	.	.	.	.	.	.	.	.	.	.	.	.	1
Luzula vulcanica	.	.	.	.	.	.	.	.	.	.	.	.	.	.	.
Lysipomia pumila	.	.	.	.	.	.	.	.	.	.	.	.	.	.	.
Marsippospermum philippii	.	.	.	.	.	.	.	.	.	.	.	.	.	.	.
Marsippospermum reichei	.	.	.	.	.	.	.	.	.	.	.	.	.	.	.
Montia fontana	.	.	.	.	.	.	.	.	.	.	.	.	.	.	.
Muhlenbergia asperifolia	.	.	.	.	.	.	.	.	.	.	.	.	.	.	.
Myriophyllum quitense	.	.	.	.	.	.	.	.	1	1	1	.	.	.	.
Myrosmodes nervosa	.	.	.	.	.	.	.	.	.	.	.	.	.	.	.
Myrosmodes paludosa	.	.	.	.	.	.	.	.	.	.	.	.	.	.	.
Myrteola nummularia	.	.	.	.	.	.	.	.	.	.	.	.	.	.	.
Nanodea muscosa	.	.	.	.	.	.	.	.	.	.	.	.	.	.	.
Neobartsia crenoloba	.	.	.	.	.	.	.	.	.	.	.	.	.	.	.
Neobartsia pedicularoides	.	.	.	.	.	.	.	.	.	.	.	.	.	.	.
Neobartsia peruviana	.	.	.	.	.	.	.	.	.	.	.	.	.	.	.
Nertera granadensis	.	.	.	.	.	.	.	.	.	.	.	.	.	.	.
Nicoraepoa andina	.	.	.	.	.	.	.	.	.	.	.	1	1	1	.

Bog	407	408	409	410	411	412	413	414	415	416	417	418	419	420	421
Nicoraepoa pugionifolia	.	.	.	.	.	.	.	.	.	.	.	.	.	.	.
Nicoraepoa subenervis	.	.	.	.	1	.	.	.	.	1	.	.	.	.	.
Nitrophila australis	.	.	.	.	.	.	.	.	.	.	.	.	.	.	.
Nothofagus antarctica	.	.	.	.	.	.	.	.	.	.	.	.	.	.	.
Nototriche rugosa	.	.	.	.	.	.	.	.	.	.	.	.	.	.	.
Ochetophila nana	.	.	.	.	1	.	.	.	.	.	1	.	1	.	.
Olsynium junceum	.	.	.	.	.	.	.	.	.	.	.	1	1	.	.
Oreobolus obtusangulus	.	.	.	.	.	.	.	.	.	.	.	.	.	.	.
Oritrophium limnophilum	.	.	.	.	.	.	.	.	.	.	.	.	.	.	.
Osmorhiza glabrata	.	.	.	.	.	.	.	.	.	.	.	1	.	.	.
Ourisia alpina	.	.	.	.	.	.	.	.	.	.	.	.	.	.	.
Ourisia muscosa	.	.	.	.	.	.	.	.	.	.	.	.	.	.	1
Ourisia ruelloides	.	.	.	.	.	.	.	.	.	.	.	.	.	.	.
Oxychloe andina	1	1	.	.	.	.	1	.	.	.	.	.	1	.	.
Oxychloe bisexualis	.	.	1	1	.	1	.	.	.	.	.	.	.	.	.
Oxychloe castellanosii	.	.	.	.	.	.	.	.	.	.	.	.	.	.	.
Oxychloe haumaniana	1	.	1	.	.	.	1	.	.	.	.	.	.	.	.
Oxychloe mendocina	.	.	.	.	.	.	.	.	.	.	.	.	.	.	.
Patosia clandestina	.	.	.	.	1	.	.	1	1	1	1	1	1	1	.
Perezia capito	.	.	.	.	.	.	.	.	.	.	.	1	.	.	.
Perezia delicata	.	.	.	.	.	.	.	.	.	.	.	.	.	.	.
Perezia fonkii	.	.	.	.	.	.	.	.	.	.	.	.	.	.	.
Perezia pedicularidifolia	.	.	.	.	.	.	.	.	.	.	.	.	.	.	.
Perezia pinnatifida	.	.	.	.	.	.	.	.	.	.	.	.	.	.	.
Petroravenia friesii	.	.	.	.	.	.	.	.	.	.	.	.	.	.	.
Petroravenia werdermannii	.	.	.	.	.	.	.	.	.	.	.	.	.	.	.
Phleum alpinum	.	.	.	.	.	.	.	1	1	.	.	1	1	1	.
Phylloscirpus acaulis	1	.	.	.	1	1	.	.	.	1	1	.	.	.	1
Phylloscirpus boliviensis	.	.	.	.	.	.	.	.	.	.	.	.	.	.	.
Phylloscirpus deserticola	.	.	.	.	.	.	.	.	.	1	.	.	.	.	.
Pinguicula antarctica	.	.	.	.	.	.	.	.	.	.	.	.	.	.	.

**Bog**	**407**	**408**	**409**	**410**	**411**	**412**	**413**	**414**	**415**	**416**	**417**	**418**	**419**	**420**	**421**
Plantago barbata	.	.	.	1	1	1	.	1	1	1	.	1	1	1	.
Plantago rigida	.	.	.	.	.	.	.	.	.	.	.	.	.	.	
Plantago tubulosa	.	.	.	.	.	.	.	.	.	.	.	.	.	.	1
Plantago uniglumis	.	.	.	.	.	.	.	.	.	.	.	.	.	.	.
Poa alopecurus	.	.	.	.	.	.	.	.	.	.	.	.	1	.	.
Poa hachadoensis	.	.	.	.	.	.	.	.	.	.	.	1	1	.	.
Poa perligulata	.	.	.	.	.	.	.	.	.	.	.	.	.	.	.
Polypogon interruptus	.	.	.	.	.	.	.	.	.	.	.	.	.	.	.
Primula magellanica	.	.	.	.	.	.	.	.	.	.	.	.	.	.	.
Puccinellia frigida	.	1	.	.	.	1	.	.	.	.	.	.	.	.	.
Quinchamalium chilense	.	.	.	.	.	.	.	.	.	.	.	.	.	.	.
Ranunculus breviscapus	.	.	.	.	.	.	.	.	.	.	.	.	.	.	.
Ranunculus fuegianus	1	.	.	.	.	.	1	.	.	.	.	.	.	.	.
Ranunculus mandonianus	.	.	.	.	.	.	.	.	.	.	.	.	.	.	.
Ranunculus peduncularis	.	.	.	.	.	.	.	.	.	.	.	1	1	1	.
Ranunculus trichophyllus	.	.	.	.	.	.	.	.	.	.	.	.	.	.	.
Halerpestes uniflora	.	.	.	.	.	.	.	.	.	.	.	.	.	.	.
Rubus geoides	.	.	.	.	.	.	.	.	.	.	.	.	.	.	.
Rumex magellanicus	.	.	.	.	.	.	.	.	.	.	.	.	.	.	.
Rytidosperma lechleri	.	.	.	.	.	.	.	.	.	.	1	.	.	.	.
Sarcocornia pulvinata	.	.	.	.	.	.	.	.	.	.	.	.	.	.	.
Schoenoplectus pungens	.	.	.	.	.	.	.	.	.	.	.	1	1	1	.
Schoenus andinus	.	.	.	.	.	.	.	.	.	.	.	.	.	.	.
Senecio breviscapus	.	.	.	.	.	.	.	.	.	.	.	.	.	.	.
Senecio diemii	.	.	.	.	.	.	.	.	.	.	.	.	.	.	.
Senecio fistulosus	.	.	.	.	.	.	.	.	.	.	1	.	1	.	.
Senecio parodii	.	.	.	.	.	.	.	.	.	.	.	.	.	.	.
Senecio peteroanus	.	.	.	.	.	.	.	.	.	.	.	.	1	.	.
Senecio serratifolius	.	.	.	.	.	.	.	.	.	.	.	.	.	.	.
Senecio trifurcatus	.	.	.	.	.	.	.	.	.	.	.	.	.	.	.
Sisyrinchium chilense	.	.	.	.	.	.	.	.	.	.	.	.	.	.	.
Sisyrinchium patagonicum	.	.	.	.	.	.	.	.	.	.	.	1	.	.	.

Bog	407	408	409	410	411	412	413	414	415	416	417	418	419	420	421
Sisyrinchium pearcei	.	.	.	.	.	.	.	.	.	.	.	.	.	.	.
Stellaria debilis	.	.	.	.	.	.	.	.	.	.	.	.	.	.	.
Stuckenia filiformis	.	1	.	.	.	.	1	.	.	.	1	.	.	.	.
Stuckenia striata	.	.	.	.	.	.	.	.	.	.	.	.	.	.	.
Symphyotrichum peteroanum	.	.	.	.	.	.	.	.	.	.	.	.	.	.	.
Symphyotrichum vahlii	.	.	.	.	.	.	.	.	.	.	1	1	1	.	.
Tetroncium magellanicum	.	.	.	.	.	.	.	.	.	.	.	.	.	.	.
Tribeles australis	.	.	.	.	.	.	.	.	.	.	.	.	.	.	.
Trifolium amabile	.	.	.	.	.	.	.	.	.	.	.	.	.	.	.
Trifolium polymorphum	.	.	.	.	.	.	.	.	.	.	.	.	.	.	.
Triglochin concinna	.	.	.	.	.	.	.	.	.	.	.	.	.	.	.
Triglochin palustris	.	.	.	.	.	1	.	.	.	.	.	.	.	.	.
Triglochin striata	.	.	.	.	.	.	.	.	.	.	1	.	.	.	.
Trisetum caudulatum	.	.	.	.	.	.	.	.	.	.	.	.	.	.	.
Trisetum preslei	.	.	.	.	.	.	.	.	.	.	.	.	.	.	.
Koeleria spicata	.	.	.	.	.	.	.	.	.	.	.	.	.	.	.
Utricularia gibba	.	.	.	.	.	.	.	.	.	.	.	.	.	.	.
Vahlodea atropurpurea	.	.	.	.	.	.	.	.	.	.	.	.	.	.	.
Valeriana fonckii	.	.	.	.	.	.	.	.	.	.	.	.	.	.	.
Valeriana macrorhiza	.	.	.	.	.	.	.	.	.	.	.	1	1	.	.
Viola pygmaea	.	.	.	.	.	.	.	.	.	.	.	.	.	.	.
Werneria apiculata	.	.	.	.	.	.	.	.	.	.	.	.	.	.	.
Werneria pinnatifida	.	.	.	.	.	1	.	.	.	.	.	.	.	.	1
Werneria pygmaea	1	.	.	1	1	1	.	.	1	1	.	.	.	.	1
Werneria solivifolia	.	.	.	.	.	.	.	.	.	.	.	.	.	.	1
Werneria spathulata	.	.	.	.	.	.	.	.	.	.	.	.	.	.	.
Xenophyllum incisum	.	.	.	.	.	.	.	.	.	.	.	.	.	.	.
Zameioscirpus atacamensis	.	.	.	.	.	.	.	.	.	.	.	.	.	.	.
Zameioscirpus gaimardiodes	.	.	.	.	1	.	.	.	.	.	.	.	.	.	.
Zameioscirpus muticus	1	1	.	1	.	1	1	1	1	1	.	.	.	.	.

Additionally: **Bog 421:** *Arenaria digyna; Azorella diapensioides; Cotula australis; Cuatrecasasiella isernii; Festuca orthophylla; Gentianella potamophila; Microsteris gracilis; Ranunculus flagelliformis; Trichophorum rigidum.*
