# Supplementary figures and images for: Environmental Filters Structure Cushion Bogs’ Floristic Composition along the Southern South American Latitudinal Gradient
Source: Plants (Basel). 2024 Aug 9;13(16):2202. doi: 10.3390/plants13162202 (PMC11359879; doi:10.3390/plants13162202)

## NORTH

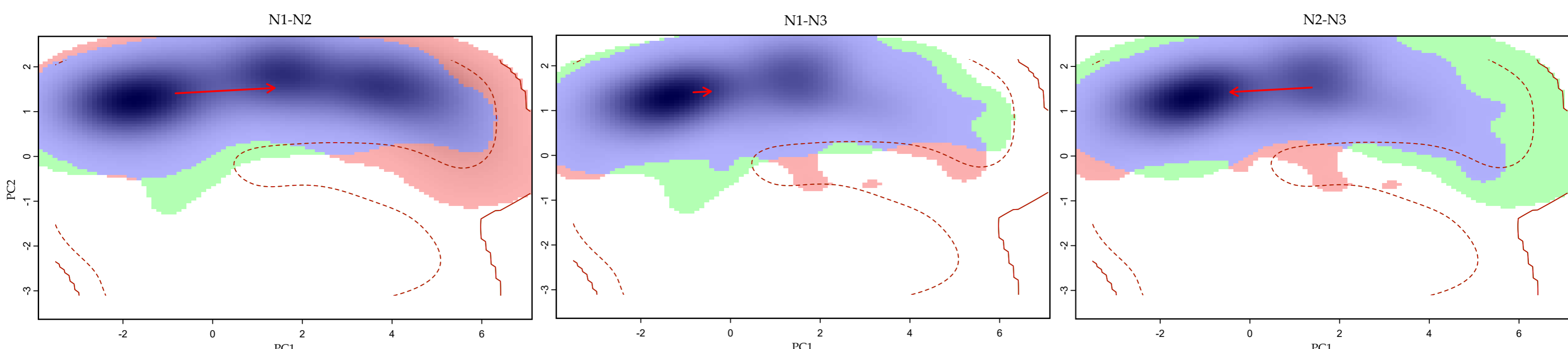

## TRANSITION

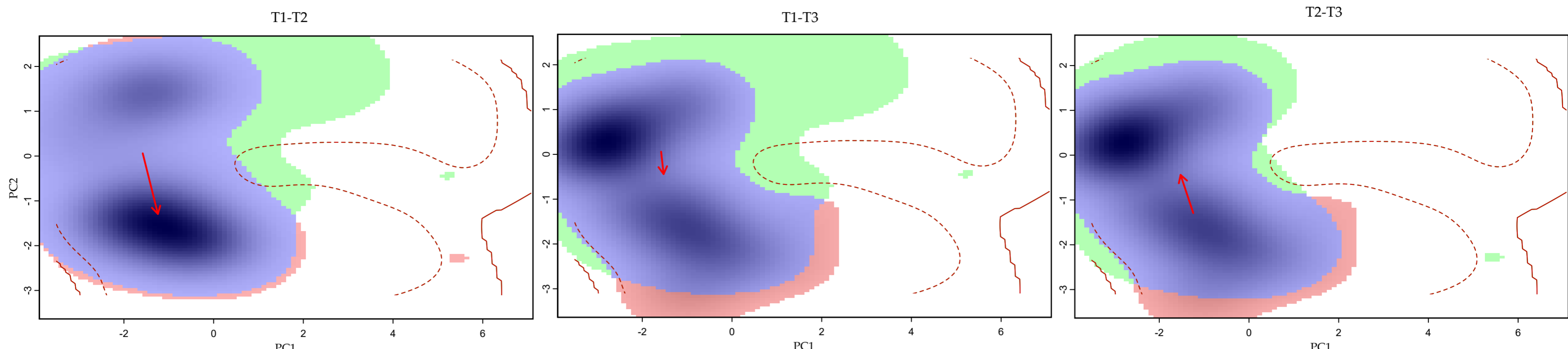

## SOUTH

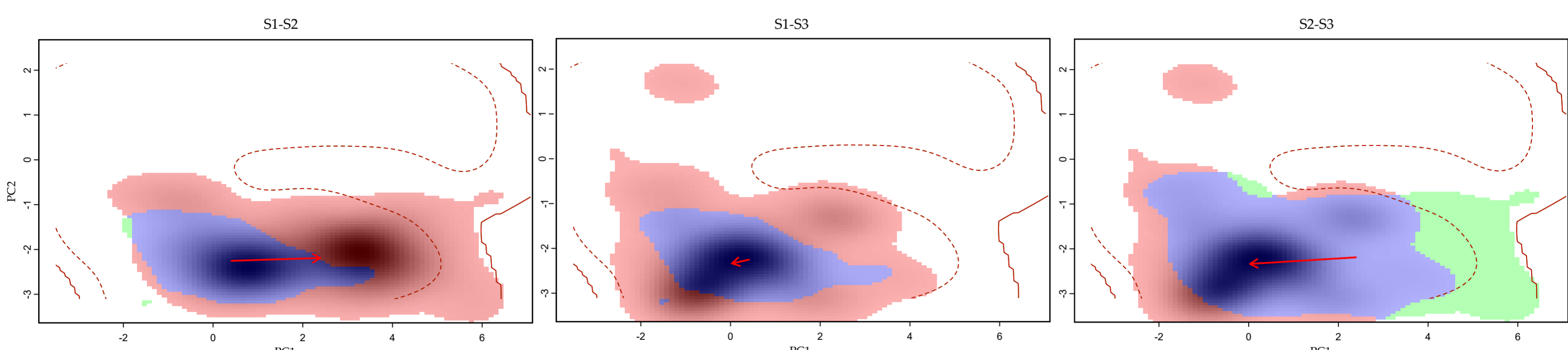

## NORTH-SOUTH

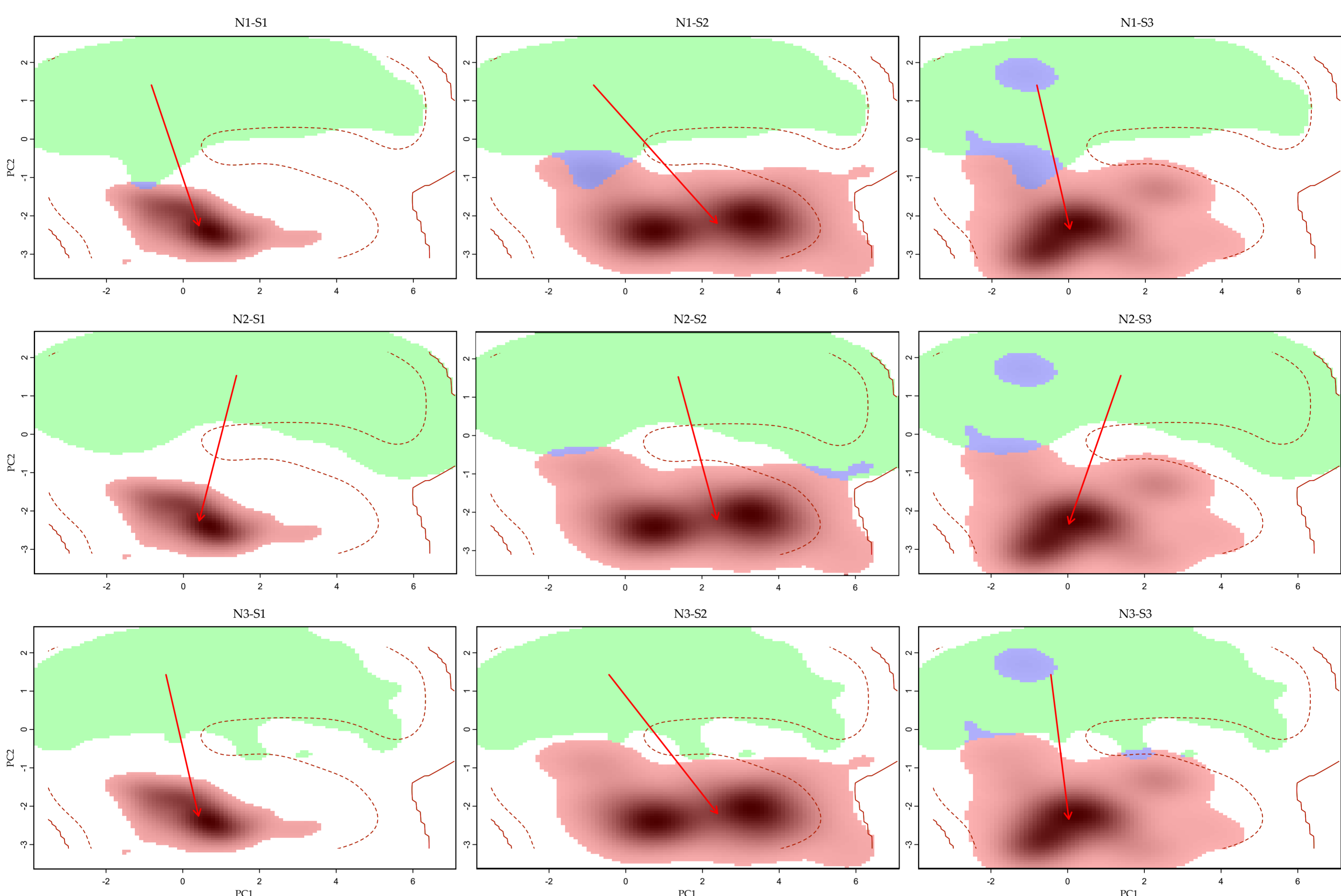

## NORTH-TRANSITION

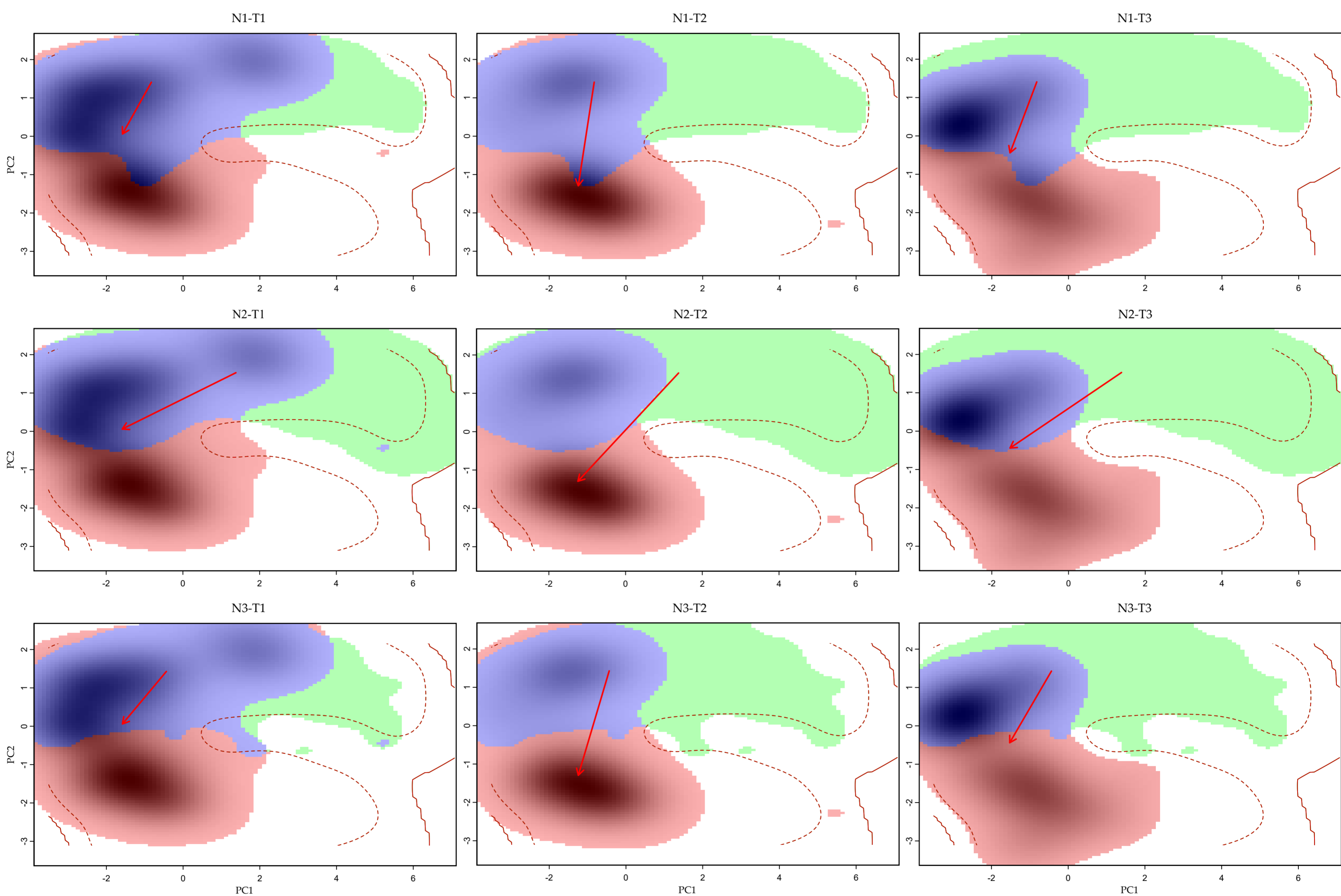

## SOUTH-TRANSITION

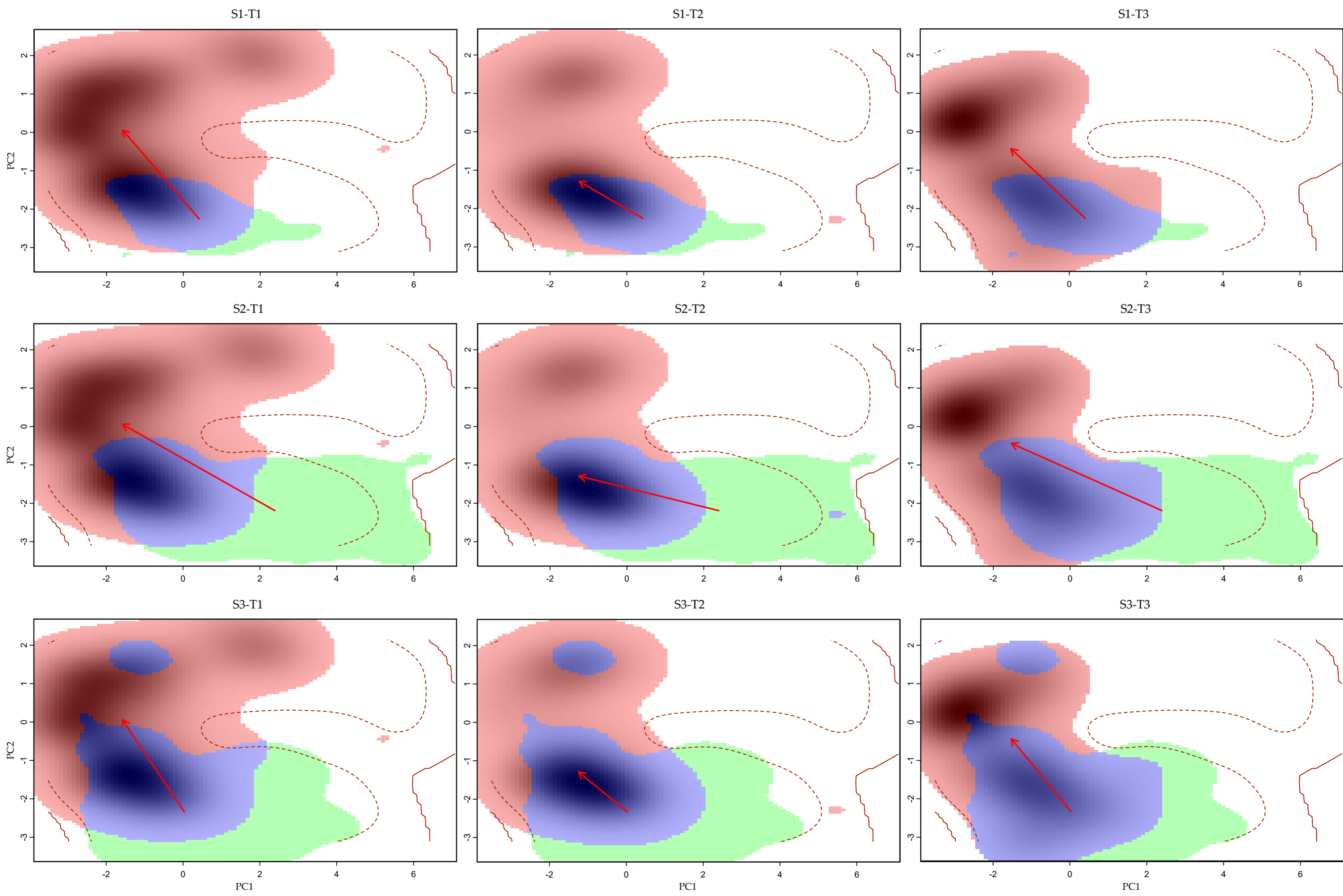

Supplement: Supplementary file 1 [file plants-13-02202-s001.zip › Figure S1.pdf]

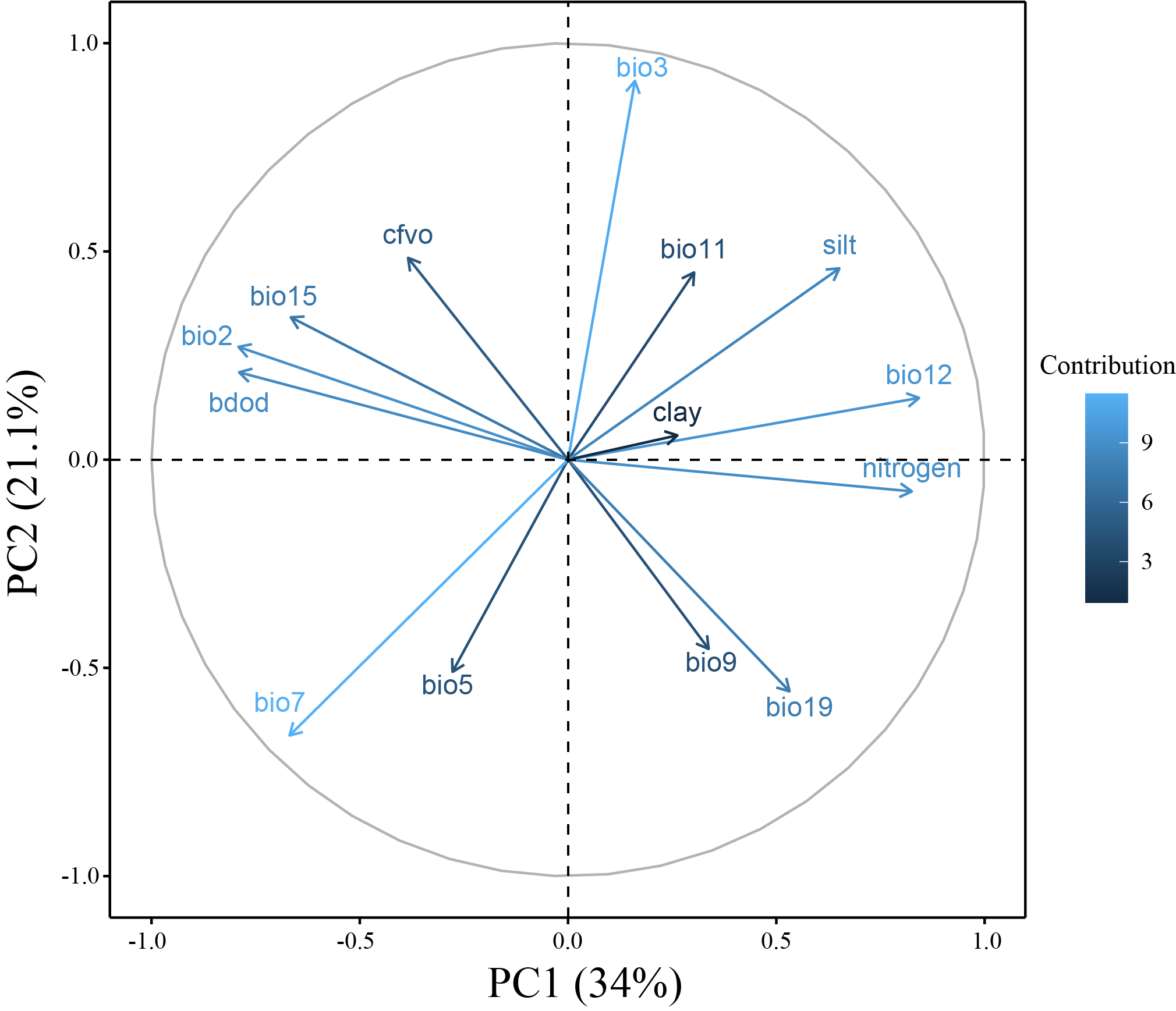

Supplement: Supplementary file 1 [file plants-13-02202-s001.zip › Figure S2.png]
